# Proteome-wide target profiling of α-helix mimetics

**DOI:** 10.1039/d6cb00065g

**Published:** 2026-07-21

**Authors:** Amrita Date, Archie Wall, Hannah Kiely-Collins, Theo Flack, Jack W. Houghton, Jianan Lu, Adam M. Thomas, Peiyu Zhang, Andrew J. Wilson, Edward W. Tate, Anna Barnard

**Affiliations:** a Department of Chemistry, Molecular Sciences Research Hub, Imperial College London 82 Wood Lane London W12 0BZ UK a.barnard@imperial.ac.uk; b Astbury Centre for Structural Molecular Biology, University of Leeds Woodhouse Lane Leeds LS2 9JT UK; c School of Chemistry, University of Leeds Woodhouse Lane Leeds LS2 9JT UK; d School of Chemistry, University of Birmingham, Edgbaston Birmingham B15 2TT UK; e The Francis Crick Institute London NW1 1AT UK

## Abstract

The dysregulation of protein–protein interactions (PPIs) in disease states is well established, yet they are challenging to target, owing to the large surface area and featureless nature of protein binding interfaces. For targeting helix-mediated interactions, α-helix mimetics present a promising strategy. These are versatile small molecule scaffolds, capable of mimicking the hotspot residues on an α-helix. A wide range of such scaffolds have been reported, yet their target protein selectivity in the context of a whole proteome requires further exploration. Here, we report the affinity-based protein profiling of three structurally distinct classes of α-helix mimetics, *N*-substituted oligobenzamides, pyrrolopyrimidines, and oxopiperazines. This represents the first direct cross-comparison of different helix mimetic scaffolds, revealing significant differences in proteome-wide selectivity.

## Abstract 2

Proteome-wide target profiling, carried out using photoactivatable probes of MDM2-targeting α-helix mimetics shows that those with a pyrrolopyrimidine backbone are least selective, while the oligobenzamide probe exhibits highest selectivity.

## Graphical abstract

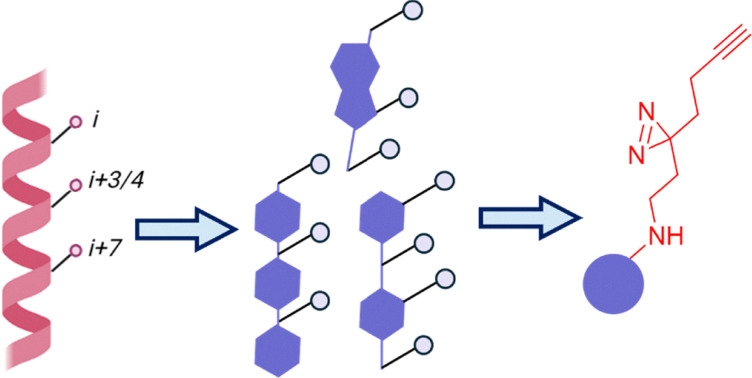

The human interactome is predicted to be composed of approximately 650 000 protein–protein interactions (PPIs).^1^ Despite their involvement in diverse disease states, they have long been considered ‘undruggable’. Unlike small molecule binding sites, which are small (300–500 Å^2^) and deep, PPI interfaces are often large (1000–2000 Å^2^), flat, and featureless.^1,2^ Despite this, a small number of amino acids, termed hotspot residues, contribute the bulk of the binding free energy of the interactions, making them more feasible to target.^1,3,4^ The interactions between proteins are typically mediated by secondary structure elements,^5^ with α-helices mediating a significant number.^6,7^

α-Helix mimetics are synthetic scaffolds that project hotspot side chain-mimicking groups towards a complementary binding interface to modulate PPIs.^5^ They offer opportunities to target helical interfaces using lower molecular weight compounds, while overcoming the challenges posed by the proteolytic liability, polarity, and cell permeability of peptides.^2^ A wide range of α-helix mimetic scaffolds have been reported, with many offering opportunities for modular synthesis and the generation of combinatorial libraries, enabling modification to target a range of PPIs.^5,8–15^

The interaction between the tumour suppressor protein p53 and the E3 ubiquitin protein ligase, murine double minute 2 (MDM2) is a widely studied PPI with great potential as an oncological target.^16–19^ This interaction has served as a model for the development of a number of peptidomimetic modalities, owing to the high affinity of the interaction.^8,20^ MDM2 inhibition has been achieved with α-helix mimetics composed of terphenyl,^21,22^ oligobenzamide,^12,23–25^ benzodiazepine,^26^ oxopiperazine,^10^ pyrrolopyrimidine,^9^ and spiroligomer scaffolds,^27^ among others.^8^ These molecules incorporate groups mimicking the Phe19, Trp23, and Leu26 hotspot residues of the p53 N-terminus that mediate MDM2 binding.^28^ Despite their potential as PPI modulators, there have been concerns that the structural simplicity of α-helix mimetics and hydrophobicity may limit the selectivity that can be achieved.^8^ Previous reports of the selectivity have only assessed binding to a limited number of alternate protein targets.^8,29^ Given that these molecules target an extensively studied PPI and the diversity of helix mimetic scaffolds that have been explored to target it, it was chosen as a model system to enable the comparison of these scaffolds.

We sought to interrogate the global target profile of three representative, structurally diverse classes of α-helix mimetic MDM2 inhibitors for the first time, through an affinity-based protein profiling (AfBPP) approach.^30^ Helix mimetics bearing *N*-substituted oligobenzamide, pyrrolopyrimidine, and oxopiperazine scaffolds were selected for analysis. Molecules of the oligobenzamide family have previously been evaluated in cell-based assays, validated for p53-dependent activity, and assessed for binding to anticipated off-targets.^12,31^ The pyrrolopyrimidine scaffold bears structural resemblance to privileged scaffolds such as purines and indoles, therefore, this class was predicted to exhibit favourable aqueous solubility and cell permeability.^9^ Molecules of this family have been reported to act as dual inhibitors of MDM2 and its homologue, murine double minute X (MDMX).^9^ The oxopiperazine moieties making up the third class of selected α-helix mimetics are also privileged scaffolds in drug discovery, so these molecules were anticipated to exhibit more ‘drug-like’ properties.^8,32^ While data on MDM2 inhibitors of this class remains limited, oxopiperazine helix mimetics targeting other PPIs have been tested in *in vivo* models.^10,33^ To investigate the target profile of the selected α-helix mimetics, photoactivatable-clickable probes of the selected scaffolds were synthesised and validated for application to liquid chromatography-tandem mass spectrometry (LC-MS/MS)-based target profiling.

The most potent reported MDM2 inhibitor parent scaffolds (OB-a, OB-b, PP, and OP) were selected for assessment.^9,10,12^ At least two structurally distinct fully functionalised photoaffinity probes of each molecule were synthesised, introducing a photoactivatable diazirine moiety and clickable alkyne group at different positions of the scaffold (Fig. 1). The R-groups, which form the key binding motifs, were left unaltered to ensure that target protein binding was not affected. Oligobenzamide probes were synthesised by introducing the modifications on the N- and C-termini (OB-a1, OB-a2, OB-b1, and OB-b2), as well as on the non-binding face of the middle monomer (OB-a3 and OB-b3). Modifications were introduced on the non-binding face (PP-1) and at the N-terminus (PP-2) of the pyrrolopyrimidine, while the oxopiperazine was modified at the N- and C-termini (OP-1 and OP-2).

**Fig. 1:**
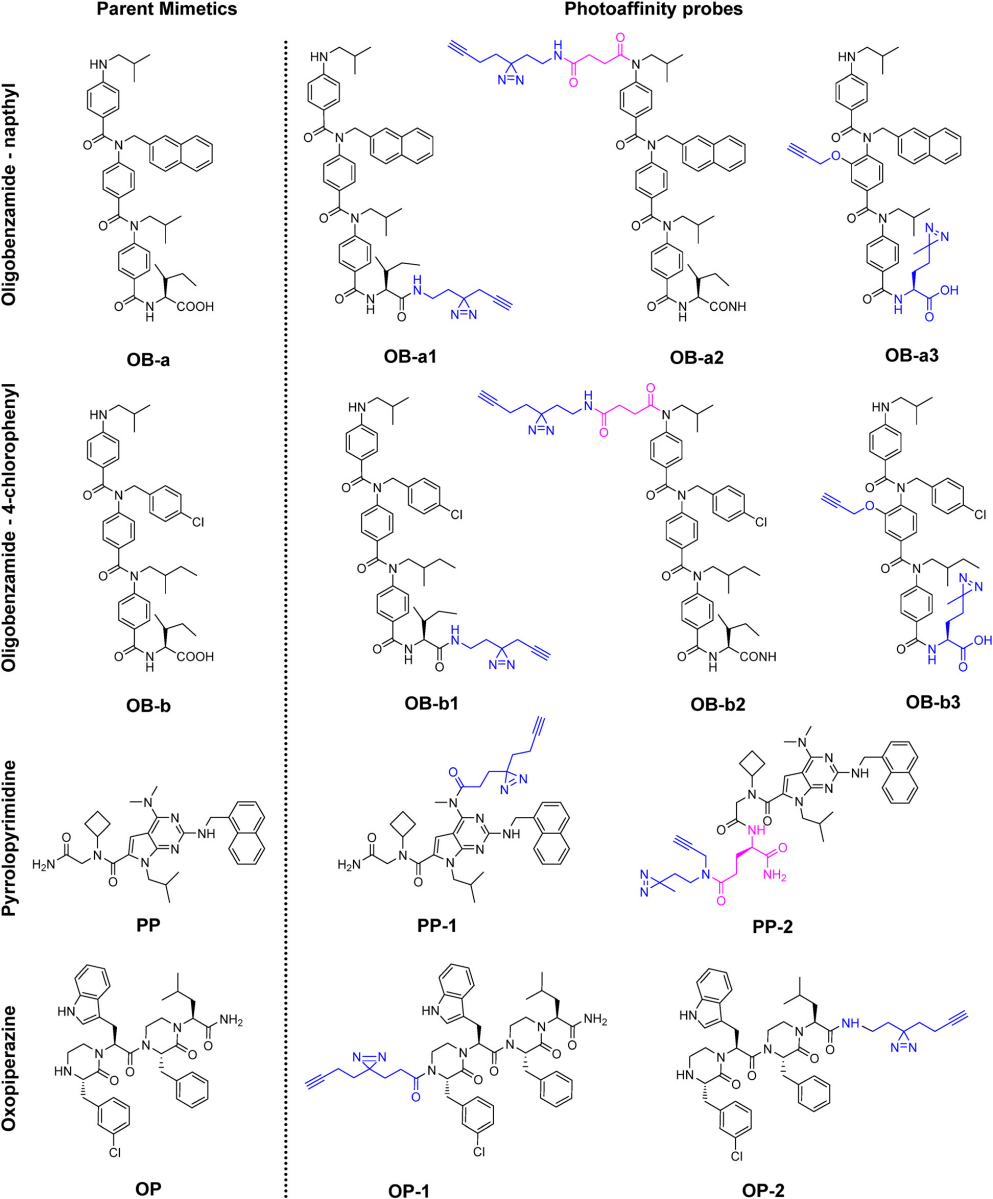
Structures of selected α-helix mimetic MDM2 inhibitors (left) and their corresponding fully functionalised photoaffinity probes (right). Alkyne-diazirine modifications are shown in blue and linkers shown in pink.

The synthesised probes were then validated for MDM2 binding through a fluorescence anisotropy competition binding assay (Fig. S1). Good retention of binding affinity of the parent molecules was observed with OB-a3, OB-b3, PP-1, and PP-2, while a slight reduction in affinity was detected with OP-1 and OP-2. The probes were then validated for retention of downstream phenotypic activity by quantifying their effect on cell viability *via* a colorimetric assay using 3-(4,5-dimethylthiazol-2-yl)-5-(3-carboxymethoxyphenyl)-2-(4-sulfophenyl)-2*H*-tetrazolium (MTS) reagent (Fig. 2A and Fig. S2). Activity was assessed in SJSA-1 cells, which express high levels of MDM2.^34^ As expected from the *in vitro* binding data, OB-a3, OB-b3, PP-1, and PP-2 exhibited comparable EC_50_ values to their parent molecules. Despite the reduction in the MDM2 binding affinity, OP-1 was found to be 17-fold more potent than its parent molecule in cells, while OP-2, which showed comparable MDM2 binding, was inactive in cells. The assay results were corroborated by live cell imaging experiments (Fig. S2).

**Fig. 2:**
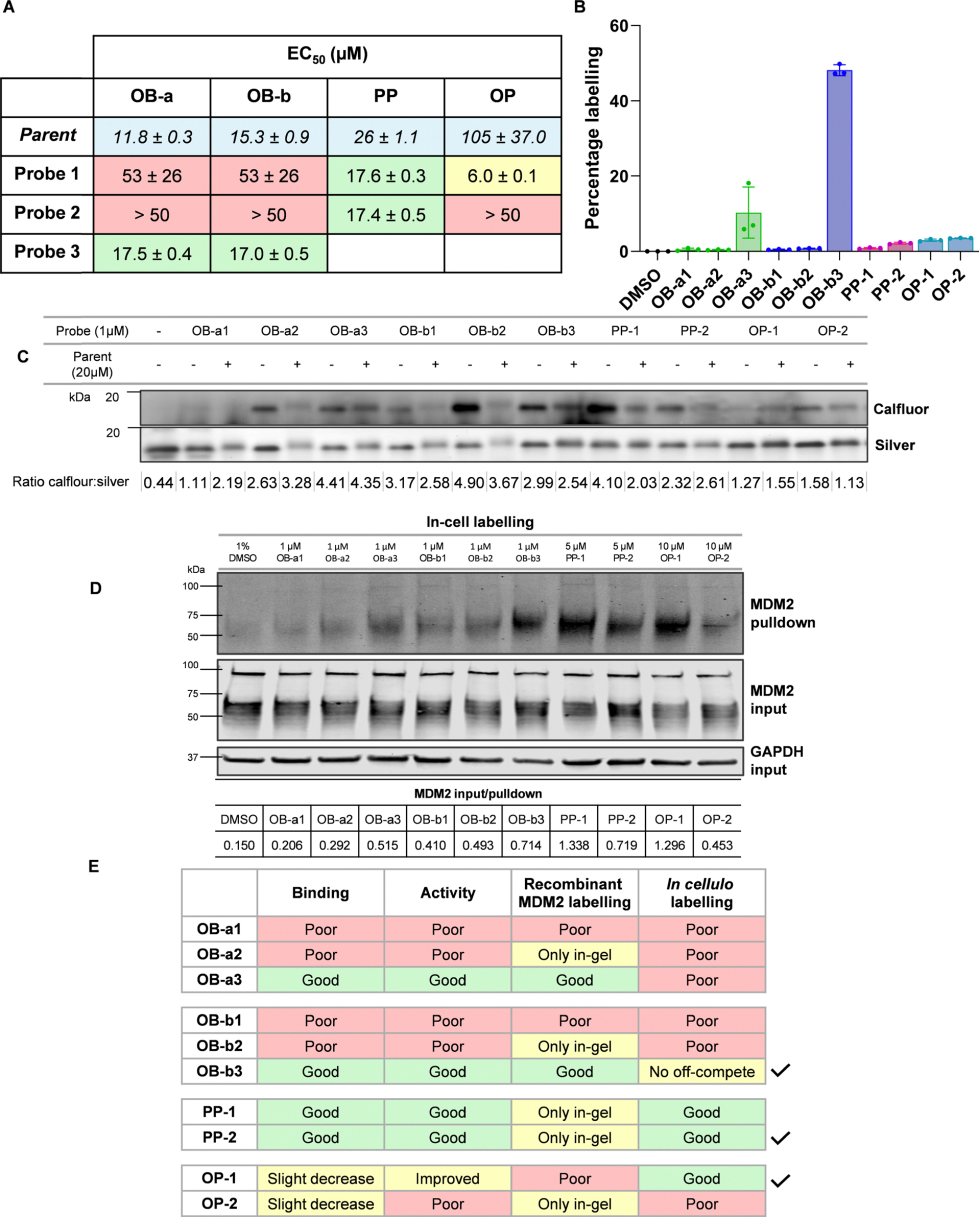
Validation of fully functionalised probes. (A) EC_50_ values determined by an MTS assay conducted 3 days after treatment of SJSA-1 cells with probes (mean ± SEM, *n* = 3) for the parent molecules, probe 1 (OB-a1, OB-b1, PP-1, OP-1), probe 2 (OB-a2, OB-b2, PP-2, OP-2), probe 3 (OB-a3, OB-b3) (B) Covalent labelling of recombinant MDM2 assessed by intact protein MS (*n* = 3). (C) Covalent labelling of recombinant MDM2 assessed *via* in-gel fluorescence through CuAAC with Calfluor-647 azide. Gel silver stained as a protein loading control. Bands quantified using ImageJ. (D) Western blot showing the labelling and pulldown of MDM2 using fully functionalised probes in SJSA-1 cells. Bands quantified using ImageJ. (E) Summary of probe validation data informing the choice of photoaffinity probe for application in AfBPP experiments.

Labelling of recombinant MDM2 was then evaluated by treatment with probe, followed by irradiation at 365 nm for 10 min, and analysis by intact protein mass spectrometry (MS). Promising levels of MDM2 labelling were observed with oligobenzamide probes OB-a3 and OB-b3, which labelled 10% and 48% of the protein, respectively (Fig. 2B). However, the pyrrolopyrimidine and oxopiperazine probes only achieved 1–3.6% protein labelling. This may arise from a number of factors: (i) the differences in MDM2 binding affinity, (ii) the impact of the alkyne-diazirine modifications on probe binding, and (iii) the orientation of the diazirines relative to the protein side chains for covalent crosslinking.

The availability of the alkyne handle for copper-catalysed alkyne-azide cycloaddition (CuAAC) following photo-crosslinking was tested through reaction of the probe-labelled protein with Calfluor647-azide. The protein was resolved by sodium dodecyl sulphate-polyacrylamide gel electrophoresis (SDS-PAGE) and imaged for in-gel fluorescence to detect protein labelling (Fig. 2C). As predicted based on the intact protein MS results, a strong fluorescent band was observed for samples treated with OB-a3 and OB-b3. When treated with an excess of the parent molecule, a reduction in band intensity was observed for OB-b3, but not OB-a3. Despite the poor labelling observed through MS, a fluorescent band and good off-compete were observed with PP-1, PP-2, and OP-2 through in-gel fluorescence. Significant labelling was not detected in samples treated with OP-1.

In-cell labelling was then validated by treatment of SJSA-1 cells with the probes, followed by irradiation at 365 nm, cell lysis, and reaction of the probe-labelled proteins with azide-TAMRA-biotin to allow enrichment onto Neutravidin agarose resin. The isolated proteins were analysed by Western blot to validate the presence of MDM2 (Fig. 2D and Fig. S3). The lowest treatment concentration at which MDM2 labelling could be detected by Western blot using a probe of a particular scaffold was selected for all probes in that family, *e.g.* MDM2 labelling was observed at 1 µM using OB-b3, therefore all oligobenzamide probes were tested at a 1 µM concentration. Effective MDM2 labelling was observed with OB-b3, PP-1, PP-2, and OP-1. A reduction in MDM2 enrichment in the presence of excess parent molecule was observed with PP-1, PP-2, and OP-1, but not with OB-b3, which resulted in a higher degree of overall protein labelling (Fig. S4). This was thought to be an artefact of increased membrane permeability, resulting from initiation of cell death induced by the presence of a cytotoxic molecule.

Based on these validation results, OB-b3, PP-2, and OP-1 were selected for application to AfBPP workflows (Fig. 2E). OB-b3 and OP-1 were the only members of their respective families to effectively label MDM2 in live cells. PP-1 and PP-2 performed comparably through the validation pipeline, however PP-2 appeared less promiscuous than PP-1, judged by the overall in-cell protein labelling detected by in-gel fluorescence from TAMRA (Fig. S3).

A null probe (NP), composed of an alkyne-diazirine minimalist tag attached to a phenyl ring, was synthesised as a negative control (Fig. S5A).^35^ This molecule was validated to ensure that it did not bind to MDM2, impact cell proliferation, or label MDM2 as recombinant protein or in cells. Its target profile was characterised (Fig. S5B–F) and encouragingly, overlap with well-reported diazirine off-targets was noted (Fig. S5G).^36^ The target profile of the three selected probes was then interrogated. SJSA-1 cells were treated with the probes, irradiated at 365 nm, and lysed to extract proteins, which were subjected to CuAAC and azide-biotin enrichment on Neutravidin agarose resin. The isolated proteins were digested using trypsin and analysed by LC-MS/MS (Fig. 3A–C). Probe concentrations were selected based on the minimum concentration required to detect MDM2 labelling *via* Western blot *i.e.* OB-b3 (1 µM), PP-2 (5 µM) and OP-1 (10 µM). PP-2 was found to be most promiscuous, while OB-b3 and OP-1 exhibited comparable selectivity (Fig. 3D) Of the 4252 total proteins quantified, 2565 were found to be significantly enriched (fold change ≥ 2, *p* ≤ 0.01) by PP-2, while OB-b3 and OP-1 significantly labelled 1742 and 1831 proteins, respectively. It is worth noting that, the differences in probe concentration used for each class of α-helix mimetics may also have contributed to the number of proteins labelled. OB-b3 was in fact most selective. However, PP-2 was found to be significantly more promiscuous than OP-1 despite being applied at half the concentration.

The top 25% significantly enriched proteins were selected for further analysis. While MDM2 was found to be significantly enriched by all three probes, it was among the top 25% only for OB-b3, consistent with the significantly higher degree of recombinant protein labelling. A large overlap was observed in the top 25% enriched proteins between probes (Fig. S5H). Gene enrichment analysis using Metascape revealed involvement of these proteins in membrane organisation and protein localisation to organelles, indicating that the labelling of these proteins may be an artefact of cellular localisation or aggregation with cell membranes.

While this allowed us to gauge the differences in selectivity of the probes, it did not enable filtering for targets of the inhibitors themselves. To account for this, competition AfBPP experiments were conducted, facilitating the identification of proteins from which the probes could be displaced by their parent inhibitors. Here, cells were concurrently treated with the probe and the parent compound. The parent compound was used at a two-fold higher concentration compared to the probe in order to ensure that a sufficient excess was present to displace the probe from potential binding sites, while also ensuring solubility and avoiding cytotoxic effects at high treatment concentrations. High confidence hits were identified from the data obtained based on whether they were significantly enriched in the probe-treated condition compared to the competition (fold change ≥ 2, *p* ≤ 0.01) and significantly enriched (fold change ≥ 2, *p* ≤ 0.01) in the probe-treated condition compared to the parent control.

The oligobenzamide probe, OB-b3, as anticipated based on in-gel fluorescence data, demonstrated a higher degree of protein labelling in the competition compared to the probe-treated condition (Fig. 3D and Fig. S6A) consistent with our observation of a lack of off-compete (Fig. 2E). As a result, only six proteins were observed to be significantly enriched but the small number of hits is not a true reflection of the selectivity of this molecule. MDM2 was, however, significantly enriched upon OB-b3 treatment compared to the parent control. The pyrrolopyrimidine probe, PP-2, resulted in 56 hits, significantly more than those observed with the two other scaffolds (Fig. 3E and Fig. S6B). Of these, nine were not significantly enriched compared to NP so were disregarded. Despite being observed through Western blot analysis, MDM2 was not found to be enriched compared to the parent inhibitor. Oxopiperizine OP-1 resulted in 32 statistically significant hits, of which only 11 were significantly enriched compared to NP (Fig. 3F and Fig. S6C). Compared to the parent control, MDM2 was found to be significantly enriched by the probe. However, the off-compete observed was not statistically significant.

**Fig. 3:**
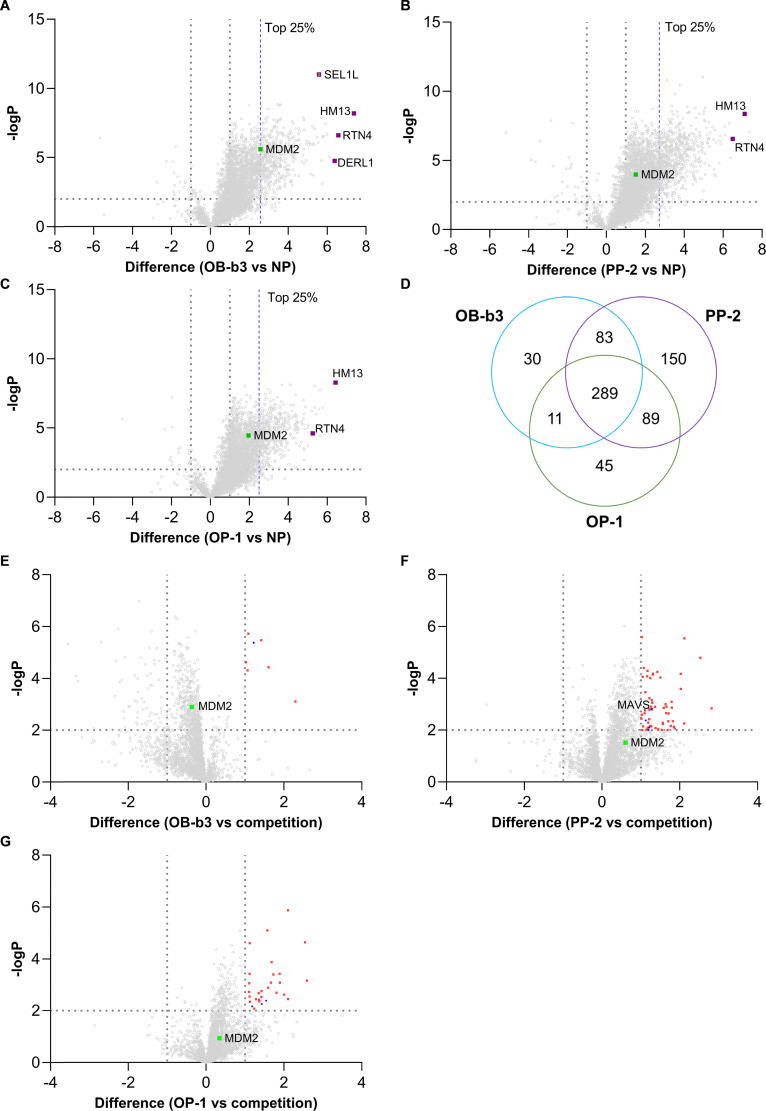
(A, B, C) Target engagement profile of OB-b3, PP-2, and OP-1 in SJSA-1 cells. Volcano plots showing differences in enrichment (*x*-axis) between live cells treated with 1 µM OB-b3/5 µM PP-2/10 µM OP-1 (right) *versus* 20 µM NP as a negative control (left). Associated significance (*y*-axis) is determined by paired Student's *t*-test (FDR = 0.05, S0 = 0.1, *n* = 4). MDM2 is highlighted in green and other proteins of interest are highlighted in purple. Total proteins quantified = 4252. Blue line indicates cut-off for top 25% of enriched proteins by fold change. (D) Venn diagram summarising overlap in the top 25% of statistically significant hits (fold change ≥ 2, *p* ≤ 0.01) for each probe relative to a null probe control. (E, F, G) Volcano plots showing differences in enrichment (*x*-axis) between live cells treated with (E) 1 µM OB-b3 (right) *versus* 1 µM OB-b3 + 2 µM OB-b (left) (F) 5 µM PP-2 (right) *versus* 5 µM PP-2 + 10 µM PP (left), (G) 10 µM OP-1 (right) *versus* 10 µM OP-1 + 20 µM OP (left). Associated significance (*y*-axis) is determined by paired Student's *t*-test (FDR = 0.05, S0 = 0.1, *n* = 4). Statistically significant hits identified are highlighted in red and hits that appear significant relative to the competition control but have been disregarded due to insignificant labelling are shown in blue. Proteins of interest highlighted in purple. Total proteins quantified = 2663 (E), 2502 (F), 2663 (G).

One of the proteins most significantly enriched by all three probes in comparison to NP was histocompatibility minor 13 (HM13). However, this did not appear as a statistically significant hit relative to a competition control. Like MDM2, inhibition of activity of this membrane protein is reported repress tumour progression through upregulation of the p53 pathway.^37^ Reticulon 4 (RTN4), was also among the proteins most significantly enriched by all three probes. Nogo-B, an isoform of RTN4, is reported to interact with antiapoptotic proteins, Bcl-2 and Bcl-x_L_,^38,39^ which themselves are involved in several α-helix mediated PPIs.^40^ The observed enrichment of mitochondrial antiviral signalling protein (MAVS) by PP-2 relative to a competition control was particularly interesting, since it functions in competition with MDM2, associating directly with p53 to block its ubiquitination and upregulate its activity.^41^ Given its direct reported interaction with p53, off-target labelling by an MDM2-directed probe is perhaps not entirely surprising. Similarly, the appearance of SWI/SNF related BAF chromatin remodelling complex subunit D2 (SMARCD2) as a binder of PP-2 was somewhat foreseeable, since it also possesses the SWIB domain, which is the region of MDM2 responsible for p53 binding.^42^ In the case of OB-b3, the most significantly enriched proteins relative to NP were derlin-1 (DERL1)^43^ and protein sel-1 homolog 1 (SEL1L),^44^ both components of the ER-associated degradation (ERAD) pathway, a process responsible for ensuring appropriate protein folding.^45^ This process involves the recognition of misfolded proteins, their ubiquitination, and consequent degradation.^46^ The labelling of multiple components of this pathway suggests the potential targeting of this pathway by OB-b3.

Overall, and not unsurprisingly, all three molecules were found to be more promiscuous than potent small molecule MDM2 inhibitors idasanutlin and navtemadlin, which have been reported to significantly label only 2–6 proteins in a single AfBPP experiment.^35,47^ However, key differences exist between the assessment of selectivity of high-affinity small molecule inhibitors and molecules that are less well-optimised. Primarily, significantly higher probe concentrations are required to achieve effective target protein labelling, meaning that more opportunities exist for off-target labelling events. As a result, it is difficult to determine whether the high levels of protein labelling by certain molecules tested here were a result of poor selectivity or of limited affinity for the target protein. Additionally, the high probe concentrations restrict the fold-excess of parent molecule that can be used to off-compete the probes; particularly when the probes exhibit comparable binding affinity to the parent molecule, a 2-fold excess may not be sufficient to control for the identification of proteins from non-specific labelling events. The application of more stringent competition controls, however, would require further optimisation of these scaffolds to first achieve higher affinity for the target protein.

To further gauge the effect of the selected MDM2 inhibitors on the p53 pathway, the whole proteome of a parent α-helix mimetic-treated SJSA-1 cells was evaluated through bottom-up proteomics (Fig. 4). Surprisingly, no p53-downstream proteins, such as p21, were upregulated upon treatment with any of the helix mimetic parent molecules. The changes in the global proteome were far more subtle than those induced by well-established MDM2 inhibitors.^47–49^ All three inhibitors were found to upregulate the expression of membrane trafficking protein rabenosyn-5 (RBSN) and the kinase/ATPase RIO kinase 1 (RIOK1).^50,51^ RIOK1 induces p53 degradation through the phosphorylation and activation of G3BP stress granule assembly factor 2 (G3BP2),^50^ so its upregulated activity may be competing with the activation of p53 achieved through MDM2 inhibition, resulting in a nullified net effect on the expression of its downstream proteins.

**Fig. 4:**
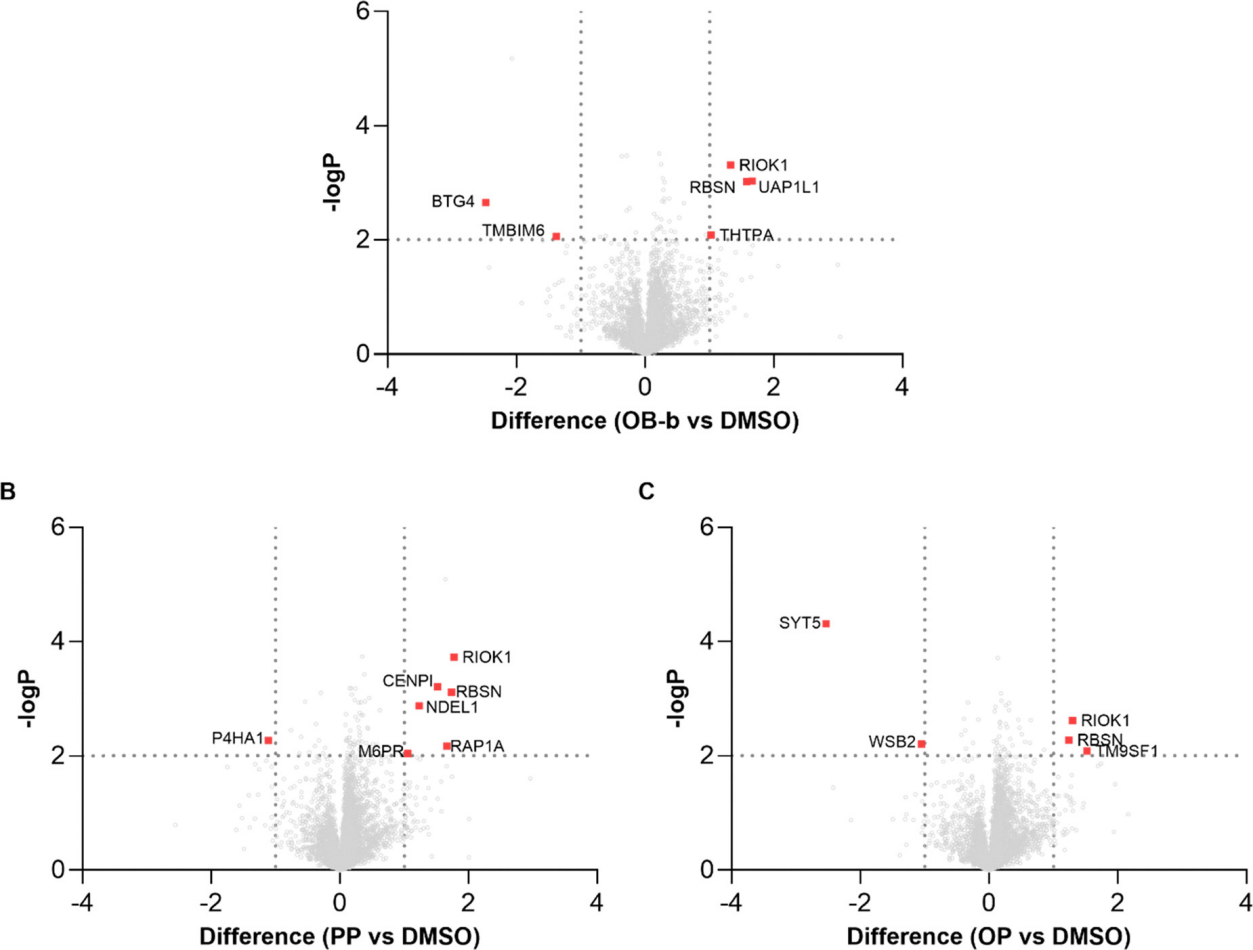
Whole proteome analysis of SJSA-1 cell line treated with (A) 1 µM OB-b, (B) 5 µM PP, or (C) 10 µM OP (right) *versus* vehicle (1% DMSO, left). Associated significance (*y*-axis) is determined by paired Student's *t*-test (FDR = 0.05, S0 = 0.1, *n* = 4). Total proteins quantified = 5303.

Overall, this first cross-scaffold comparison of α-helix mimetics revealed a clear difference in the selectivities of the three selected MDM2 inhibitors, with the oligobenzamides appearing least promiscuous. Moreover, while the three selected fully functionalised probes all enriched MDM2 relative to the null probe control, it was among the top 25% of enriched proteins only for the oligobenzamide probe OB-b3, reflective of its higher binding affinity and recombinant protein labelling. Additionally, for all three scaffolds, previously unknown targets have been identified which are more significantly labelled than MDM2 but which, nonetheless, represent compelling avenues for further exploration. Efforts thus far have firmly established the ability of α-helix mimetics to modulate a range of PPIs and achieve selectivity over anticipated off-targets. However, these results emphasise the need for further medicinal chemistry efforts to gain improved potency and selectivity. Given the advantages of modularity and adaptability that helix mimetics possess they continue to represent excellent tool compounds for the rapid identification of effective PPI inhibitors. This work also demonstrates that, without any medicinal chemistry optimisation, helix mimetics can engage their desired target in a competitive cellular environment and can enrich other targets with a high degree of significance Therefore, the development of α-helix mimetics is still of significant value to expanding the limits of the ligandable proteome.

## Author contributions

A. B, E. W. T and A. J. W conceived and supervised the project. A. D, A. W, H. K. C, T. F, J. W. H, J. L, A. M. T and P. Z performed the experiments. All authors contributed to data analysis. A. D and A. B wrote the manuscript, and all authors contributed to manuscript editing.

## Conflicts of interest

EWT is a founder and shareholder of Myricx Bio Ltd and Siftr Bio Ltd, an advisor to and holds share options in Samsara Therapeutics and Dunad Therapeutics, and receives current or recent funding from Myricx Bio Ltd, Pfizer Ltd, Kura Oncology, AstraZeneca, Merck & Co. and GSK. AW is a founder and shareholder of Siftr Bio Ltd.

## Supplementary Material

CB-OLF-D6CB00065G-s001

## Data Availability

Raw data associated with this study is currently available through the Imperial College Data Repository: https://doi.org/10.14469/hpc/15292. The mass spectrometry proteomics data have been deposited to the ProteomeXchange Consortium (https://www.proteomexchange.org) *via* the PRIDE partner repository. Raw data files can be found at https://doi.org/10.14469/hpc/15292 and the following DOIs: The mass spectrometry proteomics data have been deposited to the ProteomeXchange Consortium (https://www.proteomexchange.org) *via* the PRIDE partner repository with the dataset identifiers listed below:^52^ Supplementary information (SI): supplementary figures, experimental methods, probe NMR spectra, uncropped gel images and links to online data repositories. See DOI: https://doi.org/10.1039/d6cb00065g.
