# Supplementary material for: Proteome-wide target profiling of α-helix mimetics
Source: RSC Chem Biol. 2026 Jul 21;7(9):1818–27. doi: 10.1039/d6cb00065g (PMC13417162; doi:10.1039/d6cb00065g)

Supplementary Information (SI) for RSC Chemical Biology.
This journal is © The Royal Society of Chemistry 2026

Proteome-wide Target Profiling of α-helix Mimetics

Amrita Date,[1] Archie Wall,[1] Hannah Kiely-Collins,[1] Theo Flack,[1] Jack W. Houghton,[1] Jianan Lu,[1] Adam M. Thomas,[1] Peiyu Zhang,[2,3] Andrew J. Wilson,[2,3,4] Edward W. Tate,[1,5] Anna Barnard[1]*

1. Department of Chemistry, Molecular Sciences Research Hub, Imperial College London, 82 Wood Lane, London, W12 0BZ, United Kingdom
2. Astbury Centre for Structural Molecular Biology, University of Leeds, Woodhouse Lane, Leeds LS2 9JT, United Kingdom
3. School of Chemistry, University of Leeds, Woodhouse Lane, Leeds, LS2 9JT, United Kingdom
4. School of Chemistry, University of Birmingham, Edgbaston, Birmingham B15 2TT, United Kingdom
5. The Francis Crick Institute, London, NW1 1AT, United Kingdom

* Correspondence to: Dr Anna Barnard, Molecular Sciences Research Hub, Imperial College London, 82 Wood Lane, London W12 0BZ, United Kingdom. Email: a.barnard@imperial.ac.uk

Contents

Supplementary Figures 2
Experimental 14
Online data repository 52
References 53
NMR spectra of final products 55
Analytical HPLC traces for final products 85
Uncropped blots 101

Supplementary Figures

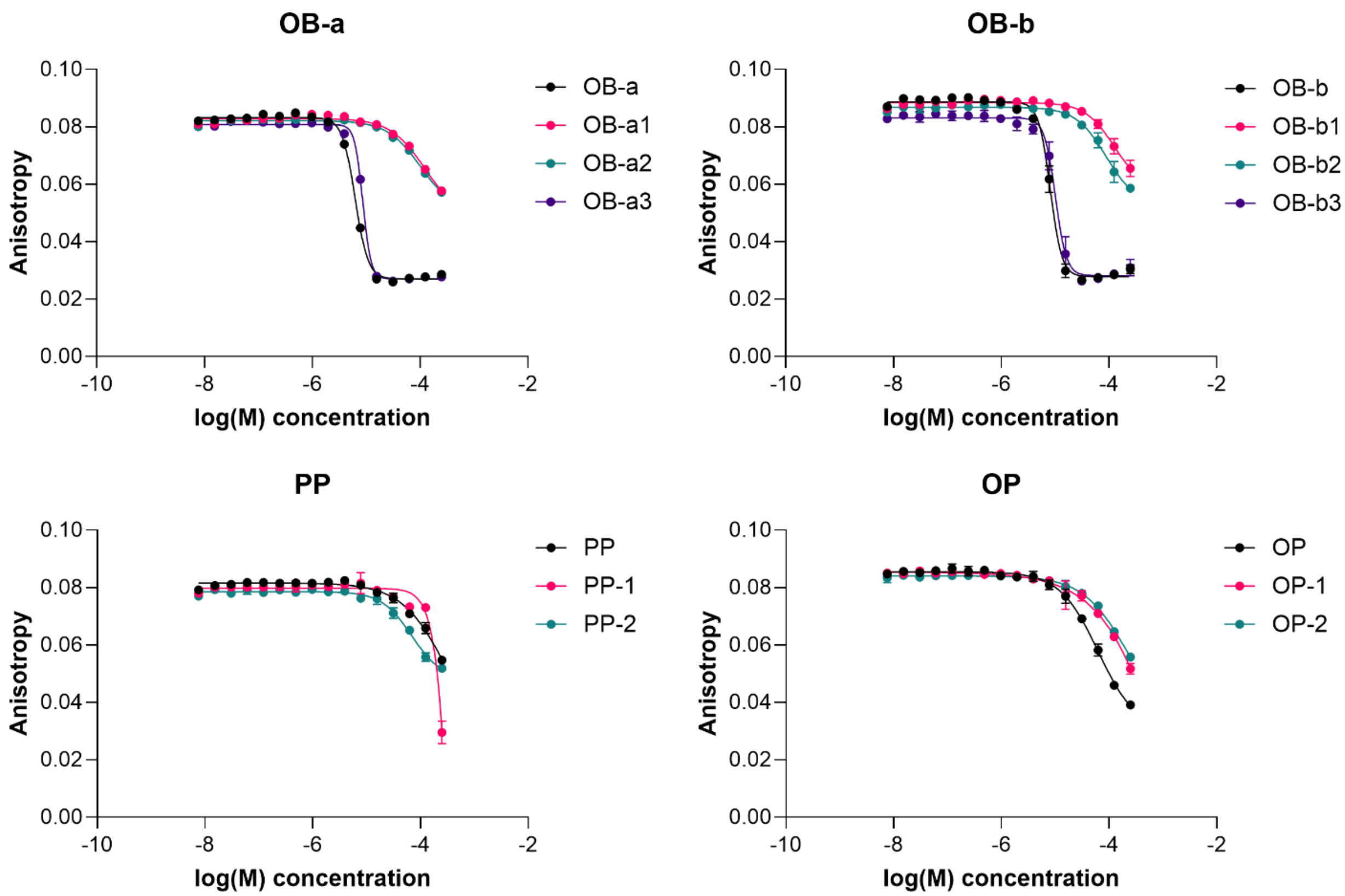

	IC$_{50}$ (μM)			
	**OB-a**	**OB-b**	**PP**	**OP**
**Parent**	6.3 ± 0.2	8.2 ± 0.1	282.6 ± 5.2	75.3 ± 1.0
**Probe 1**	141.0 ± 12.6	147.2 ± 9.9	168.6 ± 2.4	212.5 ± 4.1
**Probe 2**	101.5 ± 4.4	101.9 ± 5.2	210.5 ± 1.1	268.8 ± 2.7
**Probe 3**	8.6 ± 0.2	10.2 ± 0.2		

Figure S1: *Fluorescence anisotropy competition assay conducted by titrating analyte against MDM2 (150 nM) and Flu-p53 (25 nM). Corresponding IC$_{50}$ values reported from n = 3. Probe binding is denoted as good (<2-fold increase in IC$_{50}$), slight decrease (2 to 10-fold increase in IC$_{50}$), poor (>10-fold increase in IC$_{50}$)*

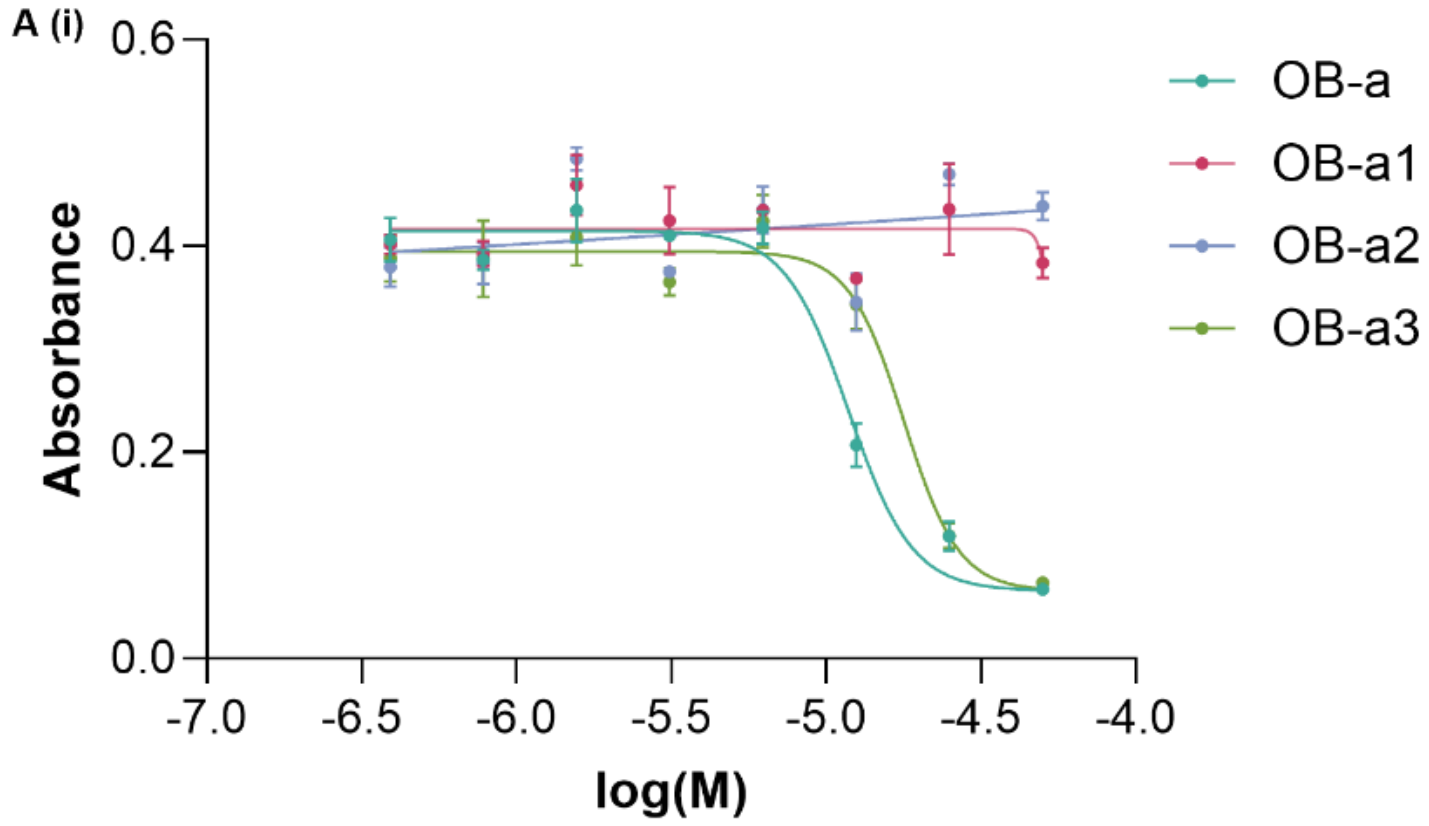

	EC_{50} (μM)
**OB-a**	11.8 ± 0.3
**OB-a1**	53.2 ± 25.5
**OB-a2**	Unstable
**OB-a3**	17.5 ± 0.4

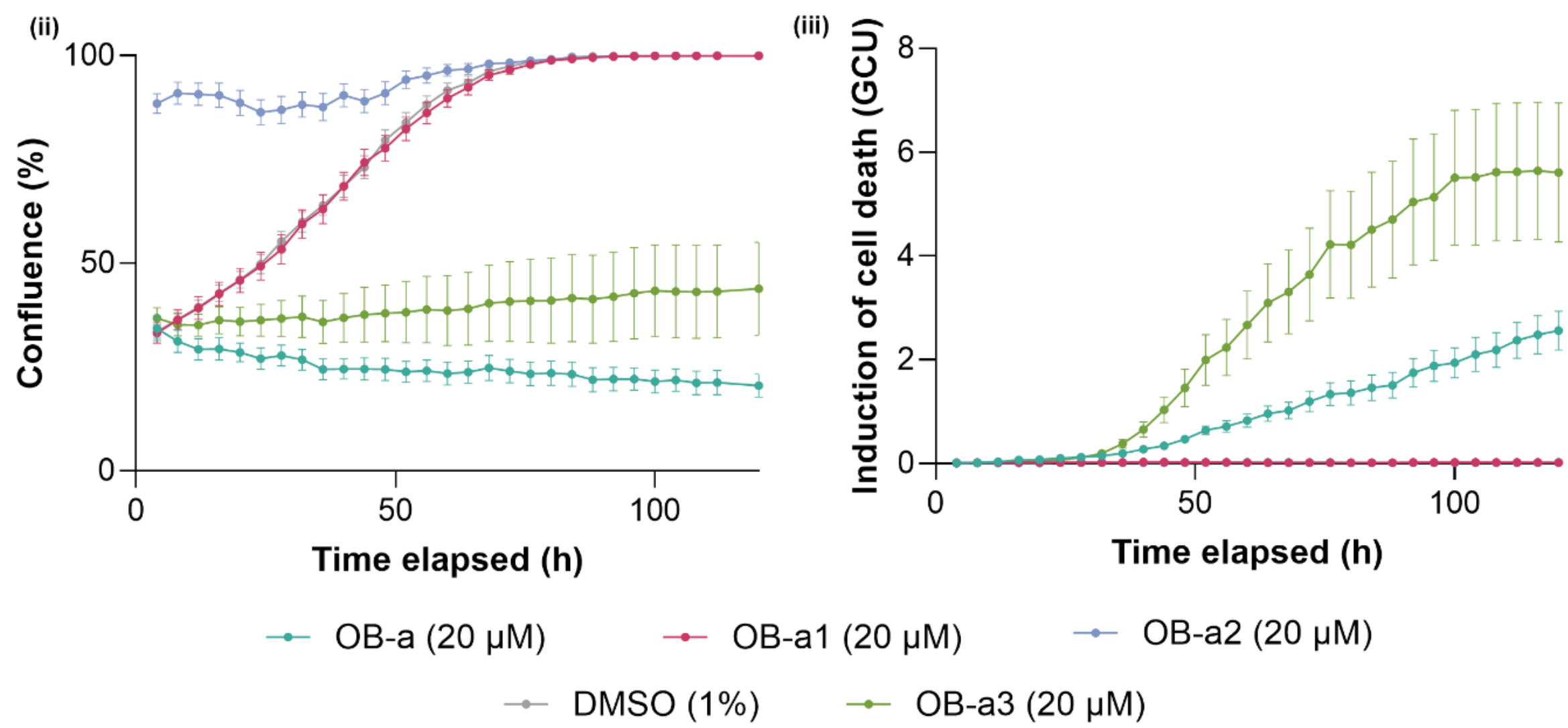

B (i)

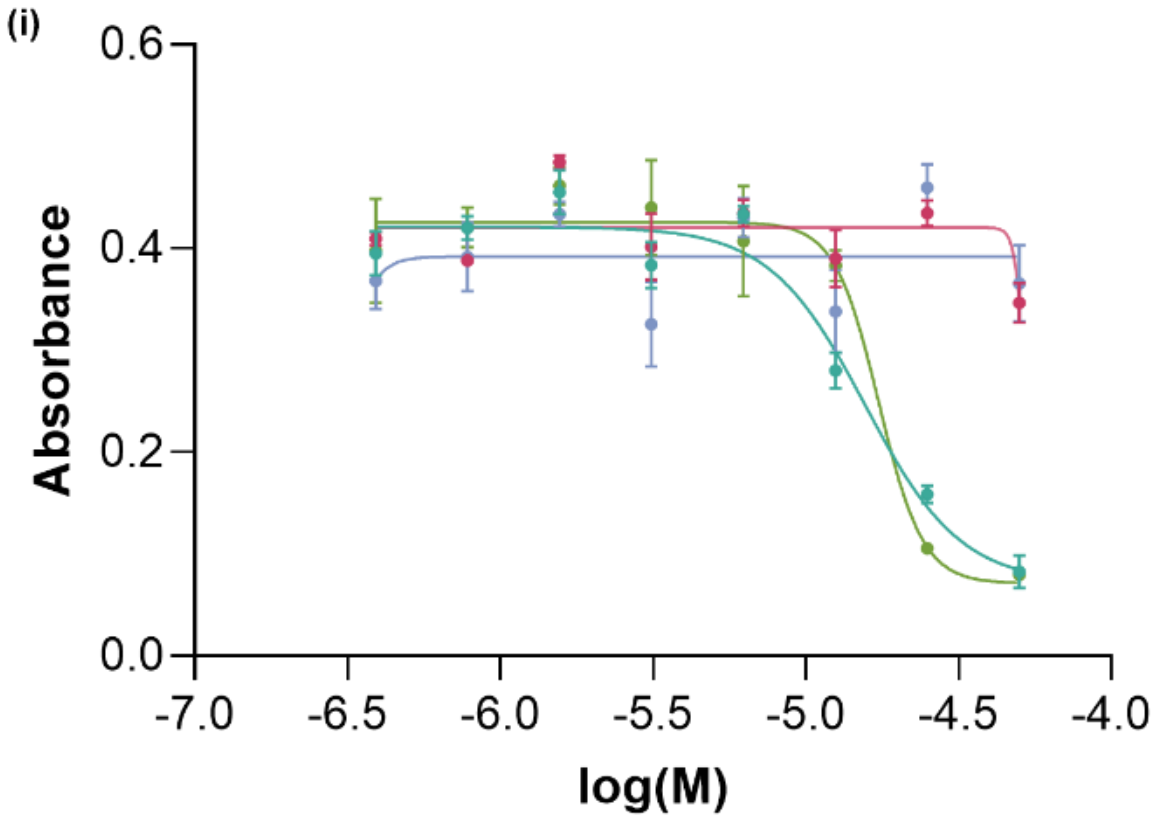

	EC_{50} (μM)
**OB-b**	15.3 ± 0.9
**OB-b1**	53.2 ± 25.5
**OB-b2**	Unstable
**OB-b3**	17.0 ± 0.5

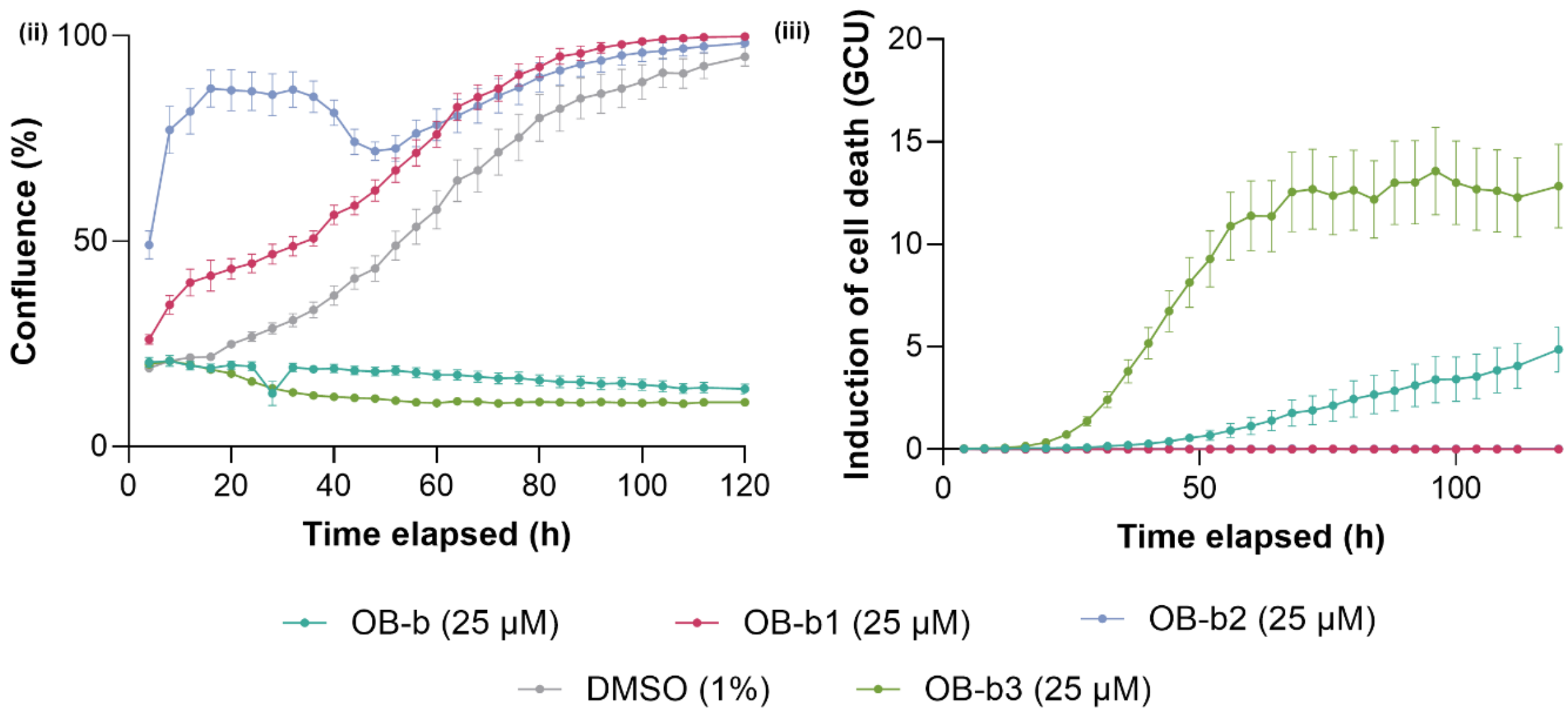

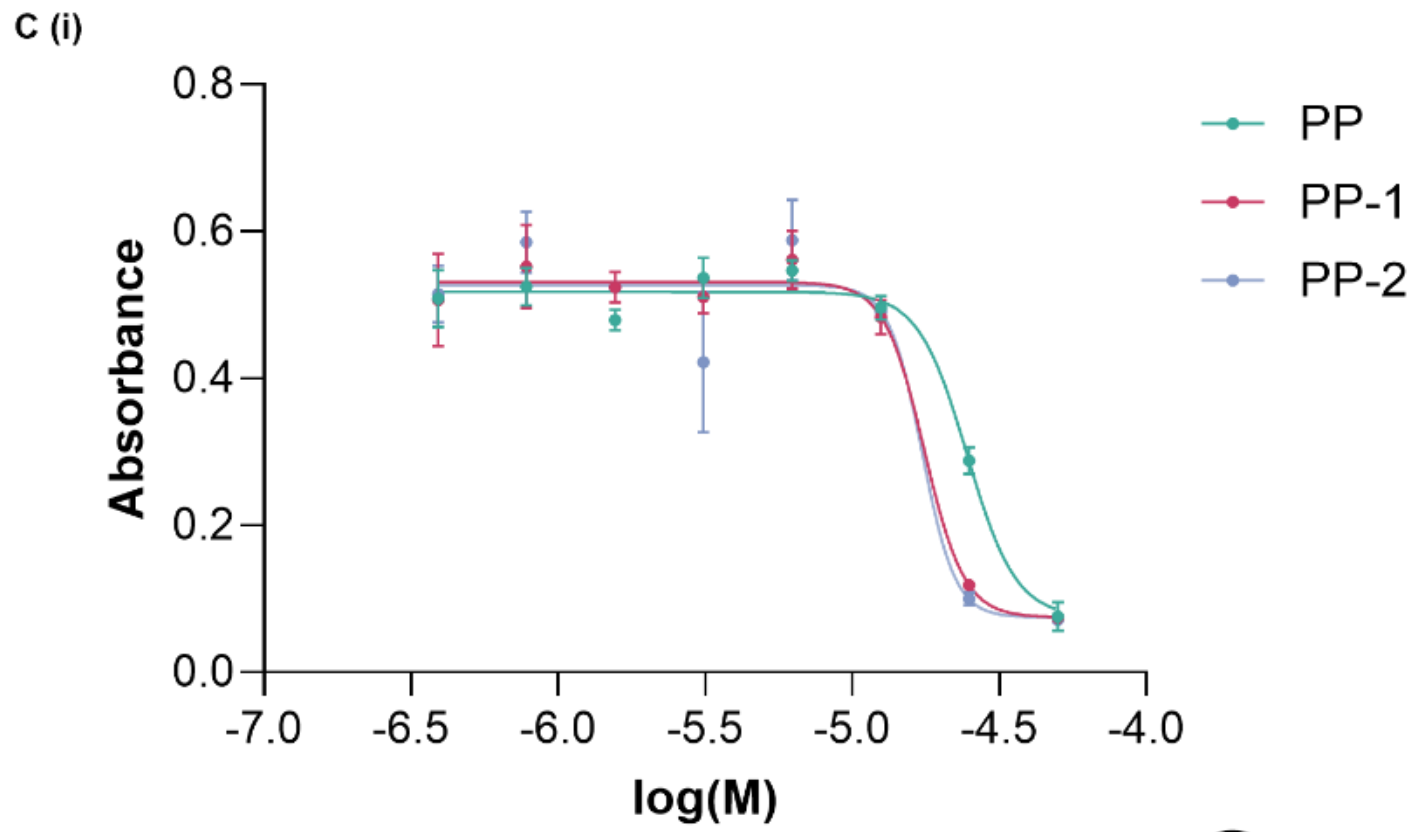

	EC_{50} (μM)
**PP**	25.6 ± 1.1
**PP-1**	17.6 ± 0.3
**PP-2**	17.4 ± 0.5

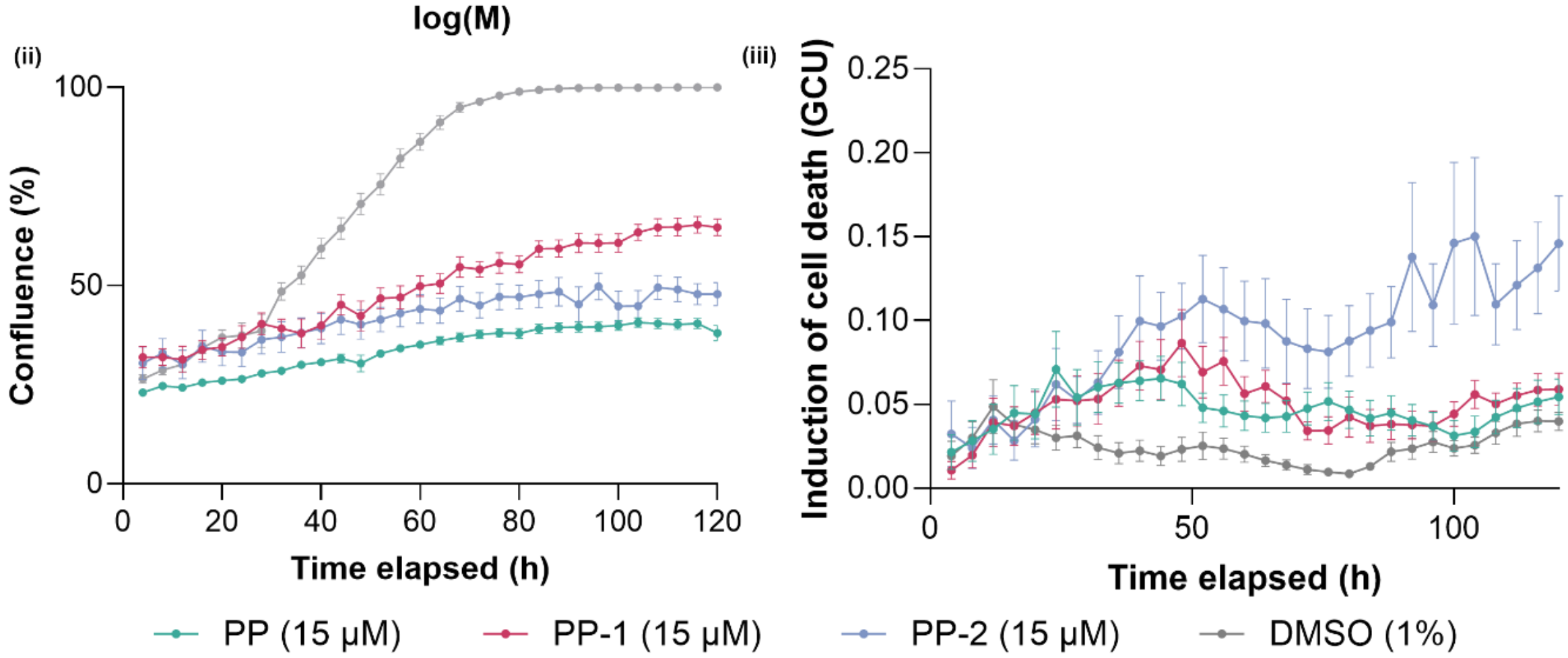

D (i)

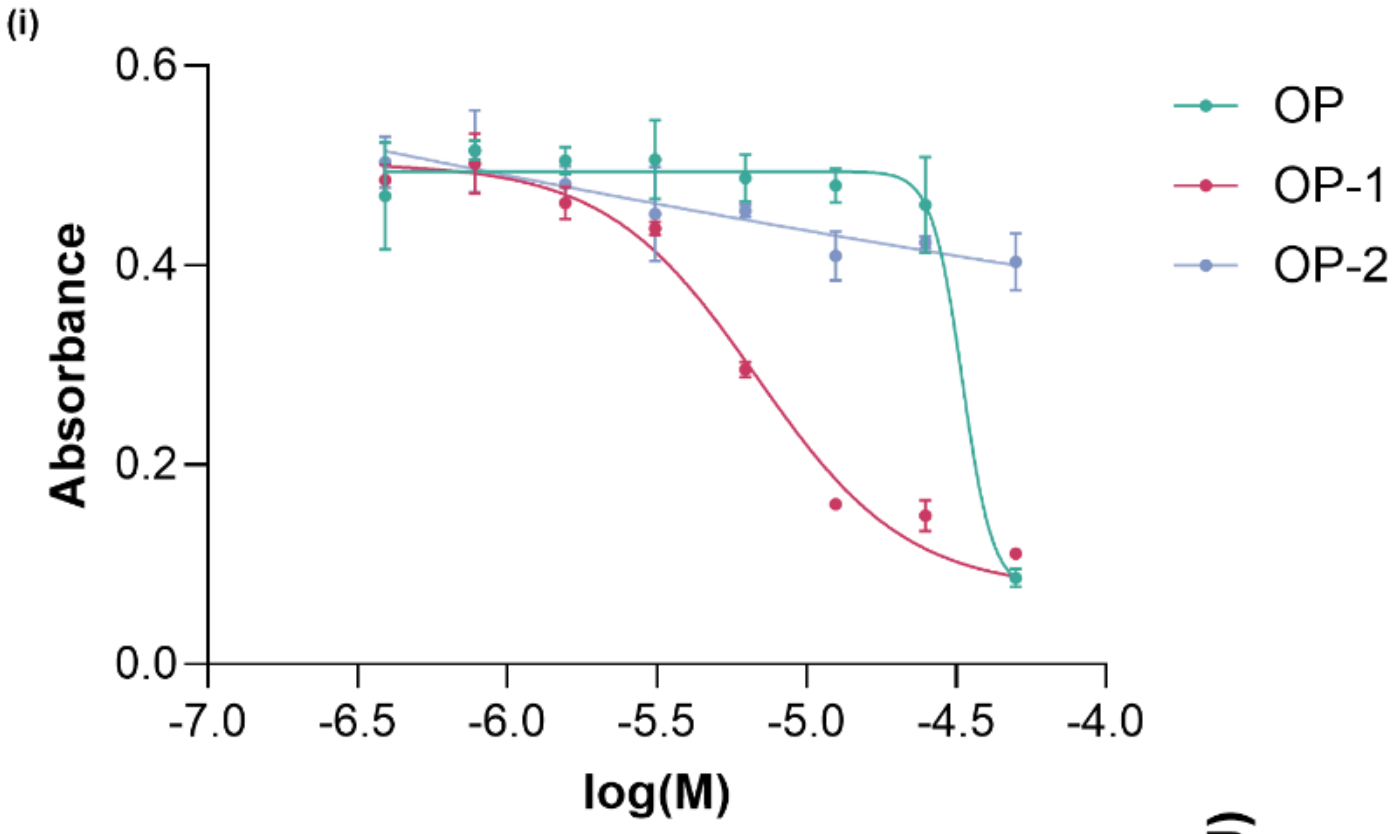

	EC_{50} (μM)
**OP**	104.6 ± 37.0
**OP-1**	6.0 ± 0.1
**OP-2**	Unstable

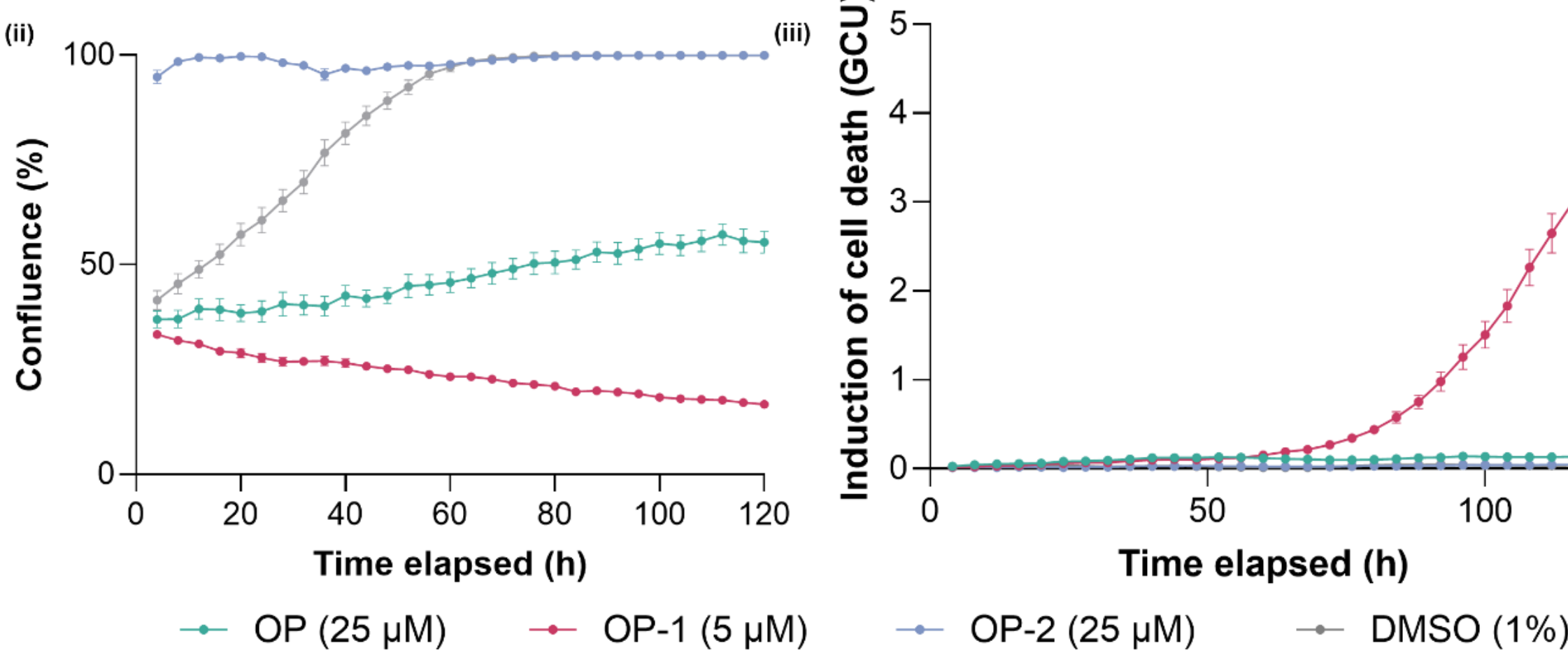

Figure S2: *Effect of* ***(A)*** *napthyl-substituted oligobenzamide,* ***(B)*** *4-chlorophenyl-substituted oligobenzamide,* ***(C)*** *pyrrolopyrimidine), and* ***(D)*** *oxopiperazine probes on cell proliferation and cytotoxicity.* ***(i)*** *MTS assay conducted 3 days after treatment of SJSA-1 cells with OP and its probes, and the EC_{50} values determined (mean ± SEM, n = 3). Real-time live cell imaging data, showing the effect of treatment with OP and its probes (20 μM) on confluence* ***(ii)*** *and cytotoxicity (plotted as a ratio of the total intensity of the fluorescent response from SYTOX green per well to the area covered by cells,* ***iii)*** *in SJSA-1 cells, recorded over five days using an IncuCyte S3 cell imager (mean ± SEM, n = 3).*

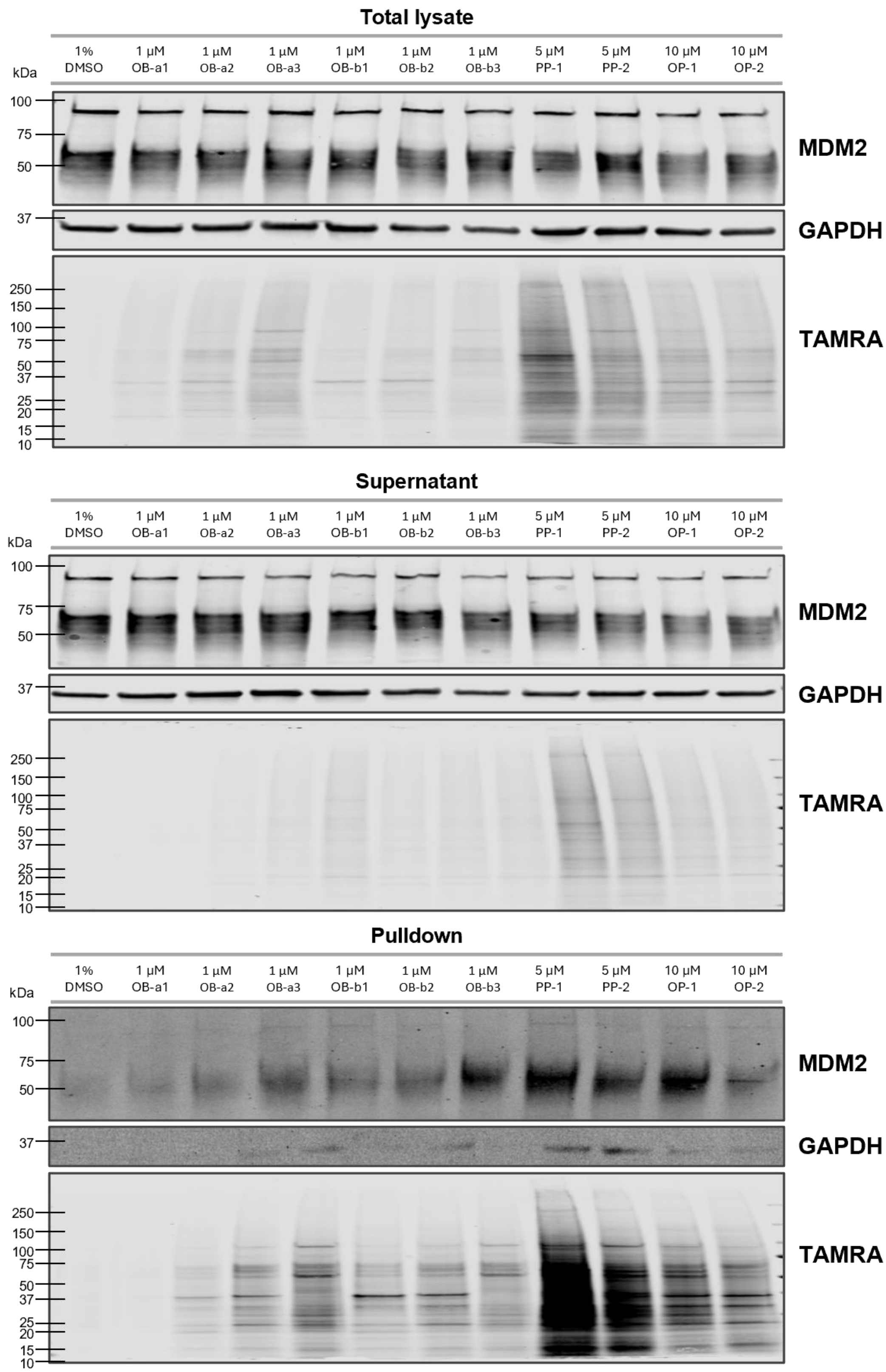

Figure S3: *Western blot showing the labelling and pulldown of MDM2 using helix mimetic probes in SJSA-1 cells. Overall protein labelling was detected as in-gel fluorescence for TAMRA.*

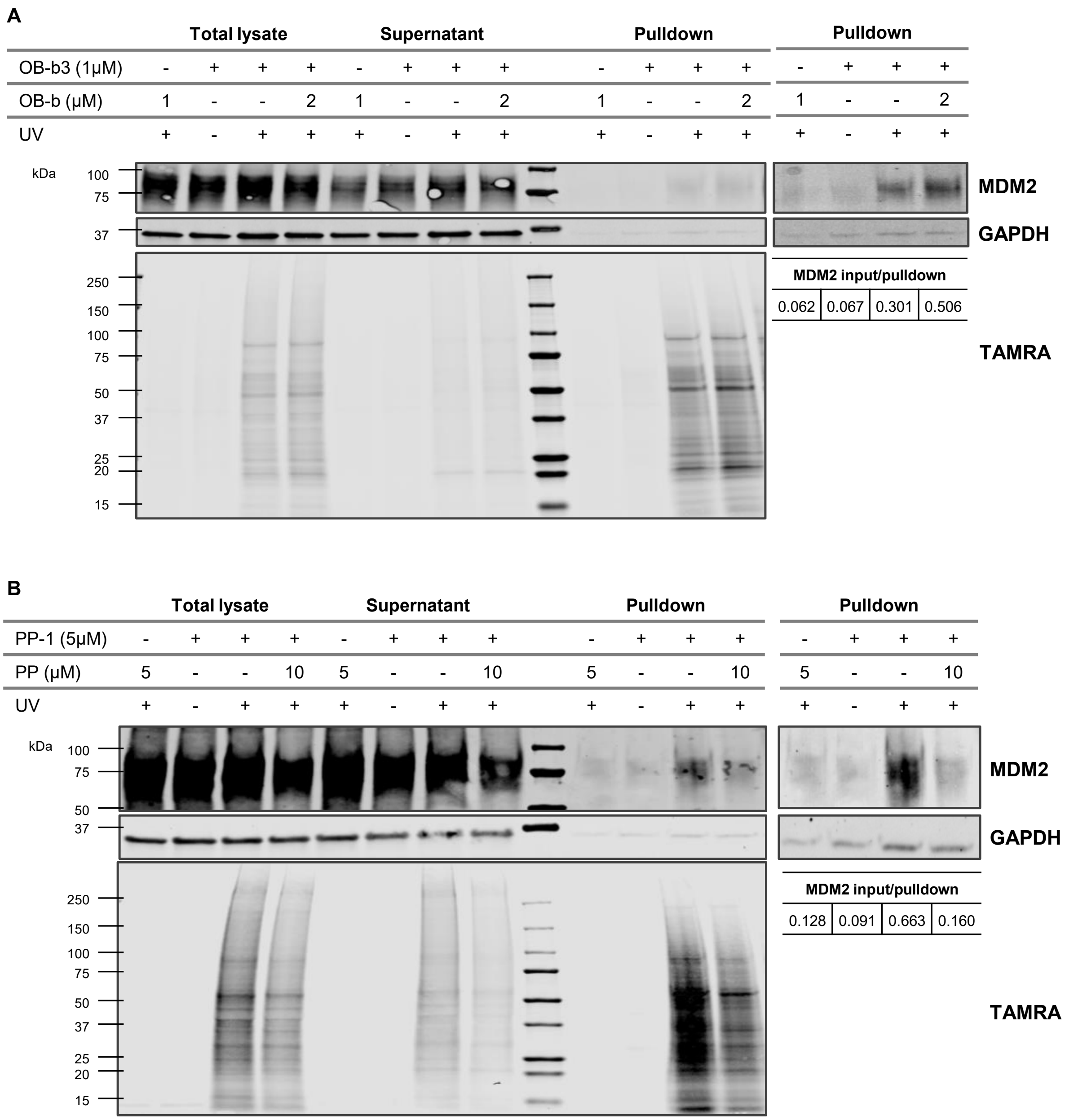
A
Total lysate
Supernatant
Pulldown
Pulldown
OB-b3 (1μM)
OB-b (μM)
UV
kDa
100
75
37
250
150
100
75
50
37
25
20
15
MDM2
GAPDH
MDM2 input/pulldown
0.062 | 0.067 | 0.301 | 0.506
TAMRA
B
Total lysate
Supernatant
Pulldown
Pulldown
PP-1 (5μM)
PP (μM)
UV
kDa
100
75
50
37
250
150
100
75
50
37
25
20
15
MDM2
GAPDH
MDM2 input/pulldown
0.128 | 0.091 | 0.663 | 0.160
TAMRA

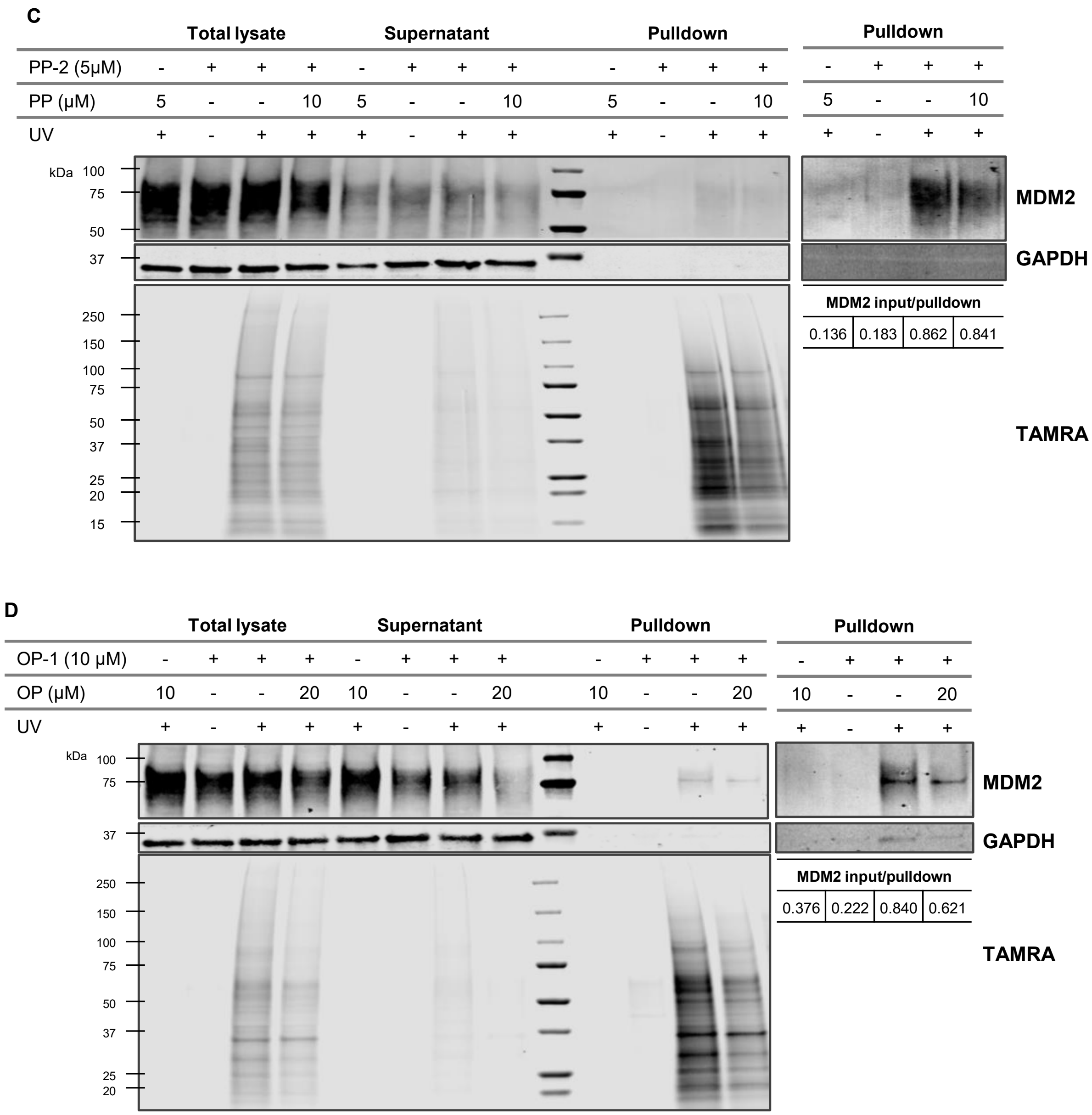

Figure S4: *Western blot showing the light-dependent labelling and pulldown of MDM2 using OB-b3* ***(A)****, PP-1* ***(B)****, PP-2* ***(C)****, and OP-1* ***(D)*** *in SJSA-1 cells. Blots for MDM2 and GAPDH for total lysate, supernatant, and pulldown fractions have been imaged together. Blots for the pulldown fraction are also shown imaged separately at higher exposure to achieve improved dynamic range. Overall protein labelling was detected as in-gel fluorescence for TAMRA. Blots quantified using ImageJ.*

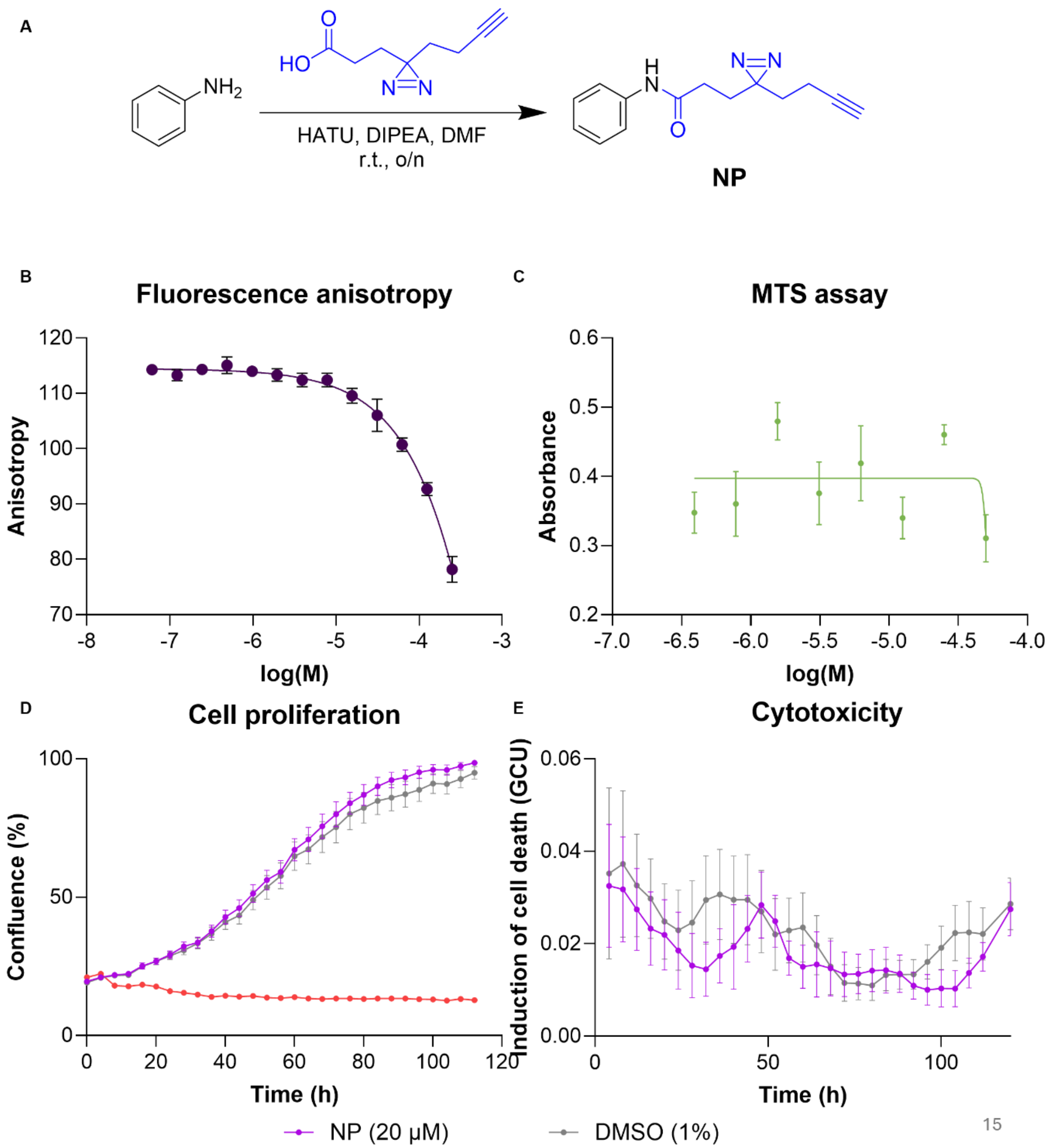
A
NH2
HO
O
N=N
HATU, DIPEA, DMF
r.t., o/n
H
N
O
N=N
NP
B
Fluorescence anisotropy
Anisotropy
log(M)
C
MTS assay
Absorbance
log(M)
D
Cell proliferation
Confluence (%)
Time (h)
E
Cytotoxicity
Induction of cell death (GCU)
Time (h)
NP (20 µM)
DMSO (1%)

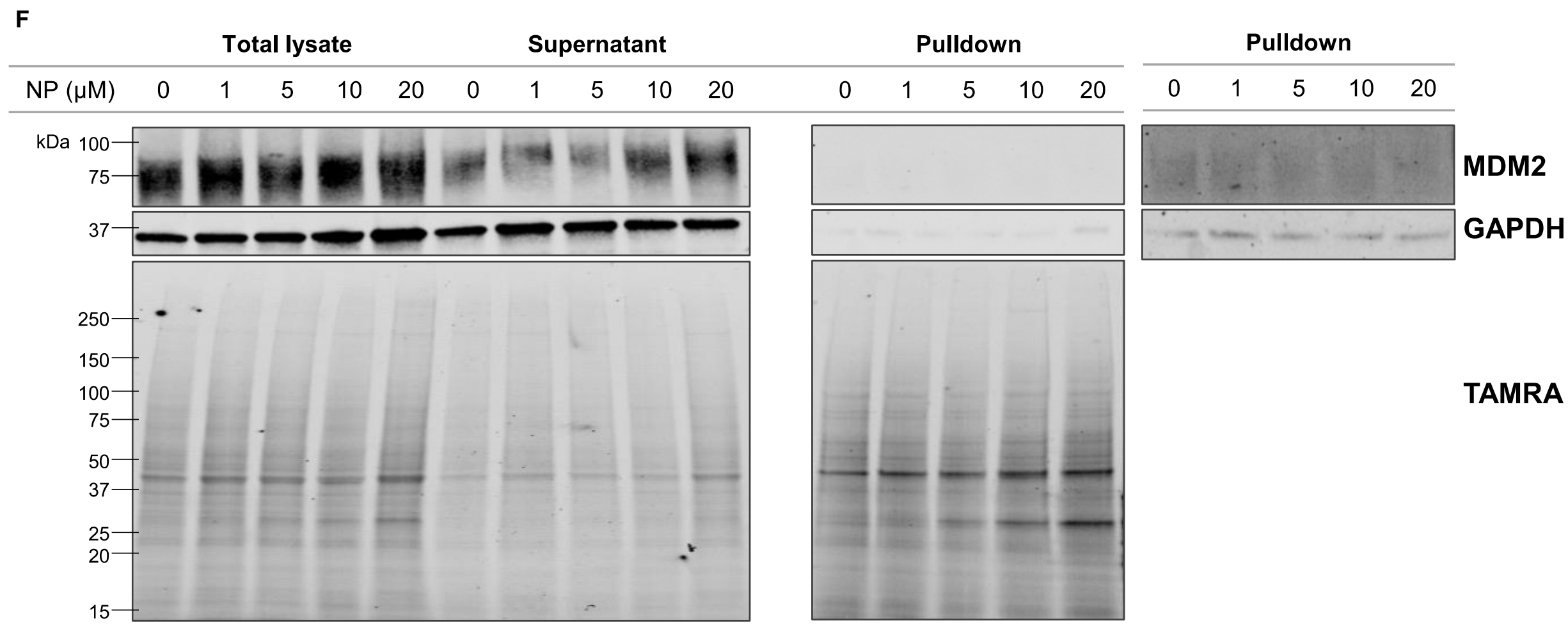

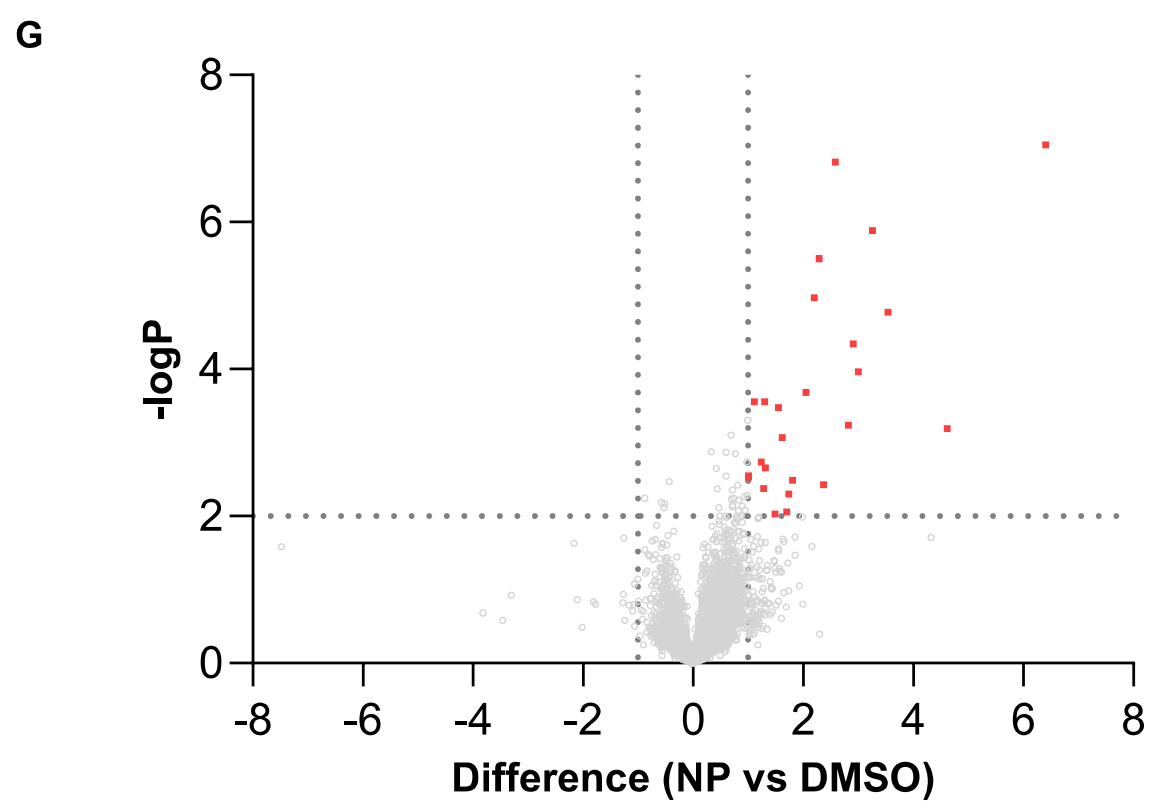

NP targets

MAOA	CTSD	HMOX2	TBL1XR1	ECE1
LPCAT3	IPO9	SCARB2	CRABP2	ECH1
PCYOX1	NRDC	PSMB4	CSNK2B	HSDL1
PRCP	CCDC47	SMARCC2	B4CALNT1	
GLB1	HLA-A	THOP1	ZMPSTE24	

Figure S5: ***(A)*** *Synthesis of NP from aniline.* ***(B)*** *Fluorescence anisotropy competition assay conducted applying assay conditions reported by Wilson and coworkers[9] (n = 3). Analytes titrated against MDM2 (150 nM) and Flu-p53 (25 nM).* ***(C)*** *MTS assay conducted 3 days after treatment of SJSA-1 cells with NP (n = 3). Real-time live cell imaging data, showing the effect NP (20 µM) treatment on confluence* ***(D)*** *and cytotoxicity (plotted as a ratio of the total intensity of the fluorescent response from SYTOX green per well to the area covered by cells;* ***E****) in SJSA-1 cells, recorded over five days using an IncuCyte S3 cell imager (mean ± SEM, n = 3).* ***(F)*** *Western blot showing a lack of labelling and pulldown of MDM2 using NP in SJSA-1 cells. Blots for MDM2 and GAPDH for total lysate, supernatant, and pulldown fractions have been imaged together. Blots for the pulldown fraction are also shown imaged separately at higher exposure to achieve improved dynamic range. Overall protein labelling was detected as in-gel fluorescence for TAMRA.* ***(G)*** *Target engagement profile of NP in SJSA-1 cells. Volcano plots showing differences in enrichment (x-axis) between live cells treated with 20 µM NP (right) versus 1% DMSO as a negative control (left). Associated significance (y-axis) is determined by paired Student's t-test (FDR = 0.05, S0 = 0.1, n = 4). Statistically significant hits identified are highlighted in red. Total proteins quantified = 4252. List of identified NP targets, with those that have previously been reported as off-targets of diazirine probes marked in green.*

A

(i)

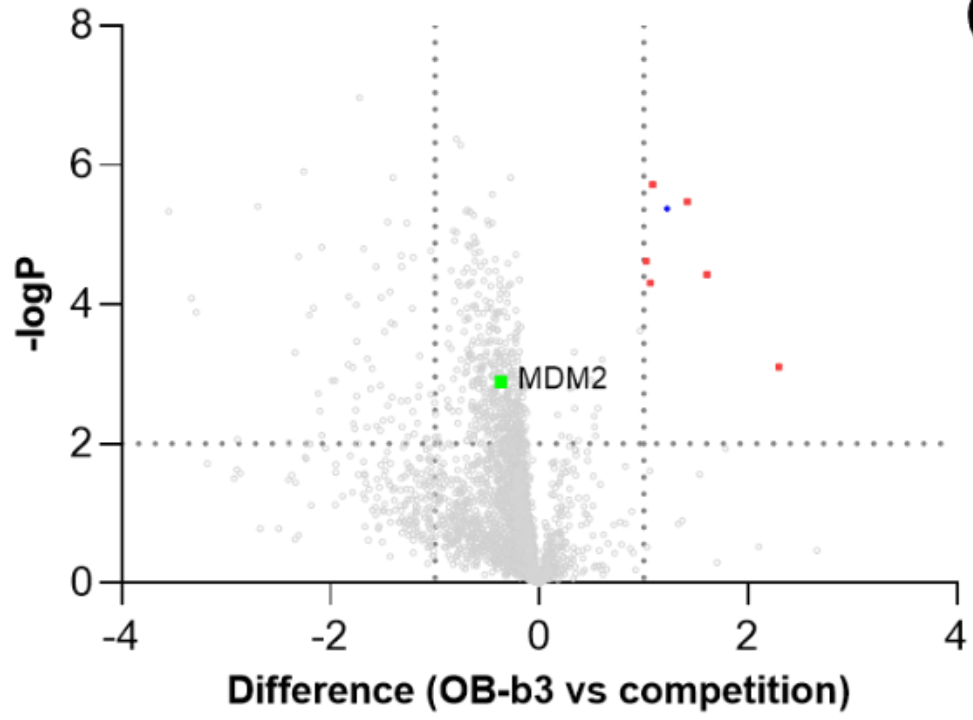

(ii)

OB-b3 Hits		
DPP8	LTA4H	SPR
CYP27A1	HSD17B4	MTAP

(iii)

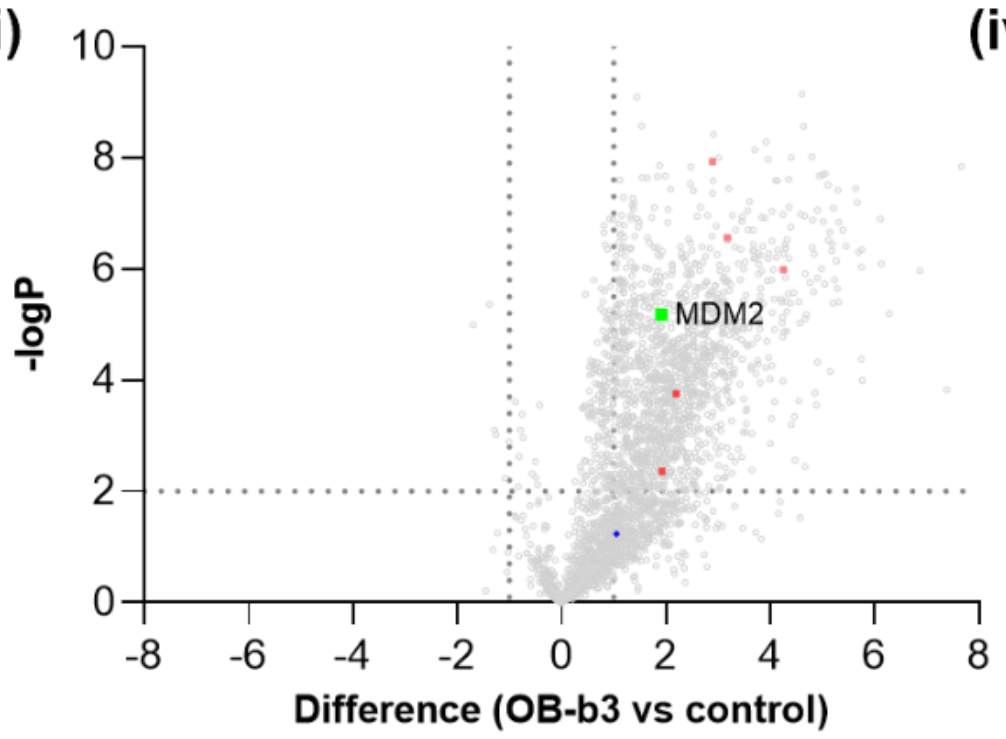

(iv)

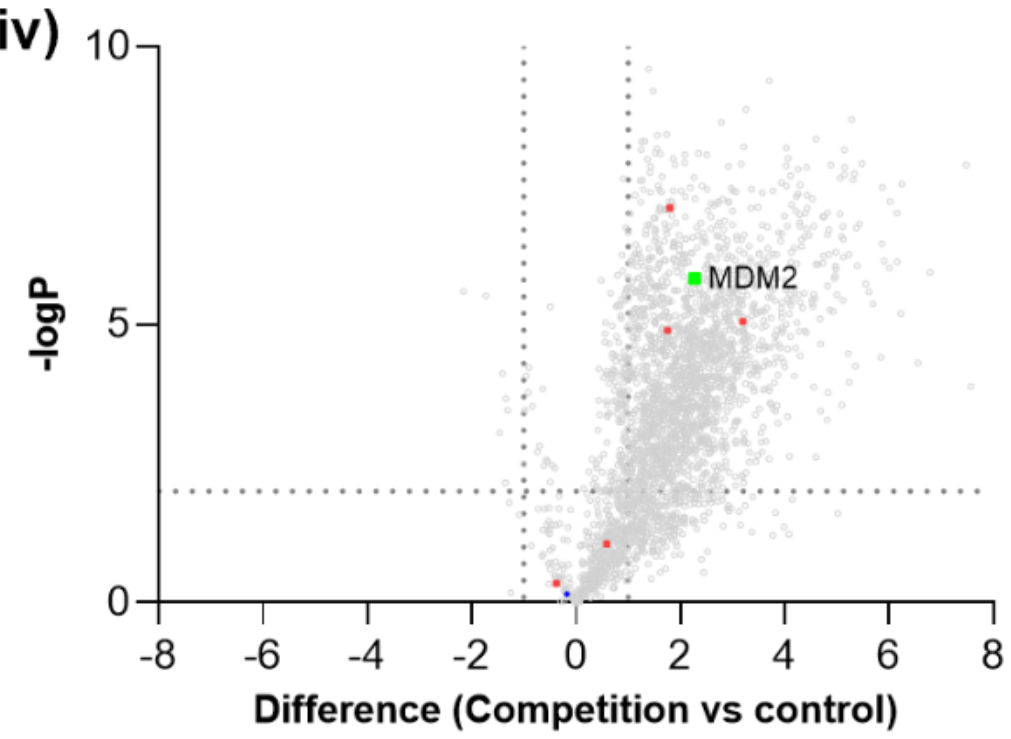

B

(i)

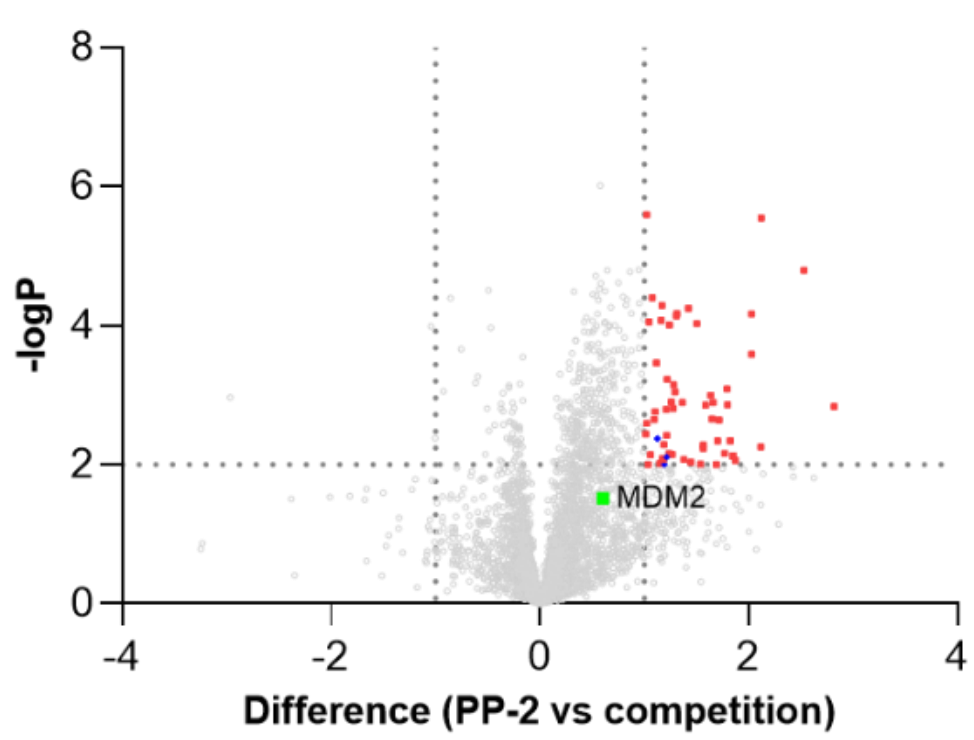

(ii)

PP-2 Hits					
ARL1	DHCR24	MBTPS2	PLD3	SMARCD2	TXNIP
ARMCX2	DMAC1	MICOS13	PPT1	SMDT1	UCHL5
ARSB	FAM168B	MPDU1	PRR11	SMPD1	VAMP7
B4GAT1	FASTKD2	NDUFA3	RBP1	SPRYD7	VEZT
C1QL1	GMPPB	NDUFA8	RHBDD3	SQLE	VMA21
CHCHD2P9	IFITM1	NOSIP	SARAF	SSR3	XXYLT1
COX17	IL1RAP	NUCB1	SCPEP1	TMEM258	
CRLS1	LIPA	ORMDL3	SGCB	TMEM41B	
CTSD	MAVS	PEX13	SLC35C1	TMUB2	
CYB5B	MBOAT2	PLA2G15	SLK	TOR1AIP2	

(iii)

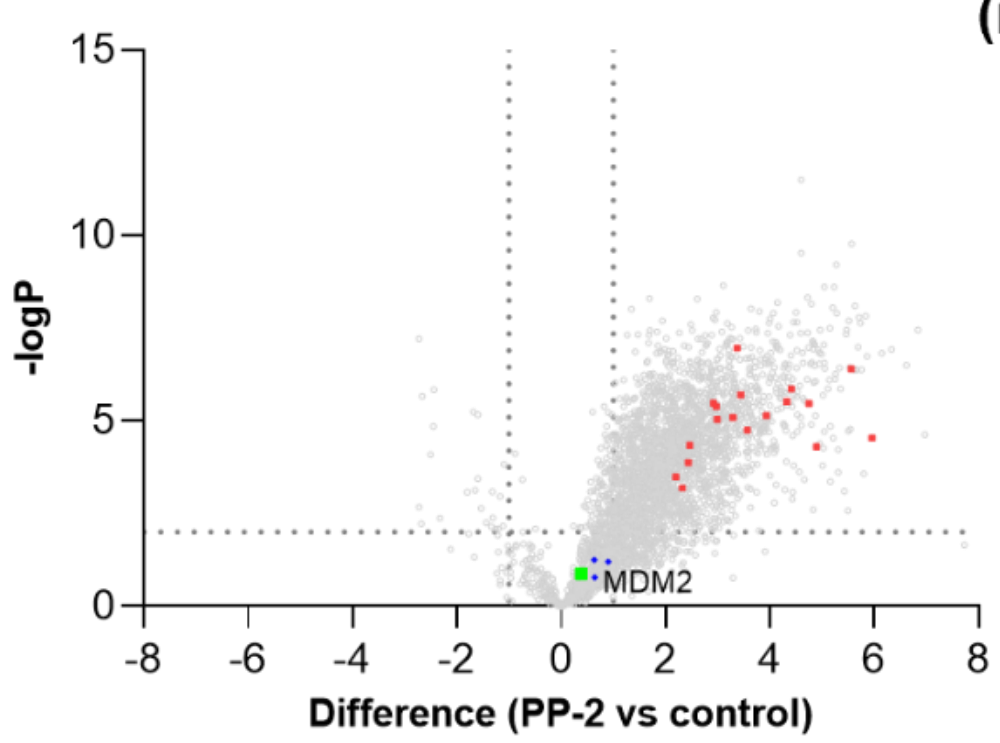

(iv)

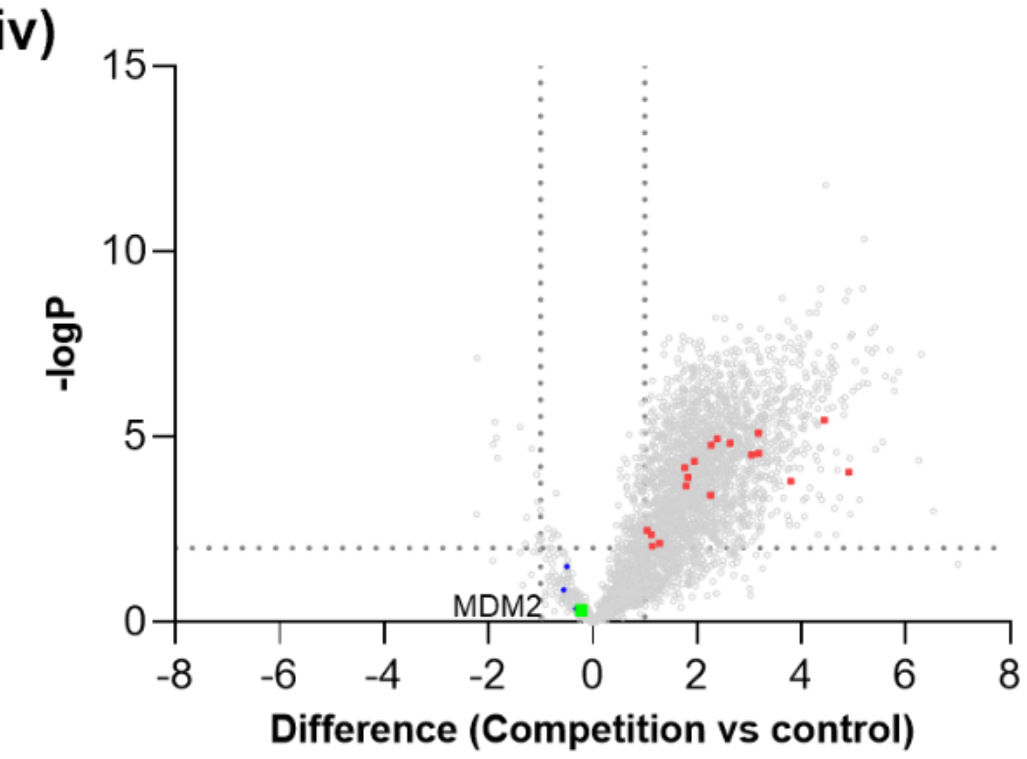

C

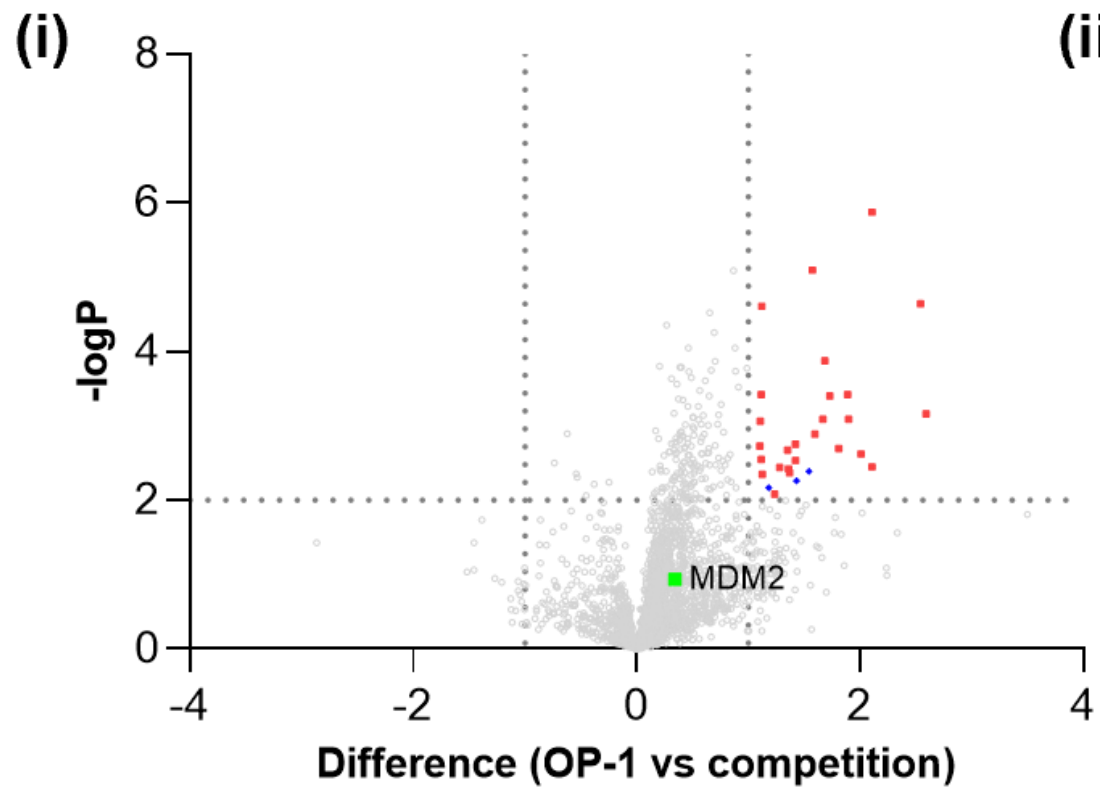

(ii)

OP-1 Hits				
ALG11	FAM20B	NDUFB10	PRCP	TMEM160
CELF1	ITPR3	NUCB1	RTN4	YIF1B
CHCHD2P9	LAPTM4A	PARL	SLC35C1	
CYP2U1	M6PR	PEX14	SQSTM1	
EMC10	MICOS13	PLD3	TMEM123	
FAM177A1	MTX2	POMK	TMEM14C	

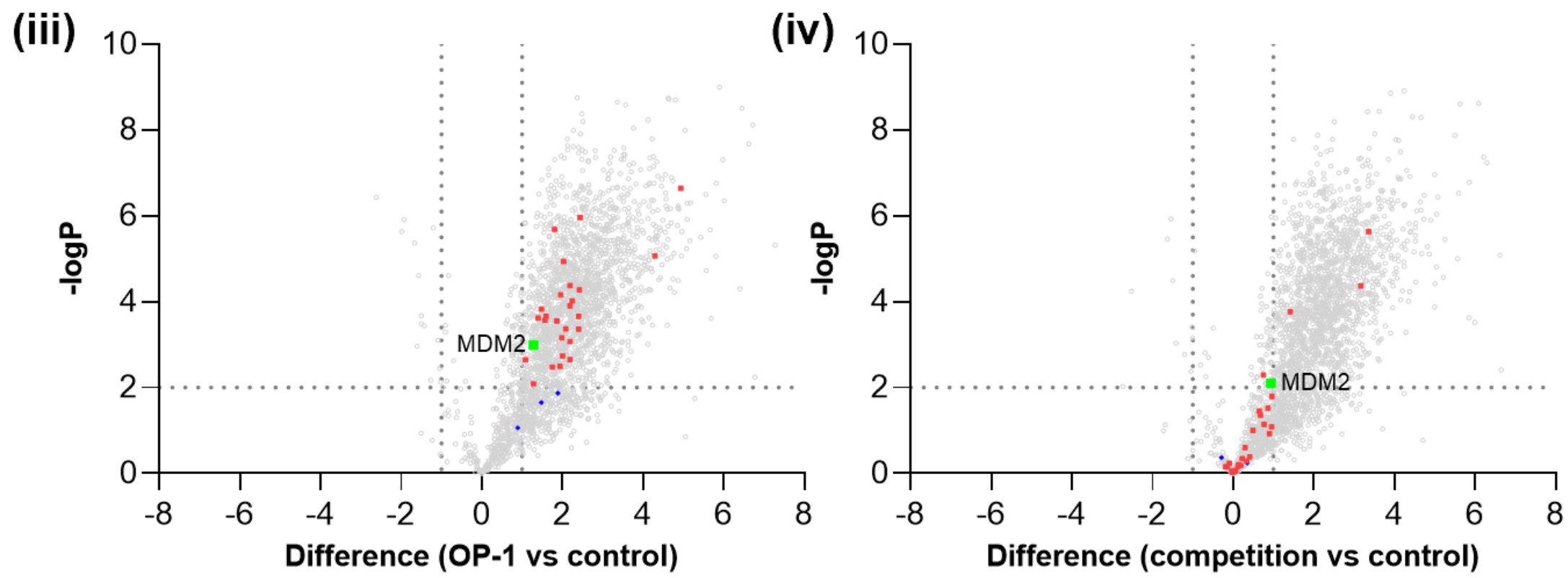

Figure S6: (A) *Target engagement profile of OB-b3 in SJSA-1 cells. Volcano plots showing differences in enrichment (x-axis) between live cells treated with 1 µM OB-b3 ± 2 µM OB-b (right) versus 1 µM OB-b as a negative control (left) or 1 µM OB-b3 (right) versus 1 µM OB-b3 + 2 µM OB-b (competition, left). Total proteins quantified = 2663.* ***(B)*** *Target engagement profile of PP-2 in SJSA-1 cells. Volcano plots showing differences in enrichment (x-axis) between live cells treated with 5 µM PP-2 ± 10 µM PP (right) versus 5 µM PP as a negative control (left) or 5 µM PP-2 (right) versus 5 µM PP-2 + 10 µM PP (competition, left).* ***(C)*** *Target engagement profile of OP-1 in SJSA-1 cells. Volcano plots showing differences in enrichment (x-axis) between live cells treated with 10 µM OP-1 ± 20 µM OP (right) versus 10 µM OP as a negative control (left) or 10 µM OP-1 (right) versus 10 µM OP-1 + 20 µM OP (competition, left). Associated significance (y-axis) is determined by paired Student's t-test (FDR = 0.05, S0 = 0.1, n = 4). Statistically significant hits identified are highlighted in red and hits that appear significant in the top volcano plot but have been disregarded due to insignificant labelling are shown in blue. Statistically significant hits are listed in the table; those not enriched relative to a null probe control shown in grey.*

Experimental

Synthesis

Reagents and solvents used in the synthesis and purification of the molecules were purchased as high-grade commercial products from Sigma-Aldrich, Fluorochem, VWR International or Alfa Aesar. Navtemadlin was purchased from MedChem Express. Azide-TAMTA-biotin (AzTB) was synthesised in-house as previously described.[1] *N*-(2-(3-methyl-3*H*-diazirin-3-yl)ethyl)prop-2-yn-1-aminium chloride was synthesised in-house as previously described.[2]

Synthesis of α-helix mimetics was conducted on Rink-amide or pre-loaded Wang resin. Room temperature (rt) reactions, reaction draining, and resin washes were conducted in polypropylene syringes fitted with polyethylene frits (20 μm porosity). Prior to commencing synthesis, the resin was swollen in DMF for 30 min, and deprotected by agitation with piperidine (20% in DMF) for 2 x 20 min at rt. After this, resin was washed with DMF (8 x 20 mL) and taken forward for synthesis. Where changes in solvent system were required between steps, the resin was washed with DCM (2 x 20 mL) and diethyl ether (2 x 20 mL) and air dried, before swelling in the solvent for the next reaction. At every step, a test cleavage was conducted by treating an aliquot of resin-bound compound with 95% TFA in DCM for 1 h at rt. The cleavage solution was collected, diluted with DCM, dried under a stream of air, and analysed by LC-MS to ensure that the reaction had proceeded successfully. Synthesis of parent molecules was conducted as previously reported.[3, 4] Microwave assisted reactions were carried out in sealed vials using a Biotage Initiator microwave reactor, with pressure limits set at 10 mbar.

Reactions were monitored by liquid chromatography-mass spectrometry (LC-MS), using one of the following systems:

- Waters micromass ZQ spectrometer, equipped with an XBridge peptide BEH C18 5 μm 4.6 x 100 mm column for peptides. A gradient of 50–98% acetonitrile (MeCN) in water, both containing 0.1% formic acid (FA), was run over 15 min at a flow rate of 1.2 mL/min.
- Agilent system A: Agilent 1260 Infinity II system fitted with an Agilent Poroshell HPH C18 2.7 μm, 3.0 x 50 mm column and MSD XT mass detector. A gradient of 4.5–90% MeCN and water, both containing either 0.1% formic acid (acidic method) or 0.3% ammonium hydroxide (basic method) was run over 7 min at a flow rate of 0.8 mL/min. Unless stated otherwise, the acidic method was used for analysis.
- Agilent system B: Agilent system b: Agilent 1260 Infinity II system fitted with a Raptor C18 2.7 μm, 2.1 x 50 mm column maintained at 40°C and an Agilent G6125B MSD mass detector. A gradient of MeCN and water, both containing 0.1% FA was run over 1 or 2 min, as specified.
- Agilent system C: Agilent 1260 Infinity II fitted with a Poroshell 120 EC-C18, 2.1 x 50 mm, 1.9 μm column, held at 40°C and an LC single-quad InfinityLab LC/MSD for mass detection. A gradient of 5–95% MeCN and water, both containing 0.1% FA was run over 1.5 min at a flow rate of 0.5 mL/min.

Purification of the final molecules was carried out by high performance liquid chromatography (HPLC), using one of the flowing systems:

- Shimadzu LC-20AR preparative HPLC system equipped with a Phenomenex Aeris Peptide 5 μm XB-C18 column (150 x 21 mm, 100 Å). A gradient of MeCN and water, both containing 0.1% trifluoroacetic acid (TFA) was applied at a flow rate of 20 mL/min. Detection of absorption at 200 and 280 nm was used to isolate the desired product.
- Agilent 1290 Infinity II preparative HPLC system equipped with a Poroshell 120, 4 μm SB-C18 (150 x 21.2 mm). Gradients of MeCN and water were run at a flow rate of 20 mL/min. Chromatograms were detected at the λ_{max} of the compound.

Unless stated otherwise, the Shimadzu system was used for purification.

Nuclear magnetic resonance spectra were obtained using a Bruker Avance 400 MHz (1H, 400 MHz; ^{13}C, 100 MHz) or a 500 MHz (^{1}H, 500 MHz; 13C, 125 MHz) instrument. Chemical shifts (δ) have been reported in parts per million (ppm) and coupling constants (J) in hertz (Hz). Where applicable, splitting patterns have been described as singlet (s), doublet (d), triplet (t), quartet (q), and multiplet (m).

Product purity was assessed by analytic HPLC, run on a Shimadzu LC-2030C 3d plus system, equipped with an Aeris 3.6 μm XB-C18 column. A gradient of MeCN and water, both containing 0.1% TFA was run at a flow rate of 1.5 mL/min. Chromatograms reported show absorbance at 220 nm.

Peptide synthesis

Solid phase peptide synthesis

Standard solid phase peptide synthesis was conducted through a Fmoc/*tert*-butyl approach, using a Liberty Blue™ Automated Microwave Peptide Synthesiser (CEM). Synthesis was conducted on Tentagel-S-RAM Rink amide resin (0.24 mmol/g loading) on a 0.05 mmol scale. Coupling reactions were conducted by addition of a solution of the Fmoc-protected amino acid (0.25 mmol, 5 eq, 0.2 M), Oxyma (0.25 mmol, 5 eq, 0.5 M), and DIC (0.50 mmol, 10 eq, 0.5 M) in DMF. The reaction is heated to 90°C by the microwave reactor for 2 min (single coupling) or 2 x 2 min (double coupling). Deprotection was carried out using piperidine (10%) and Oxyma (0.1 M) in DMF, and heating to 90°C using the microwave reactor for 1.5 min. Where necessary, acetylation of the N-terminus was carried out by addition of acetic anhydride (5% v/v) and DIPEA (5% v/v) in DMF, and heating to 65°C for 2 min using the microwave reactor. After synthesis, the resin was washed with DMF (5 x 10 mL) and DCM (5 x 10 mL).

Peptide 1: Ac-SQETFSDLWKLLPENNVC-NH_2

Fluorescein labelling

Simultaneous side chain deprotection and cleavage of **peptide 1** was conducted for 3 h at rt, using a cleavage cocktail composed of 92.5% TFA, 2.5% TIPS, 2.5% 2,2′-(ethylenedioxy)diethanethiol (DODT), and 2.5% water (5 mL). Peptide was precipitated from the cleavage solution by addition of cold diethyl ether (0°C, 35 mL). The precipitate was isolated and

washed with cold diethyl ether (3 x 20 mL). Peptide was then redissolved in a mixture of MeCN and water, and dried by lyophilisation, before purification by HPLC. LC-MS (50–98% MeCN, Waters system, peptide column) Rt = 8.18 min; m/z 1083.10 ($[M+2H]^{+2}$).

Fluorescein labelling of the cysteine residue was conducted by reaction of purified **peptide 1** (11 mg, 0.0051 mmol) in 1 x phosphate buffered saline (PBS) pH 7.4 (3 mL) with fluorescein-5-maleimide (4.5 mg, 0.011 mmol, 2 eq) in dimethyl sulfoxide (DMSO; 0.5 mL) for 2 h at rt. The resulting product was purified by HPLC. Fluorescein-labelled p53 was isolated as a pale yellow solid (1.7 mg, 13%). HRMS (m/z) for $[M+2H]^{+2}$: 1296.5213.

Oligobenzamide monomers

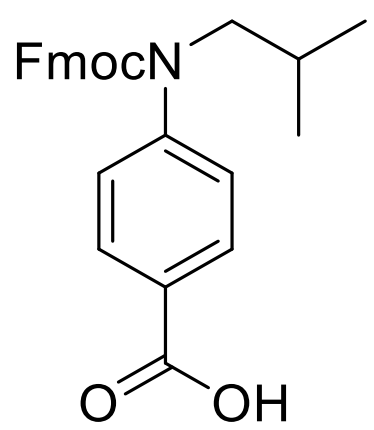

A

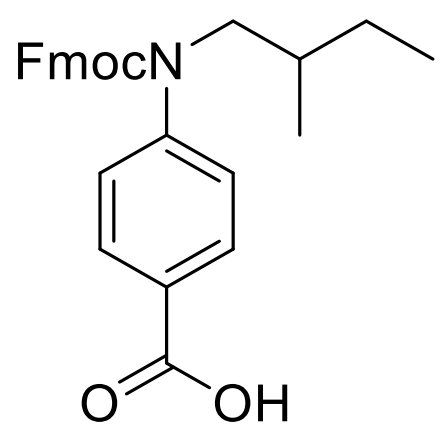

B

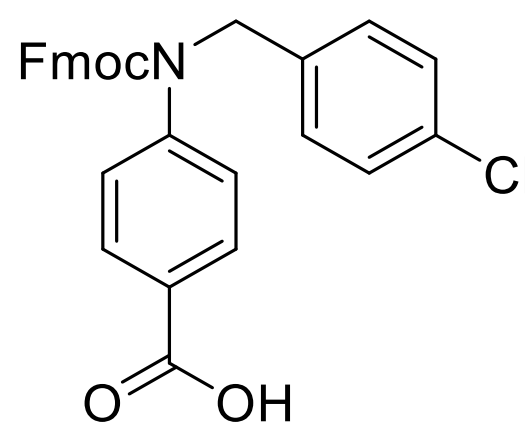

C

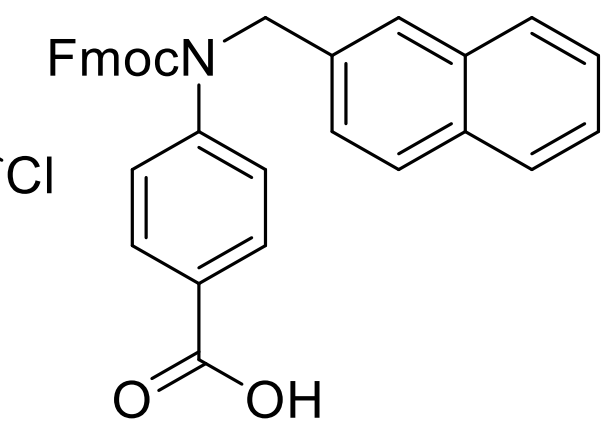

D

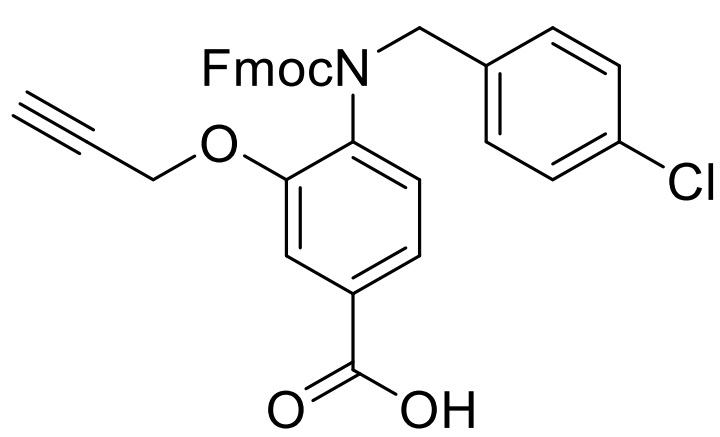

E

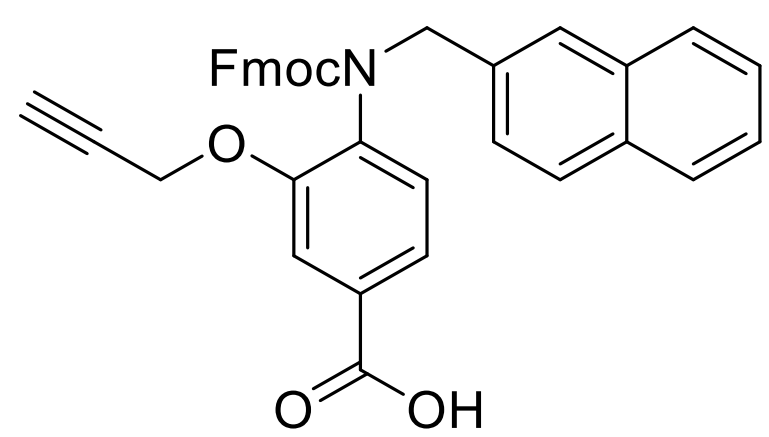

F

Monomers A-F were synthesised as described previously.[5]

Oligobenzamide helix mimetics and probes

General synthetic procedure

The general procedure, adapted from reference,[6] is shown below. Where probe synthesis required a deviation in procedure from the general synthesis, this has been indicated next to the molecule in the characterisation data below. Post cleavage, mimetics were purified by semi-preparative HPLC (H_2O/MeCN with 0.1% TFA modifier, Phenomenex Aeris Peptide 5 μm XB-C18 column (150 x 21 mm, 100 Å))

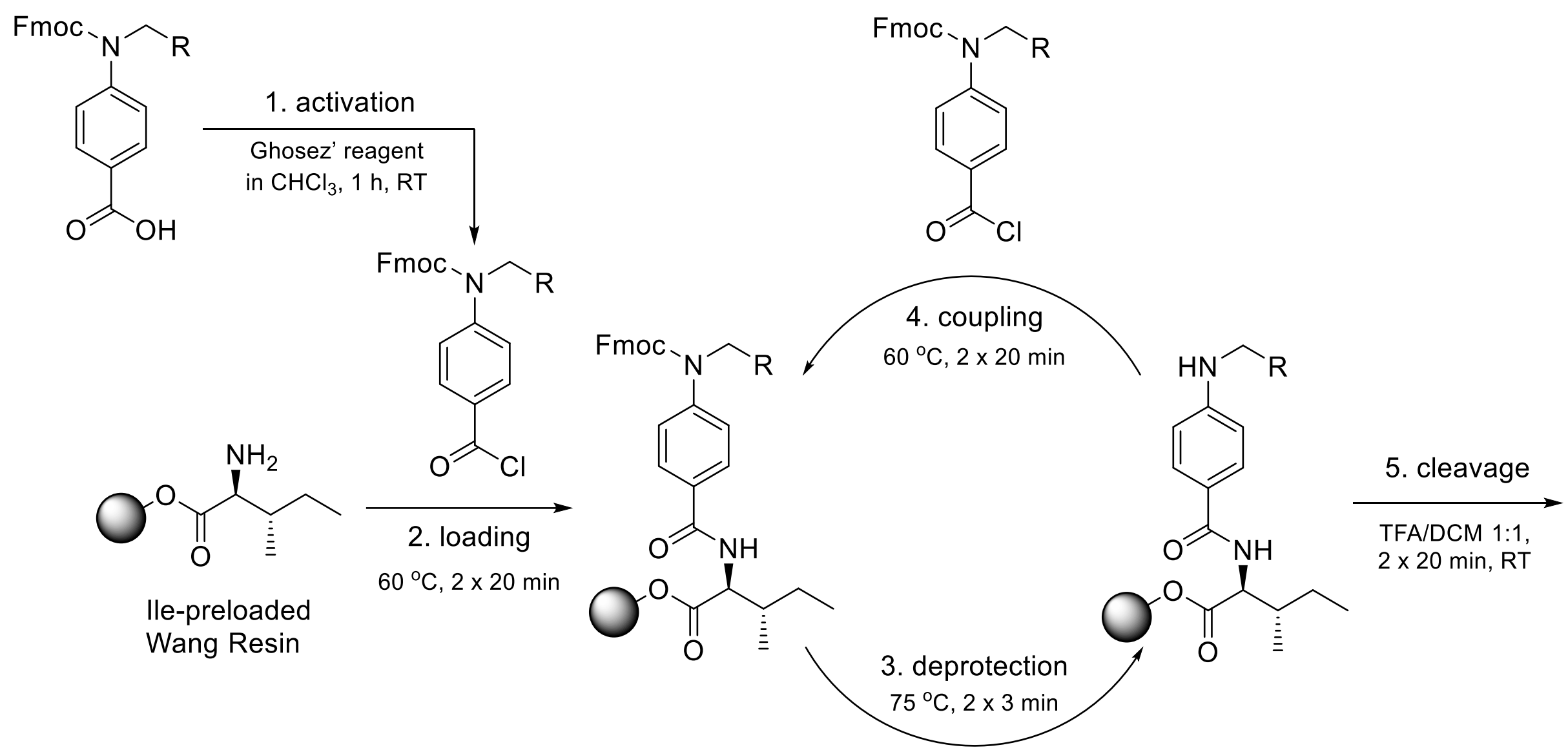

***Scheme 1**: General procedure for automated synthesis of oligobenzamide mimetics on solid phase.*

Oligobenzamide characterisation data

(4-(4-(4-(isobutylamino)-N-(naphthalen-2-ylmethyl)benzamido)-N-(2-methylbutyl)benzamido)benzoyl)-L-alloisoleucine **(OB-a)**

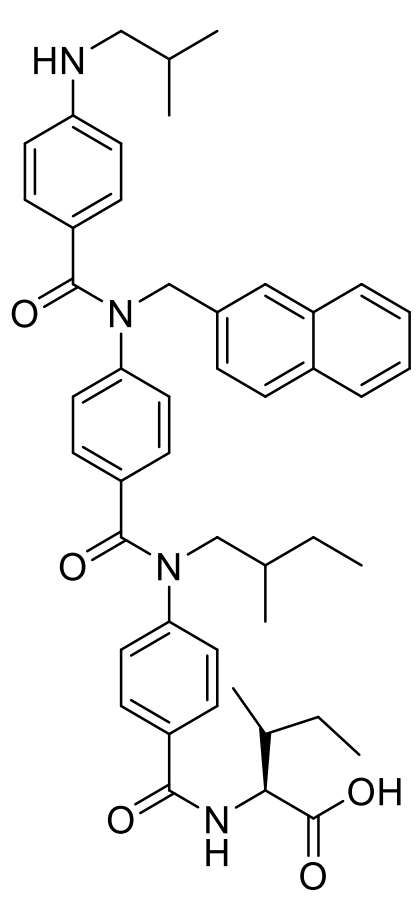

OB-a was synthesised as reported previously.[5]

^{1}H NMR (400 MHz, $CDCl_3$) δ 0.86 (t, *J* = 7.4 Hz, 3H, 3-Hδ), 0.90 (dd, *J* = 6.7, 4.4 Hz, 3H, 3-Hε), 0.97 (t, *J* = 7.1 Hz, 3H, 4-Hδ), 0.99 – 1.02 (m, 9H, 1-Hγ, 4-Hε), 1.11 – 1.23 (m, 1H, 3-Hγ'), 1.22 – 1.34 (m, 1H, 4-Hγ'), 1.36 – 1.48 (m, 1H, 3-Hγ), 1.52 – 1.68 (m, 2H, 3-Hβ, 4-Hγ), 1.84 – 1.99 (m, 1H, 1-Hβ), 2.01 – 2.14 (m, 1H, 4-Hβ), 2.86 – 3.00 (m, 2H, 1-Hα), 3.73 – 3.89 (m, 2H, 3-Hα), 4.69 – 4.76 (m, 1H, 4-Hα), 5.14 – 5.27 (m, 2H, 2-Hα), 6.42 – 6.54 (m, 2H, ArH), 6.70 – 6.84 (m, 3H, 4-NH, ArH), 6.94 – 7.02 (m, 2H, ArH), 7.07 (d, *J* = 8.2 Hz, 2H, ArH), 7.09 – 7.18 (m, 2H, ArH), 7.39 (dd, *J* = 8.5, 1.7 Hz, 1H, NapH), 7.46 (dt, *J* = 6.3, 3.4 Hz, 2H, ArH), 7.58 – 7.64 (m, 2H, ArH), 7.67 (s, 1H, NapH), 7.73 – 7.78 (m, 2H, ArH), 7.80 (dd, *J* = 6.1, 3.4 Hz, 1H, NapH). ^{13}C NMR (101 MHz, $CDCl_3$) δ 174.0 (*C*OOH), 170.5 (*C*ONH), 170.1 (*C*ONH), 166.7 (*C*ONH), 146.7 (Ar*C*), 145.4 (Ar*C*), 134.9 (Ar*C*), 134.2 (Ar*C*), 133.3 (Ar*C*), 132.7 (Ar*C*), 131.3 (Ar*C*), 129.4 (Ar*C*), 128.3 (Ar*C*), 128.1 (Ar*C*), 127.9 (Ar*C*), 127.6 (Ar*C*), 127.4 (Ar*C*), 127.1 (Ar*C*), 126.9 (Ar*C*), 126.3 (Ar*C*), 126.1 (Ar*C*), 125.9 (Ar*C*), 118.4 (Ar*C*), 113.3 (Ar*C*), 57.3 (*C*H), 55.3 (*C*H_2), 54.1 (*C*H_2), 53.2 (*C*H_2), 37.6 (*C*H), 33.3 (*C*H), 27.4 (*C*H), 27.0 (*C*H_2), 25.3 (*C*H_2), 20.4 (2*C*H_3), 16.9 (*C*H_3), 15.6 (*C*H_3), 11.6 (*C*H_3), 11.2 (*C*H_3). LC-MS found 755.803 $[M+H]^+$; HRMS (ESI+) calcd for $C_{47}H_{55}N_4O_5$ $[M+H]^+$ = 755.4172, observed 755.4183.

(4-(4-(N-(4-chlorobenzyl)-4-(isobutylamino)benzamido)-N-isobutylbenzamido)benzoyl)-L-alloisoleucine **(OB-b)**

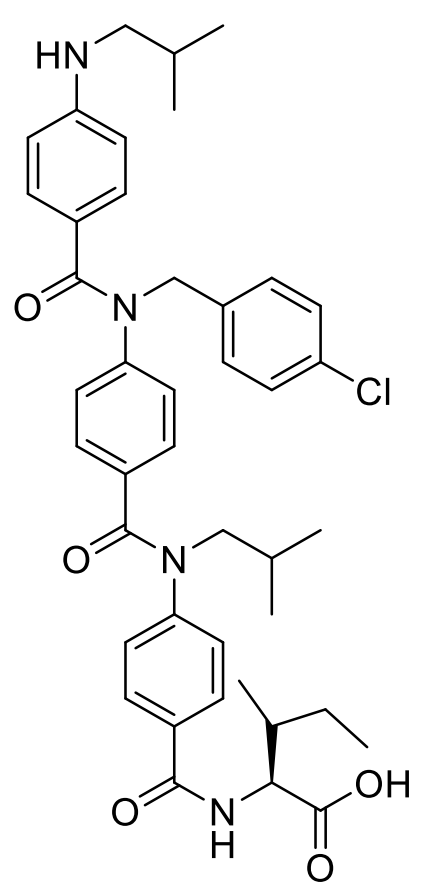

OB-b was synthesised as reported previously.[5]

<u>^{1}H NMR</u> (400 MHz, MeOD) 0.92 (d, 6H, J = 6.6 Hz, 3-Hγ), 0.95 (t, 3H, J = 7.4 Hz, 4-Hδ), 0.95 (d, 6H, J = 6.7 Hz, 1-Hγ), 1.01 (d, 3H, J = 6.9 Hz, 4-Hε), 1.38-1.26 (m, 2H, 4-Hγ, NH), 1.66-1.55 (m, 1H, 4-Hγ'), 1.90-1.77 (m, 2H, 1-Hβ, 3-Hβ), 2.06-1.96 (m, 1H, 4-Hβ), 2.88 (d, 2H, J = 6.9 Hz, 1-Hα), 3.79 (d, 2H, J = 7.4 Hz, 3-Hα), 4.56 (d, 1H, J = 6.2 Hz, 4-Hα), 5.06-4.94 (m, 2H, 2-Hα), 6.33 (d, 2H, J = 8.8 Hz, 1-H3), 6.84 (d, 2H, J = 8.5 Hz, 2-H3), 7.00 (d, 2H, J = 8.8 Hz, 1-H2), 7.18-7.11 (m, 6H, 2-H2, 2-HAr2, 3-H3), 7.24-7.19 (m, 2H, 2-HAr3), 7.73 (d, 2H, J = 8.6 Hz, 3-H2). <u>^{13}C NMR</u> (101 MHz, MeOD) δ 174.9 (*C*OOH), 173.0 (*C*ONH), 172.5 (*C*ONH), 169.4 (*C*ONH), 152.2 (Ar*C*), 147.2 (Ar*C*), 146.4 (Ar*C*), 137.5 (Ar*C*), 135.7 (Ar*C*), 134.2 (Ar*C*), 133.8 (Ar*C*), 132.3 (Ar*C*), 131.1 (Ar*C*), 130.2 (Ar*C*), 129.7 (Ar*C*), 129.5 (Ar*C*), 129.0 (Ar*C*), 128.5 (Ar*C*), 122.6 (Ar*C*), 112.1 (Ar*C*), 58.9 (*C*H), 57.8 (*C*H_2), 53.8 (*C*H_2), 52.3 (*C*H_2), 40.4 (DMSO imp.), 38.2 (*C*H), 29.0 (*C*H), 28.2 (*C*H), 26.7 (*C*H_2), 20.8 (2*C*H3), 20.5 (2*C*H3), 16.1 (*C*H_3), 11.7 (*C*H3). <u>LC-MS</u> found 725.5 $[M+H]^+$. <u>HRMS</u> (ESI+) calcd for $C_{42}H_{50}N_4O_5Cl$ $[M+H]^+$ = 725.3464, observed 725.3464.

(4-(4-(4-((((9H-fluoren-9-yl)methoxy)carbonyl)(isobutyl)amino)-N-(naphthalen-2-ylmethyl)benzamido)-N-(2-methylbutyl)benzamido)benzoyl)-L-alloisoleucine **(OB-a-i)**

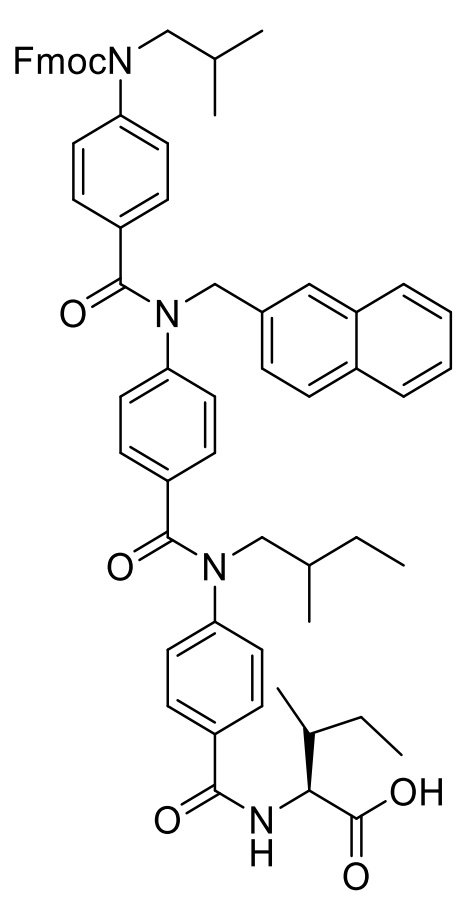

Fmoc-Ile-Wang resin (208 mg, 0.1 mmol) was swelled in 10 mL DMF for 30 min, before being further modified via the general oligobenzamide synthesis method above, according to established methods,[5] with omission of the final Fmoc-deprotection step. The crude product was purified by HPLC and then freeze dried to obtain product (23.2 mg, 24% yield). <u>LC-MS</u> expected 977.48 $[M+H]^+$, observed 977.65 $[M+H]^+$

(4-(4-(4-((((9H-fluoren-9-yl)methoxy)carbonyl)(isobutyl)amino)-N-(4-chlorobenzyl)benzamido)-N-isobutylbenzamido)benzoyl)-L-alloisoleucine **(OB-b-i)**

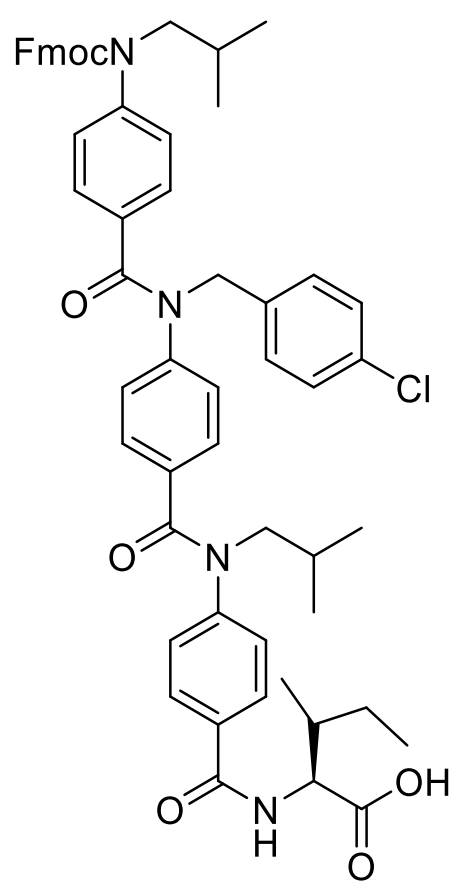

Fmoc-Ile-Wang resin (208 mg, 0.1 mmol) was swelled in DMF (10 mL) for 30 min, before being further modified via the general oligobenzamide synthesis method above, according to established methods,[5] with omission of the final Fmoc-deprotection step. Purified by HPLC and then freeze dried to obtain product (21.1 mg, 22% yield). LC-MS expected 947.41, observed 947.70 $[M+H]^+$

N-(4-(((2S,3R)-1-((2-(3-(but-3-yn-1-yl)-3H-diazirin-3-yl)ethyl)amino)-3-methyl-1-oxopentan-2-yl)carbamoyl)phenyl)-4-(4-(isobutylamino)-N-(naphthalen-2-ylmethyl)benzamido)-N-(2-methylbutyl)benzamide **(OB-a1)**

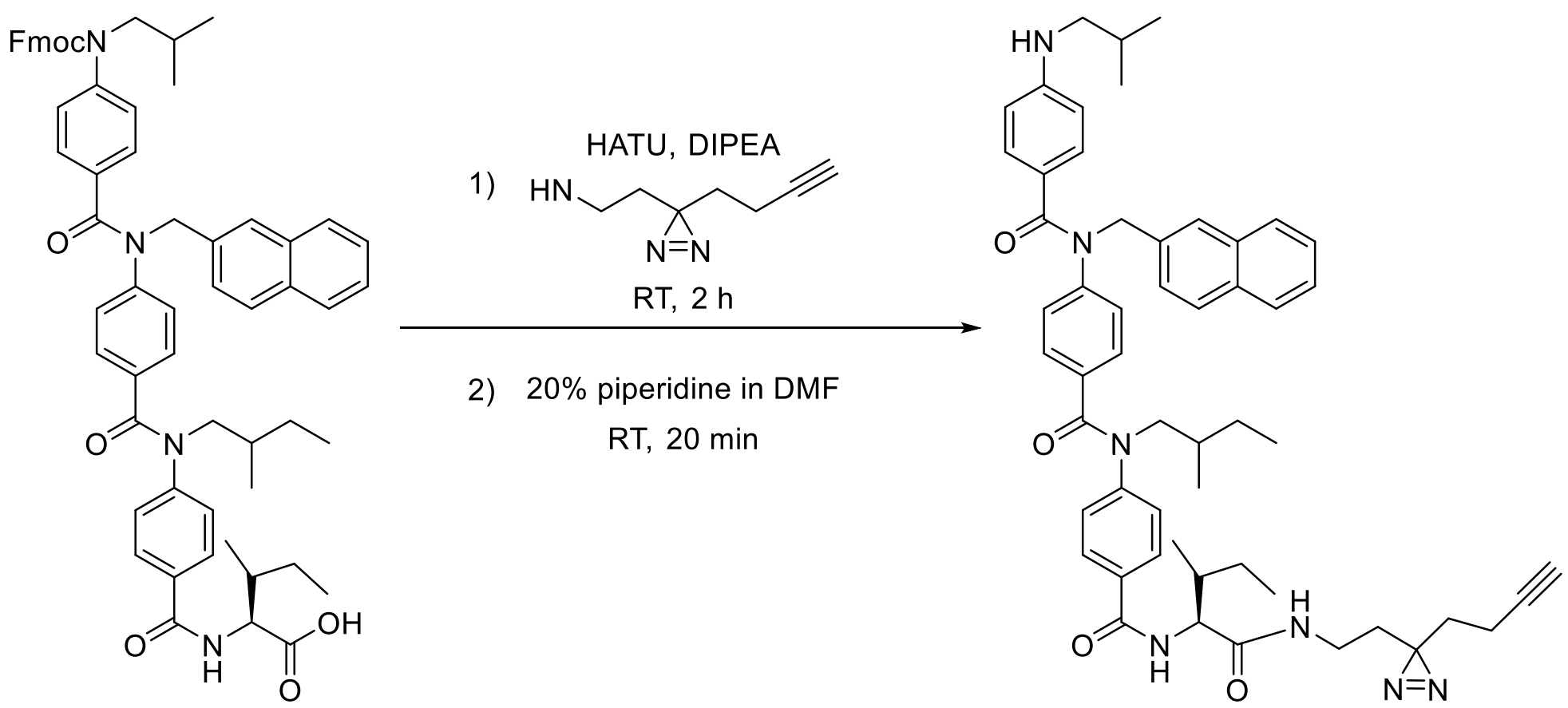

To **OB-a-i** (14.8 mg, 0.015 mmol) in DMF (300 uL), was added HATU (8.6 mg, 0.023 mmol) and DIPEA (5.81 uL, 0.033 mmol). After 5 minutes, 2-(3-(But-3-yn-1-yl)-3*H*-diazirin-3-yl)ethan-1-amine (2.33 uL, 0.018 mmol) was added. The reaction was monitored by LCMS. Upon reaction completion, 20% piperidine in DMF was added for 20 min to facilitate Fmoc group deprotection. The solvent was removed under nitrogen flow and the crude material purified by HPLC to give the final product (6.0 mg, 45% yield). ^{1}H NMR (400 MHz, $CDCl_3$, 328 K) δ 0.86 (t, *J* = 7.4 Hz, 3H, 3-Hδ), 0.89 – 0.93 (m, 3H, 3-Hε), 0.95 – 1.06 (m, 12H, 1-Hγ, 4-Hδ, 4-Hε), 1.13 – 1.23 (m, 1H, 3-Hγ'), 1.28 – 1.33 (m, 1H, 4-Hγ'), 1.38 – 1.49 (m, 1H, 3-Hγ), 1.52 – 1.64 (m, 1H, 4-Hγ), 1.64 – 1.77 (m, 5H, 3-Hβ, 4-tagNHCH2CH_2, 4-tagHCCCH2CH_2), 1.87 – 1.95 (m, 1H, 1-Hβ), 1.97 – 2.06 (m, 4H, 4-Hβ, 4-tagHCCCH_2, 4-tagCC*H*), 2.96 (d, *J* = 6.7 Hz, 2H, 1-Hα), 3.16 (dp, *J* = 17.9, 6.1 Hz, 2H, 4-NHCH_2), 3.73 – 3.91 (m, 2H, 3-Hα), 4.40 – 4.49 (m, 0.4H, 4-Hα), 4.56 (dd, *J* = 8.4, 5.8 Hz, 0.7H, 4-Hα), 5.11 – 5.28 (m, 2H, 2-Hα), 5.92 (s, 1H, 4-N*H*-tag), 6.32 (dd, *J* = 8.8, 2.5 Hz, 2H, ArH), 6.73 (d, *J* = 8.9 Hz, 1H, 4-N*H*), 6.78 (dd, *J* = 8.5, 1.8 Hz, 2H, ArH), 6.99 (dd, *J* = 8.4, 3.4 Hz, 2H, ArH), 7.05 – 7.09 (m, 2H, ArH), 7.09 – 7.14 (m, 2H, ArH), 7.36 – 7.47 (m, 3H, ArH), 7.63 (dd, *J* = 8.6, 4.4 Hz, 2H, ArH, 2-NapH),

7.68 (s, 1H, ArH), 7.75 (d, *J* = 7.8 Hz, 2H, ArH), 7.78 – 7.82 (m, 1H, 2-NapH). ^{13}C NMR (101 MHz, $CDCl_3$) δ 171.9, 169.6, 166.5, 146.6, 145.4, 145.2, 135.0, 134.9, 134.1, 133.3, 132.7, 131.2, 131.1, 129.8, 129.7, 128.3, 128.1, 127.9, 127.6, 127.3, 127.2, 127.0, 126.4, 126.1, 125.8, 82.6, 77.3, 77.2, 69.6, 58.9, 58.2, 55.3, 55.2, 54.0, 37.5, 34.6, 33.3, 33.3, 32.4, 32.0, 27.2, 27.1, 27.0, 26.9, 26.6, 26.1, 25.2, 20.4, 20.3, 16.9, 16.8, 15.4, 14.9, 13.2, 11.5, 11.2, 11.0. Additional peaks observed due to the presence of rotamers. LC-MS found 874.9 $[M+H]^+$. HRMS (ESI+) calcd for $C_{54}H_{64}N_7O_4$ $[M+H]^+$ = 874.5014, observed 874.5008.

N-(4-(((2S,3R)-1-((2-(3-(but-3-yn-1-yl)-3H-diazirin-3-yl)ethyl)amino)-3-methyl-1-oxopentan-2-yl)carbamoyl)phenyl)-4-(N-(4-chlorobenzyl)-4-(isobutylamino)benzamido)-N-isobutylbenzamide **(OB-b1)**

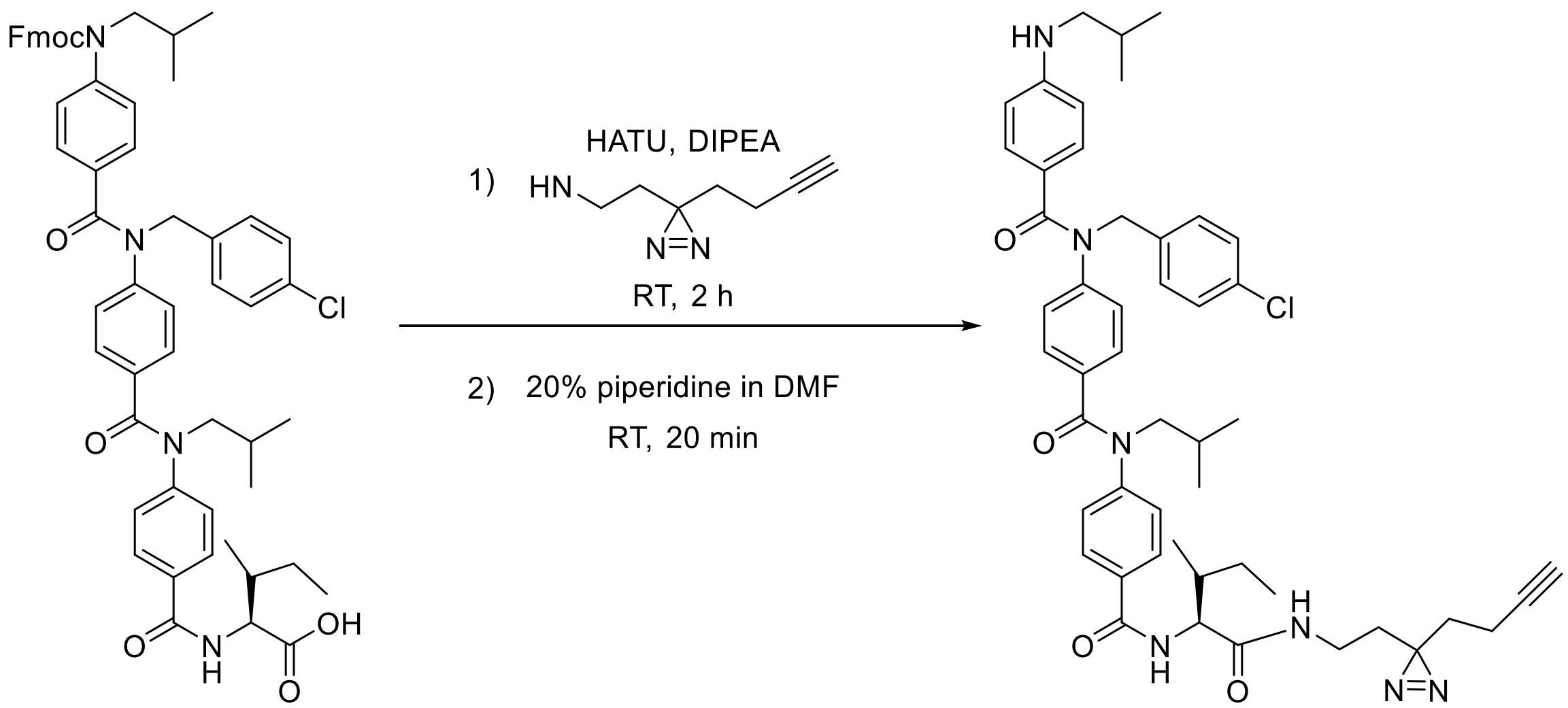

To **(OB-b-i)** (5.7 mg, 0.006 mmol) in DMF (300 uL), was added HATU (3.4 mg, 0.009 mmol) and DIPEA (2.30 uL, 0.013 mmol). After 5 minutes, 2-(3-(But-3-yn-1-yl)-3*H*-diazirin-3-yl)ethan-1-amine (0.9 uL, 0.007 mmol) was added. The reaction was monitored by LCMS. Upon reaction completion, 20% piperidine in DMF was added for 25 min to facilitate Fmoc group deprotection. The solvent was removed under nitrogen flow and purified by HPLC to give the final product (4.2 mg, 21% yield). ^{1}H NMR (400 MHz, $CDCl_3$, 298 K) δ 0.88 – 0.93 (m, 6H, 3-Hγ), 0.93 – 0.98 (m, 9H, 1-Hγ, 4-Hδ) 1.12 – 1.37 (m, 4H, 4-Hγ', 4-Hε), 1.46 – 1.65 (m, 5H, 4-Hγ, 4-tagNHCC*H*₂, 4-tagNHCCCC*H*₂), 1.61 – 1.68 (m, 1H), 1.69 – 1.77 (m, 3H), 1.84 – 1.97 (m, 2H, 1-Hβ, 3-Hβ), 1.99 – 2.05 (m, 4H, 4-Hβ, 4-tagHCCC*H*₂, 4-tagCC*H*), 2.90 (d, *J* = 6.7 Hz, 2H, 1-Hα), 3.00 – 3.21 (m, 2H, 4-tagNHC*H*₂), 3.67 – 3.83 (m, 2H, 3-Hα), 4.40 (app. t, *J* = 7.9 Hz, 0.5H, 4-Hα), 4.52 (dd, *J* = 8.6, 6.0 Hz, 0.5H, 4-Hα), 4.88 – 4.99 (m, 2H, 2-Hα), 5.95 – 6.03 (m, 1H, 4-N*H*-tag), 6.30 (app. t, *J* = 8.4 Hz, 2H, Ar*H*) 6.70 (dd, *J* = 8.6, 2.3 Hz, 2H, Ar*H*), 6.81 – 6.92 (m, 1H, 4-N*H*), 6.97 (dd, *J* = 8.5, 4.7 Hz, 2H, Ar*H*), 7.00 – 7.07 (m, 4H, Ar*H*) , 7.13 (app. d, *J* = 8.4 Hz, 2H, Ar*H*), 7.19 (app. d, *J* = 8.3 Hz, 2H, Ar*H*), 7.61 (dd, *J* = 8.5, 4.6 Hz, 2H, Ar*H*). ^{13}C NMR (101 MHz, $CDCl_3$) δ 171.5, 171.3, 170.5, 166.4, 146.9, 145.9, 136.4, 134.0, 133.2, 131.6, 131.4, 130.0, 129.9, 129.6, 129.5, 128.7, 128.2, 127.6, 127.1, 111.7, 111.5, 82.8, 77.4, 69.8, 58.4, 57.6, 56.9, 53.3, 51.8, 38.3, 37.8, 34.7, 32.6, 32.2, 28.0, 27.2, 26.8, 26.4, 25.3, 20.6,20.3, 20.3, 15.7,

14.9, 13.3, 11.9, 11.4. Additional peaks observed due to the presence of rotamers. LC-MS found 844.7538 [M+H]$^+$. HRMS (ESI+) calcd for $C_{49}H_{59}N_7O_4Cl$ [M+H]$^+$ = 844.4312, observed 844.4324.

N1-(4-((4-((4-(((2S,3R)-1-amino-3-methyl-1-oxopentan-2-yl)carbamoyl)phenyl)(2-methylbutyl)carbamoyl)phenyl)(naphthalen-2-ylmethyl)carbamoyl)phenyl)-N4-(2-(3-(but-3-yn-1-yl)-3H-diazirin-3-yl)ethyl)-N1-isobutylsuccinamide **(OB-a2)**

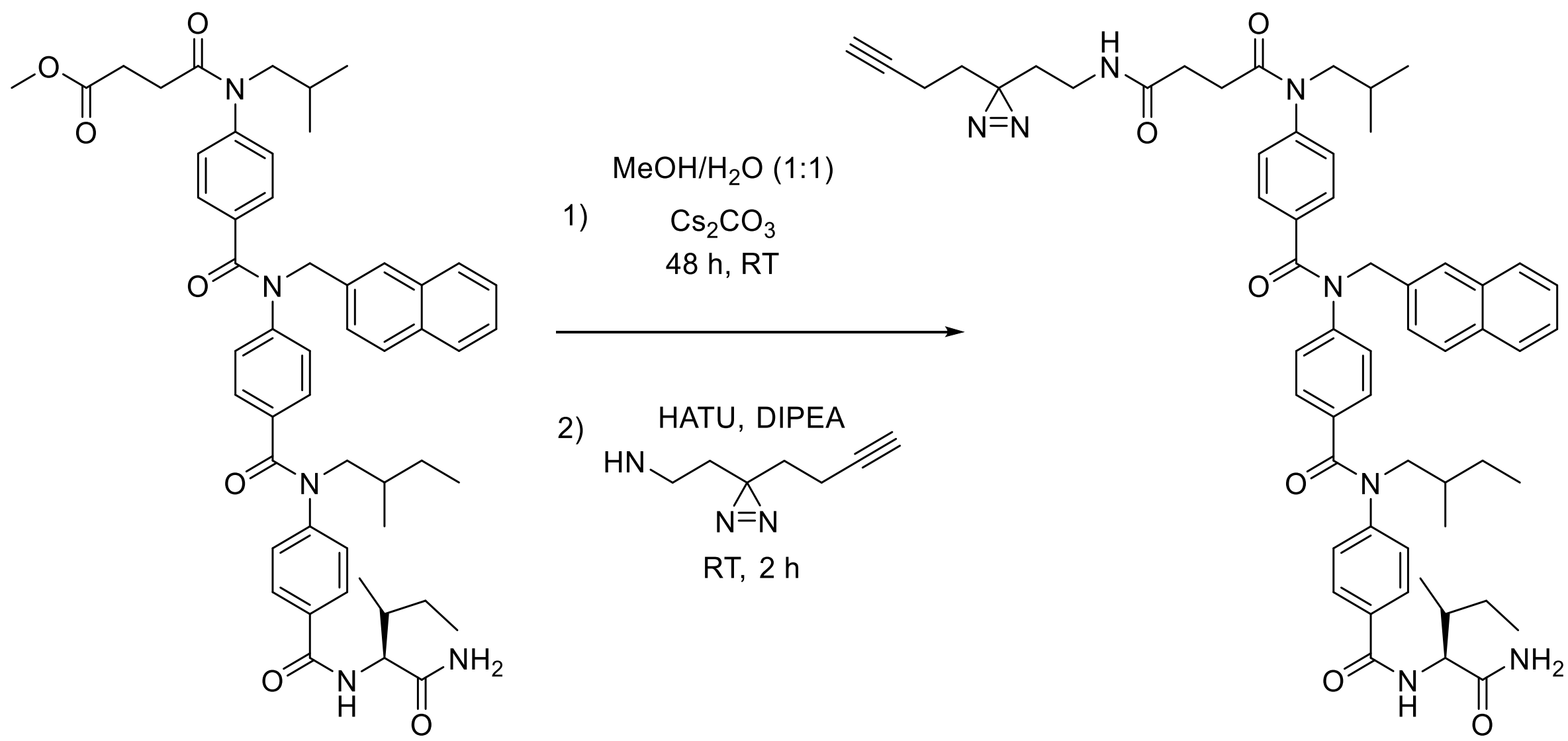

Fmoc-Ile-Rink resin (0.1 mmol) was swelled in 10 mL DMF for 30 min, before being further modified via the general oligobenzamide synthesis method above, with monomethyl hydrogen succinate included as an extra *N*-terminal monomer. The resulting methyl ester was then dissolved in MeOH (500 uL) and H_2O (200 uL), and to the solution was added caesium carbonate (15.7 mg, 0.048 mmol). The reaction was stirred at room temperature for 16 h and monitored by LCMS. Upon reaction completion, the mixture was acidified to pH 4 with HCl (0.1 M) and the product extracted with EtOAc (2 mL). The solvent was removed under nitrogen flow and the crude solid dissolved in DMF (300 uL). To the solution was added HATU (10.6 mg, 0.028 mmol) and DIPEA (12.95 uL, 0.074 mmol). After 5 minutes at room temperature, 2-(3-(But-3-yn-1-yl)-3*H*-diazirin-3-yl)ethan-1-amine was added (2.86 uL, 0.022 mmol). The reaction was stirred at room temperature for 2 h and the reaction monitored by LCMS. The DMF was removed under nitrogen flow, before the crude product was purified by HPLC (6.0 mg, 6% yield over 3 steps). ^{1}H NMR (500 MHz, 328K, $CDCl_3$) δ 0.85 – 0.94 (m, 10H, 1-Hγ, 3-Hδ, 3-Hε), 0.98 (t, *J* = 7.4 Hz, 3H, 4-Hδ), 1.05 (d, *J* = 6.8 Hz, 3H, 4-Hε), 1.14 – 1.50 (m, 7H, 3-Hγ, 4-Hγ'), 1.58 – 1.77 (m, 10H, 1-Hβ, 3-Hβ, 1-tag-CONHCH2CH2C, 1-tag-CONHCH2CH2CCH2, 4-Hγ), 1.98 – 2.06 (m, 3H, 1-tag-HCCCH2, 4-Hβ), 2.24 – 2.38 (m, 2H, 1-tag-NHCOCH2), 2.44 (s, 2H, 1-tagNCOCH2), 3.07 (d, *J* = 6.7 Hz, 2H, 1-tag-CONHCH2), 3.50 – 3.58 (m, 2H, 1-Hα), 3.68 – 3.88 (m, 2H, 3-Hα), 4.48 – 4.61 (m, 1H, 4-Hα), 5.23 (s, 2H, 2-Hα), 5.68 (s, 1H, 4-N*H*H), 6.17 (s, 1H, 4-NH*H*), 6.35 (s, 1H, 1-tag-NH), 6.76 – 6.82 (m, 2H, 2-H3), 6.94 (s, 1H, 4-NH), 7.00 (d, *J* = 8.1 Hz, 2H, 1-H2), 7.08 (dd, *J* = 8.5, 2.5 Hz,

4H, ArH), 7.31 – 7.39 (m, 3H, ArH, 2-NapH), 7.43 – 7.52 (m, 2H, ArH), 7.69 (d, *J* = 8.2 Hz, 3H, ArH, 2-NapH), 7.76 – 7.80 (m, 2H, ArH), 7.81 – 7.85 (m, 1H, 2-NapH). ^{13}C NMR (126 MHz, $CDCl_3$) δ 173.5, 172.9, 172.2, 169.8, 169.2, 166.1, 146.7, 146.6, 144.5, 144.0, 135.2, 134.9, 134.6, 133.4, 133.0, 132.0, 130.6, 129.7, 128.6, 128.2, 128.0, 127.8, 127.5, 127.4, 127.0, 126.4, 126.3, 126.2, 82.9, 69.5, 64.1, 57.8, 56.4, 55.6, 55.5, 54.1, 53.7, 53.5, 43.9, 42.1, 41.1, 37.7, 34.5, 33.5, 33.4, 32.6, 32.2, 31.8, 30.4, 27.1, 27.1, 27.0, 25.3, 20.2, 18.7, 17.6, 17.1, 17.0, 15.6, 13.4, 11.9, 11.5, 11.3. Additional peaks observed due to the presence of rotamers. LC-MS found 973.8829 $[M+H]^+$. HRMS (ESI+) calcd for $C_{58}H_{69}N_8O_6$ $[M+H]^+$ = 973.5335, observed 973.5332.

N1-(4-((4-((4-(((2S,3R)-1-amino-3-methyl-1-oxopentan-2-yl)carbamoyl)phenyl)(isobutyl)carbamoyl)phenyl)(4-chlorobenzyl)carbamoyl)phenyl)-N4-(2-(3-(but-3-yn-1-yl)-3H-diazirin-3-yl)ethyl)-N1-isobutylsuccinamide **(OB-b2)**

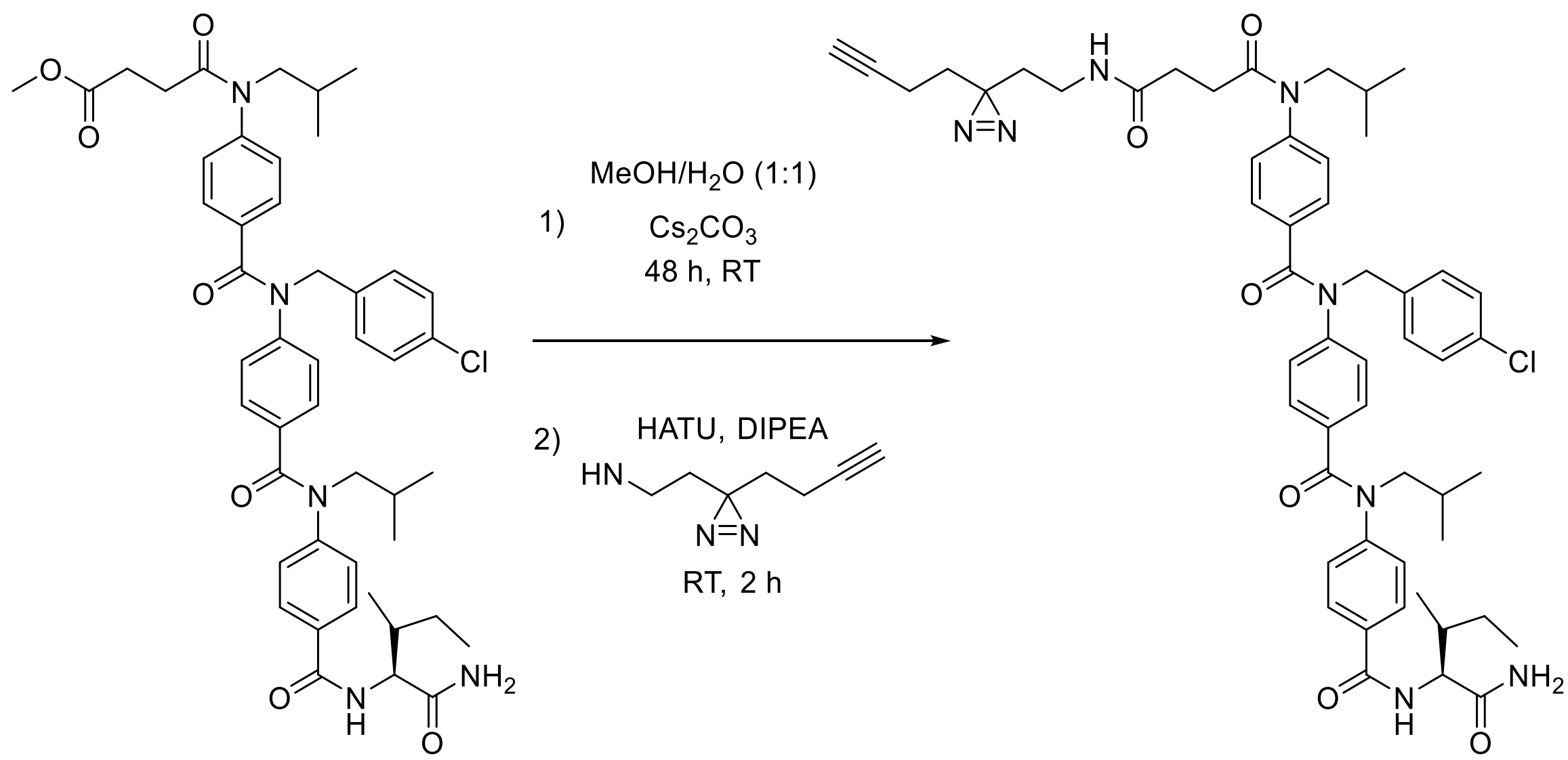

Fmoc-Ile-Rink resin (0.1 mmol) was swelled in 10 mL DMF for 30 min, before being further modified via the general oligobenzamide synthesis method above, with monomethyl hydrogen succinate included as an extra *N*-terminal monomer. The resulting methyl ester was then dissolved in MeOH (500 uL) and H_2O (200 uL), and to the solution was added caesium carbonate (25.0 mg, 0.077 mmol). The reaction was stirred at room temperature for 16 h and monitored by LCMS. Upon reaction completion, the mixture was acidified to pH 4 with HCl (0.1 M) and the product extracted with EtOAc (2 mL). The solvent was removed under nitrogen flow and the resulting crude solid was dissolved in DMF (300 uL). To the solution was added HATU (10.6 mg, 0.028 mmol) and DIPEA (12.93 uL, 0.074 mmol). After 5 minutes at room temperature, 2-(3-(But-3-yn-1-yl)-3*H*-diazirin-3-yl)ethan-1-amine was added (2.86 uL, 0.022 mmol). The reaction was stirred at room temperature for 2 h and the reaction monitored by LCMS. The DMF was removed under nitrogen flow, before the crude product was purified by HPLC (8.0 mg, 8% yield over 3 steps). ^{1}H NMR (500 MHz, 328K, $CDCl_3$) δ 0.88 (d, *J* = 6.7 Hz, 6H, 1-Hγ), 0.94 – 1.00 (m, 9H, 3-Hγ, 4-Hδ), 1.05 (d, *J* = 6.8 Hz, 3H, 4-Hε), 1.22 – 1.35 (m, 1H, 4-Hγ), 1.61 – 1.74 (m, 5H, 1-Hβ, 4-

Hγ', 1-tag-CONHCH$_2$CH_2C, 1-tag-CONHCH$_2$CH$_2$CCH_2), 1.93 (dq, J = 13.8, 6.9 Hz, 1H, 3-Hβ), 2.01 (qd, J = 7.9, 4.0 Hz, 4H, 1-tag-HCCCH_2, 4-Hβ), 2.36-2.26 (m, 2H, 1-tag-NCOCH$_2$CH_2), 2.44 (br s, 2H, 1-tag-NCOCH_2), 3.07 (d, J = 6.5 Hz, 2H, 1-tag-CONHCH_2), 3.47 – 3.57 (m, 2H, 1-Hα), 3.80 (qd, J = 13.6, 7.4 Hz, 2H, 3-Hα), 4.55 (t, J = 7.6 Hz, 1H, 4-Hα), 5.01 (d, J = 1.8 Hz, 2H, 2-Hα), 5.67 (br s, 1H, 4-N*H*H), 6.17 (br s, 1H 4-NH*H*), 6.32 (s, 1H, 1-tag-NH), 6.72 – 6.77 (m, 2H, 2-H3), 6.95 (br s, 1H, 4-NH), 7.02 (d, J = 8.2 Hz, 2H, 1-H2), 7.08 (d, J = 8.5 Hz, 2H, ArH), 7.11 (d, J = 8.5 Hz, 2H, ArH), 7.14 – 7.18 (m, 2H, ArH), 7.26 (d, J = 8.5 Hz, 2H, ArH), 7.28-7.31 (m, 2H, ArH), 7.71 (d, J = 8.4 Hz, 2H, 3-H2). ^{13}C NMR (101 MHz, CDCl$_3$) δ 173.5, 172.8, 172.1, 169.8, 169.2, 166.0, 146.7, 144.2, 135.5, 133.7, 130.6, 129.9, 129.7, 128.9, 128.2, 127.9, 127.6, 127.1, 82.9, 69.5, 57.8, 56.9, 56.4, 53.4, 41.1, 37.7, 34.5, 32.6, 32.2, 31.8, 30.3, 27.2, 27.0, 25.3, 20.3, 20.3, 20.2, 17.6, 15.6, 13.4, 11.5. LC-MS found 943.7858 [M+H]$^+$. HRMS (ESI+) calcd for $C_{53}H_{64}N_8O_6Cl$ [M+H]$^+$ = 943.4632, observed 943.4639.

2-chlorotrityl chloride-loaded methyl 2-((((9H-fluoren-9-yl)methoxy)carbonyl)amino)-4-(3-methyl-3H-diazirin-3-yl)butanoate **(PM-CTC)**

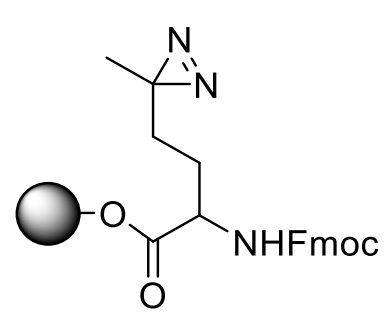

2-chlorotrityl chloride resin (138 mg, 1.6 mmol/g loading), was weighed into a fritted 6 ml syringe, and swelled in dry DCM for 30 min, before addition of DIPEA (0.2 mL, 1.22 mmol) and Fmoc-photomethionine-OH (152 mg, 0.44 mmol). The reaction was shaken in the dark for 16 h at RT, before being washed with DMF (4 x 5 mL) and DCM (3 x 5 mL) to remove excess reagent. The resin was then capped (DCM:MeOH:DIPEA, 17:2:1 ratio, 4 mL, 2 x 5 min). The resin was washed again with DMF (4 x 5 mL) and DCM (3 x 5 mL) to remove any remaining capping solution. The resin was then washed with Et$_2$O (2 x 5 mL) and dried in vacuo. The resin loading was measured with a published spectroscopic method at 290 nm,[7] (0.83 mmol/gram), before being dried under vacuum and stored in the dark.

2-chlorotrityl chloride- loaded methyl 4-((((9H-fluoren-9-yl)methoxy)carbonyl)(isobutyl)amino)benzoate **(A-CTC)**

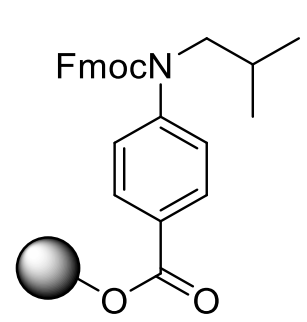

2-chlorotrityl chloride resin (250 mg, 1.6 mmol/gram loading), was weighed into a fritted 6 mL syringe, and swelled in dry DCM for 30 min, before addition of DIPEA (0.35 mL, 2.0 mmol) and Fmoc-monomer **A** (332 mg, 0.8 mmol). The reaction was shaken 16 h at RT. The resin was then capped (DCM:MeOH:DIPEA, 17:2:1 ratio, 4 mL, 2 x 5 min). The resin was washed again with DMF (4 x 5 mL) and DCM (3 x 5 mL) to remove any remaining capping solution. The resin was then washed with Et$_2$O (2 x 5 mL) and dried in vacuo. The resin loading was measured with a published spectroscopic method at 290 nm,[7] (0.97 mmol/gram).

2-chlorotrityl chloride- loaded methyl 4-((((9H-fluoren-9-yl)methoxy)carbonyl)(2-methylbutyl)amino)benzoate **(B-CTC)**

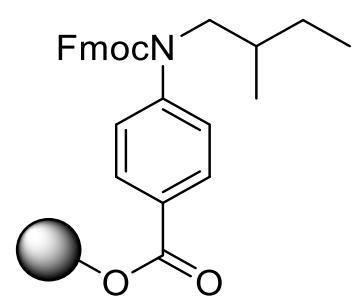

2-chlorotrityl chloride resin (250 mg, 1.6 mmol/gram loading), was weighed into a fritted 6 mL syringe, and swelled in dry DCM for 30 min, before addition of DIPEA (0.35 mL, 2.0 mmol) and Fmoc-monomer **B** (344 mg, 0.8 mmol). The reaction was shaken for 16 h at RT. The resin was then capped (DCM:MeOH:DIPEA, 17:2:1 ratio, 4 mL, 2 x 5 min). The resin was washed again with DMF (4 x 5 mL) and DCM (3 x 5 mL) to remove any remaining capping solution. The resin was then washed with Et_2O (2 x 5 mL) and dried in vacuo. The resin loading was measured with a published spectroscopic method at 290 nm,[7] (0.96 mmol/gram).

4-(4-(4-((((9H-fluoren-9-yl)methoxy)carbonyl)(isobutyl)amino)-N-(naphthalen-2-ylmethyl)benzamido)-N-(2-methylbutyl)-3-(prop-2-yn-1-yloxy)benzamido)benzoic acid **(OB-a-ii)**

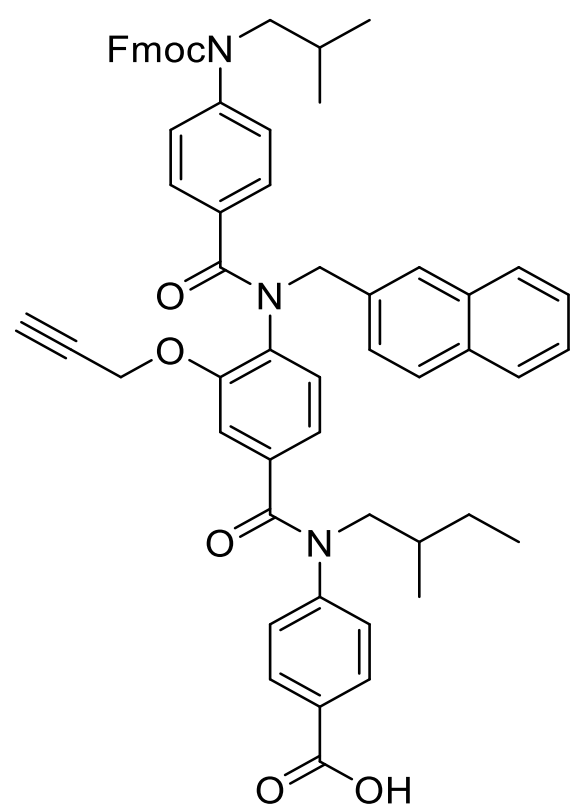

B-CTC resin (103 mg, 0.10 mmol) was added to a microwave vial, swelled in DCM for 30 min, before the addition of Ghosez preactivated monomer **F** in DCM (0.12 mmol, in 2 mL DCM) and DIPEA (0.30 mmol). The coupling reaction was heated under microwave irradiation at 55 °C for 30 min, before repeating the coupling step to push the reaction to completion. The resin was washed with DMF (5 x 5 mL). To the resin was added Ghosez pre-activated monomer **A** (0.12 mmol, in 2 mL DCM) and DIPEA (0.30 mmol), and the coupling reaction was heated under microwave irradiation at 55 °C for 30 min. The coupling step was repeated. The resin was then washed with DMF (5 x 5 mL). The product was cleaved from the resin with 1:1 TFA:DCM (2 x 2 mL for 30 min each). The crude product was dried under nitrogen flow and the presence of desired trimer product confirmed by LCMS analysis. The crude product was used in the next step without further purification.

4-(4-(4-((((9H-fluoren-9-yl)methoxy)carbonyl)(isobutyl)amino)-N-(4-chlorobenzyl)benzamido)-N-isobutyl-3-(prop-2-yn-1-yloxy)benzamido)benzoic acid **(OB-b-ii)**

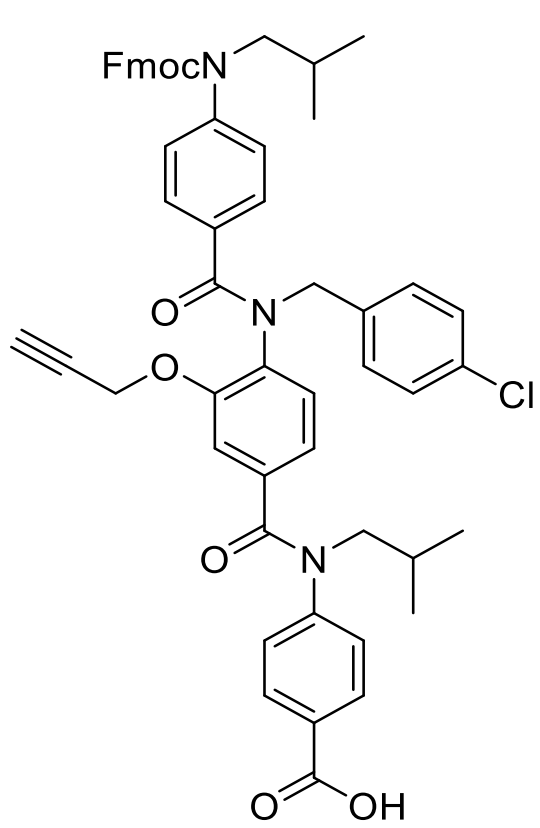

A-CTC resin (104 mg, 0.10 mmol) was swelled in DCM for 30 min, before the addition of Ghosez preactivated monomer **E** in DCM (0.12 mmol, in 2 mL DCM) and DIPEA (0.30 mmol). The coupling reaction was heated under microwave irradiation at 55 °C for 30 min, before repeating the coupling step to push the reaction to completion. The resin was washed with DMF (5 x 5 mL). To the resin was added Ghosez pre-activated monomer **A** (0.12 mmol, in 2 mL DCM) and DIPEA (0.30 mmol), and the coupling reaction was heated under microwave irradiation at 55 °C for 30 min. The coupling step was repeated. The resin was then washed with DMF (5 x 5 mL). The product was cleaved from the resin with 1:1 TFA:DCM (2 x 2 mL for 30 min each). The crude product was dried under nitrogen

flow and the presence of desired trimer product confirmed by LCMS analysis. The crude product was used in the next step without further purification.

2-(4-(4-(4-(isobutylamino)-N-(naphthalen-2-ylmethyl)benzamido)-N-(2-methylbutyl)-3-(prop-2-yn-1-yloxy)benzamido)benzamido)-4-(3-methyl-3H-diazirin-3-yl)butanoic acid **(OB-a3)**

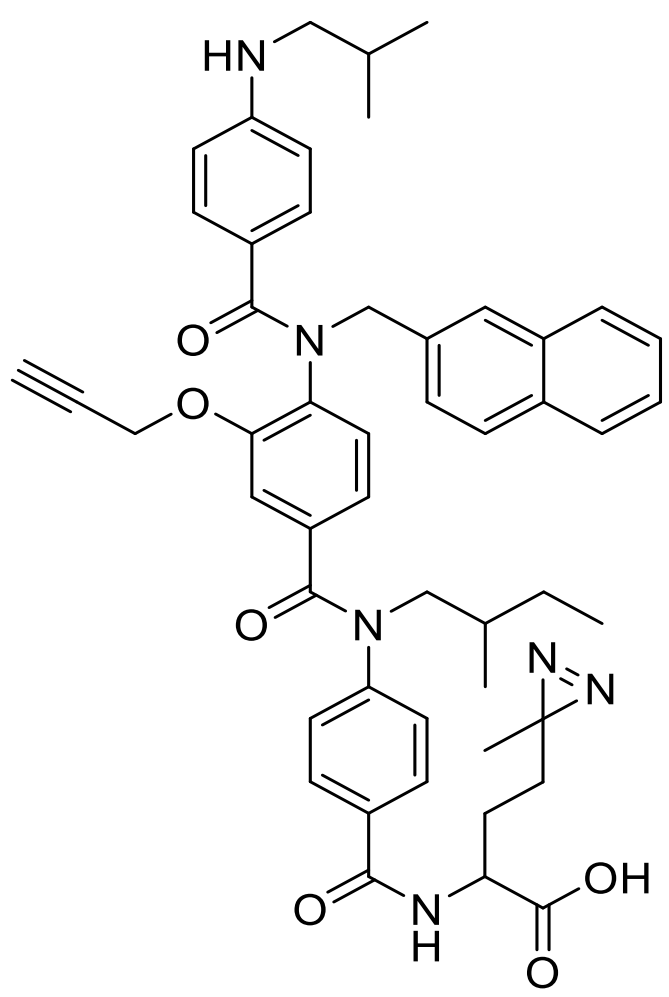

PM-CTC resin (50 mg, 0.041 mmol) was added to a fritted 6 mL syringe, swelled in DMF (5 mL) for 30 min, before deprotection with 20% piperidine in DMF (2 mL, 2 x 20 min). The resin was then washed with DMF (5 x 5 mL).

Meanwhile, to crude Fmoc-protected trimer **OB-a-ii** (46 mg, 70% pure by LCMS), was added DMF (500 µL), DIPEA (25.07 µL, 0.144 mmol) and HATU (24 mg, 0.062 mmol). After 5 minutes, the solution of active ester was added to the deprotected **PM-CTC** resin, and the mixture was shaken in the dark for 2 h at RT. The resin was then washed with DMF (5 x 5 mL), before Fmoc deprotection with 20% piperidine in DMF (1 x 20 min). The crude mixture was purified using HPLC to give the final product (10.8 mg, 32% yield). ^{1}H NMR (500 MHz, 328K, $CDCl_3$) δ 0.85 (t, J = 7.4 Hz, 3H, 3-Hδ), 0.88 (d, J = 6.7 Hz, 3H, 3-Hε), 0.97 (d, J = 6.6 Hz, 6H, 1-Hγ), 0.98 (s, 3H, 4-Hε), 1.21 – 1.11 (m, 1H, 3-Hγ), 1.53 – 1.26 (br m, 5H, 3-Hγ', 4-Hγ, 1-NH), 1.74 – 1.59 (m, 2H, 3-Hβ, 4-Hβ'), 1.96 – 1.83 (m, 2H, 1-Hβ, 4-Hβ), 2.43 (t, J= 2.3 Hz, 1H, 2-OCH_2CCH), 2.90 (d, J = 6.9 Hz, 2H, 1-Hα), 3.79 (dd, J = 7.4, 2.7 Hz, 2H, 3-Hα), 4.35 (s, 2H, 2-OCH_2CCH), 4.73 – 4.68 (m, 1H, 4-Hα), 5.69 (br s, 2H, 2-Hα), 6.36 (d, J = 8.3 Hz, 2H, 2H-2), 6.59 – 6.53 (m, 2H, 2H-2, 2H-3), 6.86 – 6.79 (m, 2H, ArH, 4-NH), 6.93 – 6.87 (m, 2H, 3-H3), 7.15 – 7.08 (m 2H, 2H-3), 7.38 (dd, J = 8.4, 1.8 Hz, 1H, ArH), 7.41 (dd, *J* = 6.2, 3.2 Hz, 2H, ArH), 7.57 – 7.54 (m, 2H, 3-H2), 7.62– 7.60 (m, 1H, ArCH), 7.70 (d, J = 8.2 Hz, 1H, ArH), 7.73 –7.71 (m, 1H, ArH), 7.76 (dd, *J* = 6.1, 3.5 Hz, 1H, ArH). ^{13}C NMR (126 MHz, $CDCl_3$) δ 173.4, 172.7, 172.0, 169.7, 169.1, 166.0, 146.5, 144.4, 143.9, 135.0, 134.8, 134.5, 133.3, 132.8, 131.9, 130.5, 129.5, 128.5, 128.1, 127.9, 127.7, 127.4, 127.2, 126.9, 126.2, 126.1, 126.0, 82.7, 77.3, 77.0, 76.8, 69.4, 64.0, 57.7, 56.3, 55.5, 55.4, 54.0, 53.5, 53.4, 43.8, 41.9, 41.0, 37.6, 34.4, 33.4, 33.3, 32.5, 32.1, 31.7, 30.2, 27.0, 27.0, 26.9, 25.2, 20.1, 18.6, 17.5, 17.0, 16.9, 15.5, 13.3, 11.8, 11.4, 11.2. Additional peaks observed due to the presence of rotamers. LC-MS found 835.4054 $[M+H]^+$. HRMS (ESI+) calcd for $C_{50}H_{55}N_6O_6$ $[M+H]^+$ = 835.4183, observed 835.4189.

2-(4-(4-(N-(4-chlorobenzyl)-4-(isobutylamino)benzamido)-N-isobutyl-3-(prop-2-yn-1-yloxy)benzamido)benzamido)-4-(3-methyl-3H-diazirin-3-yl)butanoic acid **(OB-b3)**

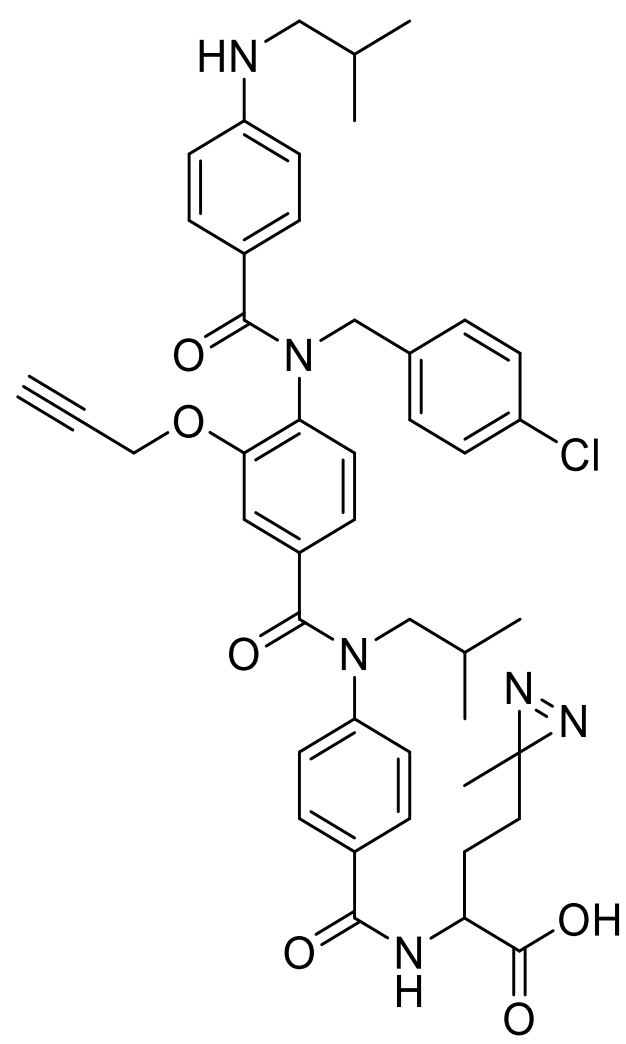

PM-CTC resin (50 mg, 0.041 mmol), was added to a fritted 6 mL syringe, swelled in DMF (5 mL) for 30 min, before deprotection with 20% piperidine in DMF (2 mL, 2 x 20 min). The resin was then washed with DMF (5 x 5 mL).

Meanwhile, to crude Fmoc-protected trimer **OB-b-ii** (44 mg, 50% pure by LCMS), was added DMF (500 µL), DIPEA (21.6 µL, 0.1242 mmol) and HATU (24 mg, 0.062 mmol). After 5 minutes, the solution of active ester was added to the deprotected **PM-CTC** resin, and the mixture was shaken in the dark for 2 h at RT. The resin was then washed with DMF (5 x 5 mL), before Fmoc deprotection with 20% piperidine in DMF (1 x 20 min). The crude mixture was purified using HPLC to give the final product (10.1 mg, 31% yield). ^{1}H NMR (500 MHz, 328K, $CDCl_3$) δ 0.93 (6H, d, *J* = 6.7 Hz, 1-Hγ), 0.97 (6H, d, *J* = 6.6 Hz, 3-Hγ), 1.00 (3H, s, 4-Hε), 1.48 (3H, qdd, *J* = 14.7, 10.3, 5.8 Hz, 4-Hγ, 1-NH), 1.66 – 1.75 (1H, m, 4-Hβ), 1.81 – 1.98 (3H, m, 4-Hβ’, 1-Hβ, 3-Hβ), 2.46 (1H, t, *J* = 2.4 Hz, 2-OCH$_2$CC*H*), 2.89 (2H, d, *J* = 6.7 Hz, 1-Hα), 3.78 (2H, d, *J* = 7.4 Hz, 3-Hα), 4.37 (2H, S, 2-OC*H*$_2$CCH), 4.74 (1H, q, *J* = 6.8 Hz, 4-Hα), 5.05 (brs, 2H, 2-Hα), 6.26 (2H, d, *J* = 8.4 Hz, 1-H3), 6.54 (1H, d, *J* = 8.1 Hz, 2-H3), 6.63 (1H, dd, *J* = 8.1, 1.7 Hz, 2-H2), 6.70 (1H, d, *J* = 7.4 Hz, 4-NH), 6.84 (1H, d, *J* = 1.7 Hz, 2-H1), 6.92 – 6.98 (2H, m, 3-H3), 7.03 – 7.08 (2H, m, 1-H2), 7.13 (2H, d, *J* = 8.6 Hz, 2-HAr2/3), 7.15 – 7.18 (2H, m, 2-HAr2/3), 7.56 – 7.63 (2H, , 3-H2). ^{13}C NMR (126 MHz, $CDCl_3$) δ 173.8, 172.8, 169.4, 165.9, 151.8, 146.8, 136.0, 135.7, 133.6, 133.3, 131.1, 130.7, 130.4, 130.2, 130.0, 129.9, 128.5, 128.2, 127.2, 122.2, 113.8, 111.5, 111.5, 111.2, 76.5, 56.9, 55.9, 52.2, 52.0, 36.1, 32.1, 32.1, 30.7, 29.9, 29.9, 29.7, 29.5, 29.5, 29.4, 29.3, 29.3, 28.0, 27.4, 27.3, 27.2, 27.2, 25.6, 25.5, 22.8, 20.7, 20.3, 19.8, 14.3, 1.2. Additional peaks observed due to the presence of rotamers. LC-MS found 805.4 $[M+H]^+$. HRMS (ESI+) calcd for $C_{45}H_{50}N_6O_6Cl$ $[M+H]^+$ = 805.3480, observed 805.3490.

Pyrrolopyrimidine

PP was synthesised as reported by Lim and coworkers.[4]

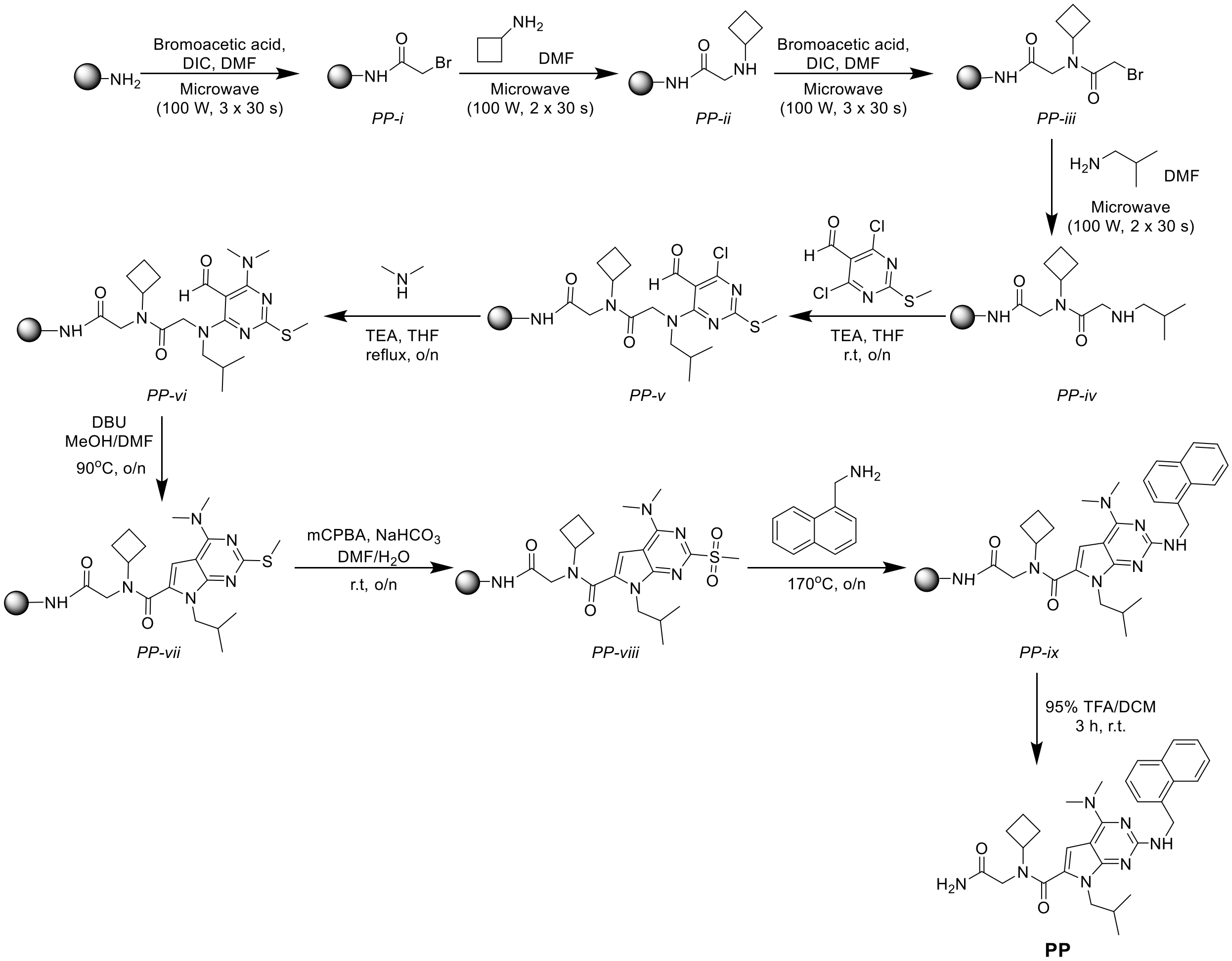

***Scheme 2:** Route for the synthesis of **PP**, modified from Lim and coworkers.[4] Grey circle represents solid support.*

PP

Rink amide-loaded 2-bromoacetic acid **(PP-i)**

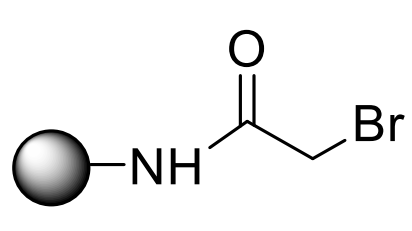

To 500 mg of deprotected Rink amide resin, swollen in DMF, a solution of bromoacetic acid (2 M) and DIC (2 M) in anhydrous DMF (15 mL) was added. The reaction mixture was heated in a microwave reactor at 100 W for 3 x 30 s pulses, with stirring. The resin was washed with DMF (8 x 20 mL) and anhydrous DMF (2 x 20 mL) before proceeding to the next step.

Rink amide-loaded cyclobutylglycine **(PP-ii)**

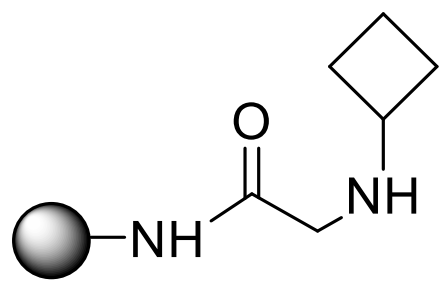

PP-i (500 mg) was treated with a solution of cyclobutylamine (2 M) in anhydrous DMF (15 mL) and heated in a microwave reactor at 100 W for 2 x 30 s pulses, with stirring. The resin was washed with DMF (8 x 20 mL) and anhydrous DMF (2 x 20 mL) before proceeding to the next step.

Rink amide-loaded *N*-(2-bromoacetyl)-*N*-cyclobutylglycine **(PP-iii)**

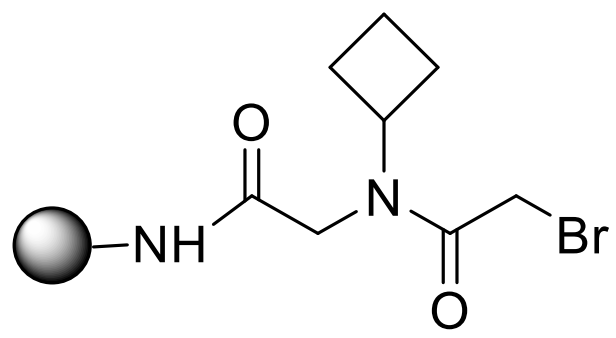

The reaction procedure described for the synthesis of **PP-i** was repeated using **PP-ii**.

Rink amide-loaded *N*-cyclobutyl-*N*-(isobutylglycyl)glycine **(PP-iv)**

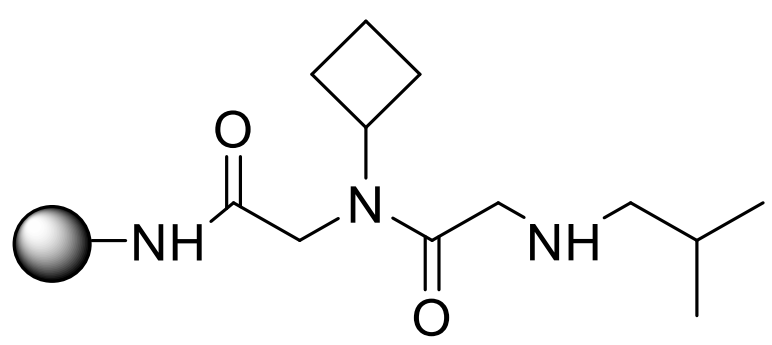

The reaction procedure described for the synthesis of **PP-ii** was repeated using **PP-iii**. Resin was washed with DMF (8 x 20 mL), chloroform (2 x 20 mL), methanol (2 x 20 mL), and DMF (2 x 20 mL). A test cleavage was conducted on an aliquot of resin and analysed by LC-MS. LC-MS (4.5–90% MeCN, Agilent system) Rt = 3.09 min; m/z 242.10 ($[M+H]^+$).

Rink amide-loaded *N*-(*N*-(6-chloro-5-formyl-2-(methylthio)pyrimidin-4-yl)-*N*-isobutylglycyl)-*N*-cyclobutylglycine **(PP-v)**

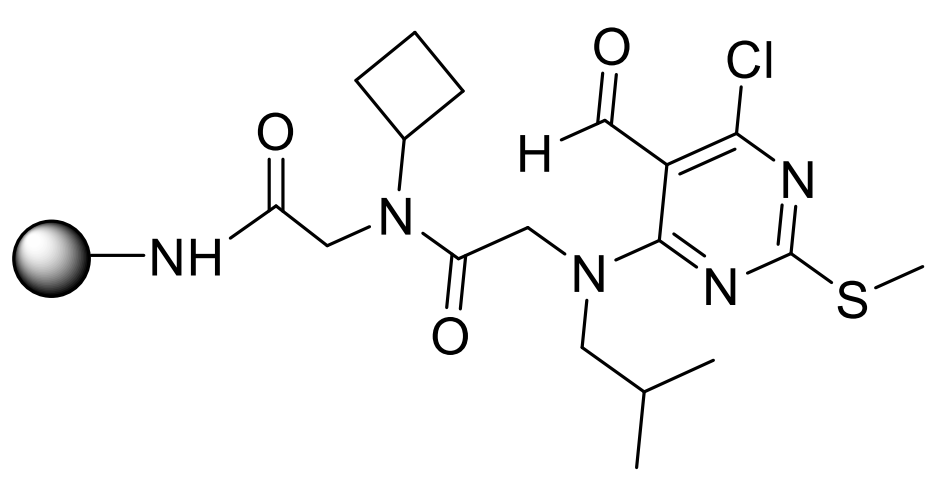

To 1 g of **PP-iv** (up to 0.8 mmol) swollen in THF, a solution of 4,6-dichloro-2-(methylthio)pyrimidine-5-carboxaldehyde (890 mg, 4 mmol, 5 eq) and triethylamine (558 µL, 4 mmol, 5 eq) in THF (15 mL) was added and the reaction was agitated for 18 h at rt. Resin was washed with DMF (3 x 20 mL), DCM (2 x 20 mL), methanol (2 x 20 mL), and DMF (2 x 20 mL). A test cleavage was conducted on an aliquot of resin and analysed by LC-MS. LC-MS (5–95% MeCN, 1 min, ROAR system) Rt = 2.77 min; m/z 428.7 ($[M+H]^+$).

Rink amide-loaded *N*-cyclobutyl-*N*-(4-(dimethylamino)-7-isobutyl-2-(methylthio)-7*H*-pyrrolo[2,3-d]pyrimidine-6-carbonyl)glycine **(PP-vi)**

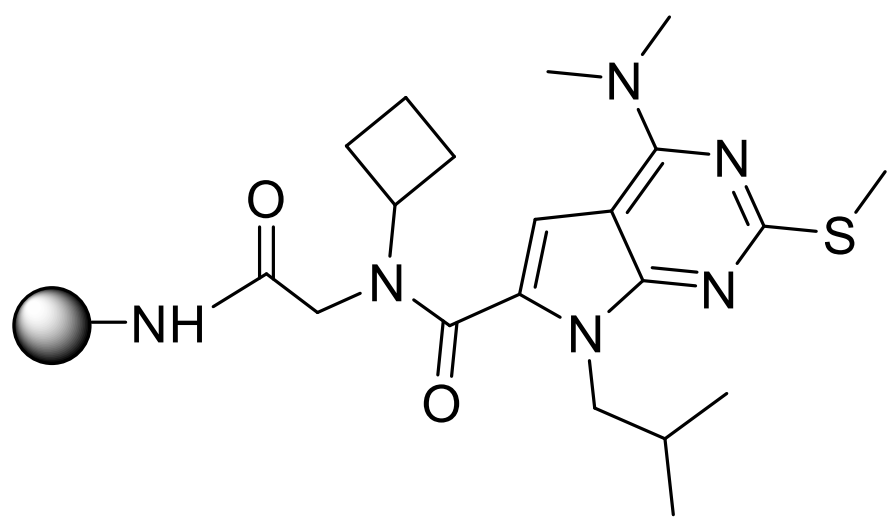

PP-v (1 g, up to 0.8 mmol) was treated with a solution of 1,8-diazabicyclo[5.4.0]undec-7-ene (DBU; 2.38 mL, 16 mmol, 20 eq) in methanol and DMF (1:2, 12 mL) and heated to 90°C for 18 h. Resin was washed with DMF (3 x 20 mL), chloroform (2 x 20 mL), methanol (2 x 20 mL), and DMF (2 x 20 mL). A test cleavage was conducted on an aliquot of resin and analysed by LC-MS. <u>LC-MS</u> (5–95% MeCN, 1 min, ROAR system) Rt = 2.83 min; m/z 419.2 ($[M+H]^+$).

Rink amide-loaded *N*-cyclobutyl-*N*-(4-(dimethylamino)-7-isobutyl-2-(methylsulfonyl)-7*H*-pyrrolo[2,3-d]pyrimidine-6-carbonyl)glycine **(PP-vii)**

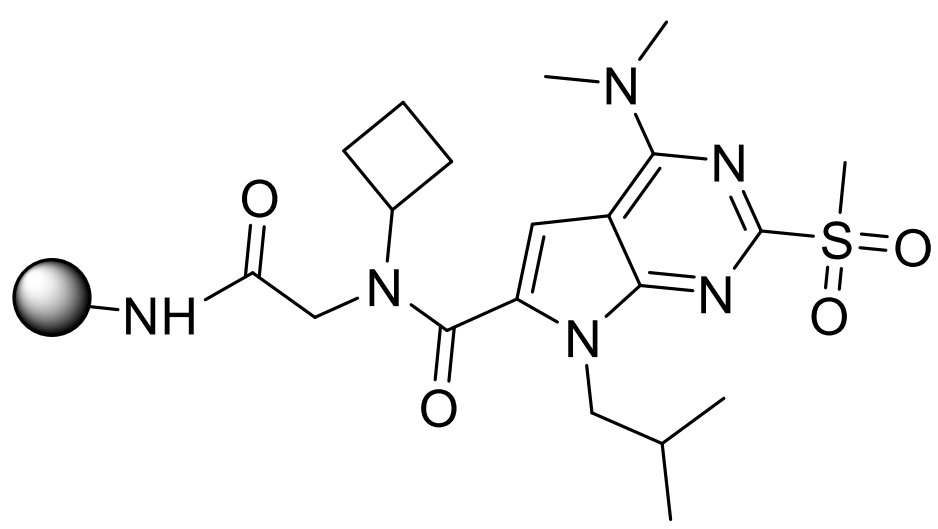

To 1 g of **PP-vi** (up to 0.8 mmol), a solution of meta-chloroperoxybenzoic acid (1.38 g, 8 mmol, 10 eq) in DMF (10 mL) and a solution of sodium hydrogen carbonate (1.00 g, 12 mmol, 15 eq) in water (2 mL) were added. The reaction was agitated at rt for 18 h before washing the resin with water (3 x 20 mL), DMF (3 x 20 mL), chloroform (2 x 20 mL), methanol (2 x 20 mL), and DMF (2 x 20 mL). A test cleavage was conducted on an aliquot of resin and analysed by LC-MS. <u>LC-MS</u> (4.5–90% MeCN, Agilent system) Rt = 4.12 min; m/z 451.10 ($[M+H]^+$).

Rink amide-loaded *N*-cyclobutyl-*N*-(4-(dimethylamino)-7-isobutyl-2-((naphthalen-1-ylmethyl)amino)-7*H*-pyrrolo[2,3-d]pyrimidine-6-carbonyl)glycine **(PP-viii)**

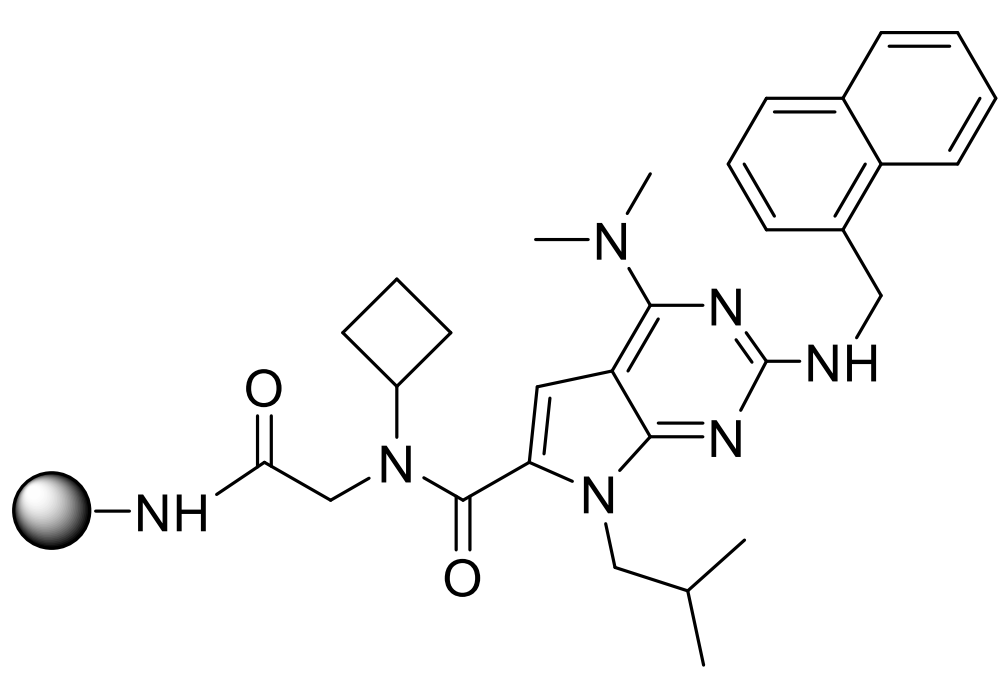

PP-vii (1 g, up to .0.8 mmol), dried under vacuum, was treated with 1-napthylmethylamine (7 mL) under anhydrous conditions. The reaction was heated to 170°C for 18 h. The resin was washed with DMF (3 x 20 mL), chloroform (2 x 20 mL), methanol (2 x 20 mL), and DMF (2 x 20 mL). A test cleavage was conducted on an aliquot of resin and analysed by LC-MS. <u>LC-MS</u> (4.5–90% MeCN, Agilent system) Rt = 6.10 min; m/z 528.30 ($[M+H]^+$).

N-(2-amino-2-oxoethyl)-*N*-cyclobutyl-4-(dimethylamino)-7-isobutyl-2-((naphthalen-1-ylmethyl)amino)-7*H*-pyrrolo[2,3-d]pyrimidine-6-carboxamide **(PP)**

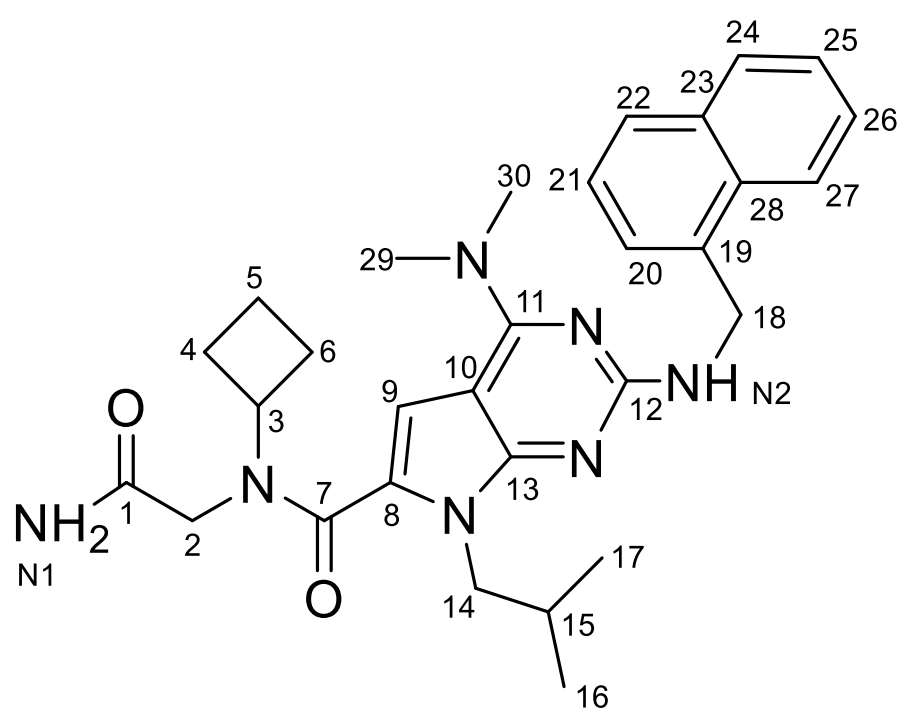

PP was obtained by cleavage off Rink amide resin by treatment of **PP-viii** (1 g, up to 0.8 mmol) with 95% TFA in DCM (5 mL) for 3 h at rt. The cleavage solution was collected and diluted with DCM before drying under a stream of air. The crude product was purified by HPLC. Two rounds of purification were required and were carried out using the Agilent 1290 Infinity II HPLC system. In the first round of purification, a gradient of MeCN and water, both containing 0.1% formic acid, was used for elution. In the second purification, a gradient of MeCN and ammonium formate (20 mM) in water was applied. **PP** was isolated as a white solid (12 mg, 3% across 8 steps, 99% purity). ^{1}H NMR (400 MHz, $CDCl_3$) δ 8.23 – 8.16 (m, 1H, H_{Ar}), 7.91 – 7.83 (m, 1H, H_{Ar}), 7.78 (d, *J* = 8.2 Hz, 1H, H_{Ar}), 7.55 – 7.46 (m, 3H, H_{Ar}), 7.41 (dd, *J* = 8.2, 7.0 Hz, 1H, H_{Ar}), 6.91 (s, 1H, H_N), 6.66 (s, 1H, H_9), 5.45 (s, 1H, H_N), 5.11 (d, *J* = 4.2 Hz, 2H, H_{18}), 4.84 (p, *J* = 8.8 Hz, 1H, H_3), 4.21 – 4.14 (m, 4H, H_2, H_{14}), 3.28 (s, 6H, H_{29}, H_{30}), 2.34 (qd, *J* = 9.9, 2.8 Hz, 2H, H_4), 2.13 (qt, *J* = 7.7, 2.3 Hz, 2H, H_6), 1.97 (hept, *J* = 6.8 Hz, 1H, H_{15}), 1.74 (d, *J* = 10.8 Hz, 2H, H_5, H_N), 1.66 – 1.49 (m, 1H, H_5), 0.80 (d, *J* = 6.7 Hz, 6H, H_{16}, H_{17}). ^{13}C NMR (101 MHz, $CDCl_3$) δ 173.2 (C_1), 166.0 (C_7), 158.7 (C_{11}), 155.7 (C_{13}), 134.0 (C_{Ar}), 131.9 (C_{Ar}), 128.8 (C_{Ar}), 128.0 (C_{Ar}), 126.3 (C_{Ar}), 126.2 (C_{Ar}), 125.8 (C_{Ar}), 125.6 (C_{Ar}), 124.1 (C_{Ar}), 123.6 (C_8), 107.0 (C_9), 96.4 (C_{10}), 54.3 (C_3), 49.4 (C_{14}), 47.6 (C_2), 43.8 (C_{18}), 39.2 (C_{29}, C_{30}), 29.7 (C_{15}), 29.2 (C_4, C_6), 20.1 (C_{16}, C_{17}), 14.6 (C_5). LC-MS (4.5–90% MeCN, Agilent system) Rt = 4.95 min; m/z 528.30 ($[M+H]^+$). HRMS (m/z) calcd. for $[M+H]^+$ $C_{30}H_{38}N_7O_2$: 528.3087; found: 528.3081

PP-1

Rink amide-loaded *N*-cyclobutyl-*N*-(*N*-(5-formyl-6-(methylamino)-2-(methylthio)pyrimidin-4-yl)-*N*-isobutylglycyl)glycine **(PP1-vi)**

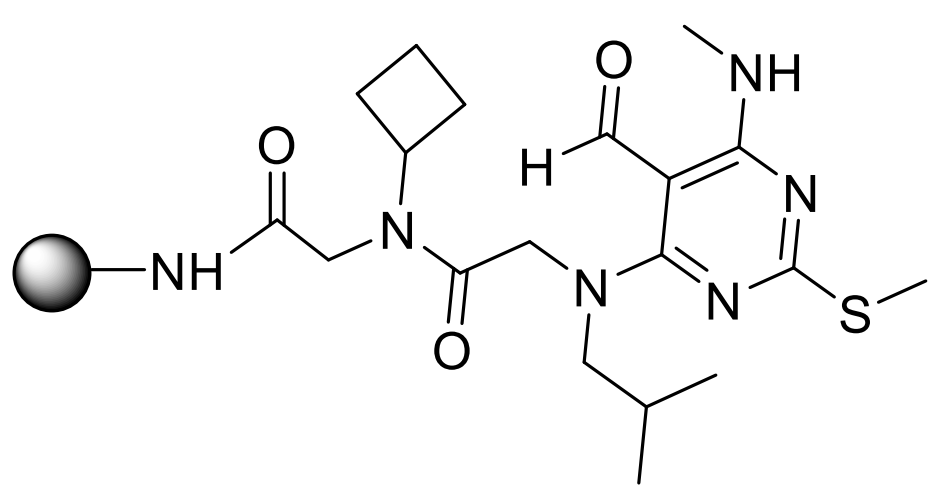

To 700 mg of **PP-v** (up to 0.56 mmol), a solution of *N*-methylamine (56 mmol, 100 eq) and triethylamine (7.8 mL, 56 mmol, 100 eq) in THF (28 mL) was added. The reaction was heated to 65°C for 18 h. Resin was then washed with DMF (3 x 20 mL), DCM (2 x 20 mL), methanol (2 x 20 mL), and DMF (2 x 20 mL). A test cleavage was conducted on an aliquot of resin and analysed by LC-MS. LC-MS (5–95% MeCN, 2 min, Agilent system B) Rt = 3.15 min; m/z 423.3 ($[M+H]^+$).

Rink amide-loaded *N*-cyclobutyl-*N*-(7-isobutyl-4-(methylamino)-2-(methylthio)-7*H*-pyrrolo[2,3-d]pyrimidine-6-carbonyl)glycine **(PP1-vii)**

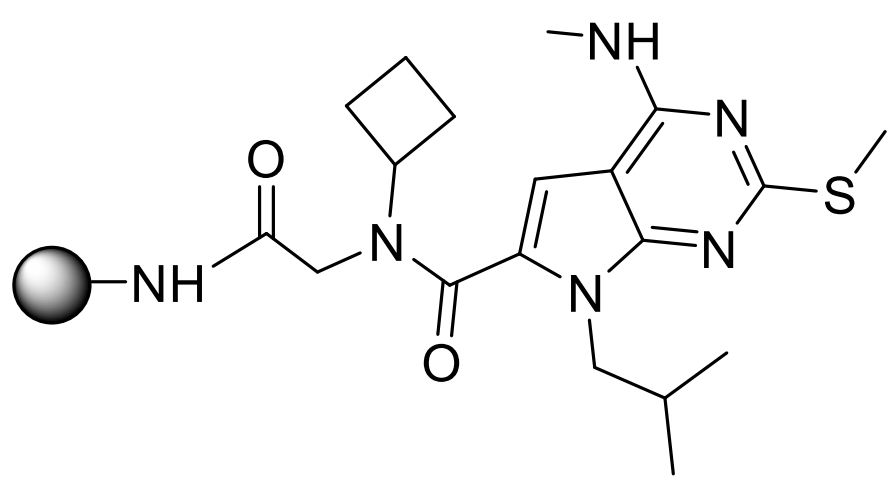

PP1-vi (700 mg, up to 0.56 mmol) was treated with a solution of DBU (8.4 mL, 56 mmol, 100 eq) in methanol and DMF (1:2, 15 mL) and heated to 90°C for 18 h. Resin was washed with DMF (3 x 20 mL), chloroform (2 x 20 mL), methanol (2 x 20 mL), and DMF (2 x 20 mL). A test cleavage was conducted on an aliquot of resin and analysed by LC-MS. LC-MS (5–95% MeCN, 2 min, Agilent system B) Rt = 3.01 min; m/z 405.3 ([M+H]$^+$).

Rink amide-loaded *N*-cyclobutyl-*N*-(7-isobutyl-4-(methylamino)-2-(methylsulfonyl)-7*H*-pyrrolo[2,3-d]pyrimidine-6-carbonyl)glycine **(PP1-viii)**

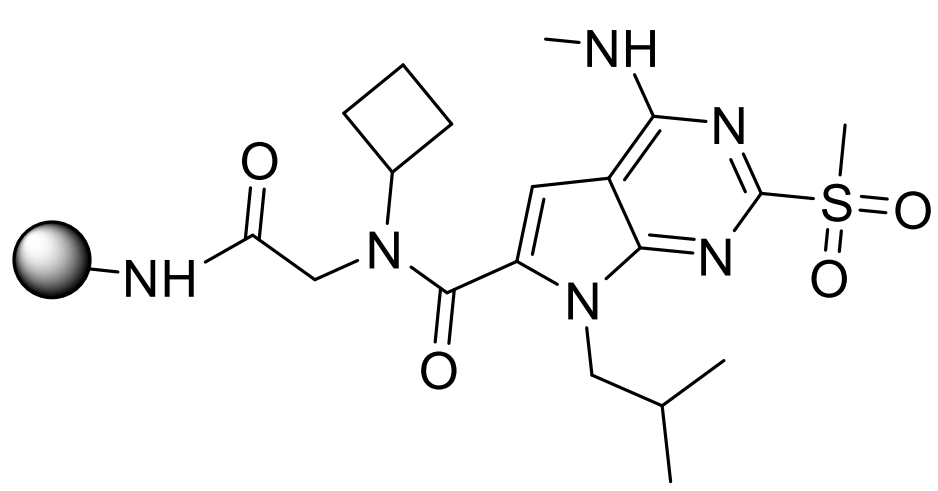

Reaction was conducted as described for **PP-vii** and analysed by LC-MS. LC-MS (5–95% MeCN, 2 min, Agilent system B) Rt = 2.84 min; m/z 437.2 ([M+H]$^+$).

Rink amide-loaded *N*-cyclobutyl-*N*-(7-isobutyl-4-(methylamino)-2-((naphthalen-1-ylmethyl)amino)-7*H*-pyrrolo[2,3-d]pyrimidine-6-carbonyl)glycine **(PP1-ix)**

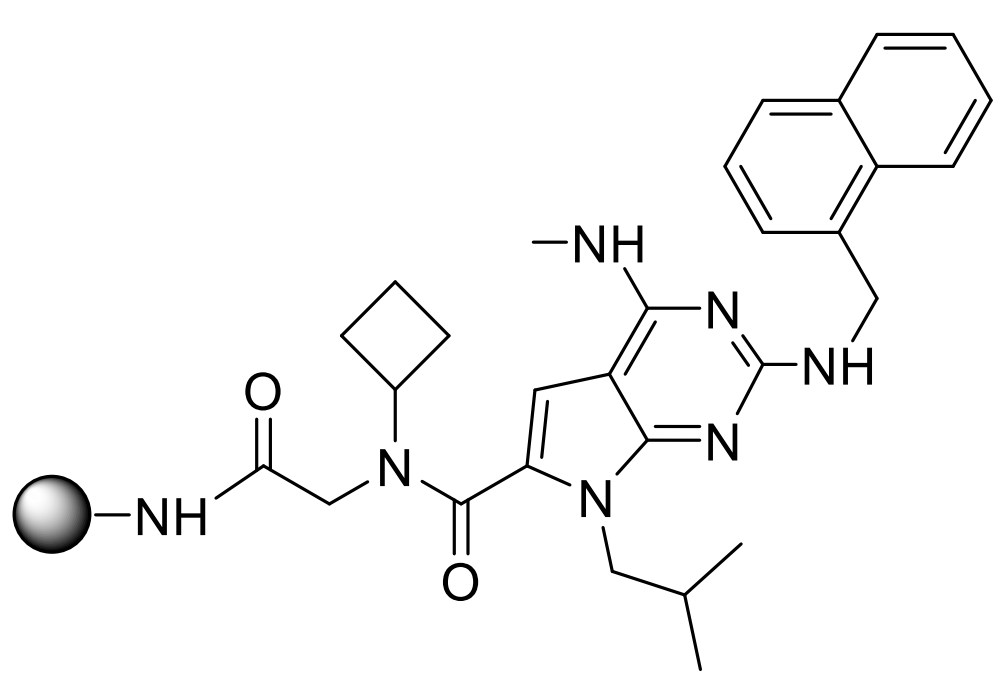

Reaction was conducted as described for **PP-viii** and analysed by LC-MS. LC-MS (35–65% MeCN, 2 min, Agilent system B) Rt = 1.24 min; m/z 514.3 ([M+H]$^+$).

Rink amide-loaded *N*-(2-amino-2-oxoethyl)-*N*-cyclobutyl-7-isobutyl-4-(methylamino)-2-((naphthalen-1-ylmethyl)amino)-7*H*-pyrrolo[2,3-d]pyrimidine-6-carboxamide **(PP1-x)**

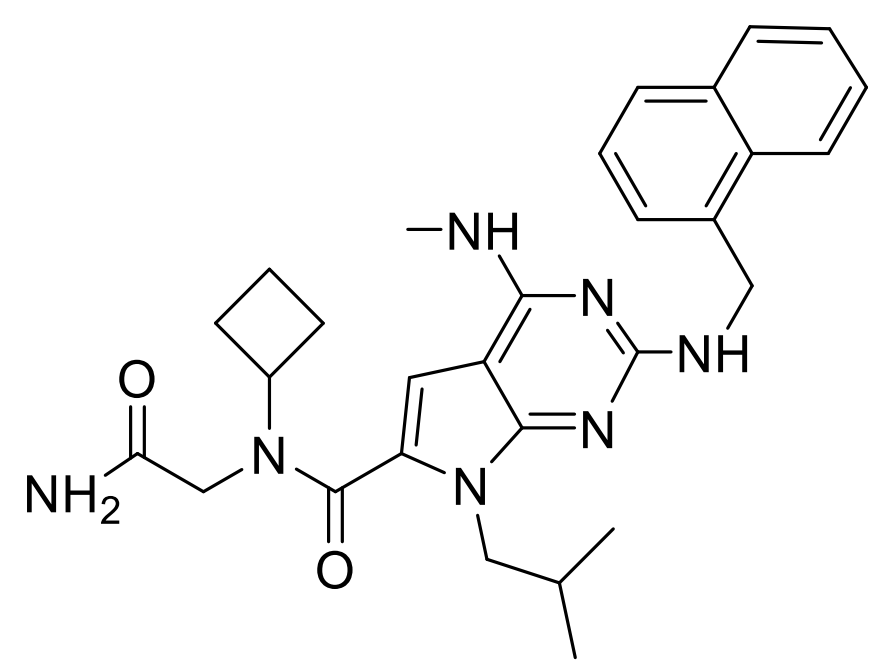

PP1-x was obtained by cleavage off Rink amide resin by treatment of **PP1-ix** (300 mg, up to 0.24 mmol) with 95% TFA in DCM (5 mL) for 3 h at rt. The cleavage solution was collected and diluted with DCM before drying under a stream of air. The crude product was purified by HPLC to yield **PP1-x** as a white solid (24.5 mg, 20% across 9 steps, >95% pure by LC-MS). LC-MS (4.5–90% MeCN, Agilent system A) Rt = 5.64 min; m/z 514.20 ($[M+H]^+$).

N-(2-amino-2-oxoethyl)-4-(3-(3-(but-3-yn-1-yl)-3*H*-diazirin-3-yl)-*N*-methylpropanamido)-*N*-cyclobutyl-7-isobutyl-2-((naphthalen-1-ylmethyl)amino)-7*H*-pyrrolo[2,3-d]pyrimidine-6-carboxamide **(PP-1)**

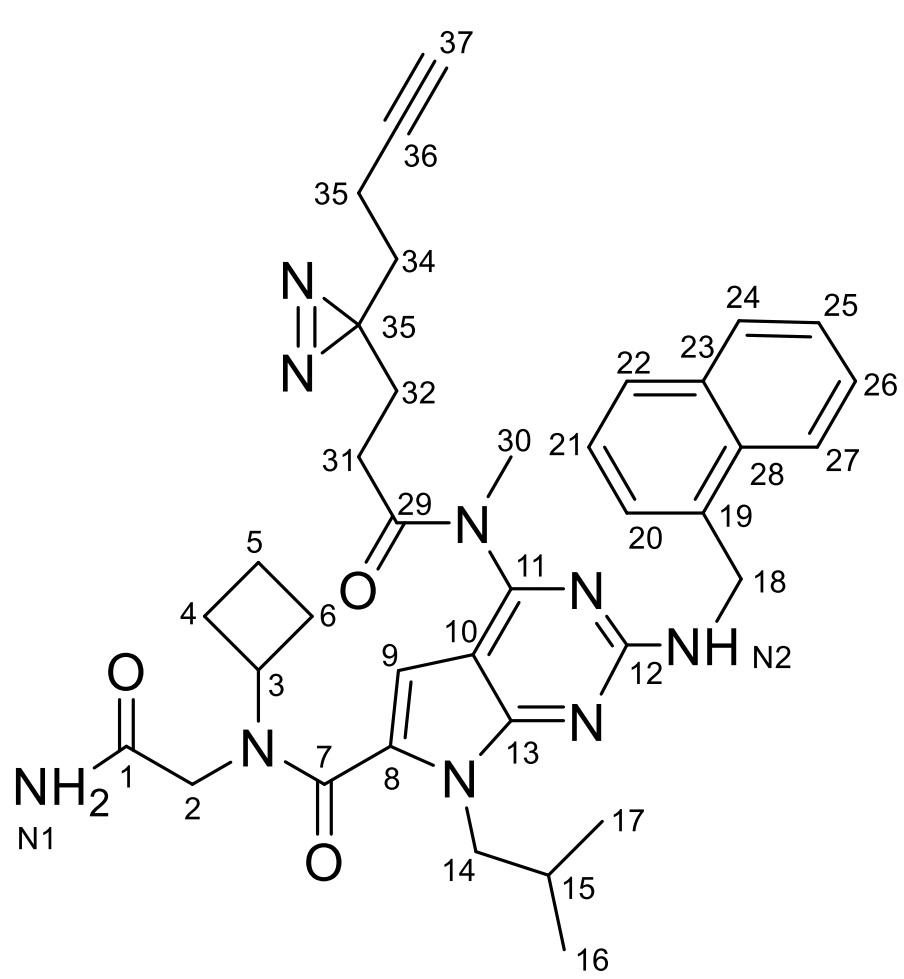

To a solution of **PP1-x** (20 mg, 0.39 mmol) in DMF (0.4 mL), DIPEA (84 µL, 0.48 mmol, 12 eq), HATU (59 mg, 0.16 mmol, 4 eq), and 3-(3-(but-3-yn-1-yl)-3*H*-diazirin-3-yl)propanoic acid (13 mg, 0.078 mmol, 2 eq) were added. The mixture was stirred in the dark at 50°C for 18 h, after which the reaction was quenched by addition of water (10 mL). The mixture was dried by lyophilisation. The crude product was purified by HPLC, to yield **PP-1** as a white solid (4.8 mg, 19% after two rounds of purification by HPLC, 97% purity). ^{1}H NMR (400 MHz, $CDCl_3$) δ 8.13 (d, *J* = 8.3 Hz, 1H, H_{Ar}), 7.88 (dd, *J* = 7.9, 1.6 Hz, 1H, H_{Ar}), 7.79 (d, *J* = 8.2 Hz, 1H, H_{Ar}), 7.55 (dqd, *J* = 14.9, 6.9, 3.6 Hz, 3H, H_{Ar}), 7.42 (dd, *J* = 8.2, 7.1 Hz, 1H, H_{Ar}), 6.52 (d, *J* = 17.1 Hz, 2H, H_{Ar}, H_N), 6.13 (s, 1H, H_N), 5.14 (s, 2H, H_{18}), 4.70 (q, *J* = 8.6 Hz, 1H, H_3), 4.20 (d, *J* = 9.4 Hz, 4H, H_2, H_{14}), 3.38 (s, 3H, H_{30}), 2.33 – 2.11 (m, 6H, H_4, H_6, H_{31}), 2.06 – 1.92 (m, 4H, H_{15}, H_{35}, H_{37}), 1.83 (s, 2H, H_{32}), 1.73 (t, *J* = 9.6 Hz, 1H, H_5), 1.60 (dt, *J* = 14.2, 8.1 Hz, 3H, H_5, H_{34}), 0.84 (d, *J* = 6.6 Hz, 6H, H_{16}, H_{17}). ^{13}C NMR (101 MHz, $CDCl_3$) δ 172.7 (C_{29}), 172.2 (C_1), 169.4 (C_7), 163.8 (C_{Ar}), 155.4 (C_{Ar}), 144.7 (C_{Ar}), 134.0 (C_{Ar}), 133.5 (C_{Ar}), 131.8 (C_{Ar}), 131.4 (C_{Ar}), 129.0 (C_{Ar}), 128.4 (C_{Ar}), 126.6 (C_{Ar}), 126.1 (C_{Ar}), 125.5 (C_{Ar}), 123.3 (C_{Ar}), 117.2 (C_{Ar}), 114.3 (C_{Ar}), 103.6 (C_{Ar}), 82.8 (C_{36}), 69.4 (C_{37}), 68.8 (C_{35}), 54.0 (C_3), 50.6 (C_{14}), 46.6 (C_2), 43.6 (C_{14}), 39.8 (C_{30}), 32.6 (C_{34}), 29.4 (C_{31}), 29.2 (C_{15}), 27.9 (C_4, C_6), 27.7 (C_{32}), 19.8 (C_{16}, C_{17}), 14.5 (C_5), 13.4 (C_{35}). LC-MS (4.5–90% MeCN, Agilent system A) Rt = 6.20 min; m/z 662.30 ($[M+H]^+$). HRMS (m/z) calcd. for $[M+H]^+$ $C_{37}H_{44}N_9O_3$: 662.3567, found: 662.3564

PP-2

Rink amide-loaded 2-amino-5-(tert-butoxy)-5-oxopentanoic acid **(PP2-i)**

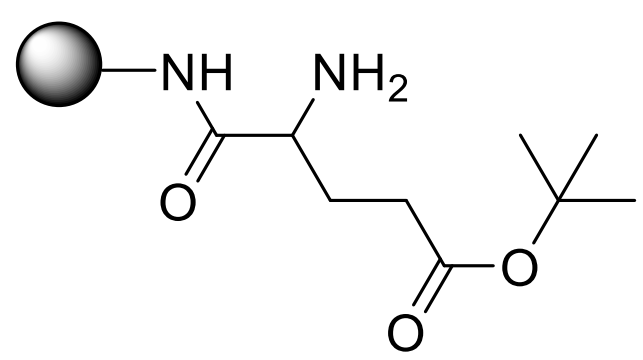

A solution of 9-fluorenylmethyloxycarbonyl (Fmoc)-L-glutamic acid 5-*tert*-butyl ester (1.02 g, 2.4 mmol, 3 eq), DIPEA (697 µL, 4 mmol, 5 eq), and hexafluorophosphate benzotriazole tetramethyl uranium (HBTU, 910 mg, 2.4 mmol, 3 eq) was added to deprotected Rink amide resin (1 g). The mixture was heated to 50°C for 30 min using a microwave reactor. The reaction mixture was drained, and the reaction was repeated to maximise loading. Resin was washed with DMF (3 x 20 mL), chloroform (2 x 20 mL), methanol (2 x 20 mL), and DMF (2 x 20 mL).

Rink amide-loaded 5-(tert-butoxy)-2-(2-(*N*-cyclobutyl-2-(isobutylamino)acetamido)acetamido)-5-oxopentanoic acid **(PP2-v)**

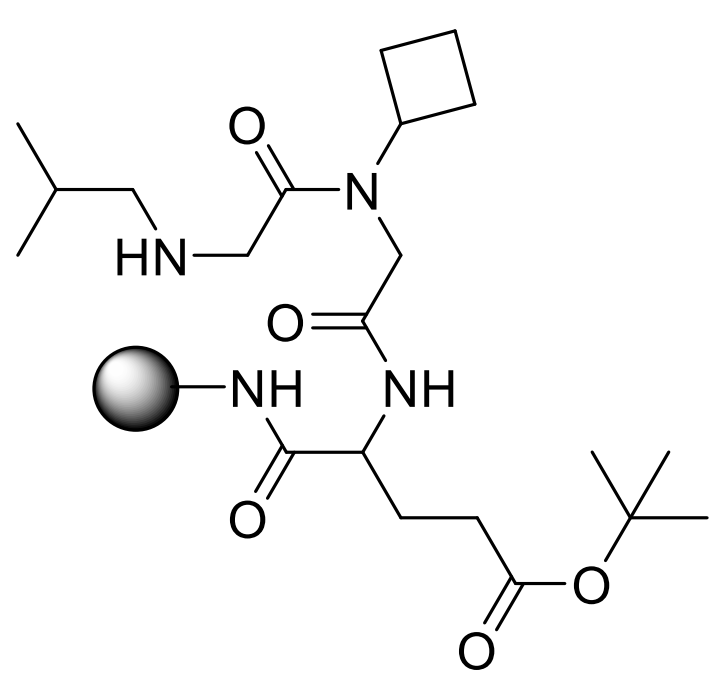

Steps for synthesis of **PP–iv** were followed using **PP2-i**. A test cleavage was conducted on an aliquot of resin and analysed by LC-MS. LC-MS (5–95% MeCN, in-lab system) Rt = 5.41 min; m/z 371.2 ($[M+H]^+$ for *t*Bu-deprotected).

Rink amide-loaded 5-(tert-butoxy)-2-(2-(2-((6-chloro-5-formyl-2-(methylthio)pyrimidin-4-yl)(isobutyl)amino)-*N*-cyclobutylacetamido)acetamido)-5-oxopentanoic acid **(PP2-vi)**

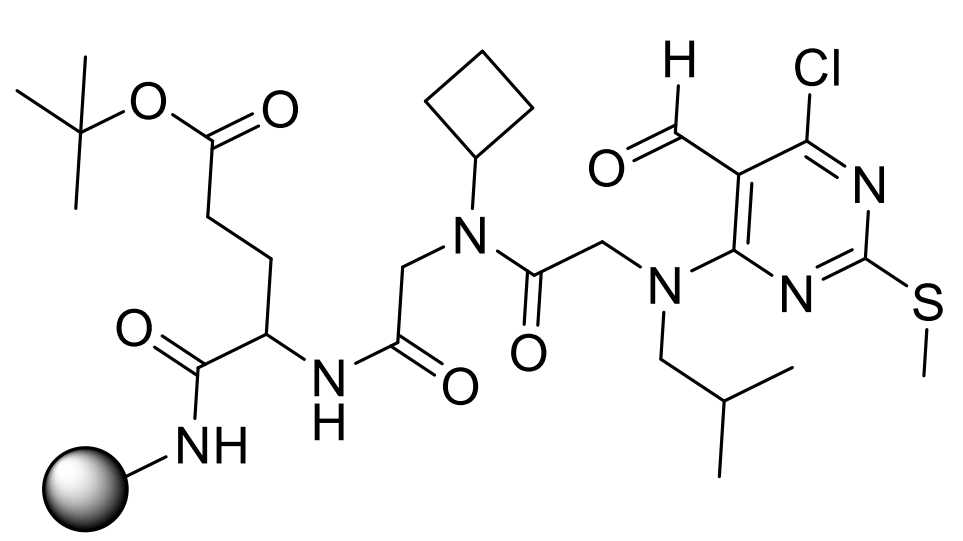

S_NAr was carried out as described for **PP-v**. A test cleavage was conducted on an aliquot of resin and analysed by LC-MS. LC-MS (4.5–90% MeCN, Agilent system) Rt = 3.59 min; m/z 556.90 ($[M+H]^+$ for *t*Bu-deprotected).

Rink amide-loaded *N*-cyclobutyl-*N*-(*N*-(6-(dimethylamino)-5-formyl-2-(methylthio)pyrimidin-4-yl)-*N*-isobutylglycyl)glycylglutamic acid **(PP2-vii)**

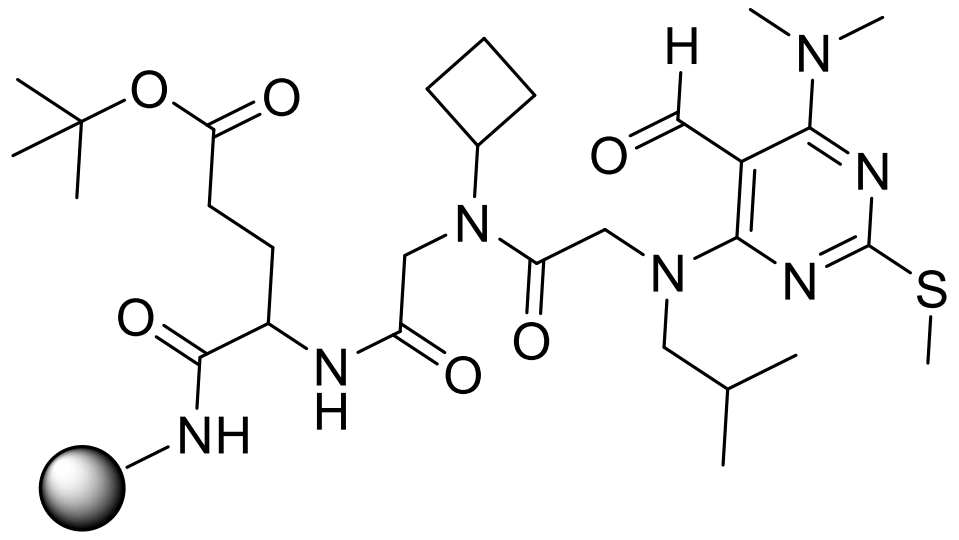

To 1 g of **PP2-vi** (up to 0.8 mmol), a solution of *N,N*-dimethylamine (40 mmol, 50 eq) in THF (20 mL) was added. The reaction was heated to 65°C for 18 h. Resin was then washed with DMF (3 x 20 mL), DCM (2 x 20 mL), methanol (2 x 20 mL), and DMF (2 x 20 mL). A test cleavage was conducted on an aliquot of resin and analysed by LC-MS. LC-MS (4.5–90% MeCN, Agilent system) Rt = 4.50 min; m/z 566.00 ($[M+H]^+$ for *t*Bu-deprotected).

Rink amide-loaded 5-(tert-butoxy)-2-(2-(*N*-cyclobutyl-4-(dimethylamino)-7-isobutyl-2-(methylthio)-7*H*-pyrrolo[2,3-d]pyrimidine-6-carboxamido)acetamido)-5-oxopentanoic acid **(PP2-viii)**

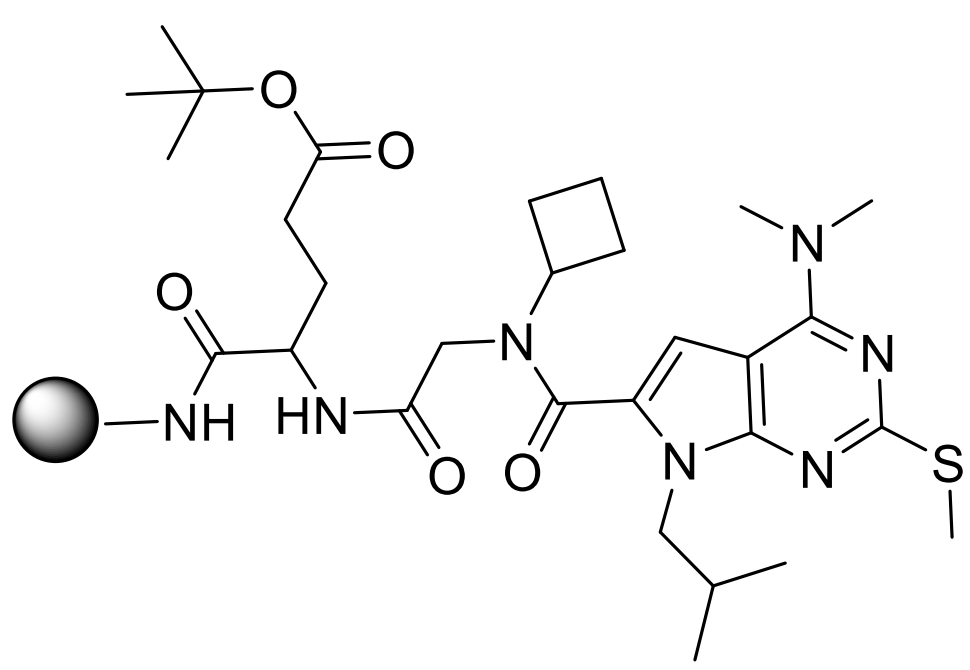

Reaction was conducted as described for **PP1-vii** and analysed by LC-MS. LC-MS (4.5–90% MeCN, Agilent system) Rt = 3.98 min; m/z 548.00 ($[M+H]^+$ for *t*Bu-deprotected).

Rink amide-loaded 5-(tert-butoxy)-2-(2-(*N*-cyclobutyl-4-(dimethylamino)-7-isobutyl-2-(methylsulfonyl)-7*H*-pyrrolo[2,3-d]pyrimidine-6-carboxamido)acetamido)-5-oxopentanoic acid **(PP2-ix)**

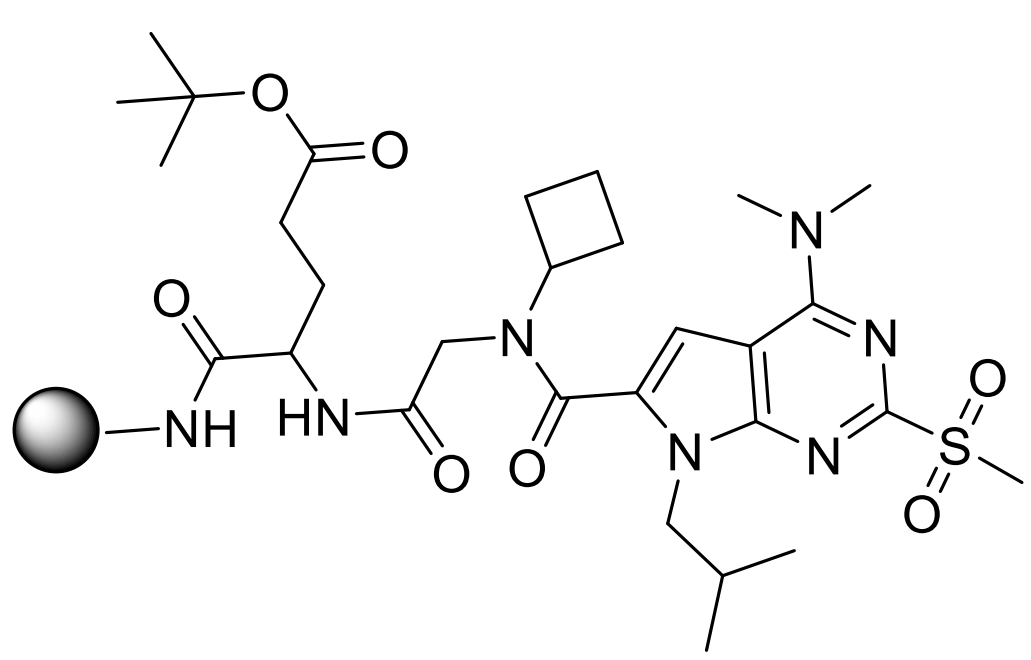

Reaction was conducted as described for **PP-vii** and analysed by LC-MS. LC-MS (5–95% MeCN, in-lab system) Rt = 3.48 min; m/z 580.3 ($[M+H]^+$ for *t*Bu-deprotected).

Rink amide-loaded 5-(tert-butoxy)-2-(2-(*N*-cyclobutyl-4-(dimethylamino)-7-isobutyl-2-((naphthalen-1-ylmethyl)amino)-7*H*-pyrrolo[2,3-d]pyrimidine-6-carboxamido)acetamido)-5-oxopentanoic acid **(PP2-x)**

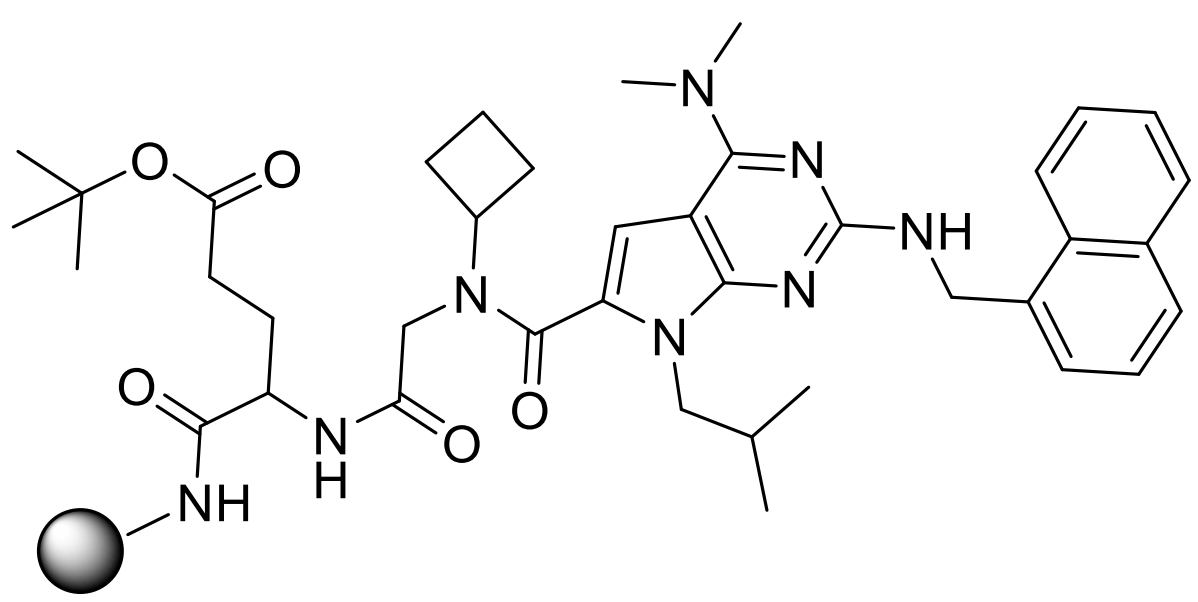

Reaction was conducted as described for **PP-viii**, but at 140°C. Test cleavage was conducted and analysed by LC-MS. LC-MS (4.5–90% MeCN, Agilent system, basic method) Rt = 4.83 min; m/z 657.30 ($[M+H]^+$ for *t*Bu-deprotected).

5-amino-4-(2-(*N*-cyclobutyl-4-(dimethylamino)-7-isobutyl-2-((naphthalen-1-ylmethyl)amino)-7*H*-pyrrolo[2,3-d]pyrimidine-6-carboxamido)acetamido)-5-oxopentanoic acid **(PP2-xi)**

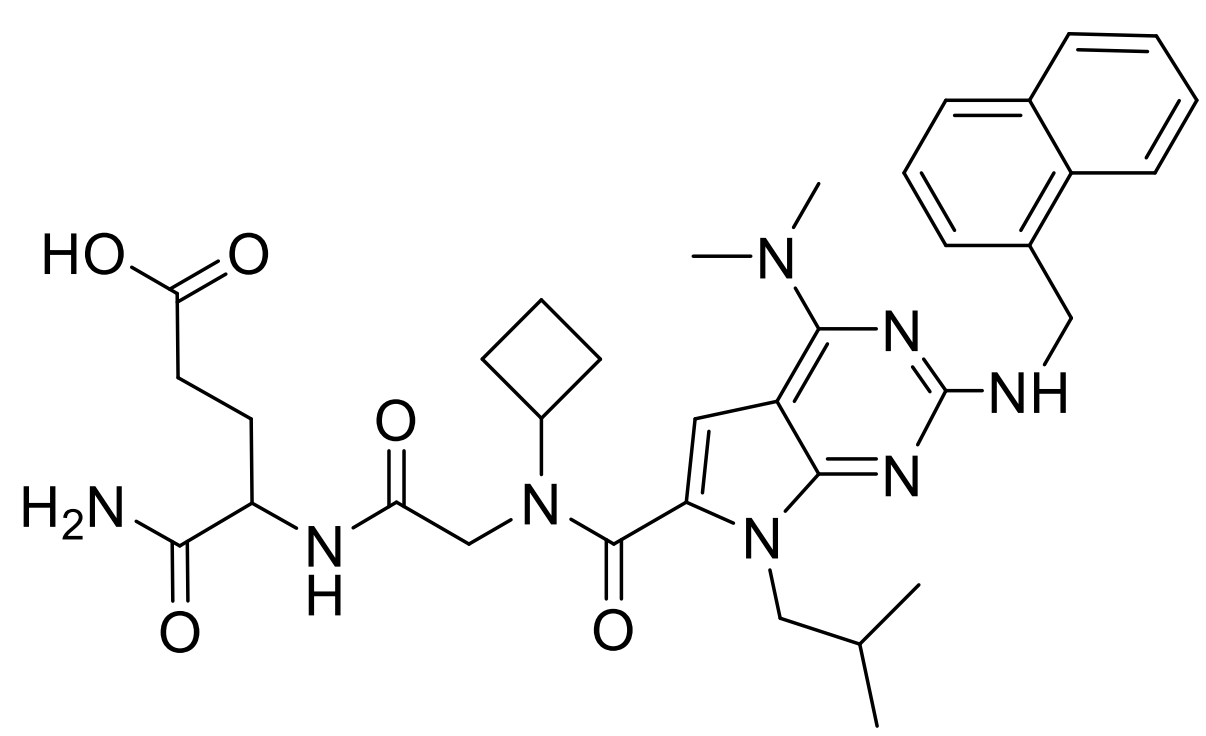

PP2-xi was obtained by cleavage off Rink amide resin by treatment of **PP2-x** (1 g, up to 0.8 mmol) with 95% TFA in water (5 mL) for 3 h at rt. The cleavage solution was collected and diluted with DCM before drying under a stream of air. The crude product was purified by HPLC to yield **PP2-x** as a white solid (12 mg, 2% across 10 steps, > 83% pure by LC-MS). LC-MS (4.5–90% MeCN, Agilent system, basic method) Rt = 4.02 min; m/z 657.00 ($[M+H]^+$).

4-(2-(*N*-cyclobutyl-4-(dimethylamino)-7-isobutyl-2-((naphthalen-1-ylmethyl)amino)-7*H*-pyrrolo[2,3-d]pyrimidine-6-carboxamido)acetamido)-*N*[1]-((3-ethyl-3*H*-diazirin-3-yl)methyl)-*N*[1]-(prop-2-yn-1-yl)pentanediamide **(PP-2)**

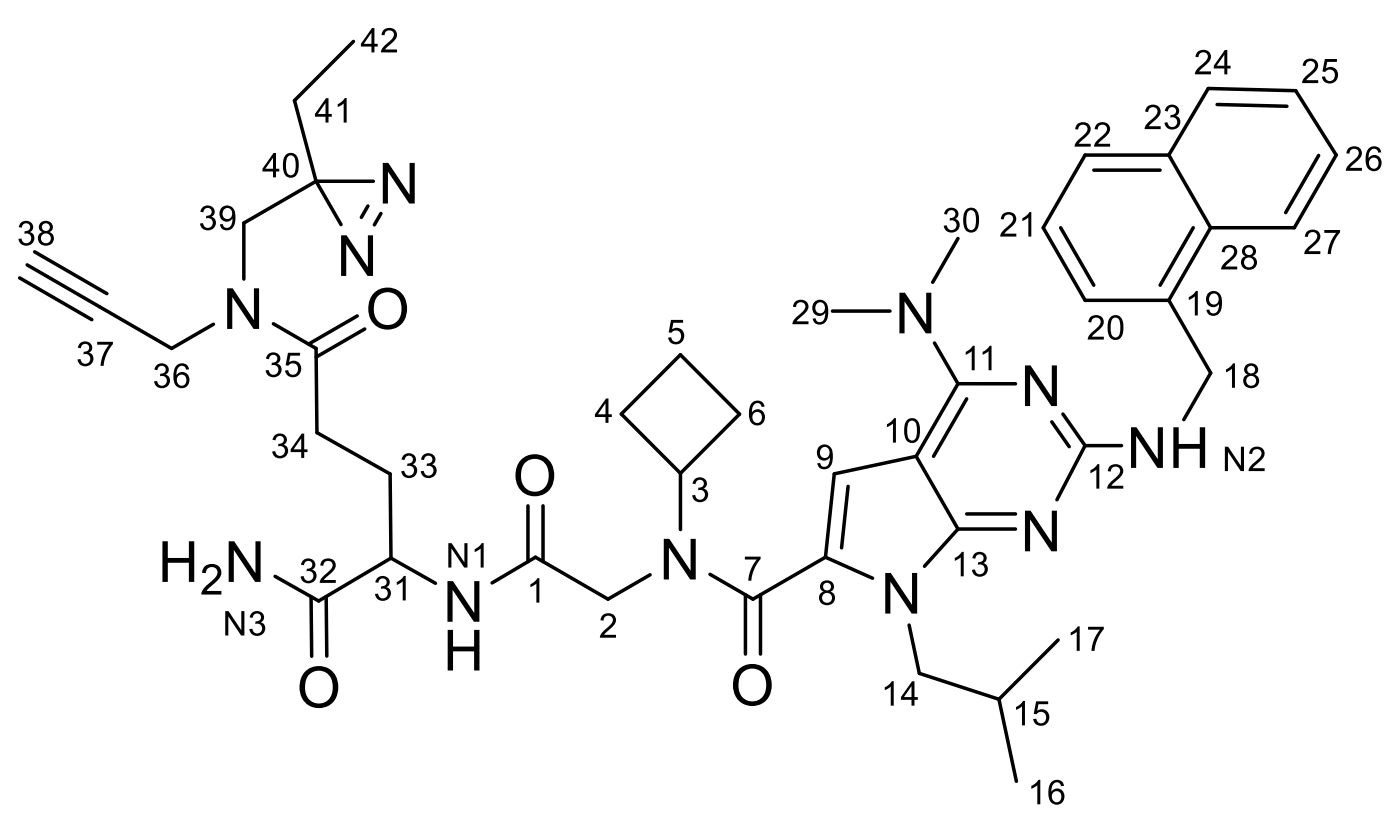

To a solution of **PP2-xi** (12 mg, 0.018 mmol) in DMF (1.5 mL), HATU (20.9 mg, 0.055 mmol, 3 eq) and DIPEA (7.9 µL, 0.046 mmol, 2.5 eq) were added. After 15 minutes, a solution of *N*-(2-(3-methyl-3*H*-diazirin-3-yl)ethyl)prop-2-yn-1-aminium chloride (9.5 mg, 0.055 mmol) and DIPEA (7.9 µL, 0.046 mmol) in DMF (0.5 mL) was added to the reaction mixture. The mixture was stirred in the dark for 2 h, until complete conversion to the amide was observed by LC-MS. The reaction was quenched by the addition of water (20 mL) and lyophilised to remove solvent. The product was purified by HPLC, to yield **PP-2** as a white solid (6.7 mg, 48%, 98% purity). ^{1}H NMR (400 MHz, DMSO-d_6, 373 K) δ 8.28 (d, *J* = 7.2 Hz, 1H,

H_{Ar}), 7.93 (dd, *J* = 8.3, 1.5 Hz, 1H, H_{Ar}), 7.81 (d, *J* = 8.1 Hz, 1H, H_{Ar}), 7.75 (d, *J* = 7.6 Hz, 1H, H_{N1}), 7.61 – 7.49 (m, 3H, H_{Ar}), 7.48 – 7.40 (m, 1H, H_{Ar}), 6.90 (s, 1H, H_N), 6.74 (s, 1H, H_{Ar}), 5.03 (s, 3H, H_{18}), 4.64 (q, *J* = 8.6 Hz, 1H, H_3), 4.32 – 4.25 (m, 1H, H_{31}), 4.17 (d, *J* = 3.8 Hz, 2H, H_{14}), 4.11 (d, *J* = 2.4 Hz, 2H, H_{36}), 3.99 (d, *J* = 7.2 Hz, 2H, H_{14}), 3.38 – 3.30 (m, 3H, H_{38}, H_{39}), 3.26 (s, 6H, H_{29}, H_{30}), 2.41 (t, *J* = 7.7 Hz, 2H, H_{33}), 2.22 – 1.97 (m, 6H, H_4, H_6, H_{34}), 1.85 (dt, *J* = 14.4, 7.7 Hz, 1H, H_{15}), 1.59 (m, *J* = 18.5, 8.8 Hz, 4H, H_5, H_{41}), 1.02 (s, 3H, H_{42}), 0.73 (d, *J* = 6.7 Hz, 6H, H_{16}, H_{17}). LC-MS (4.5–90% MeCN, Agilent system, basic method) Rt = 5.44 min; m/z 776.00 ($[M+H]^+$). HRMS (m/z) calcd. for $[M+H]^+$ $C_{42}H_{54}N_{11}O_4$: 776.4360, found: 776.4364.

Oxopiperazine

OP was synthesised as detailed by Lao and coworkers.[3]

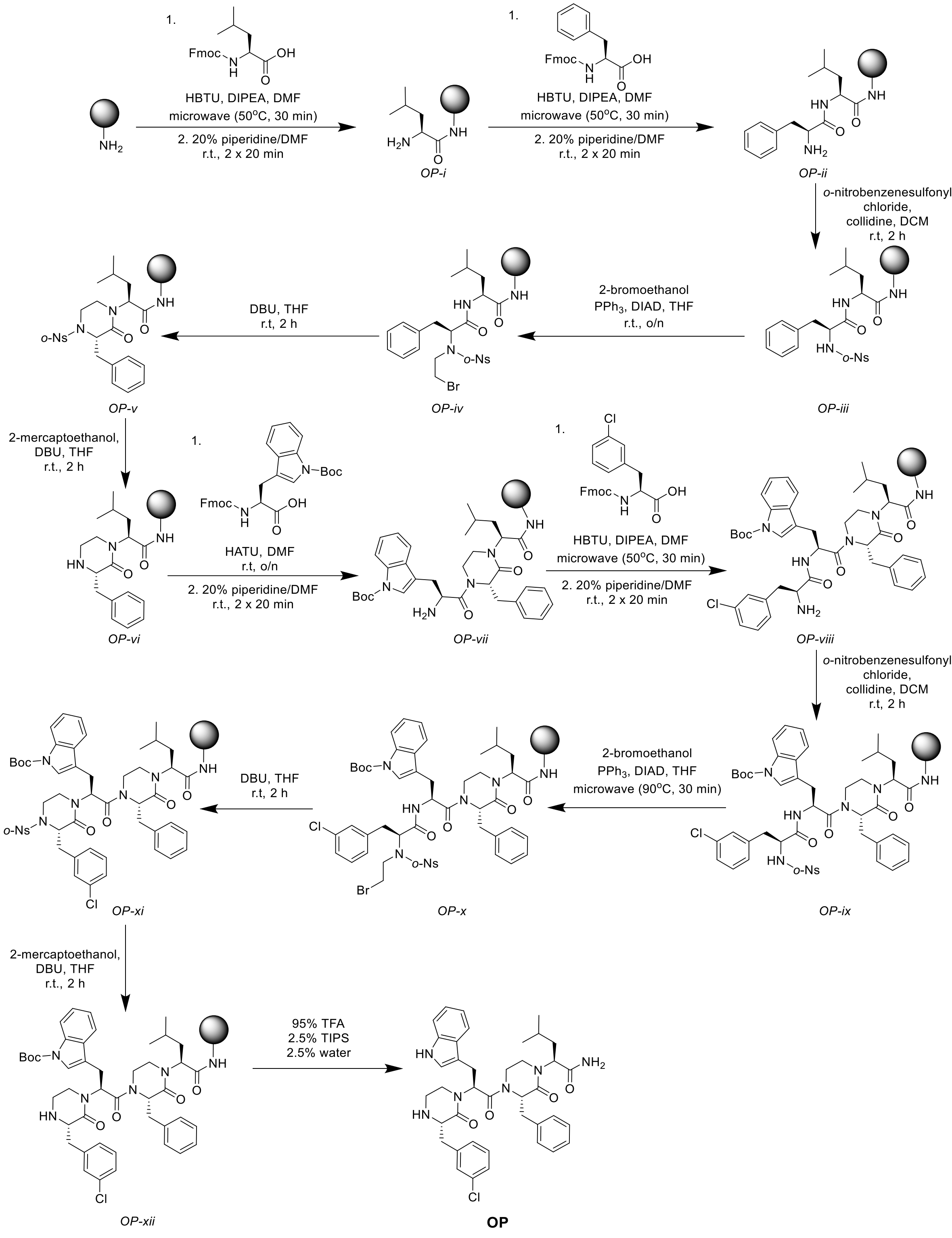

Scheme 3: Synthetic route for ***OP****.*[3] *Grey circle represents solid support.*

OP

Rink amide-loaded *L*-leucine **(OP-i)**

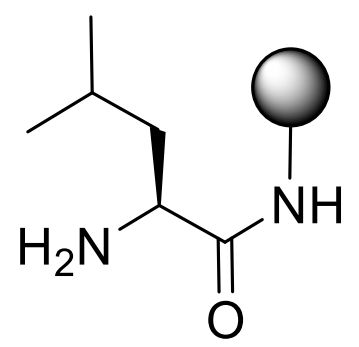

L-leucine was loaded onto deprotected Rink amide resin (2 g, up to 1.6 mmol loading) by heating the resin with a solution of Fmoc-*L*-leucine. (1.70 g, 4.8 mmol, 3 eq), HBTU (1.82 g, 4.8 mmol, 3 eq), and DIPEA (1.39 mL, 8 mmol, 5 eq) in DMF (15 mL) to 50°C in a microwave reactor for 30 min. The reaction mixture was then cooled to rt, drained, and the reaction was repeated. The resin was washed with DMF (8 x 20 mL). The loaded amino acid was deprotected by treatment of the resin with a 20% solution of piperidine in DMF (15 mL). After 20 min, the deprotection solution was drained and replaced with a fresh solution of 20% piperidine for a further 20 min. Resin was washed with DMF (8 x 20 mL).

Rink amide-loaded *L*-phenylalanyl-*L*-leucine **(OP-ii)**

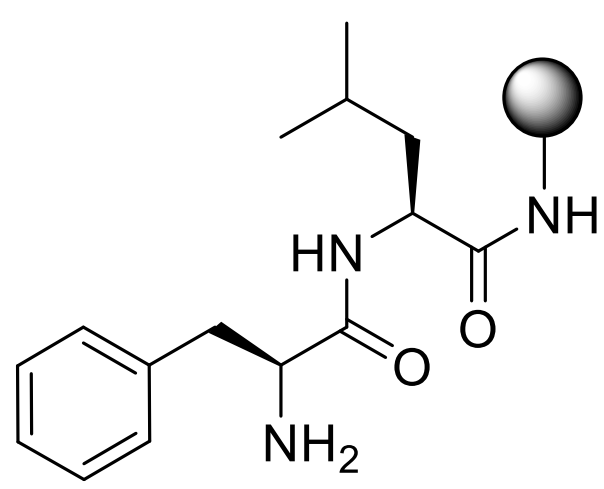

The second amino acid, Fmoc-phenylalanine, was loaded and deprotected as described for **OP-i**. A test cleavage was conducted on an aliquot of resin and analysed by LC-MS. LC-MS (4.5–90% MeCN, Agilent system) Rt = 2.12 min; m/z 278.00 ($[M+H]^+$).

Rink amide-loaded ((2-nitrophenyl)sulfonyl)-*L*-phenylalanyl-*L*-leucine **(OP-iii)**

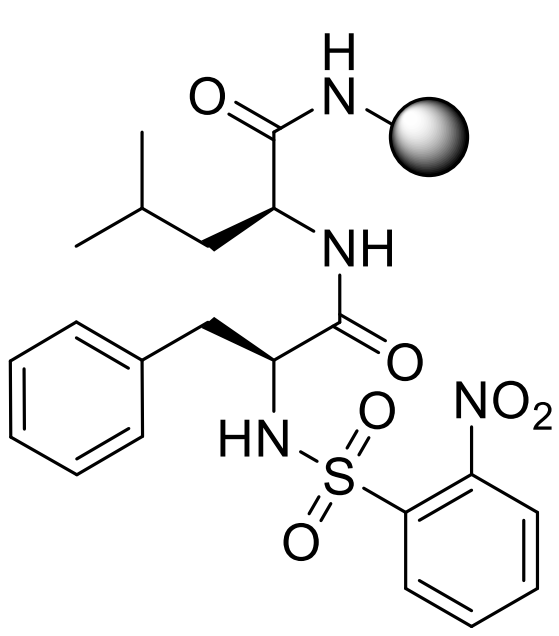

To **OP-ii** (2g, up to 1.6 mmol), a solution of *o*-nitrobenzenesulfonyl chloride (3.5 g, 16 mmol, 10 eq) and collidine (2.1 mL, 16 mmol, 10 eq) in anhydrous DCM (15 mL) was added and the mixture was agitated for 2 h at rt in a sealed reaction vessel. Resin was then washed with DMF (3 x 20 mL), DCM (3 x 20 mL), methanol (2 x 20 mL), and diethyl ether (2 x 20 mL), before drying under vacuum for 12 h. A test cleavage was conducted on an aliquot of resin and analysed by LC-MS. LC-MS (4.5–90% MeCN, Agilent system) Rt = 4.95 min; m/z 463.90 ($[M+H]^+$).

Rink amide-loaded *N*-(2-bromoethyl)-*N*-((2-nitrophenyl)sulfonyl)-*L*-phenylalanyl-*L*-leucine **(OP-iv)**

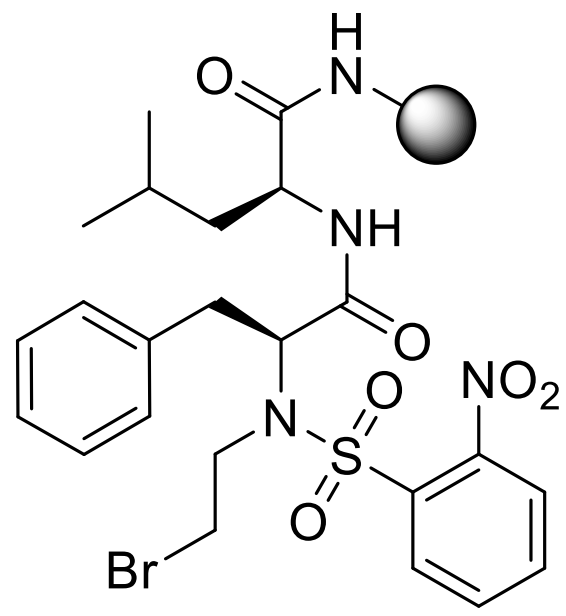

A sealed microwave vial containing triphenylphosphine (4.2 g, 16 mmol, 10 eq) and **OP-iii** (2 g, up to 1.6 mmol) was flushed with nitrogen for 30 min. A solution of 2-bromoethanol (1.1 mL, 16 mmol, 10 eq) and diisopropyl azodicarboxylate (3.1 mL, 16 mmol, 10 eq) in anhydrous THF (15 mL) was added. The reaction mixture was heated to 70°C for 30 min using a microwave reactor. The mixture was cooled to rt, drained, and the resin was washed with THF (3 x 20 mL), DMF (3 x 20 mL), and DCM (2 x 20 mL). A test cleavage was conducted on an aliquot of resin and analysed by LC-MS. LC-MS (4.5–90% MeCN, Agilent system) Rt = 5.13 min; m/z 569.80 ([M+H]$^+$).

Rink amide-loaded (*S*)-2-((*S*)-3-benzyl-4-((2-nitrophenyl)sulfonyl)-2-oxopiperazin-1-yl)-4-methylpentanoic acid **(OP-v)**

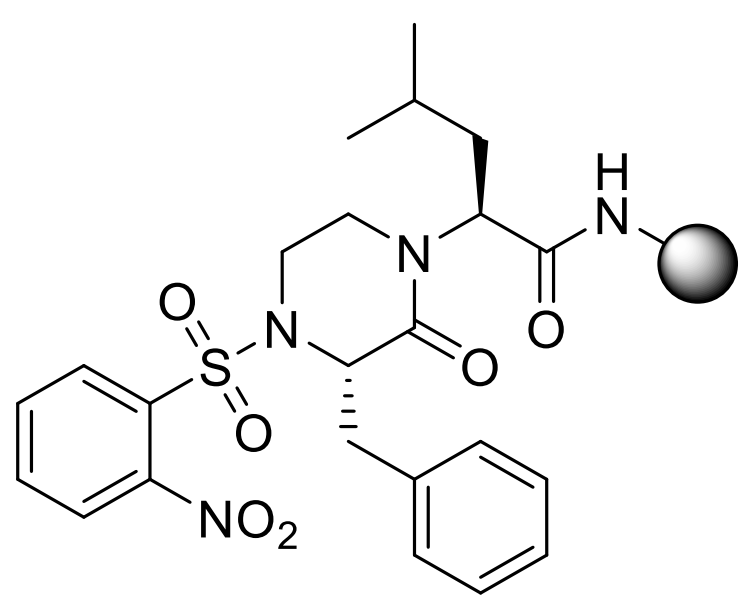

Cyclisation was carried out by treatment of **OP-iv** (2 g, up to 1.6 mmol) with DBU (4.7 mL, 32 mmol, 20 eq) in THF (10 mL). The reaction mixture was agitated for 2 h at rt, before draining and washing the resin with THF (3 x 20 mL), DMF (3 x 20 mL), DCM (2 x 20 mL), and diethyl ether (2 x 20 mL). The resin was dried under vacuum for 30 min. A test cleavage was conducted on an aliquot of resin and analysed by LC-MS. LC-MS (4.5–90% MeCN, Agilent system) Rt = 5.20 min; m/z 510.90 ([M+Na]$^+$).

Rink amide-loaded (*S*)-2-((*S*)-3-benzyl-2-oxopiperazin-1-yl)-4-methylpentanoic acid **(OP-vi)**

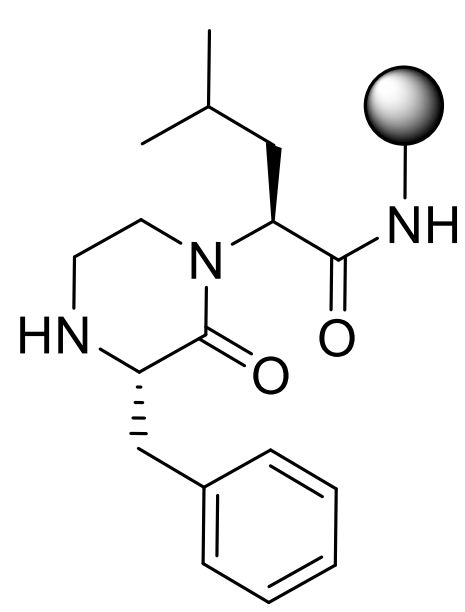

A solution of 2-mercaptoethanol (2.2 mL, 32 mmol, 20 eq) and DBU (4.8 mL, 32 mmol, 20 eq) in anhydrous DMF (5 mL) was added to **OP-v** (2 g, up to 1.6 mmol), and the mixture was agitated for 2 h at rt. Resin was then washed with DMF (3 x 20 mL), DCM (3 x 20 mL), and methanol (3 x 20 mL). A test cleavage was carried out on an aliquot of resin and analysed by LC-MS. LC-MS (4.5–90% MeCN, Agilent system) Rt = 2.99 min; m/z 304.10 ([M+H]$^+$).

Rink amide-loaded (*S*)-2-((*S*)-3-benzyl-4-(1-(tert-butoxycarbonyl)-L-tryptophyl)-2-oxopiperazin-1-yl)-4-methylpentanoic acid **(OP-vii)**

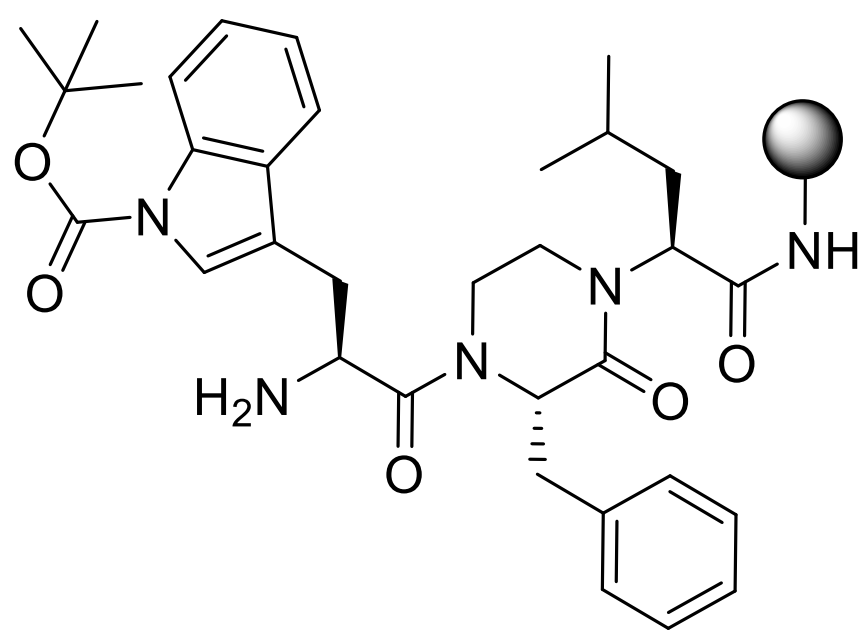

A solution of N_{α}-Fmoc-$N_{(in)}$-Boc-*L*-tryptophan (5.3 g, 10 mmol, 6.3 eq), HATU (3.8 g, 10 mmol, 6.3 eq), and DIPEA (4.4 mL, 2.5 mmol, 5 eq) in DMF (20 mL) was added to **OP-iv** (2 g, up to 1.6 mmol) and the mixture was agitated for 20 h at rt. Resin was then washed with DMF (8 x 20 mL). The amino acid was deprotected by two rounds of treatment with a 20% solution of piperidine in DMF (15 mL) for 20 min. Resin was washed with DMF (8 x 20 mL). A test cleavage was conducted on an aliquot of resin and analysed by LC-MS. LC-MS (4.5–90% MeCN, Agilent system) Rt = 4.11 min; m/z 490.00 ($[M+H]^+$ for Boc deprotected).

Rink amide-loaded (*S*)-2-((*S*)-4-(N^{α}-((*S*)-2-amino-3-(3-chlorophenyl)propanoyl)-1-(tert-butoxycarbonyl)-*L*-tryptophyl)-3-benzyl-2-oxopiperazin-1-yl)-4-methylpentanoic acid **(OP-viii)**

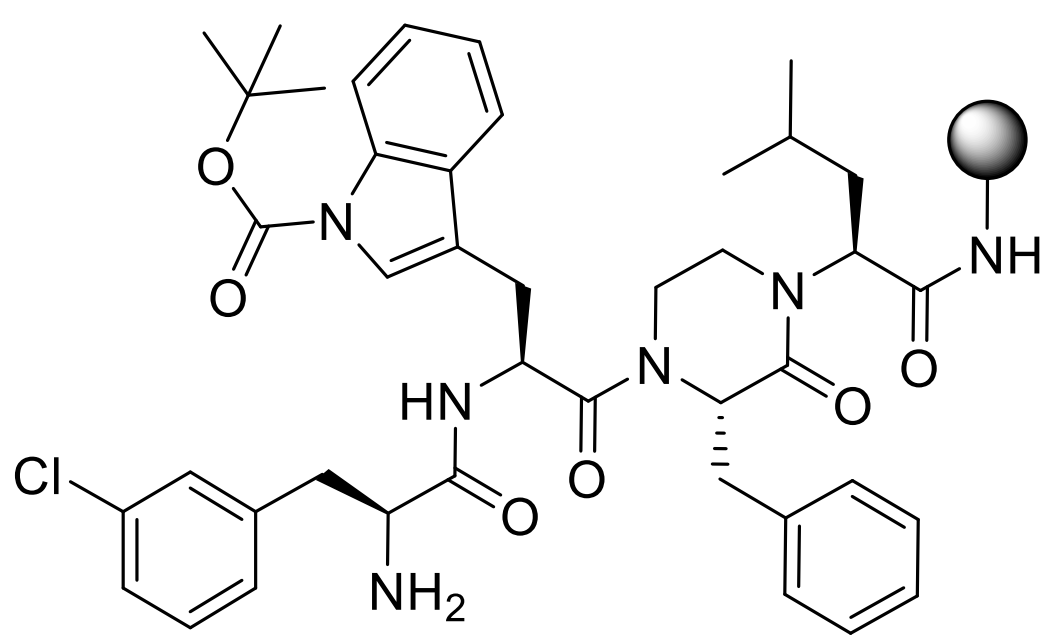

The fourth amino acid was coupled to **OP-viii** (2 g, up to 1.6 mmol) by heating with a solution of *N*-Fmoc-3-chloro-*L*-phenylalanine (2.00 g, 4.8 mmol, 3 eq), HBTU (1.82 g, 4.8 mmol, 3 eq), and DIPEA (1.39 mL, 8 mmol, 5 eq) in DMF (15 mL) to 50°C using a microwave reactor for 2 x 30 min. The amino acid was deprotected by two rounds of treatment with a 20% solution of piperidine in DMF (15 mL) for 20 min. Resin was washed with DMF (3 x 20 mL), DCM (3 x 20 mL), and methanol (3 x 20 mL). A test cleavage was conducted on an aliquot of resin and analysed by LC-MS. LC-MS (4.5–90% MeCN, Agilent system) Rt = 4.26 min; m/z 715.30 ($[M+H]^+$ for Boc deprotected).

Rink amide-loaded (*S*)-2-((*S*)-3-benzyl-4-(1-(tert-butoxycarbonyl)-N$^{\alpha}$-((*S*)-3-(3-chlorophenyl)-2-((2-nitrophenyl)sulfonamido)propanoyl)-*L*-tryptophyl)-2-oxopiperazin-1-yl)-4-methylpentanoic acid **(OP-ix)**

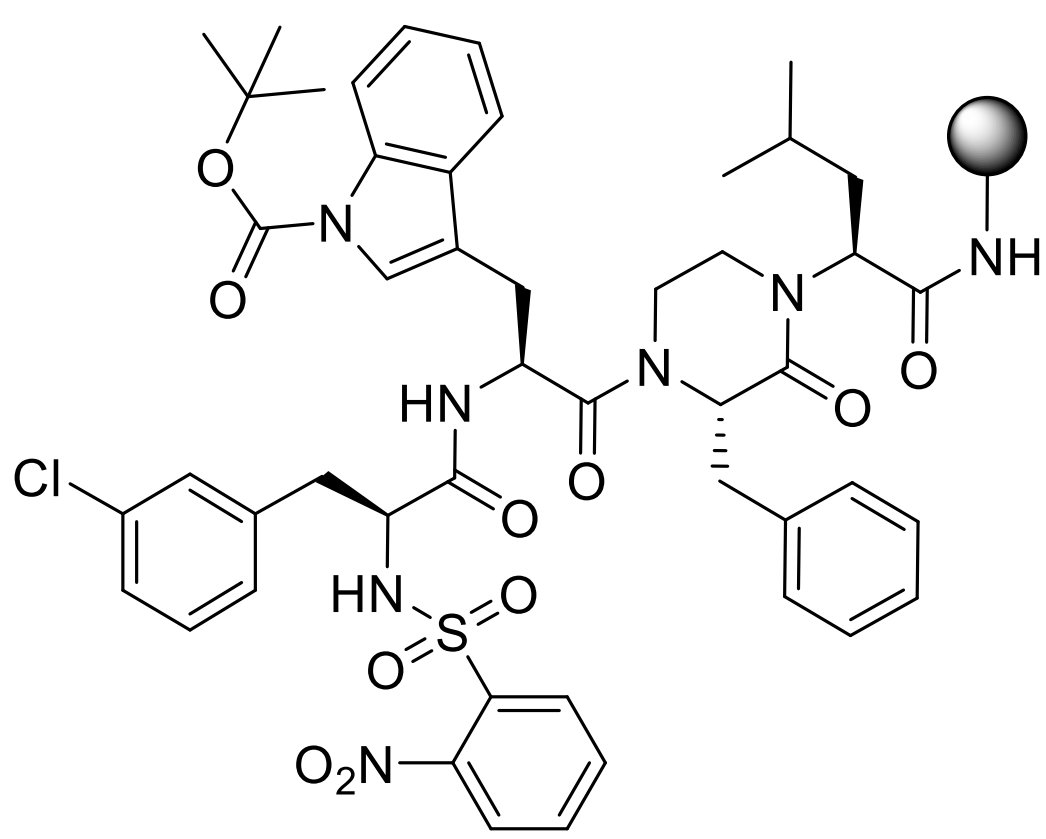

Reaction was carried out as described for **OP-iii**. LC-MS was carried out on a test cleavage sample to ensure complete conversion. LC-MS (4.5–90% MeCN, Agilent system) Rt = 5.66 min; m/z 853.60 ([M-H]$^-$ for Boc deprotected).

Rink amide-loaded (*S*)-2-((*S*)-3-benzyl-4-(*N*$^{\alpha}$-((*S*)-2-((N-(2-bromoethyl)-2-nitrophenyl)sulfonamido)-3-(3-chlorophenyl)propanoyl)-1-(tert-butoxycarbonyl)-*L*-tryptophyl)-2-oxopiperazin-1-yl)-4-methylpentanoic acid **(OP-x)**

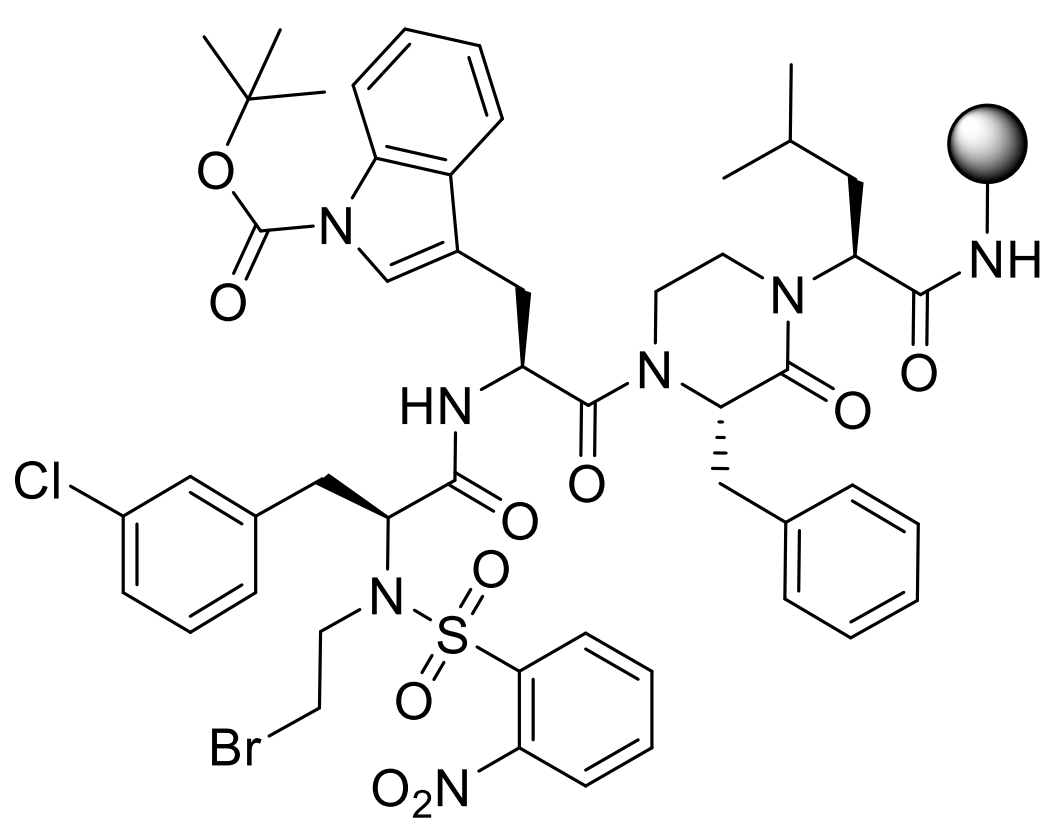

A sealed microwave vial containing triphenylphosphine (4.2 g, 16 mmol, 10 eq) and **OP-ix** (2 g, up to 1.6 mmol) was flushed with nitrogen for 30 min. A solution of 2-bromoethanol (1.1 mL, 16 mmol, 10 eq) and diisopropyl azodicarboxylate (3.1 mL, 16 mmol, 10 eq) in anhydrous THF (15 mL) was added. The reaction mixture was agitated for 18 h at rt. Resin was washed with THF (3 x 20 mL), DMF (3 x 20 mL), and DCM (2 x 20 mL). A test cleavage was conducted on an aliquot of resin and analysed by LC-MS. LC-MS (4.5–90% MeCN, Agilent system) Rt = 5.30 min; m/z 880.20 ([M-HBr-H]$^-$ for Boc deprotected).

Rink amide-loaded (*S*)-2-((*S*)-3-benzyl-4-((*S*)-3-(1-(tert-butoxycarbonyl)-1*H*-indol-3-yl)-2-((*S*)-3-(3-chlorobenzyl)-4-((2-nitrophenyl)sulfonyl)-2-oxopiperazin-1-yl)propanoyl)-2-oxopiperazin-1-yl)-4-methylpentanoic acid **(OP-xi)**

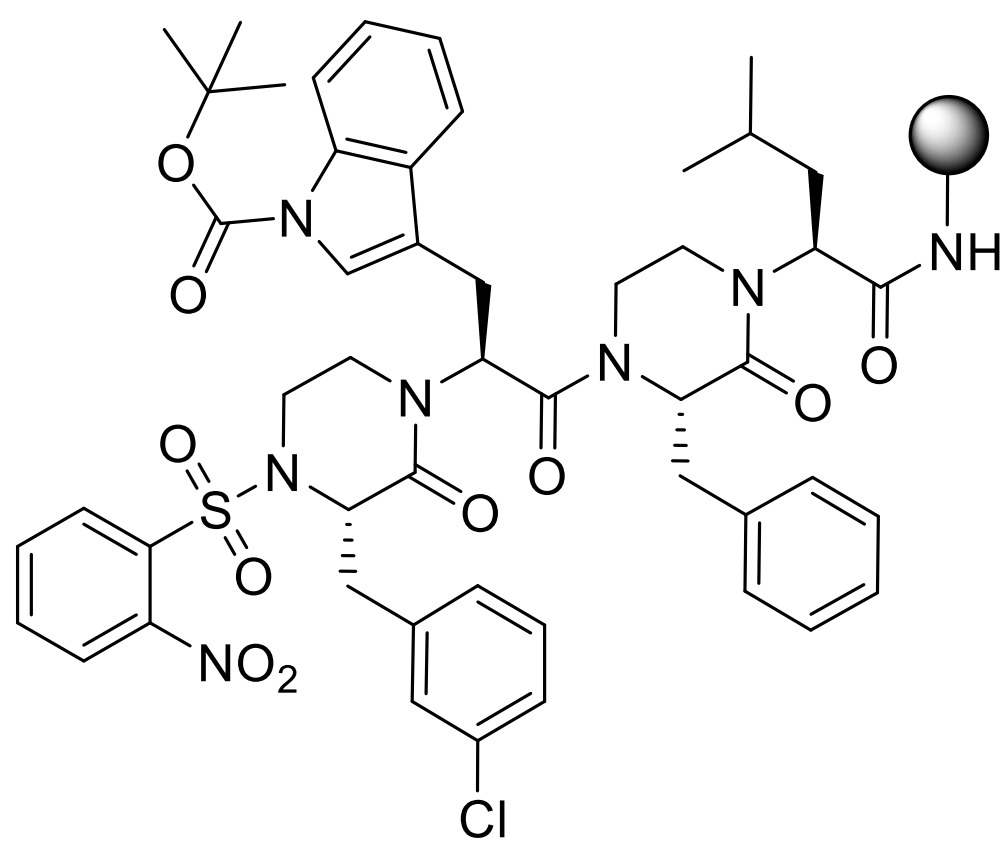

Reaction was carried out as described for **OP-v**. Test cleavage solution was analysed by LC-MS. LC-MS (4.5–90% MeCN, Agilent system) Rt = 5.24 min; m/z 883.10 ($[M+H]^+$ for Boc deprotected).

Rink amide-loaded (*S*)-2-((*S*)-3-benzyl-4-((*S*)-3-(1-(tert-butoxycarbonyl)-1*H*-indol-3-yl)-2-((*S*)-3-(3-chlorobenzyl)-2-oxopiperazin-1-yl)propanoyl)-2-oxopiperazin-1-yl)-4-methylpentanoic acid **(OP-xii)**

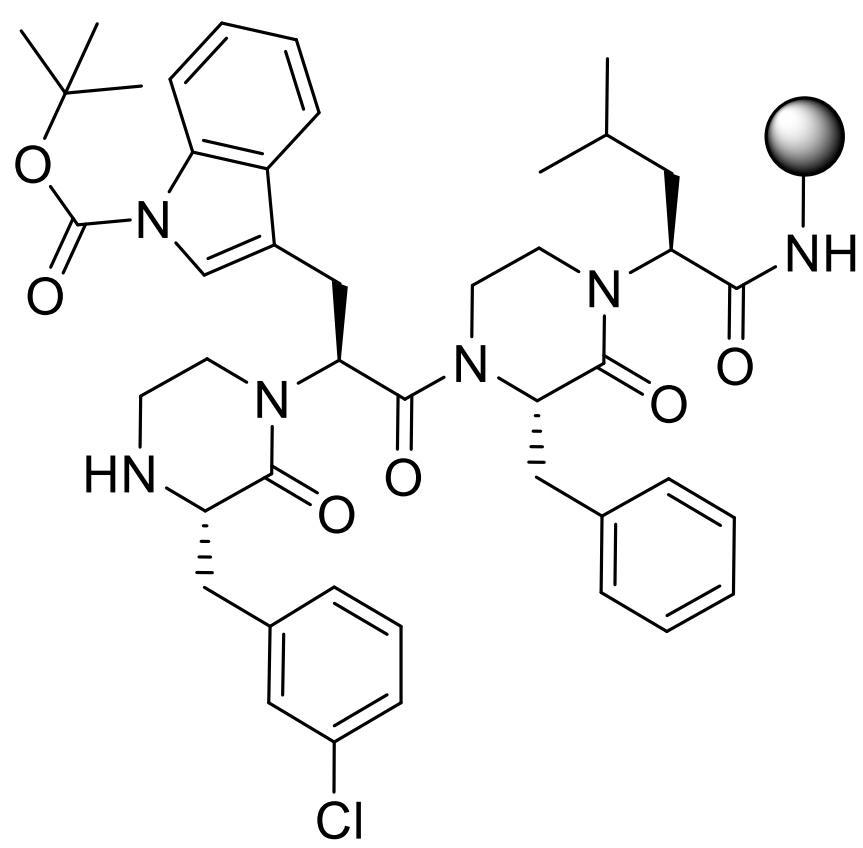

Reaction was carried out as described for **OP-vi**. Test cleavage solution was analysed by LC-MS. LC-MS (4.5–90% MeCN, Agilent system) Rt = 5.74 min; m/z 697.30 ($[M+H]^+$ for Boc deprotected).

(*S*)-2-((*S*)-3-benzyl-4-((*S*)-2-((*S*)-3-(3-chlorobenzyl)-2-oxopiperazin-1-yl)-3-(1*H*-indol-3-yl)propanoyl)-2-oxopiperazin-1-yl)-4-methylpentanamide **(OP)**

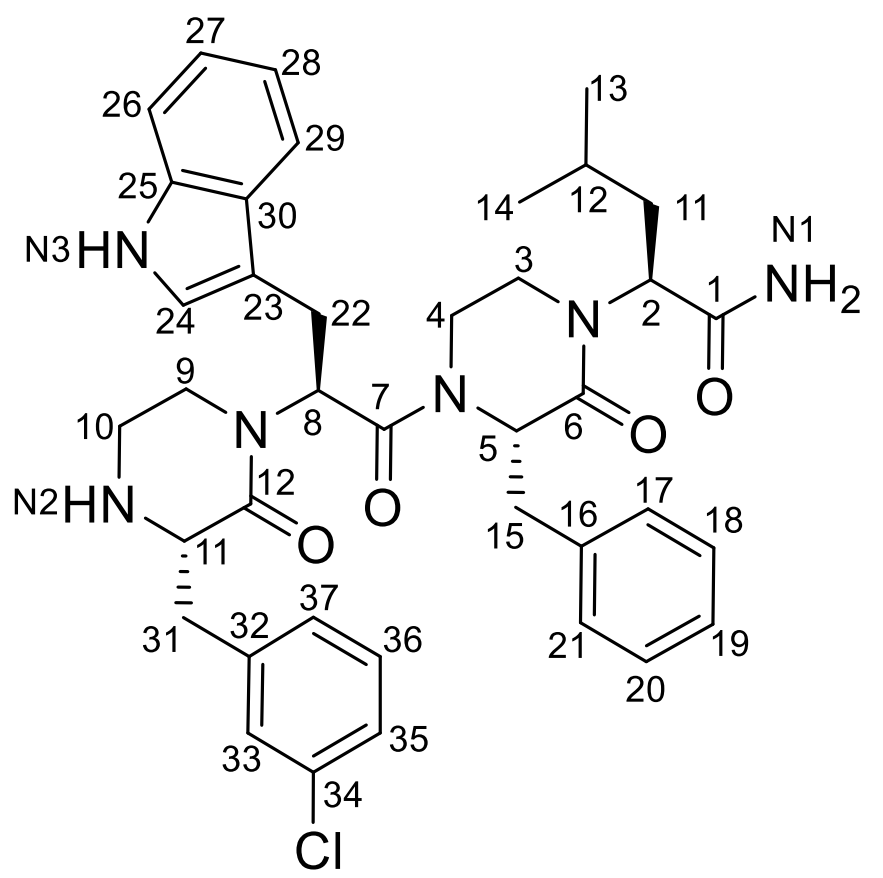

OP was cleaved off the resin using a cleavage cocktail composed of 95% TFA, 2.5% TIPS, and 2.5% water. **OP-xii** (1 g, up to 0.8 mmol) was agitated with the cleavage cocktail for 3 h at rt, and the resulting solution was collected. This was diluted with DCM and dried under a stream of air. The crude product was dissolved in a mixture of MeCN and water, and lyophilised. The solid was then purified by HPLC: three rounds of purification on the Shimadzu preparative HPLC system using a gradient of MeCN and water, both containing 0.1% TFA were carried out, followed by a final purification on the Agilent 1290 Infinity II system using a gradient of MeCN and 10 mM ammonium formate in water. **OP** was isolated as a white solid (5 mg, 1% across 12 steps, 99% purity). ^{1}H NMR (400 MHz, DMSO-d_6) δ 10.77 (d, *J* = 28.0 Hz, 1H, H_{N3}), 7.47 (dd, *J* = 59.5, 7.8 Hz, 2H, H_{Ar}), 7.34 – 6.50 (m, 14H, H_{Ar}, H_{N1}), 5.42 (dt, *J* = 193.7, 7.5 Hz, 1H, H_{CH}), 5.05 – 4.55 (m, 2H, H_{CH}), 4.40 – 3.36 (m, 1H, H_{CH}), 3.28 – 2.55 (m, 12H, H_{CH2}), 2.45 – 1.03 (m, 6H, $H_{CH2,}$ $H_{12,}$ H_{N2}), 0.93 – 0.80 (m, 6H, $H_{13,}$ H_{14}). ^{13}C NMR (101 MHz, $CDCl_3$) δ 175.9, 170.0, 167.6, 163.2, 136.5, 136.3, 136.0, 134.7, 130.4, 129.6, 128.9, 128.4, 128.1, 127.7, 127.6, 127.3, 123.2, 122.4, 119.8, 118.3, 111.6, 109.6, 60.2, 56.3, 55.7, 51.6, 43.1, 38.9, 37.4, 37.3, 36.6, 35.7, 35.1, 25.4, 23.2, 23.0, 21.5. LC-MS (4.5–90% MeCN, Agilent system) Rt = 5.64 min; m/z 697.30 ([M+H]$^+$). HRMS (m/z) calcd. for [M+H]$^+$ $C_{39}H_{46}N_6O_4Cl$: 697.3269, found: 697.3264. Characterisation was matched with that reported by Arora and coworkers.[3]

OP-1

(*S*)-2-((*S*)-3-benzyl-4-((*S*)-2-((*S*)-4-(3-(3-(but-3-yn-1-yl)-3*H*-diazirin-3-yl)propanoyl)-3-(3-chlorobenzyl)-2-oxopiperazin-1-yl)-3-(1*H*-indol-3-yl)propanoyl)-2-oxopiperazin-1-yl)-4-methylpentanamide **(OP-1)**

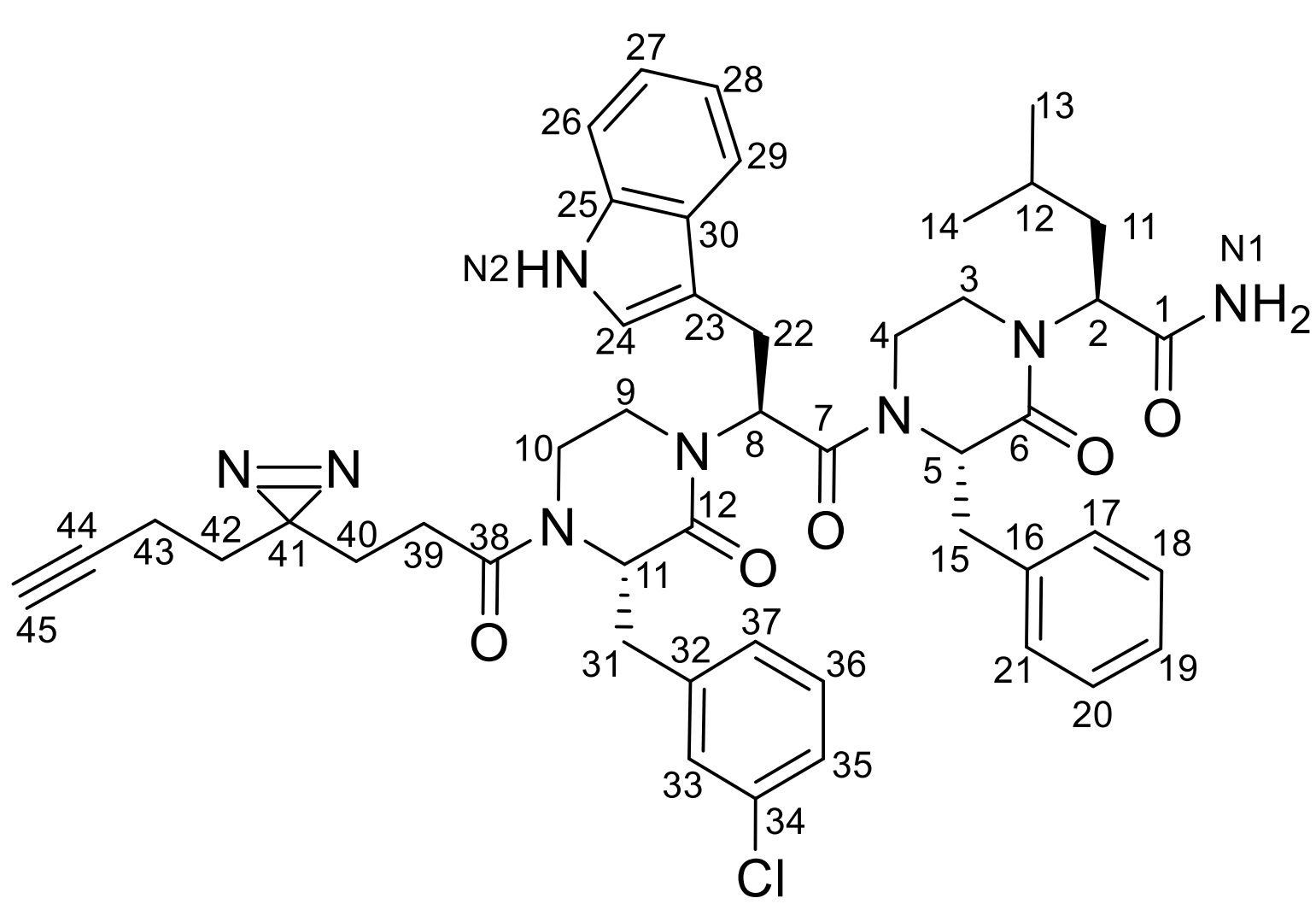

To a solution of **OP** (45 mg, 0.065 mmol, >90% pure) in DMF (1 mL), DIPEA (68 µL, 0.40 mmol, 6 eq), HATU (74 mg, 0.20 mmol, 3 eq), and 3-(3-(but-3-yn-1-yl)-3*H*-diazirin-3-yl)propanoic acid (32 mg, 0.20 mmol, 3 eq) were added. The mixture was stirred in the dark for 18 h, at rt, after which the reaction was quenched by addition of water (10 mL). The mixture was dried by lyophilisation. The crude product was purified by HPLC, to yield **OP-**

1 as a white solid (7.4 mg, 13%, 99% purity for two peaks combined). ¹H NMR (500 MHz, DMSO-d_6) δ 10.83 (d, J = 20.0 Hz, 1H, H_{N2}), 7.63 – 6.53 (m, 16H, H_{Ar}, H_{N1}), 5.91 – 3.51 (m, 8H, H_{CH}, H_{CH2}), 3.23 – 2.54 (m, 13H, H_{CH2}, H_{45}), 2.44 – 1.03 (m, 9H, $H_{CH2,}$ H_{12}), 0.94 – 0.80 (m, 6H, $H_{13,}$ H_{14}). LC-MS (4.5–90% MeCN, Agilent system A) Rt = 6.42 min; m/z 843.20 ([M-H]$^-$). HRMS (m/z) calcd. for [M+H]$^+$ $C_{47}H_{54}N_8O_5Cl$: 845.3906, found: 845.3912

OP-2

(*S*)-2-((*S*)-3-benzyl-4-((*S*)-2-((S)-3-(3-chlorobenzyl)-2-oxopiperazin-1-yl)-3-(1*H*-indol-3-yl)propanoyl)-2-oxopiperazin-1-yl)-4-methylpentanoic acid **(OP2-xiii)**

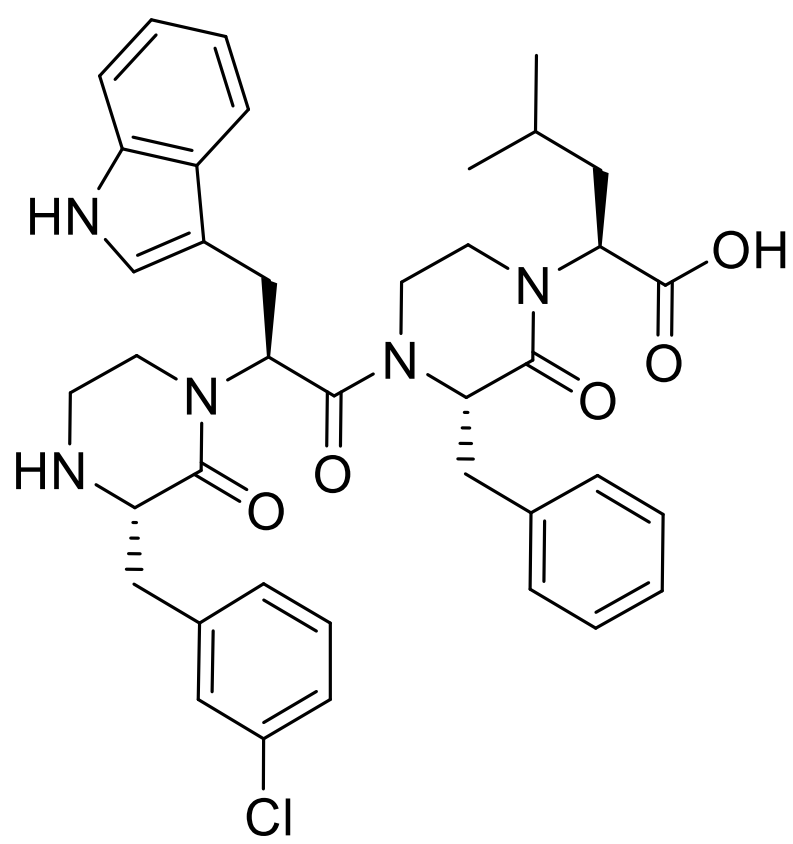

OP2-xiii was synthesised following the same route that was used for **OP**, carried out on Wang resin in place of Rink amide. Here, Wang resin pre-loaded with Fmoc-leucine was used. Purification was carried out by HPLC to yield **OP2-xiii** as a white solid (61 mg, 11% across 12 steps, >93% pure by LC-MS). LC-MS (4.5–90% MeCN, Agilent system A) Rt = 5.08 min; m/z 698.30 ([M+H]$^+$).

(*S*)-2-((*S*)-3-benzyl-4-((*S*)-2-((*S*)-4-(tert-butoxycarbonyl)-3-(3-chlorobenzyl)-2-oxopiperazin-1-yl)-3-(1*H*-indol-3-yl)propanoyl)-2-oxopiperazin-1-yl)-4-methylpentanoic acid **(OP2-xiv)**

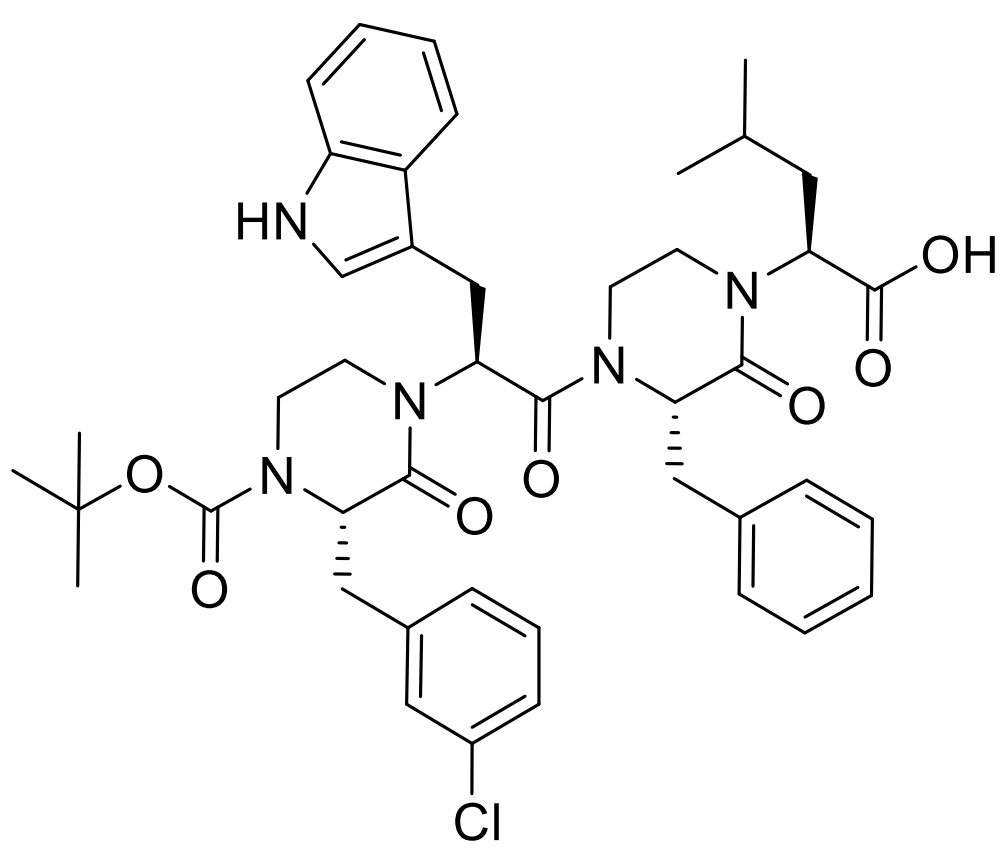

The terminal amine of **OP2-xiii** (60 mg, 0.86 mmol) was protected by reaction with di-tert-butyl decarbonate (20.7 mg, 0.095 mmol, 1.1 eq) in DCM (1 mL). Reaction was monitored by LC-MS, using a basic solvent gradient for elution. Complete conversion was observed after 1 h. The reaction mixture was dried under reduced pressure and the carried forward to the next reaction without workup or purification. LC-MS (4.5–90% MeCN, Agilent system A, basic method) Rt = 5.00 min; m/z 820.20 ([M+Na]$^+$).

tert-butyl (*S*)-4-((*S*)-1-((*S*)-2-benzyl-4-((*S*)-1-((2-(3-(but-3-yn-1-yl)-3*H*-diazirin-3-yl)ethyl)amino)-4-methyl-1-oxopentan-2-yl)-3-oxopiperazin-1-yl)-3-(1*H*-indol-3-yl)-1-oxopropan-2-yl)-2-(3-chlorobenzyl)-3-oxopiperazine-1-carboxylate **(OP2-xv)**

To a solution of **OP2-xiv** (62 mg, 0.079 mmol) in DMF (0.1 mL), DIPEA (69.7 µL, 0.40 mmol, 5 eq) and HATU (90.1 mg, 0.24 mmol, 3 eq) were added. After 15 minutes, 2-(3-(but-3-yn-1-yl)-3*H*-diazirin-3-yl)ethan-1-amine (30.4 µL, 0.24 mmol, 3 eq) was added to the reaction mixture. The mixture was stirred in the dark. After 20 h, when complete conversion to the amide was observed

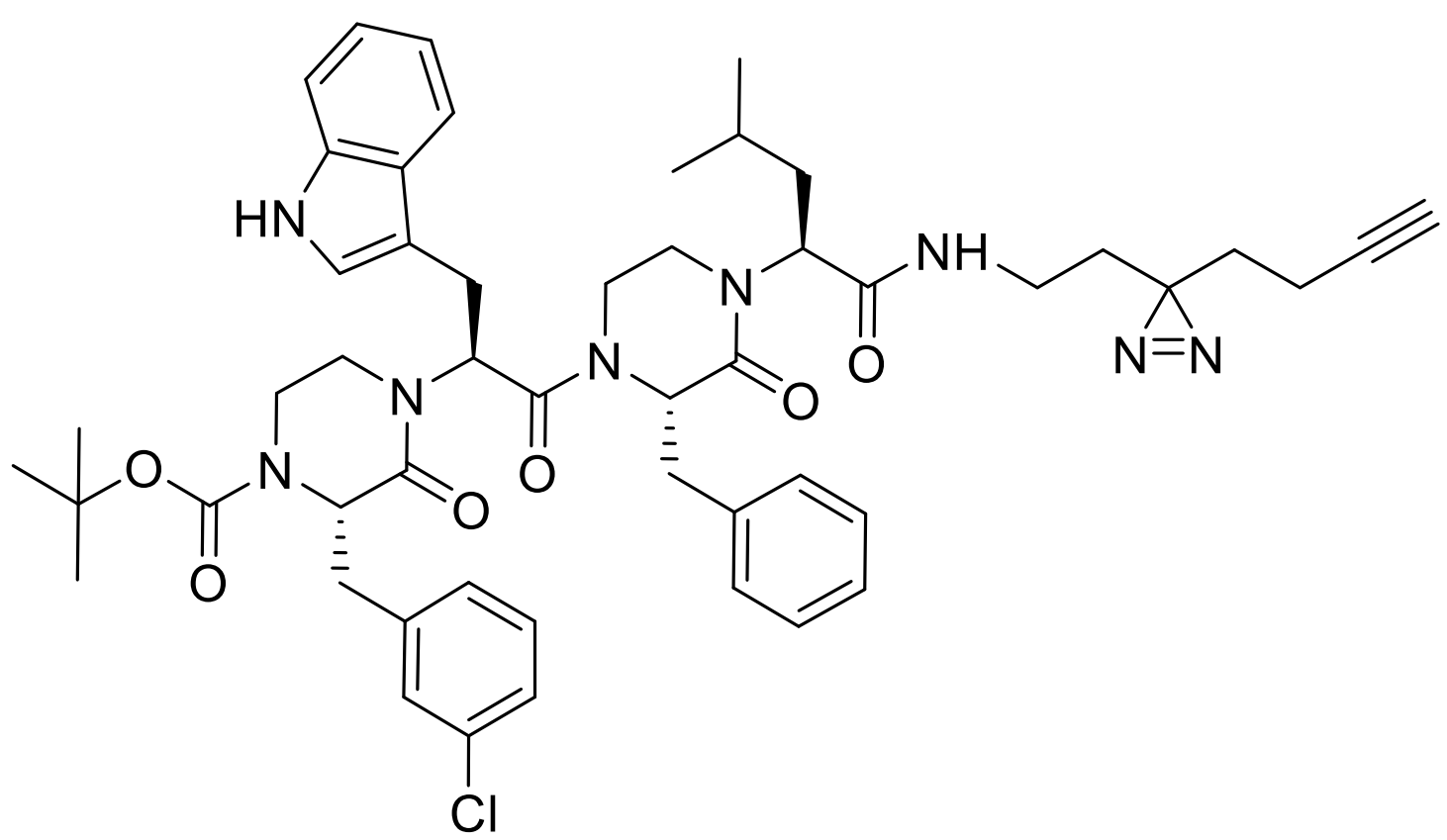

by LC-MS, the reaction was quenched by the addition of water (10 mL). The reaction mixture was dried by lyophilisation and carried forward to the next step without purification.

(*S*)-2-((*S*)-3-benzyl-4-((*S*)-2-((*S*)-3-(3-chlorobenzyl)-2-oxopiperazin-1-yl)-3-(1*H*-indol-3-yl)propanoyl)-2-oxopiperazin-1-yl)-*N*-(2-(3-(but-3-yn-1-yl)-3*H*-diazirin-3-yl)ethyl)-4-methylpentanamide **(OP-2)**

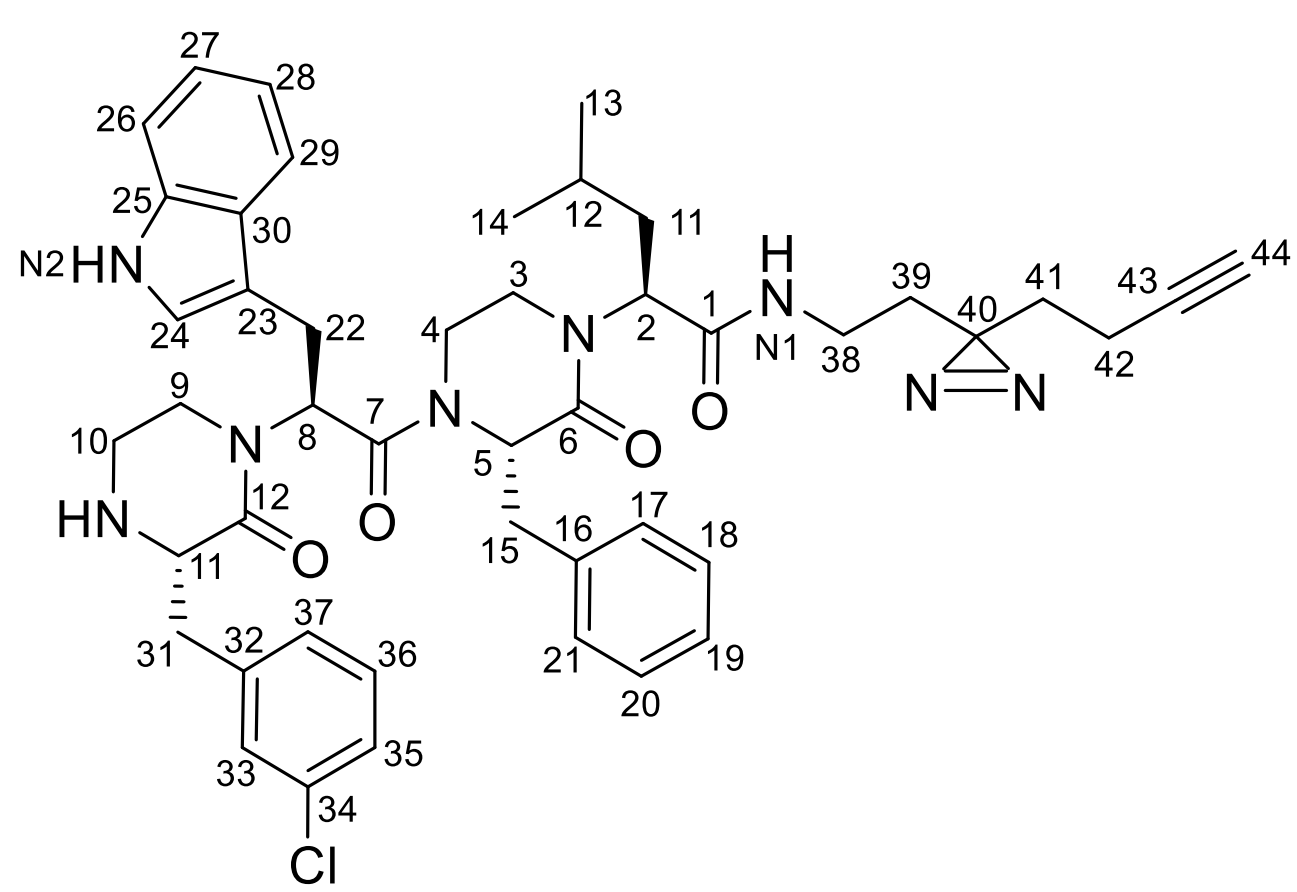

OP2-xv was deprotected by treatment with a solution of TFA (20%) in DCM (10 mL) for 1 h, at rt. Solvent was removed under reduced pressure and the solid was purified by HPLC to yield **OP-2** as a white solid (7 mg, 11%, 98% purity). <u>^{1}H NMR</u> (400 MHz, DMSO-d_6) δ 10.89 (d, *J* = 10.4 Hz, 1H, H_{N2}), 8.03 (dt, *J* = 20.8, 5.4 Hz, 1H, H_{N1}), 7.59 – 6.43 (m, 14H, H_{Ar}), 5.72 – 5.28 (m, 1H, H_{CH}), 5.10 – 4.62 (m, 2H, H_{CH}), 4.50 – 3.54 (m, 3H, H_{CH}, H_{CH2}), 3.30 – 2.57 (m, 14H, H_{CH2}, H_{44}), 2.38 – 1.01 (m, 10H, H_{CH2}, H_{12}), 0.96 – 0.80 (m, 6H, $H_{13,}$ H_{14}). <u>LC-MS</u> (4.5–90% MeCN, Agilent system A, basic method) Rt = 6.61 min; m/z 815.30 ([M-H]$^-$). <u>HRMS</u> (m/z) calcd. for [M+H]$^+$ $C_{46}H_{54}N_8O_4Cl$: 817.3957, found: 817.3958.

Null probe

3-(3-(but-3-yn-1-yl)-3*H*-diazirin-3-yl)-*N*-phenylpropanamide

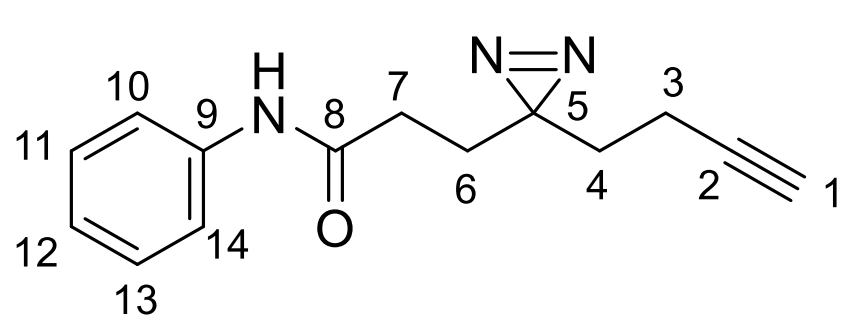

To a solution of 3-(3-(but-3-yn-1-yl)-3*H*-diazirin-3-yl)propanoic acid (10 mg, 0.06 mmol), DIPEA (34 µL, 0.20 mmol, 3.3 eq), and HATU (35 mg, 0.09 mmol, 1.5 eq) in DMF (0.5 mL), aniline (5.6 µL, 0.06 mmol, 1 eq) was added. The mixture was stirred in the dark for 18 h, at rt, after which the reaction was quenched by addition of water (5 mL). The product was extracted into ethyl acetate (3 x 15 mL), dried under reduced pressure, and purified by HPLC, to yield **NP** as a white solid (7.8 mg, 53%). <u>^{1}H NMR</u> (400 MHz, $CDCl_3$) δ 7.49 (d, *J* = 8.2 Hz, 2H, H_{10}, H_{14}), 7.32 (t, *J* = 7.9 Hz, 2H, H_{11}, H_{13}), 7.12 (q, *J* = 7.1 Hz, 1H, H_{12}), 2.12 (t, *J* = 7.5 Hz, 2H, H_3), 2.04 (td, *J* = 7.4, 2.5 Hz, 2H, H_7), 1.99 (s, 1H, H_1), 1.94 (t, *J* = 7.6 Hz, 2H, H_6), 1.68 (t, *J* = 7.4 Hz, 2H, H_4). <u>^{13}C NMR</u> (101 MHz, $CDCl_3$) δ 169.4 (C_8), 137.7 (C_9), 129.2 (C_{11}, C_{13}), 124.7 (C_{12}), 120.0 (C_{10}, C_{14}), 82.8 (C_2), 77.5 (C_2), 69.4 (C_1), 32.6 (C_4), 31.5 (C_6), 28.4 (C_7), 28.0 (C_5), 13.5 (C_3). <u>LC-MS</u> (4.5–90% MeCN, Agilent system

A) Rt = 4.86 min; m/z 240.10 ([M-H]$^-$). HRMS (m/z) calcd. for [M+H]$^+$ $C_{14}H_{16}N_3O$: 242.1293, found: 242.1293.

Fluorescence anisotropy

MDM2$^{17\text{-}135}$L33E was expressed and purified as previously described.[8, 9] A protein titration was carried out to determine the K_d of the fluorescein-labelled p53 tracer, which was found to be 64.8 ± 3.5 nM. The fluorescence anisotropy competition assay was carried out as previously described.[9, 10]

Analyte solutions were prepared as 5 mM stocks in DMSO and diluted to 1 mM in 20 mM tris pH 7.6, 150 mM NaCl, 0.01% Triton-X-100. Serial dilutions (1:2) of the analyte solutions were carried out in black flat-bottom 384-well plates (Grenier Bio-one) in triplicate. To this, solutions of MDM2 and fluorescein-labelled p53 were added to a final concentration of 150 nM and 25 nM respectively. The highest concentration of each analyte tested was 250 μM (in 10% DMSO). The plate was read using a CLARIOstar Plus plate reader (BMG Labtech), using excitation and emission wavelengths of 482 nm (16 nm bandwidth) and 530 nm (40 nm bandwidth), respectively, with a longpass dichroic beam split of 504 nm. Control samples, containing dilutions of the analyte and the protein, but no tracer, were also run, and total intensities were subtracted from the test samples. Intensity and anisotropy were calculated using the equations below:

$$I = (2PG) + S \qquad (1)$$

$$r = (S - PG)/I \qquad (2)$$

Where I = corrected total intensity; P = perpendicular intensity; S = parallel intensity; G = instrument factor which was set to 1; r = anisotropy. The data was fit to a [Inhibitor] vs. response – variable slope (four parameter) model using GraphPad Prism 10 (Dotmatics).

Cell-based assays

Cell culture

SJSA-1 cells were provided by the Francis Crick Institute Cell Services facility and cultured in Roswell Park Memorial Institute Medium 1640 (Gibco), each supplemented with 10% foetal bovine serum. All cell lines were cultured at 37°C in a 5% CO_2 humidified incubator.

Cell viability assay (MTS assay)

MTS assay was carried out using CellTiter 96® AQueous One Solution cell proliferation assay reagent (Promega), as per the manufacturer's instructions. Cells were seeded in a clear flat-bottom tissue culture-treated 96-well plate (Corning) at a density of 2000 cells/well. After 24 h, cells were treated with a range of probe concentrations (50 μM to 390 nM) in 1% DMSO in serum-containing media for 72 hours. Media was then replaced, the assay reagent was added, and the plate was incubated at 37°C for 2.5–3 h. Absorbance was recorded at 480 nm using an Envision 2104 multilabel reader (PerkinElmer). The experiment was carried out as technical replicates (n

= 3). The data was fit to a [Inhibitor] vs. response – variable slope (four parameter) model using GraphPad Prism 10.

Live cell imaging

Cells were seeded in a clear, flat-bottom tissue culture-treated 48-well plate (Falcon) at a density of 5000 cells/well. After 24 h, once cells had adhered, they were treated in triplicate with the analytes in 1% DMSO. SYTOX green nucleic acid stain (Invitrogen) was added to a final concentration of 250 nM. Puromycin (2 μg/mL) was used as a positive control and 1% DMSO was used as a negative control. The plate was placed in an Incucyte S3 cell imager (Sartorius, Essen Bioscience). Green filter and phase readings were taken (4 images per well) every 4 h, over the course of 5 days. Data analysis was conducted using the integrated analysis software (IncuCyte 2021B). The experiment was carried out as technical replicates (n = 3). The mean ± standard error for the confluence and total green object integrated intensity per well (measured in green calibrated units, GCU) was plotted as a function of time using GraphPad Prism 10.

Western blot

Cells were observed under a microscope to ensure that they were healthy and at approximately 80–90% confluence. Media was aspirated and replaced with serum-free media containing molecules in 1% DMSO. Cells were incubated in the dark with the compounds for 4 h. The media was removed, and the cells were washed twice with PBS (Sigma-Aldrich). Cells were lysed on the plate using either (i) radioimmunoprecipitation assay (RIPA) buffer (ThermoScientific) supplemented with 1 x complete protease inhibitor cocktail, or (ii) with 1% sodium dodecyl sulphate (SDS, Sigma-Aldrich) in PBS supplemented with 1 x complete protease inhibitor cocktail and Benzonase® nuclease (Millipore). A cell scraper was used to aid cell lysis. The protein content of the lysates was quantified using the DC protein assay (Bio-Rad) as per the manufacturer's instructions, and the protein concentration the samples was normalised to 1–2 mg/mL.

Proteins were resolved by SDS-PAGE. Mini-PROTEAN TGX precast protein gels (4–15%), were used. Laemmli loading buffer (Bio-Rad) with 5% 2-mercaptoethanol was added and the lysates were heated to 95°C for 5–10 min prior to loading. Proteins were blotted onto a 0.45 μm nitrocellulose blotting membrane (Amersham) using a Trans-Blot Turbo Transfer system (Bio-Rad), as per the manufacturer's instructions. The membrane was blocked using 5% milk in tris-buffered saline with 0.1% Tween 20 (TBST) for 1 h at rt. Primary antibodies for MDM2 (Abcam, ab16895, 1:1000) and GAPDH (Abcam, 1:1000) were prepared in 5% milk in TBST. Blocked membranes were incubated with the primary antibodies for 1 h at rt. Membranes were incubated with fluorophore-conjugated secondary antibodies (LiCor, 1:10000 in 5% BSA in TBST) at rt for 1 h, washed with TBST (3 x 5 min), and imaged using a LiCor Odyssey M imager for fluorescence at 800 nm.

Protein labelling

Intact protein mass spectrometry

Recombinant MDM2 (2 μM) was treated with 4 μM probe in 1% DMSO in HEPES pH 8.0. Sample treated with 1% DMSO was used as a negative control. Samples were agitated for 30 min at rt, then irradiated at 365 nm for 10 min on ice. The experiment was carried out in triplicate, in a clear flat-bottom 96-well plate (Grenier). LC-MS analysis was carried out using an AdvanceBio 6545XT LC/Q-TOF system (Agilent) fitted with an ACQUITY UPLC Protein BEH C4 VanGuard pre-column (Waters). Data was processed using BioConfirm (Agilent). The TICs were extracted for the region containing protein, and deconvoluted using MaxEnt. Percentage protein labelling was calculated using equation 4:

$$\text{Percentage protein labelling} = \frac{\text{Labelled protein intensity}}{\text{Unlabelled protein intensity+Labelled protein intensity}} \times 100 \qquad (3)$$

Recombinant protein labelling gel

Recombinant MDM2 (0.5 μM) was treated with 1 μM probes ± 20 μM parent molecules in 4% DMSO in HEPES pH 8.0. Samples (20 μL) were subjected to CuAAC with CalFluor 647 azide (BroadPharm). A CuAAC master mix was prepared by mixing stock solutions of $CuSO_4$ (50 mM in water), sodium ascorbate (50 mM in water), tris(benzyltriazolylmethyl)amine (TBTA; 10 mM in DMSO) and CalFluor-647 azide (10 mM in DMSO) in a ratio of 2:2:1:1. To each sample, 1.5 μL of the CuAAC master mix was added and samples were agitated for 1 h at rt. Reaction was quenched by addition of EDTA to a final concentration of 10 mM. Colourless Laemmli loading buffer containing 5% 2-mercaptoethanol was added, samples were heated to 95°C for 5 min, and protein was resolved by SDS-PAGE using a Mini-PROTEAN TGX precast protein gel (4–15%, Bio-Rad). The gel was imaged using a LiCor Odyssey M imager at 700 nm.

The gel was stained using a PierceTM silver stain kit (Thermo Scientific), as per the manufacturer's instructions, and imaged for absorbance of transillumination at 480 nm using a LiCor Odyssey M imager.

In-cell labelling

The procedure was modified from that previously described.[11, 12]

Photo-crosslinking

Cells were treated with the probes in 1% DMSO in serum-free media and incubated in the dark for 4 h. The media was removed, and the cells were washed with PBS. PBS was added to cover the surface of the plate. The plates were placed on ice and irradiated at 365 nm for 120 s. The PBS was then removed, and cells were lysed using 1% SDS in PBS supplemented with complete protease inhibitor cocktail and Benzonase® nuclease. The DC protein assay (Bio-Rad) was used

to determine the protein content of the lysates, and protein concentration was normalised to 1–2 mg/mL.

CuAAC and protein precipitation

A CuAAC master mix was prepared by mixing stock solutions of AzTB or azide-PEG3-biotin (AzB; Sigma-Aldrich; 10 mM in DMSO), $CuSO_4$ (50 mM in water), sodium ascorbate (50 mM in water), and TBTA (10 mM in DMSO) in a ratio of 1:2:2:1. The mixture was vortexed and allowed to stand for 2 min before adding 6 µL of the master mix per 100 µL of lysate. The reaction was agiated at rt for 1 h, and then quenched by the addition of EDTA to a final concentration of 5 mM.

Proteins were then precipitated by addition of 1 volume of methanol and 0.25 volumes of chloroform to the lysate. The mixtures were vortexed and centrifuged at 4°C, 17,000 x g for 5 min. The supernatant was discarded, and the pellet was washed with methanol (2 x 2.5 volumes). The protein pellet was allowed to air dry, before redissolving in 0.2% SDS in 50 mM HEPES pH 8.0 to a final protein concentration of approximately 1 mg/mL (50% protein loss during precipitation was assumed).

Neutravidin enrichment

Biotin enrichment was carried out using Neutravidin agarose resin (ThermoScientific). The resin was washed with 0.2% in 50 mM HEPES pH 8.0 (x 3). One volume of resin was added to every 2.5 volumes of lysate, and the samples were agitated for 2 h at rt. The samples were then centrifuged, and the supernatant was collected for Western blot analysis. The resin was washed with 0.2% SDS in 50 mM HEPES pH 8.0 (x 3).

To analyse by Western blot, the captured proteins were eluted from the resin by adding 2x Lamelli loading buffer with 5% 2-mercaptoethanol and heating to 95°C for 10 min. The samples were centrifuged, and the supernatant was collected for analysis by SDS-PAGE and Western blot. The gels were imaged using the LiCor Odyssey M imager to detect fluorescence at 520 nm. Western blot was carried out as described.

LC-MS/MS analysis

Peptides for proteomics analysis were loaded onto Evotips (Evosep) as per the manufacturer's instructions. For whole proteome analysis, 200 ng peptide was loaded, while for pulldown sample, half of the sample collected was loaded for analysis. All experiments were conducted as four biological replicates.

For whole proteome experiments:

Peptides were analysed by nanoLC-MS/MS using an Evosep One (Evosep) coupled with a timsTOF HT (Bruker) equipped with an 8 cm × 150 μm, 1.5 μm analytical column (Evosep). 200 ng peptides were separated by the Evosep 60SPD workflow (Analytical solvents A: 0.1% FA and B: acetonitrile plus 0.1% FA). Column was held at 40 °C. Data were acquired in data-independent acquisition (DIA) PASEF mode with the following settings: m/z range from 100 m/z to 1700 m/z, ion mobility range from 1/K0 = 1.30 to 0.85 Vs/cm^2 using equal ion accumulation and ramp times in the dual TIMS analyser of 100 ms each. Each cycle consisted of 8 PASEF ramps covering 21 mass steps each with 25 Da windows each with 2/3 non-overlapping ion mobility windows covering the 475 to 1000 m/z range and 0.85 and 1.26 Vs/cm^2 ion mobility range. The collision energy was lowered as a function of increasing ion mobility from 59 eV at 1/K0 = 1.6 Vs/cm^2 to 20 eV at 1/K0 = 0.6 Vs/cm^2.

For pulldown experiments:

Peptides were analysed by nanoLC-MS/MS using an Evosep One (Evosep) coupled with a timsTOF HT (Bruker) equipped with an 8 cm × 150 μm, 1.5 μm analytical column (Evosep). 200 ng peptides were separated by the Evosep 60SPD workflow (Analytical solvents A: 0.1% FA and B: acetonitrile plus 0.1% FA). Column was held at 40 °C. Data were acquired in data-dependent acquisition (DDA) PASEF mode with the following settings: m/z range from 100 m/z to 1700 m/z, ion mobility range from 1/K0 = 1.30 to 0.85 Vs/cm^2 using equal ion accumulation and ramp times in the dual TIMS analyzer of 100 ms each. The collision energy was lowered as a function of increasing ion mobility from 59 eV at 1/K0 = 1.6 Vs/cm^2 to 20 eV at 1/K0 = 0.6 Vs/cm^2. Isolation width was lowered as a function of decreasing m/s from 3 m/z at 800 m/z to 2 m/z at 700 m/z. Active exclusion was applied for 0.4 min. Each cycle consistent of 4 PASEF ramps (total cycle time 0.53 s) with 2.75 ms measuring time allowed for each selected precursor.

Data processing

Data processing for DIA

diaPASEF Bruker .d files were processed using library-free analysis in DIA-NN (version 1.8.1) [13] using the following parameters: Human database (Downloaded from Uniprot on 6 June 2022 containing 79334 proteins); “deep learning-based spectra and RTs prediction” was enabled; trypsin with 1 missed cleavages; N-term Excision, C carbamidomethylation, Oxidation and N terminal Acetylation were enabled with maximum 2 variable modifications; MBR was enabled; quantification strategy set to “Robust LC (high precision)”; heuristic protein inference was disabled; Mass and MS1 accuracy set to 0.

Data processing for DDA

ddaPASEF Bruker .d files were processed using Fragpipe version 20.0 (Nesvilab).[14] Data was searched against a human reference proteome with isoforms (Uniprot, UP000005640, accessed 6 June 2022, 203368 proteins) with 50% decoys and contaminants added. The built-in label-free quantification-match between runs workflow was used. Bruker .d files were searched using MSFragger (version 3.8) The following parameters were used: strict trypsin digestion; a maximum of 2 missed cleavages allowed; precursor ion tolerance of 20 ppm; trimming of protein N-terminal methionine; oxidation (M) and N terminal acetylation as variable modifications; carbamidomethylation (C) as a fixed modification. MaxLFQ minimum ions was set to 1 and the retention time tolerance for match between runs was set to 2 min. All other default parameters were used for processing. MSFragger search results were processed using Percolator (version 3.5) for peptide-spectrum match validation, followed by Philosopher (version 5.0.0) for protein and FDR filtering. Label-free quantification values were calculated using the MaxLFQ algorithm using IonQuant (version 1.9.8) with match between runs enabled and min ions set as 1.

Data analysis using Perseus

Data analysis was carried out using Perseus version 1.6.2.3.[15] Intensities (DIA data) or MaxLFQ intensities (DDA data) were loaded for analysis. A text filter was applied to remove contaminants and data was log2 transformed. Rows were annotated to group replicates, and data was filtered for valid values in at least three replicates in at least one condition. Data was normalized by subtracting the median from the columns and missing values were imputed based on normal distribution. Volcano plots were generated by plotting the fold-changes of the protein intensities against the -logp values for their significance. These were calculated by carrying out a two-sample t-test of the intensity values for each protein in the two conditions (permutation-based FDR = 0.05, S0 = 0.1). Data was plotted in GraphPad Prism version 10 and x- and y-axis cut-offs were set to ±1 and 2 respectively.

Online data repository

Raw Data files can be found at 10.14469/hpc/15292 and the following DOIs:

Data Type	DOI
NMR Spectra	10.14469/hpc/15293
Fluorescence Anisotropy	10.14469/hpc/15294
Cell toxicity (MTS, confluence, and cytotoxicity)	10.14469/hpc/15295
Intact Protein Labelling	10.14469/hpc/15296

The mass spectrometry proteomics data have been deposited to the ProteomeXchange Consortium (https://www.proteomexchange.org) via the PRIDE partner repository with the dataset identifiers listed below:[16]

Experiment	Project accession code
Whole proteome characterisation following treatment with α-helix mimetic inhibitors of MDM2	PXD063398
AfBPP of α-helix mimetics in SJSA-1 cells	PXD063384
AfBPP of N-substituted oligobenzamide α-helix mimetics	PXD063390
AfBPP of pyrrolopyrimidine α-helix mimetics	PXD063432
AfBPP of oxopiperazine α-helix mimetics	PXD063387

References

[1] M. Broncel, R. A. Serwa, P. Ciepla, E. Krause, M. J. Dallman, A. I. Magee, E. W. Tate, Mutlifunctional reagents for quantitative proteome-wide analysis of protein modification in human cells and dynamic profiling of protein lipidation during vertebrate development, *Angew Chem Int Ed Engl* **2015**, *54*, 5948-5951.

[2] C. R. Kennedy, A. Goya Grocin, T. Kovacic, R. Singh, J. A. Ward, A. R. Shenoy, E. W. Tate, A Probe for NLRP3 Inflammasome Inhibitor MCC950 Identifies Carbonic Anhydrase 2 as a Novel Target, *ACS Chem Biol* **2021**, *16*, 982-990.

[3] B. B. Lao, K. Drew, D. A. Guarracino, T. F. Brewer, D. W. Heindel, R. Bonneau, P. S. Arora, Rational Design of Topographical Helix Mimetics as Potent Inhibitors of Protein-Protein Interactions, *J Am Chem Soc* **2014**, *136*, 7877-7888.

[4] J. H. Lee, Q. Zhang, S. Jo, S. C. Chai, M. Oh, W. Im, H. Lu, H. S. Lim, Novel Pyrrolopyrimidine-Based α-Helix Mimetics *J Am Chem Soc* **2011**, *133*, 676-679.

[5] A. Barnard, K. Long, H. L. Martin, J. A. Miles, T. A. Edwards, D. C. Tomlinson, A. Macdonald, A. J. Wilson, Selective and potent proteomimetic inhibitors of intracellular protein-protein interactions, *Angew Chem Int Edit* **2015**, *54*, 2960-2965.

[6] K. Long, T. A. Edwards, A. J. Wilson, Microwave assisted solid phase synthesis of highly functionalized N-alkylated oligobenzamide α-helix mimetics, *Bioorg Med Chem* **2013**, *21*, 4034-4040.

[7] S. Eissler, M. Kley, D. Bachle, G. Loidl, T. Meier, D. Samson, Substitution determination of Fmoc-substituted resins at different wavelengths, *J Pept Sci* **2017**, *23*, 757-762.

[8] A. I. Green, F. Hobor, C. P. Tinworth, S. Warriner, A. J. Wilson, A. Nelson, Activity-Directed Synthesis of Inhibitors of the p53/hDM2 Protein-Protein Interaction, *Chem Eur J* **2020**, *26*, 10682-10689.

[9] J. P. Plante, T. Burnley, B. Malkova, M. E. Webb, S. L. Warriner, T. A. Edwards, A. J. Wilson, Oligobenzamide proteomimetic inhibitors of the p53/hDM2 protein-protein interaction, *Chem Commun* **2009**, 5091-5093.

[10] L. J. Liu, B. He, J. A. Miles, W. Wang, Z. Mao, W. I. Che, J. J. Lu, X. P. Chen, A. J. Wilson, D. L. Ma, C. H. Leung, Inhibition of the p53/hDM2 protein-protein interaction by cyclometallated iridium(III) compounds, *Oncotarget* **2016**, *7*, 13965-13975.

[11] R. T. Howard, P. Hemsley, P. Petteruti, C. N. Saunders, J. A. M. Bermejo, J. S. Scott, J. W. Johannes, E. W. Tate, Structure-Guided Design and In-Cell Target Profiling of a Cell Active Target Engagement Probe for PARP Inhibitors, *ACS Chemical Biology* **2020**, *15*, 325-333.

[12] W. W. Kallemeijn, T. Lanyon-Hogg, N. Panyain, A. G. Grocin, P. Ciepla, J. Morales-Sanfrutos, E. W. Tate, Proteome-wide analysis of protein lipidatino using chemical probes : in-gel fluorescence visualization, identification and quantification of N-myristoylation, N- and S-acylation, O-cholesterolation, S-farnesylation and S-geranylgeranylation, *Nat Protoc* **2021**, *16*, 5083-5122.

[13] V. Demichev, C. B. Messner, S. I. Vernardis, K. S. Lilley, M. Ralser, DIA-NN : neural networks and interference correction enable deep proteome coverage in high throughput, *Nat Methods* **2020**, *17*, 41-44.

[14] F. Yu, S. E. Haynes, G. C. Teo, D. M. Avtonomov, D. A. Polasky, A. I. Nesvizhskii, Fast Quantitative Analysis of timsTOF PASEF Data with MSFragger and IonQant, *Mol Cell Proteomics* **2020**, *19*, 1575-1585.

[15] J. Cox, M. Mann, 1D and 2D annoationa enrichment: a statistical method integrating quantitative proteomics with complementary high-throughput data, *BMC Bioinformatics* **2012**, *13*.

[16] Y. Perez-Riverol, J. W. Bai, C. Bandla, D. García-Seisdedos, S. Hewapathirana, S. Kamatchinathan, D. J. Kundu, A. Prakash, A. Frericks-Zipper, M. Eisenacher, M. Walzer,

S. B. Wang, A. Brazma, J. A. Vizcaíno, The PRIDE database resource in 2022: a hub for mass spectrometry-based proteomics evidences, *Nucleic Acids Res* **2022**, *50*, D543-D552.

NMR spectra of final products

^{1}H NMR of **OB-a** at rt in $CDCl_3$

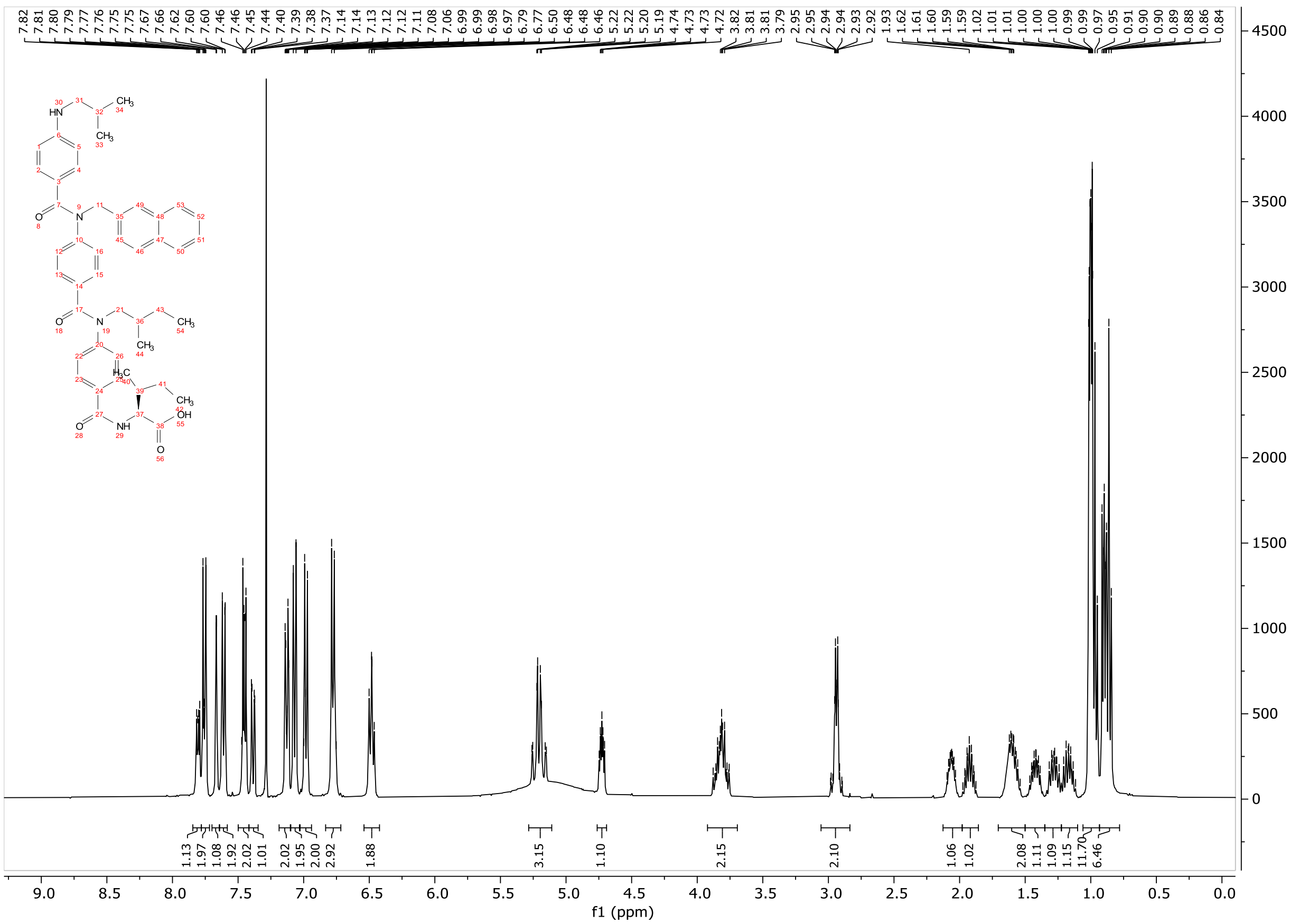

^{13}C NMR of **OB-a** at rt in $CDCl_3$

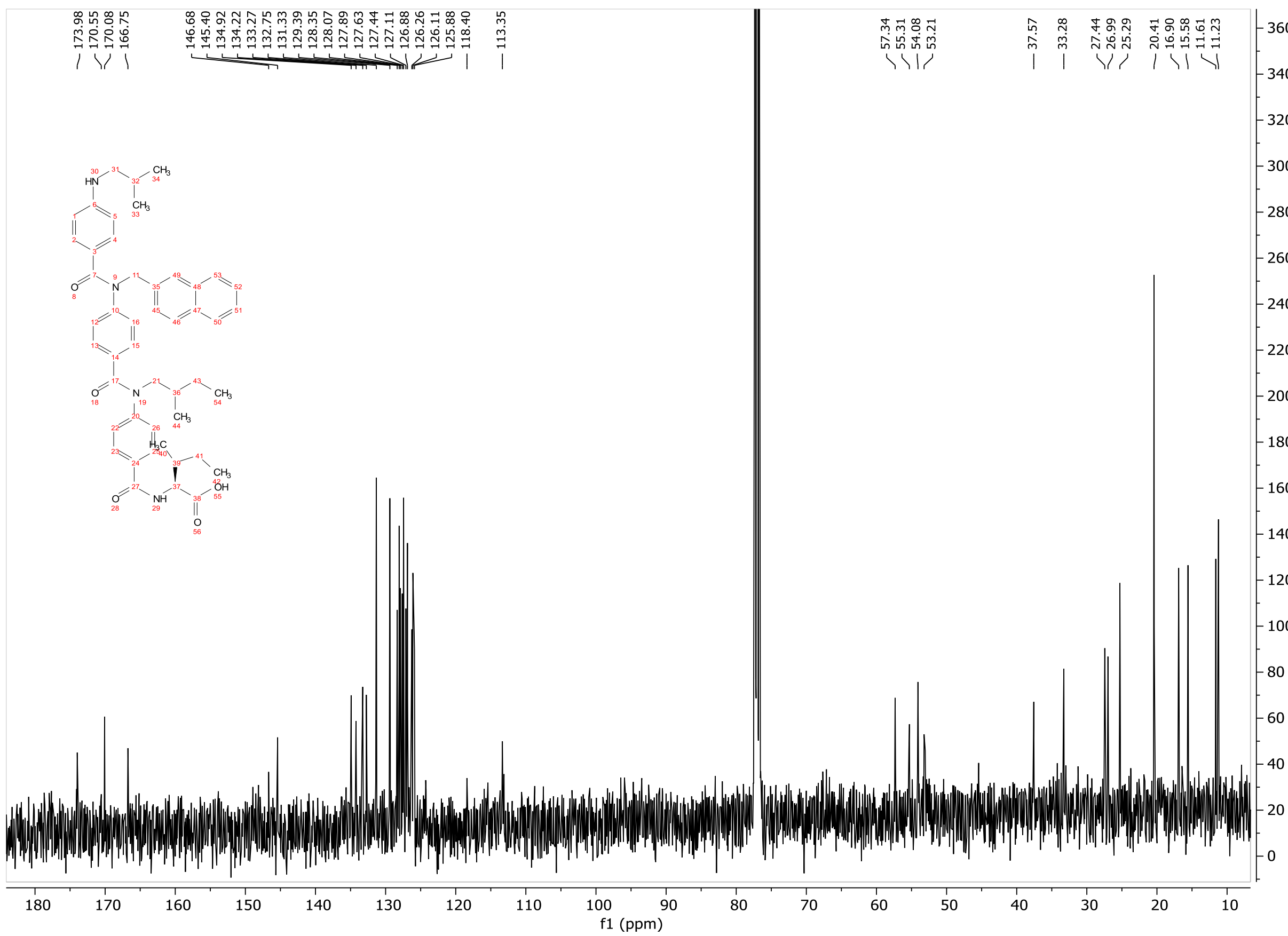

^{1}H NMR of **OB-a1** at rt in $CDCl_3$

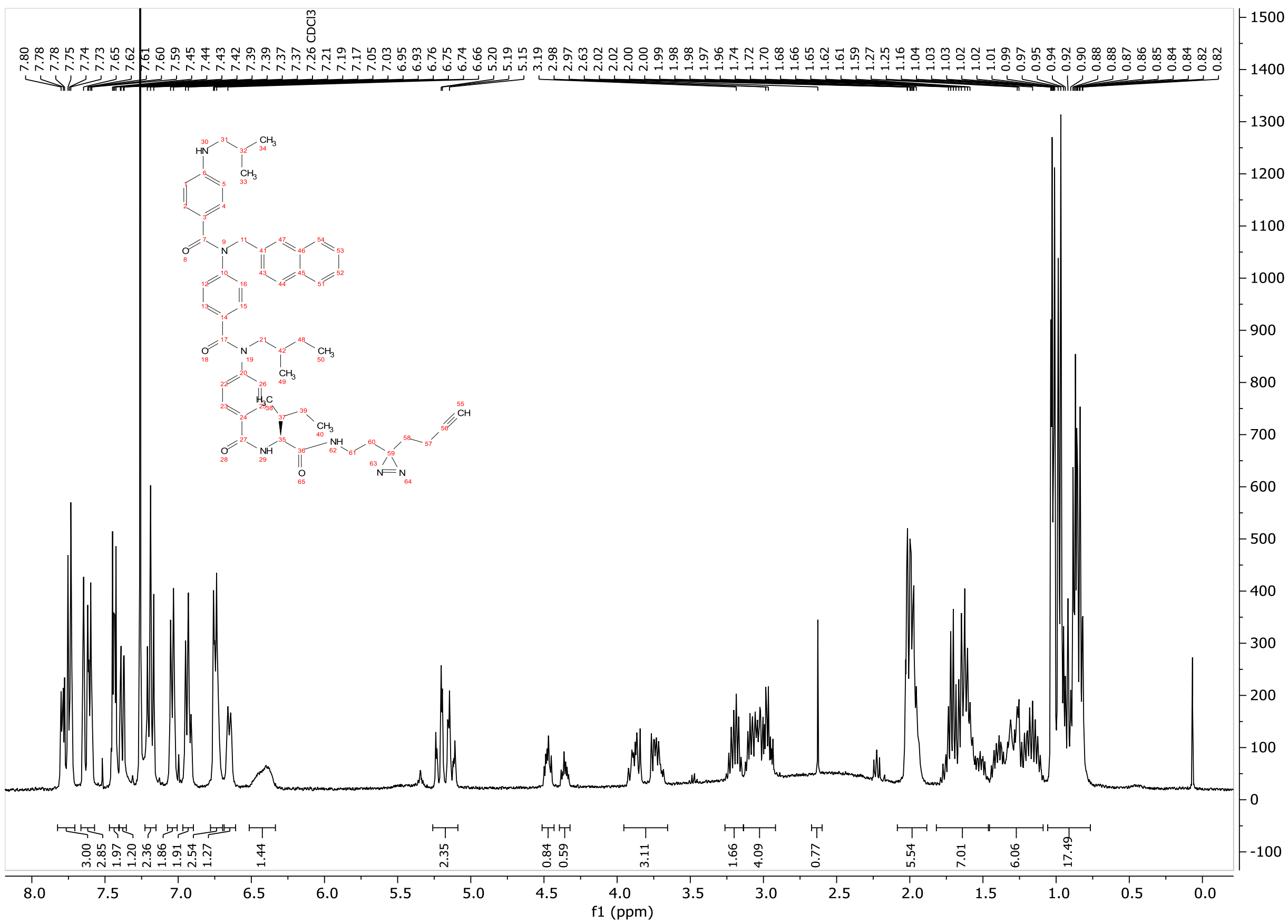

^{13}C NMR of **OB-a1** at rt in $CDCl_3$

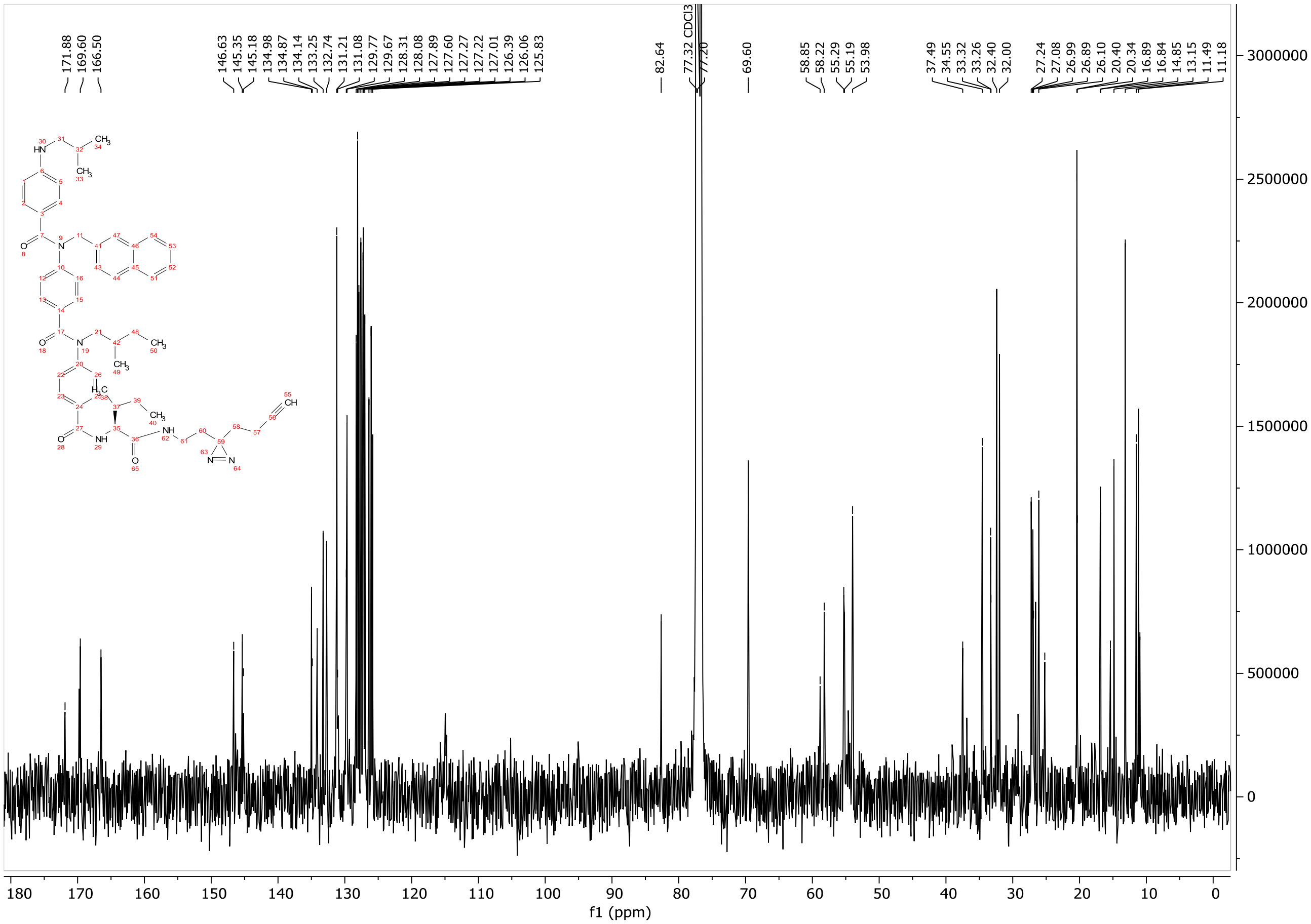

^1H NMR of **OB-a2** at 298 K in $CDCl_3$

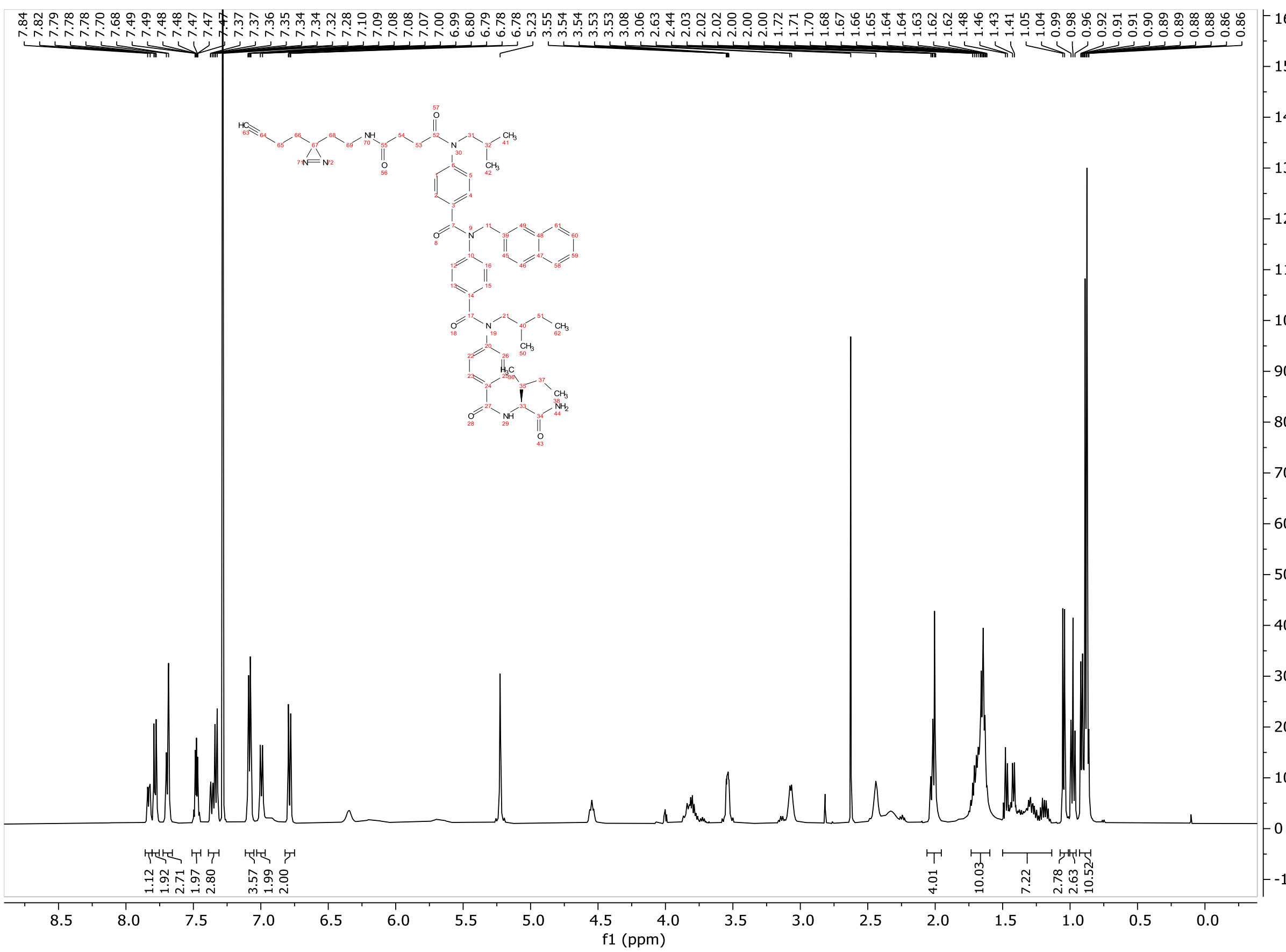

^{13}C NMR of **OB-a2** at rt in $CDCl_3$

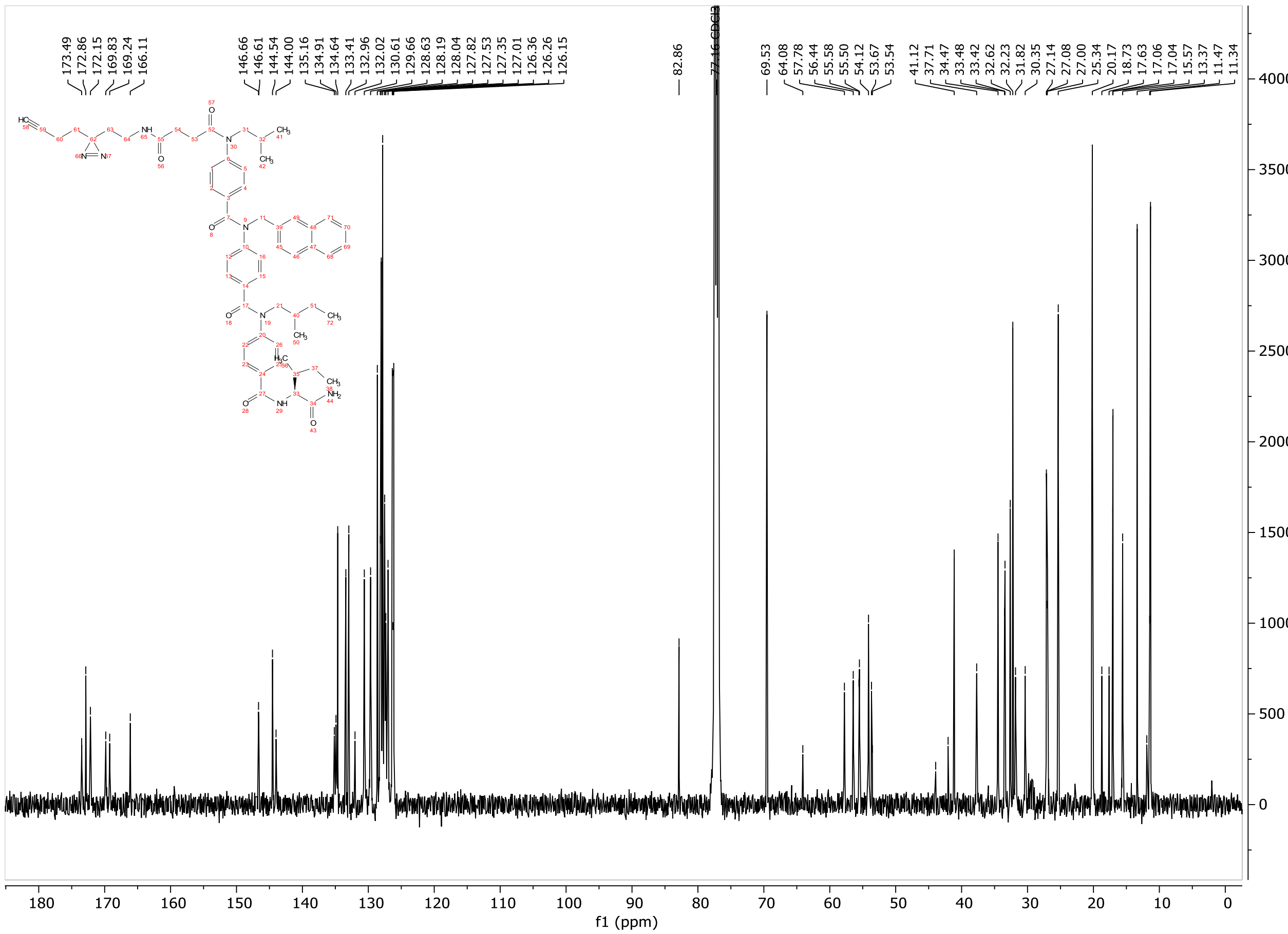

^{1}H NMR of **OB-a3** at 328 K in $CDCl_3$

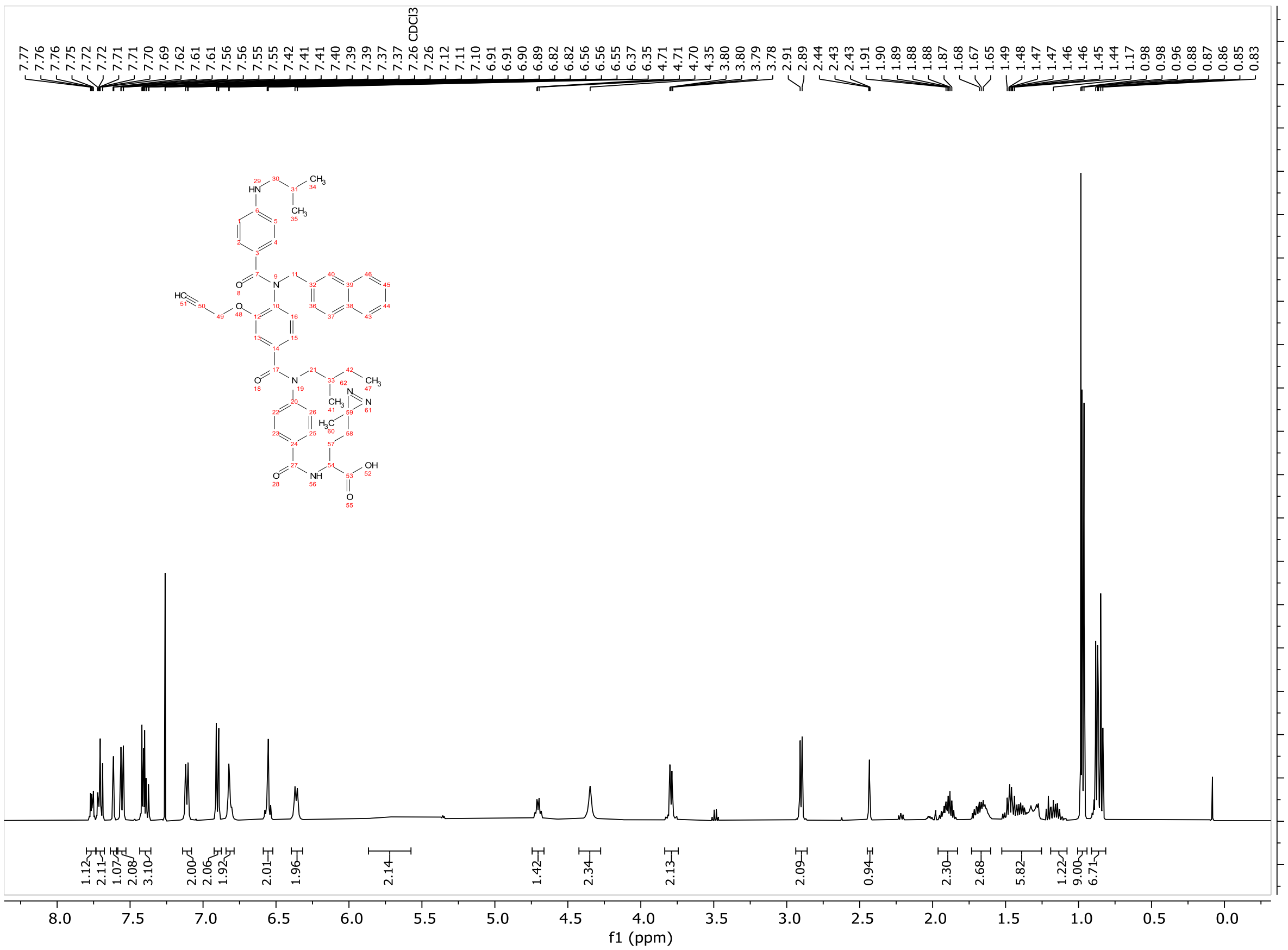

^{13}C NMR of **OB-a3** at rt in $CDCl_3$

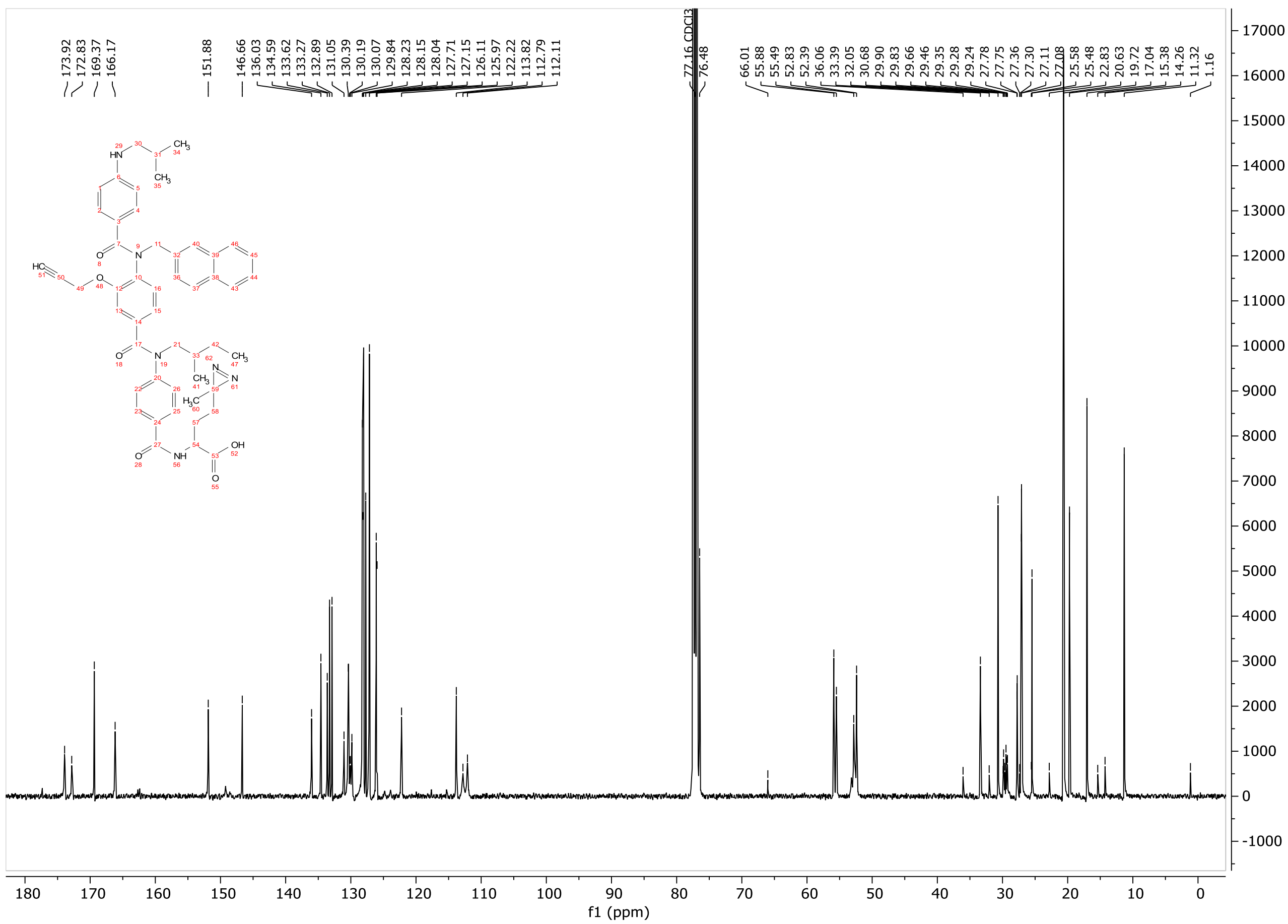

^{1}H NMR of **OB-b** at rt in MeOD

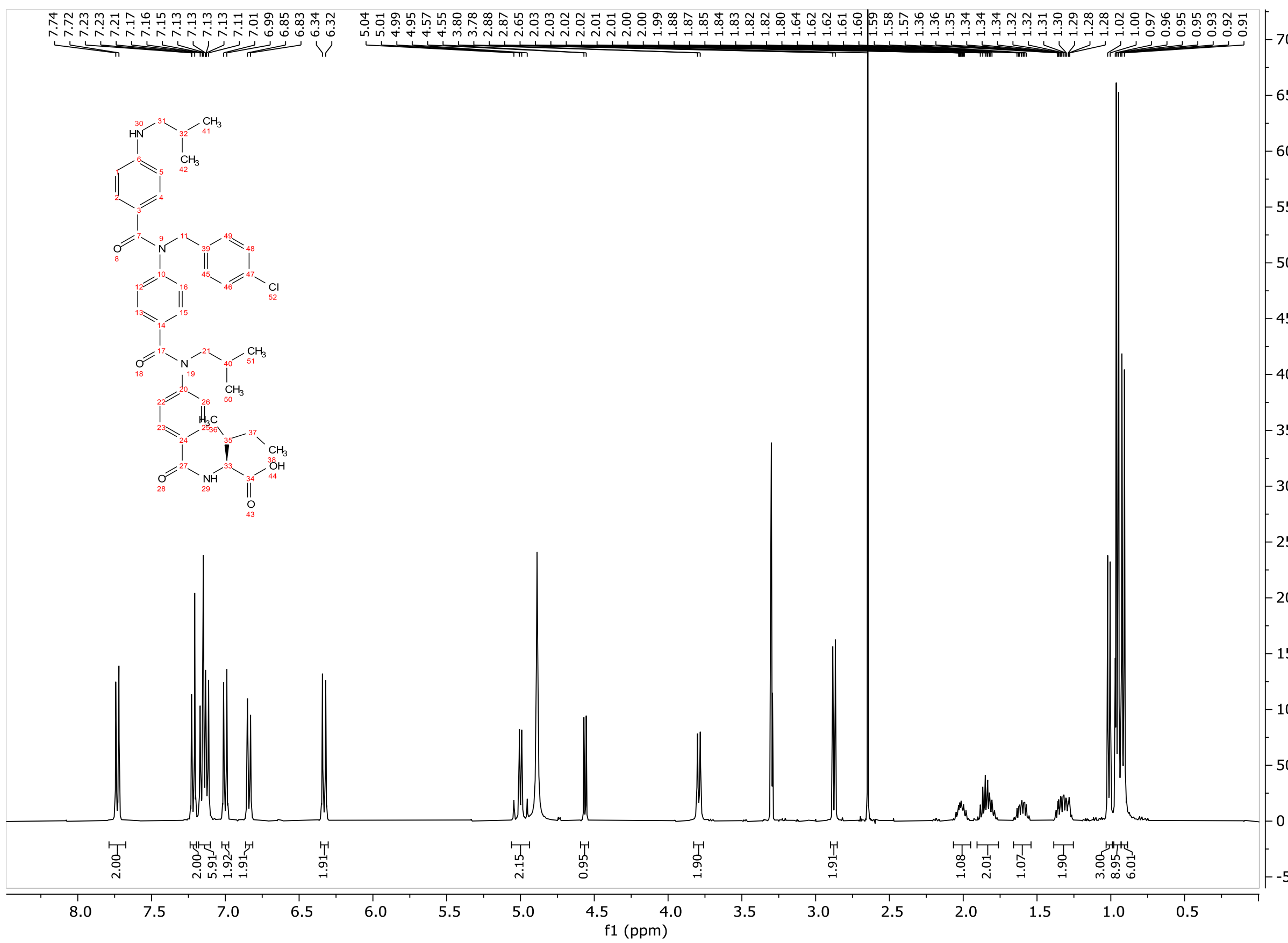

^{13}C NMR of **OB-b** at rt in MeOD

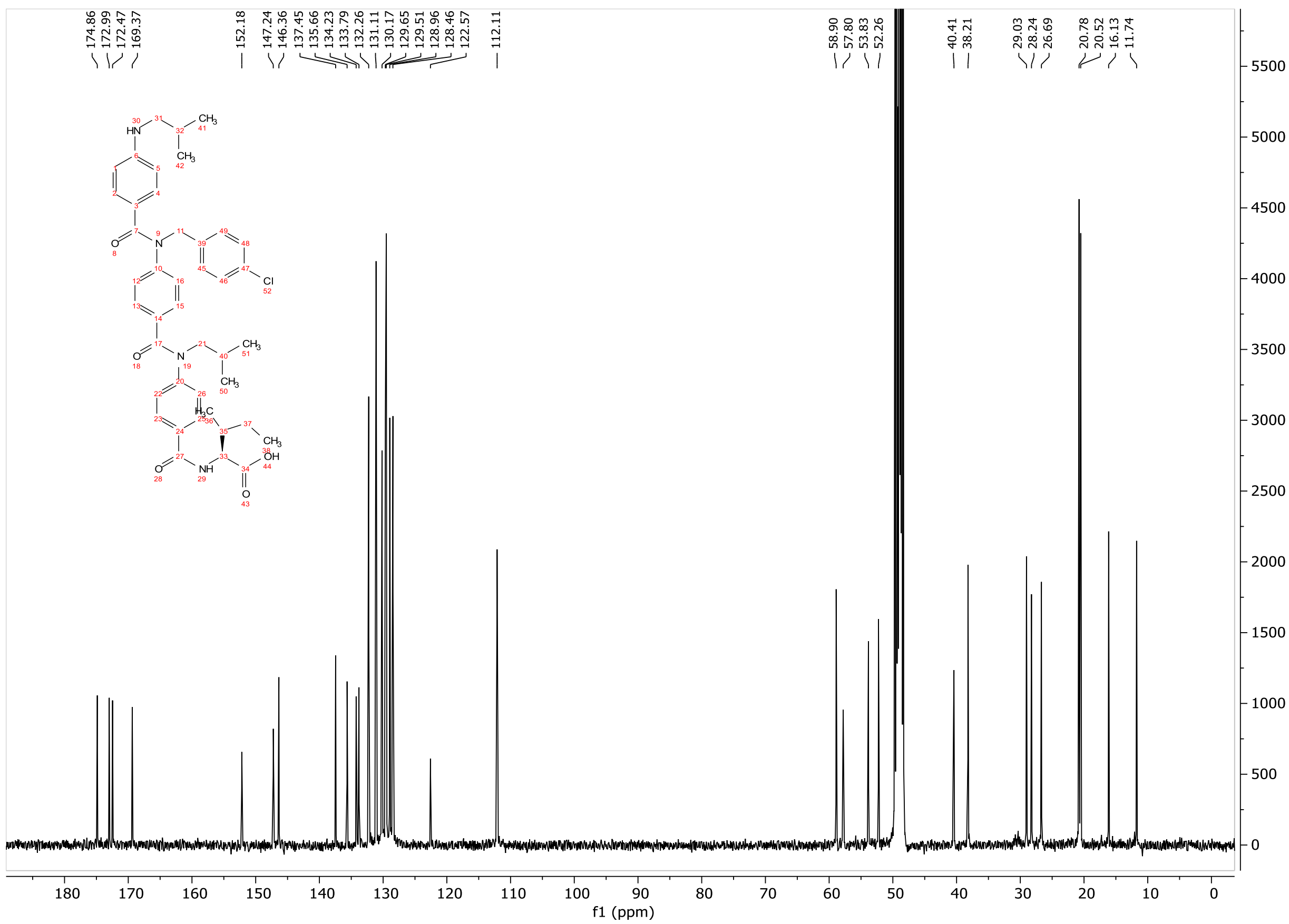

^{1}H NMR of **OB-b1** at 328 K in $CDCl_3$

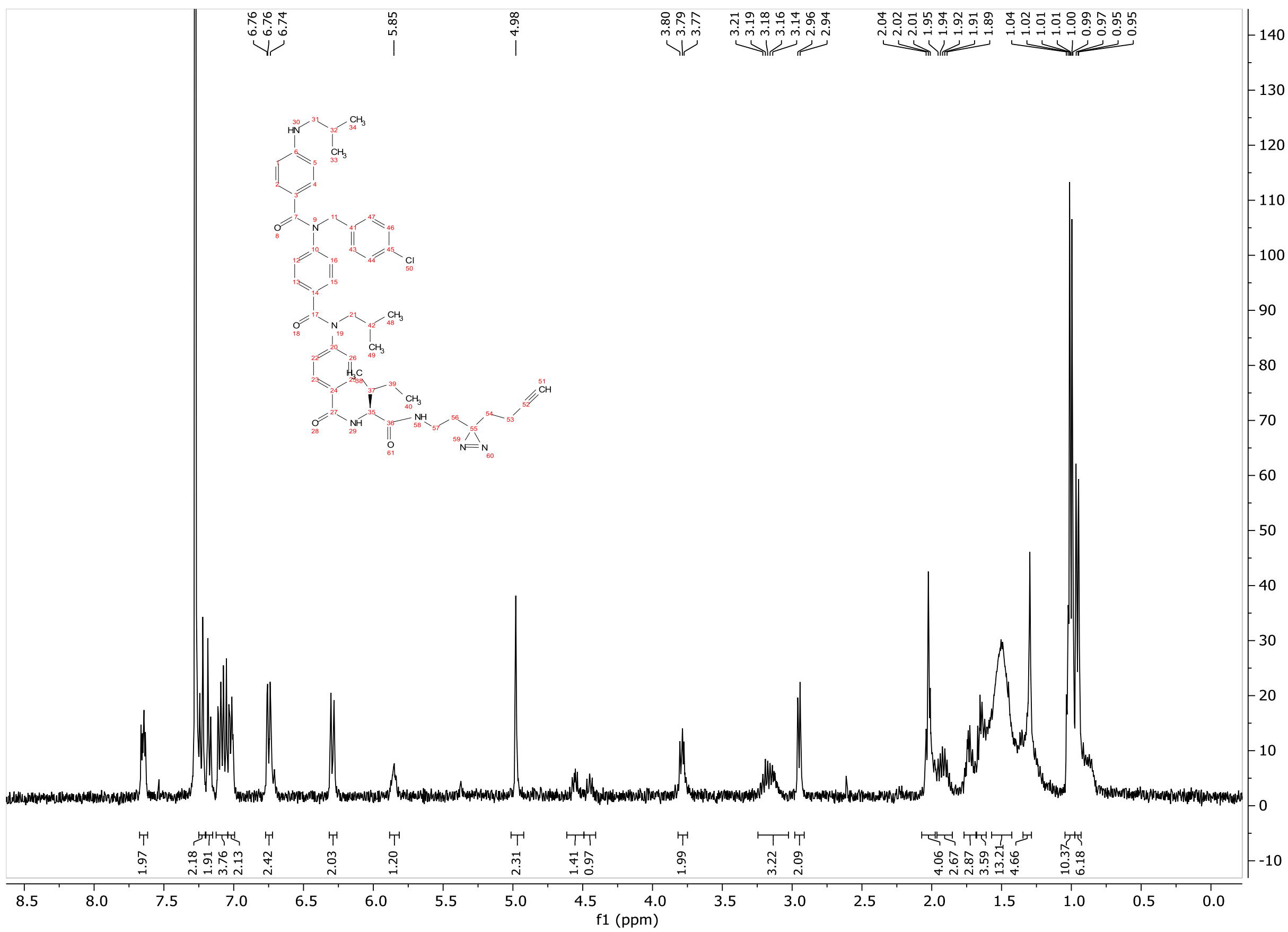

^{13}C NMR of **OB-b1** at rt in $CDCl_3$

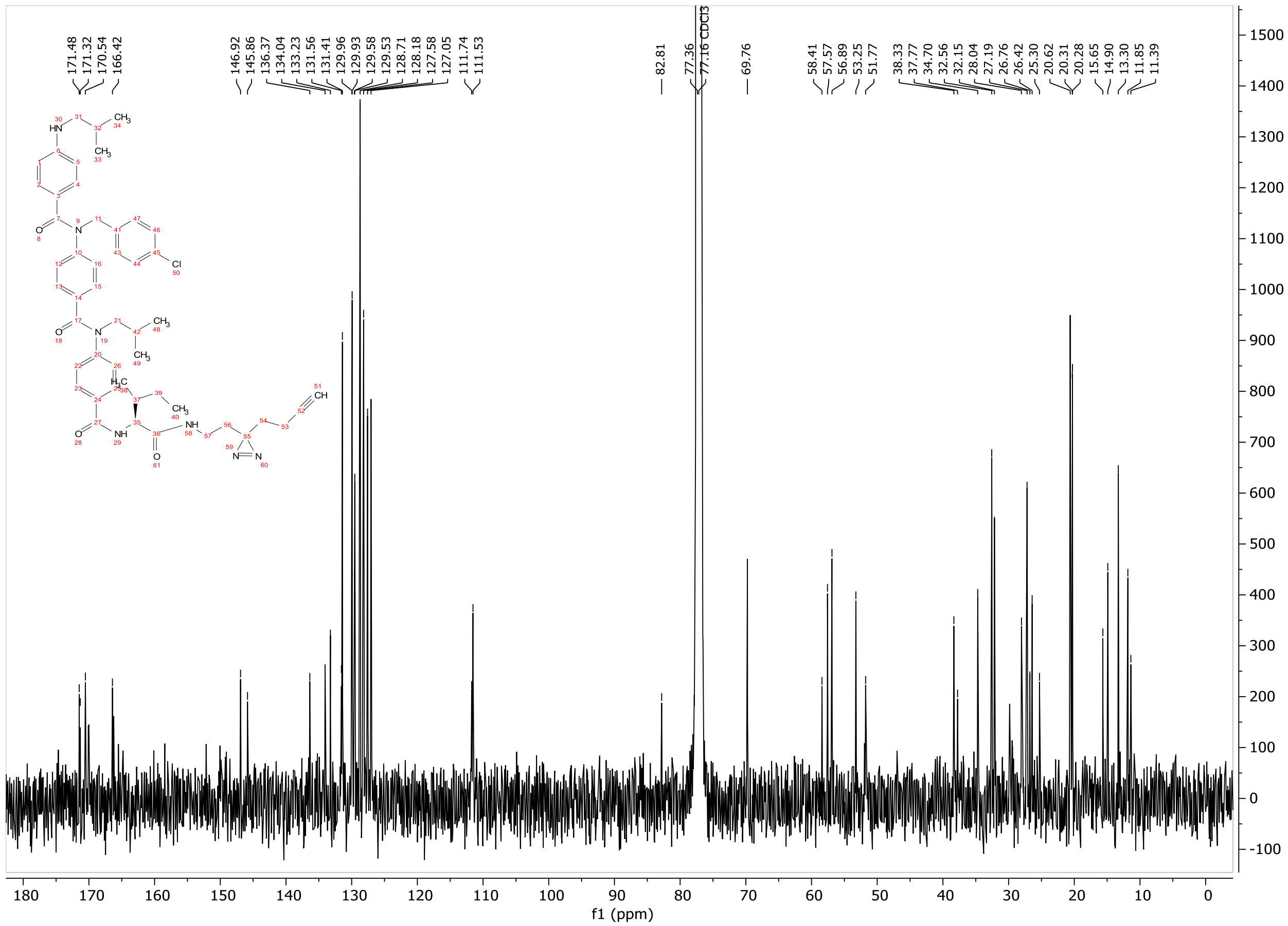

^{1}H NMR of **OB-b2** at 328 K in $CDCl_3$

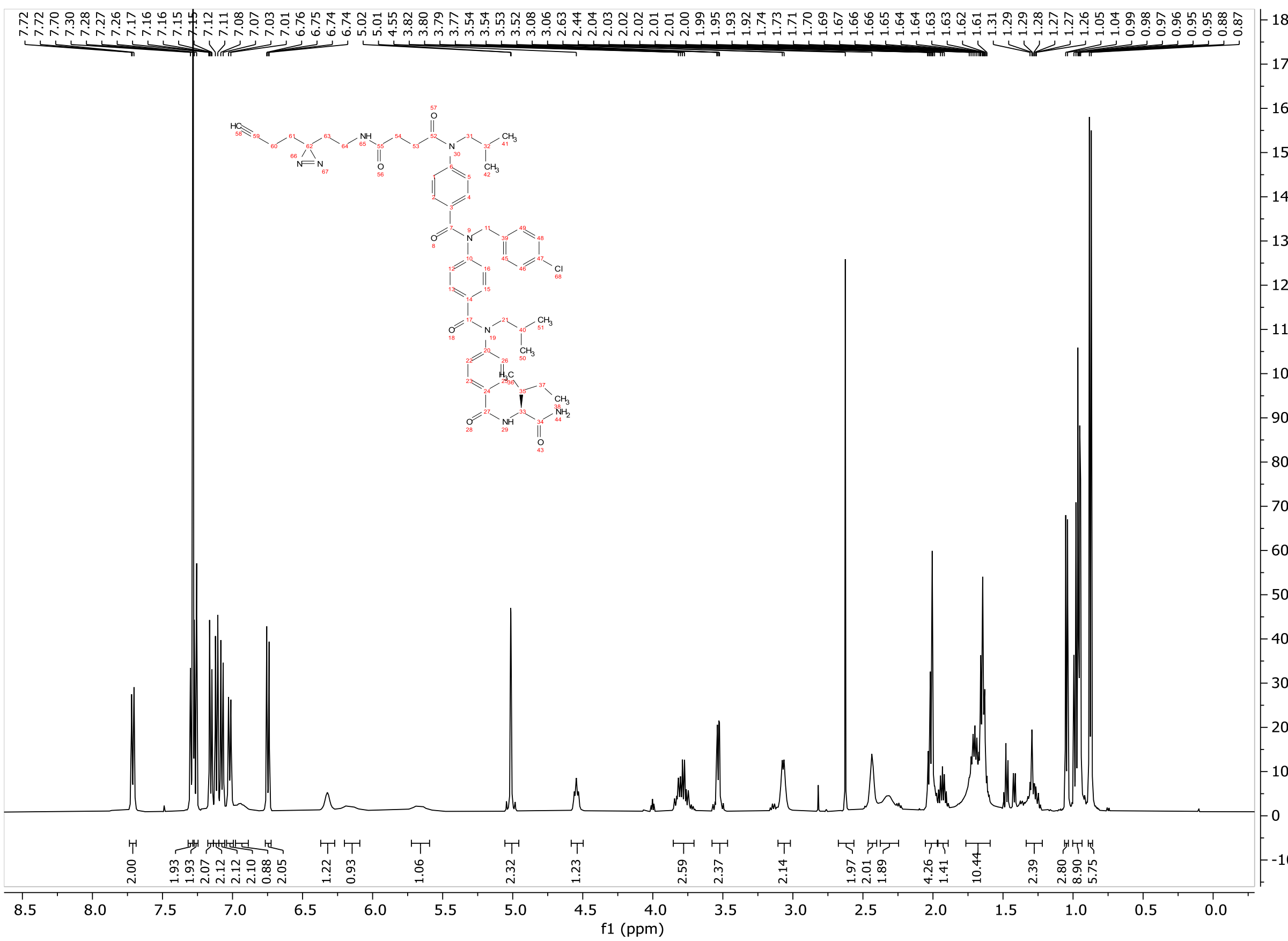

^{13}C NMR of **OB-b2** at rt in $CDCl_3$

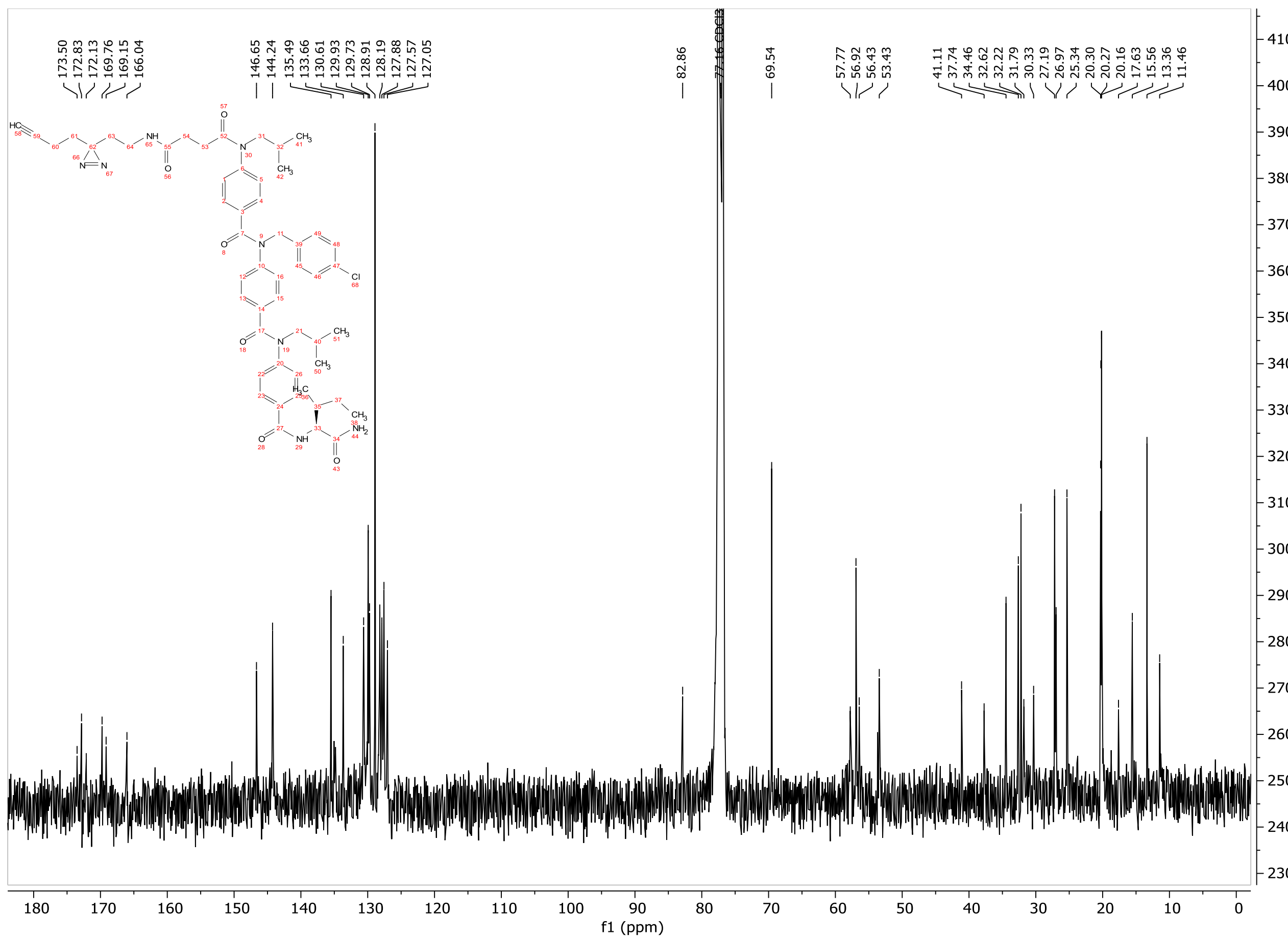

^{1}H NMR of **OB-b3** at 328 K in $CDCl_3$

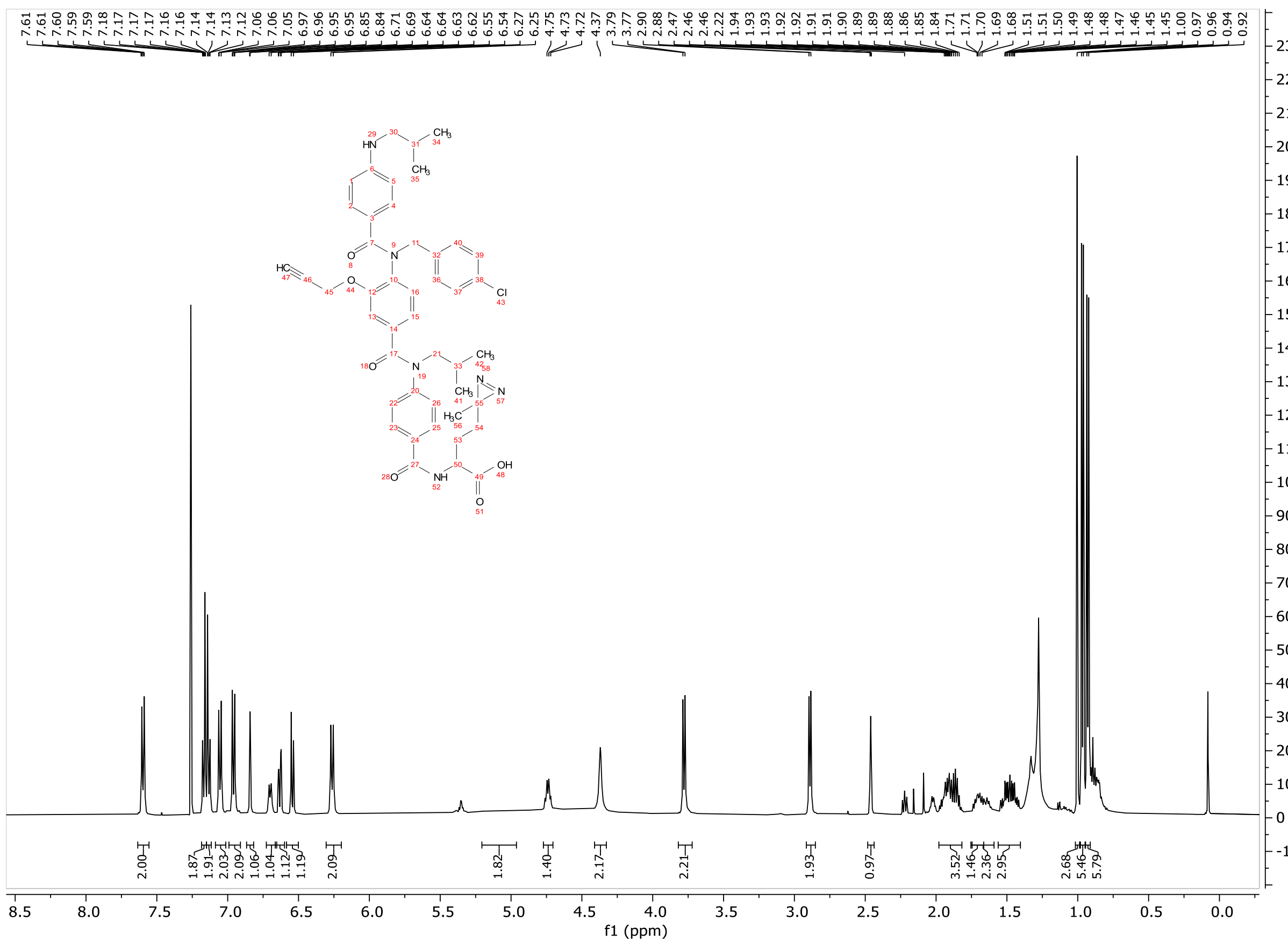

^{13}C NMR of **OB-b3** at rt in $CDCl_3$

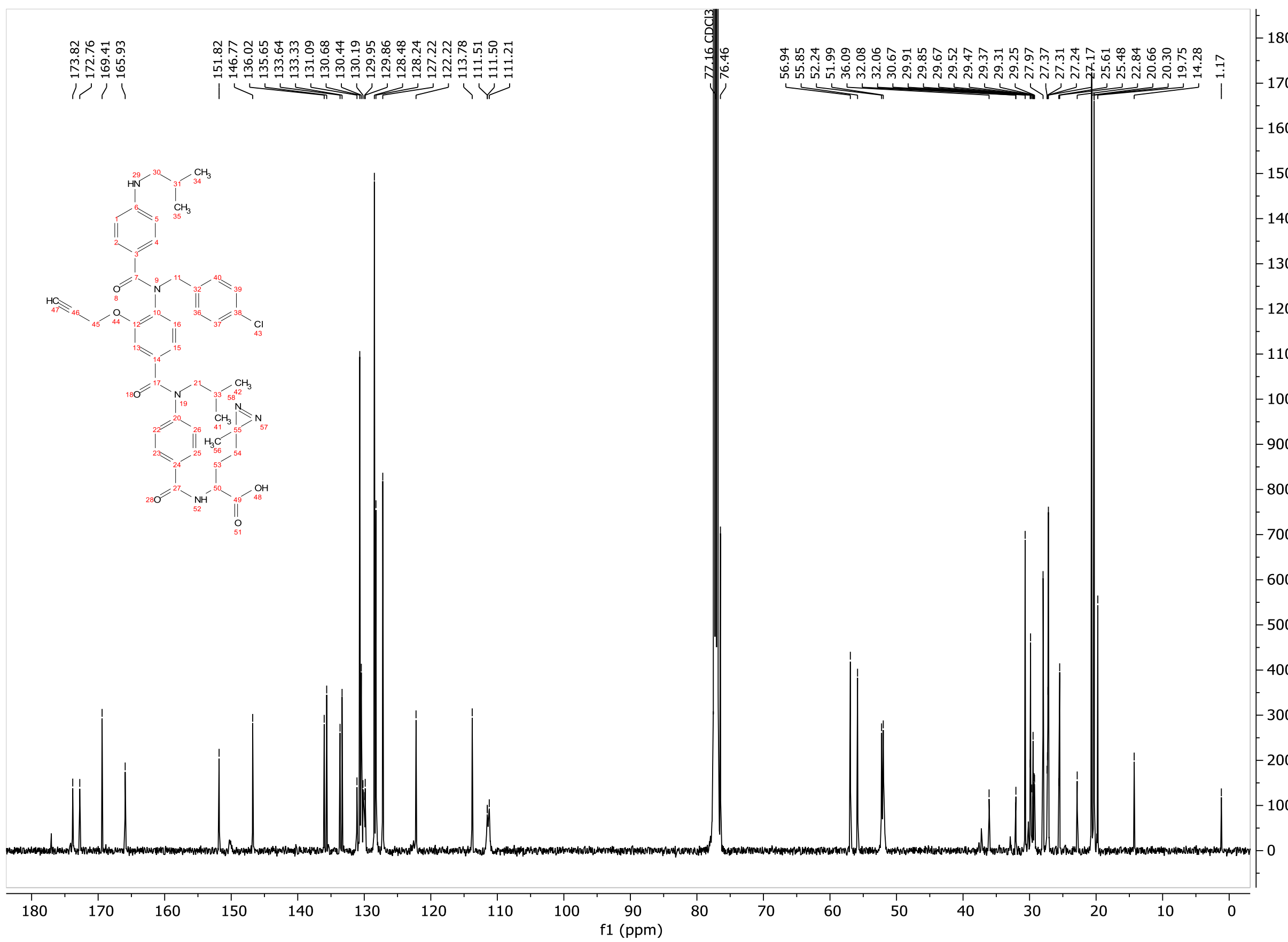

*¹H NMR of **PP** at rt in $CDCl_3$*

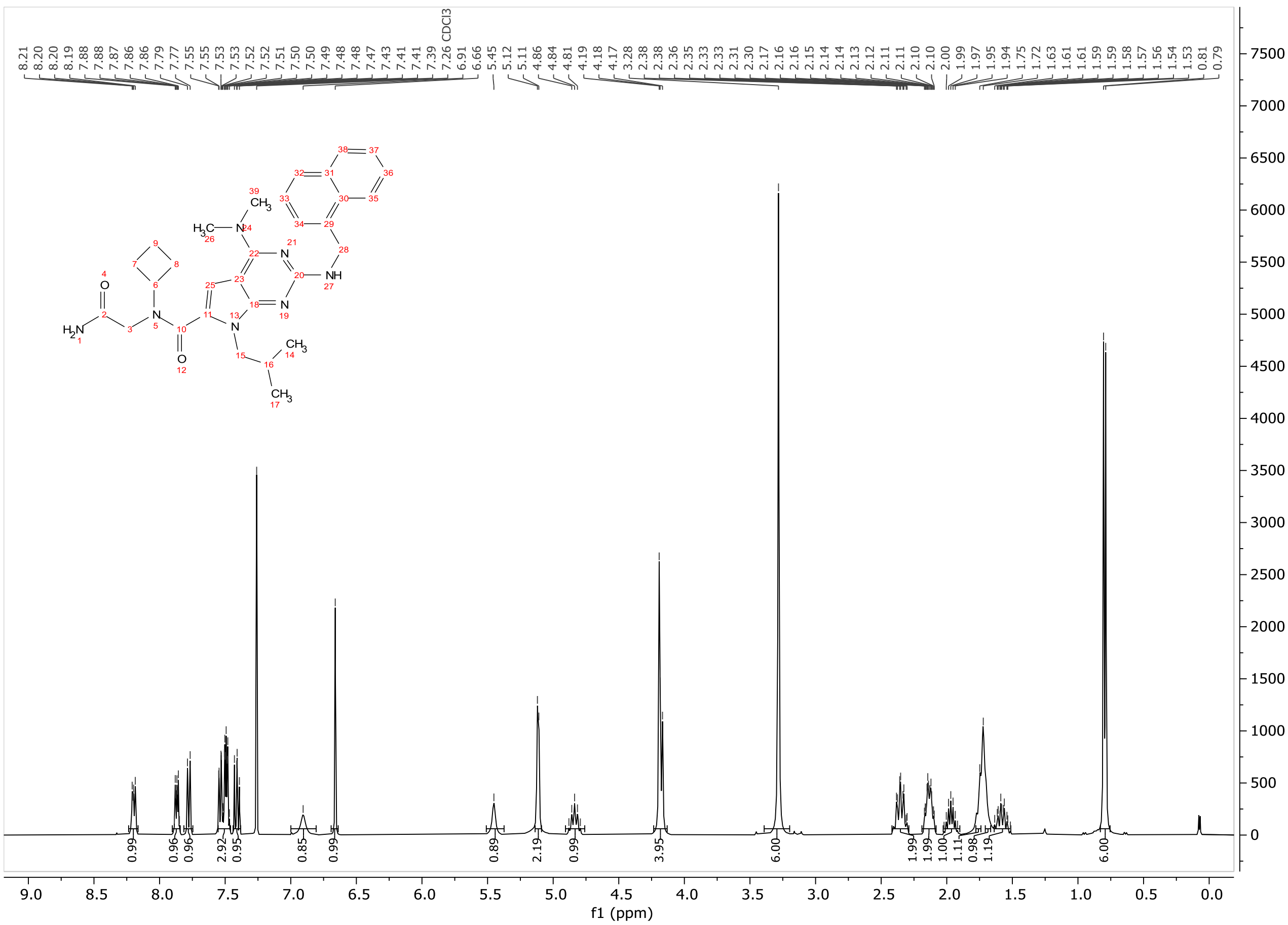

^{13}C NMR of ***PP*** *at rt in* $CDCl_3$

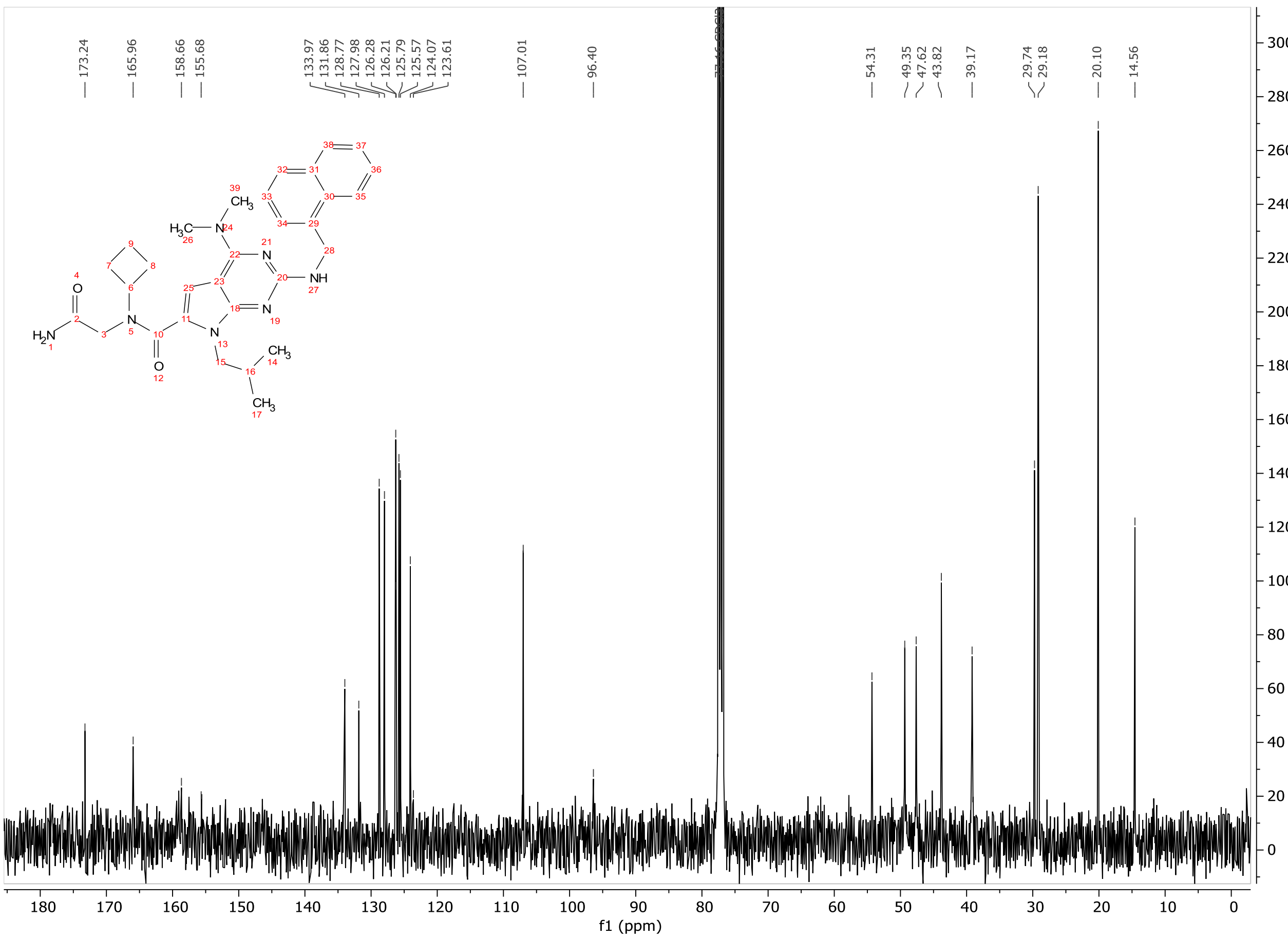

^{1}H NMR of **PP-1** at rt in $CDCl_3$

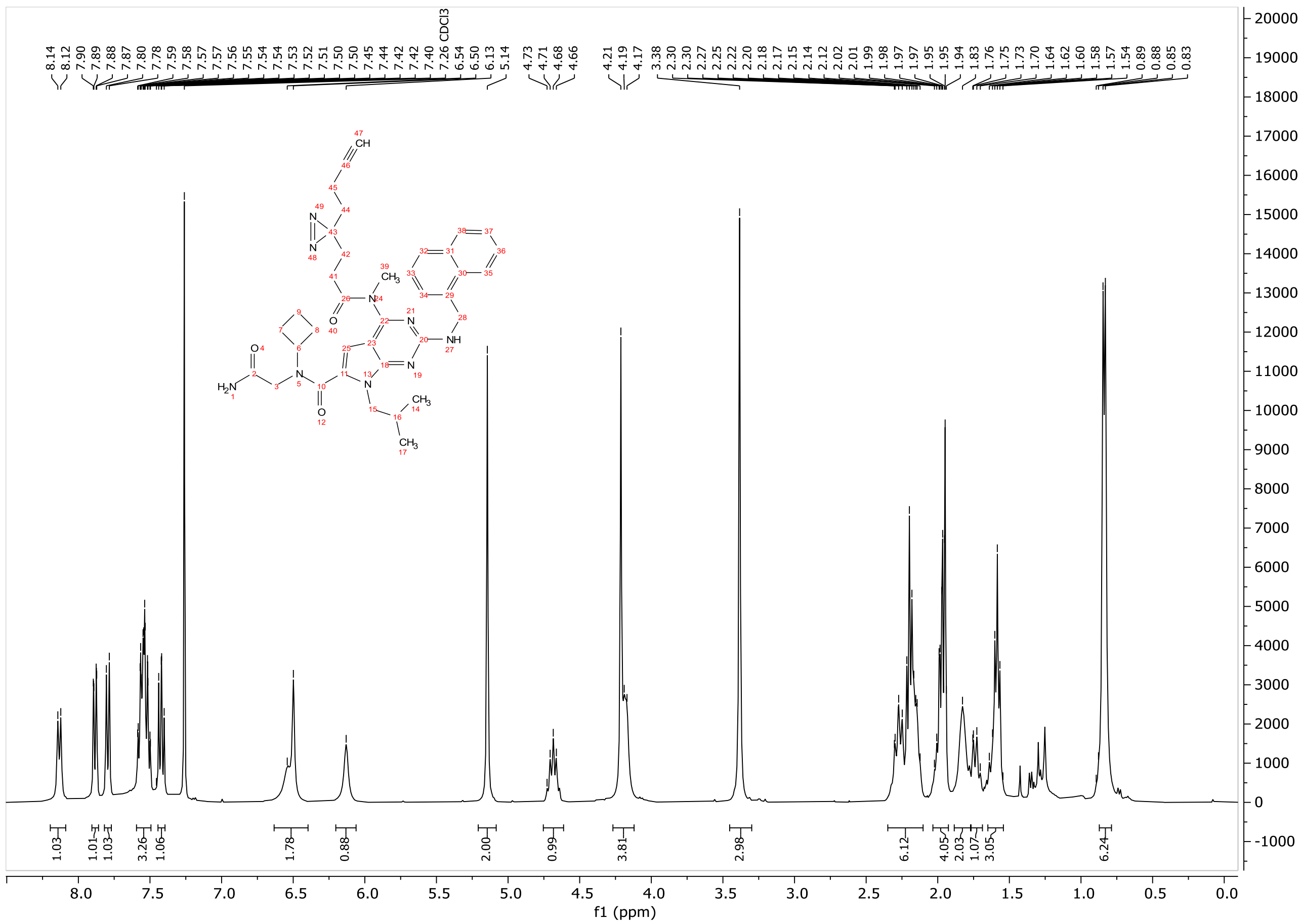

*^{13}C NMR of **PP-1** at rt in $CDCl_3$*

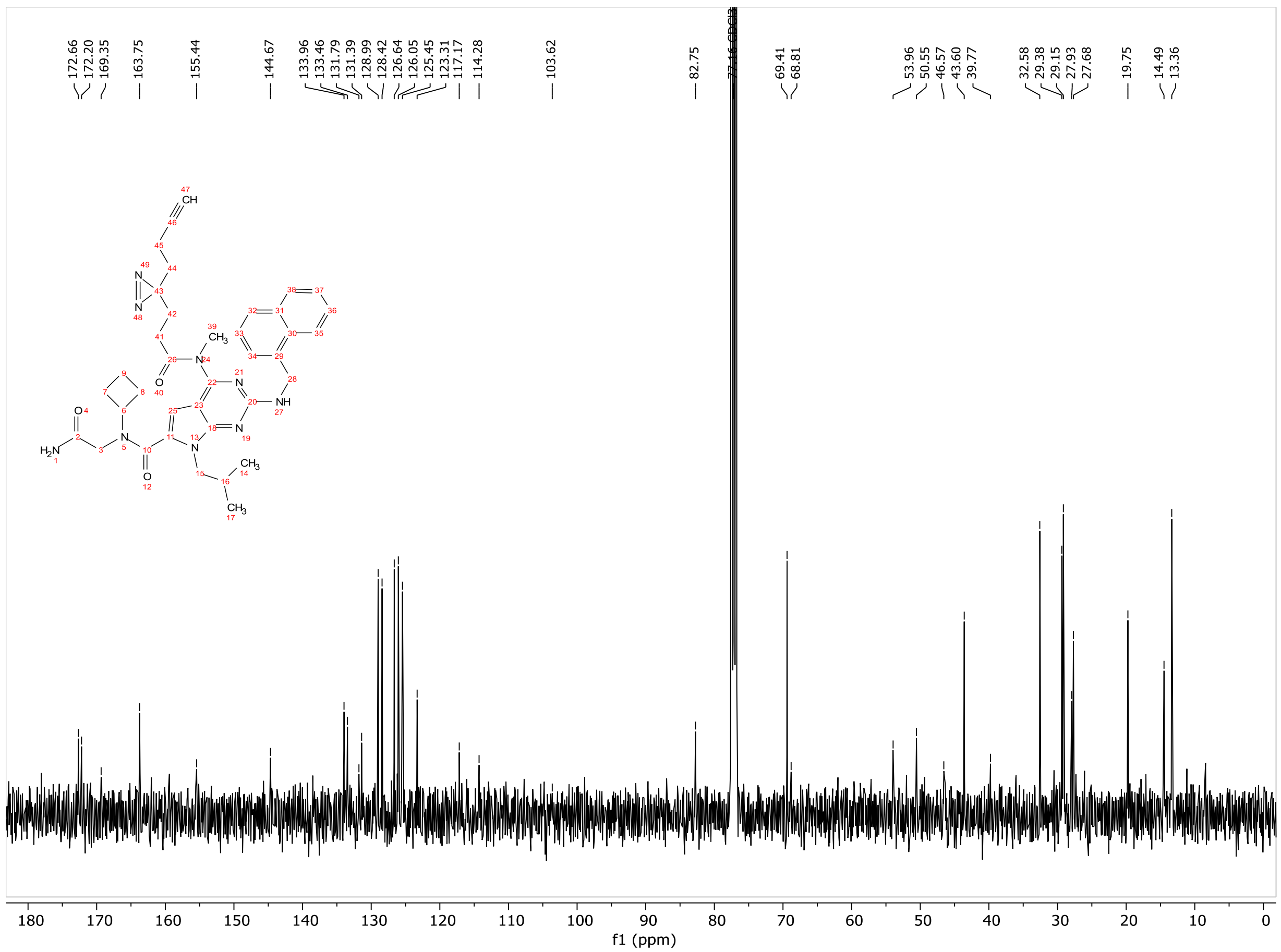

^{1}H NMR of ***PP-2*** *at 373 K in DMSO-*d_6

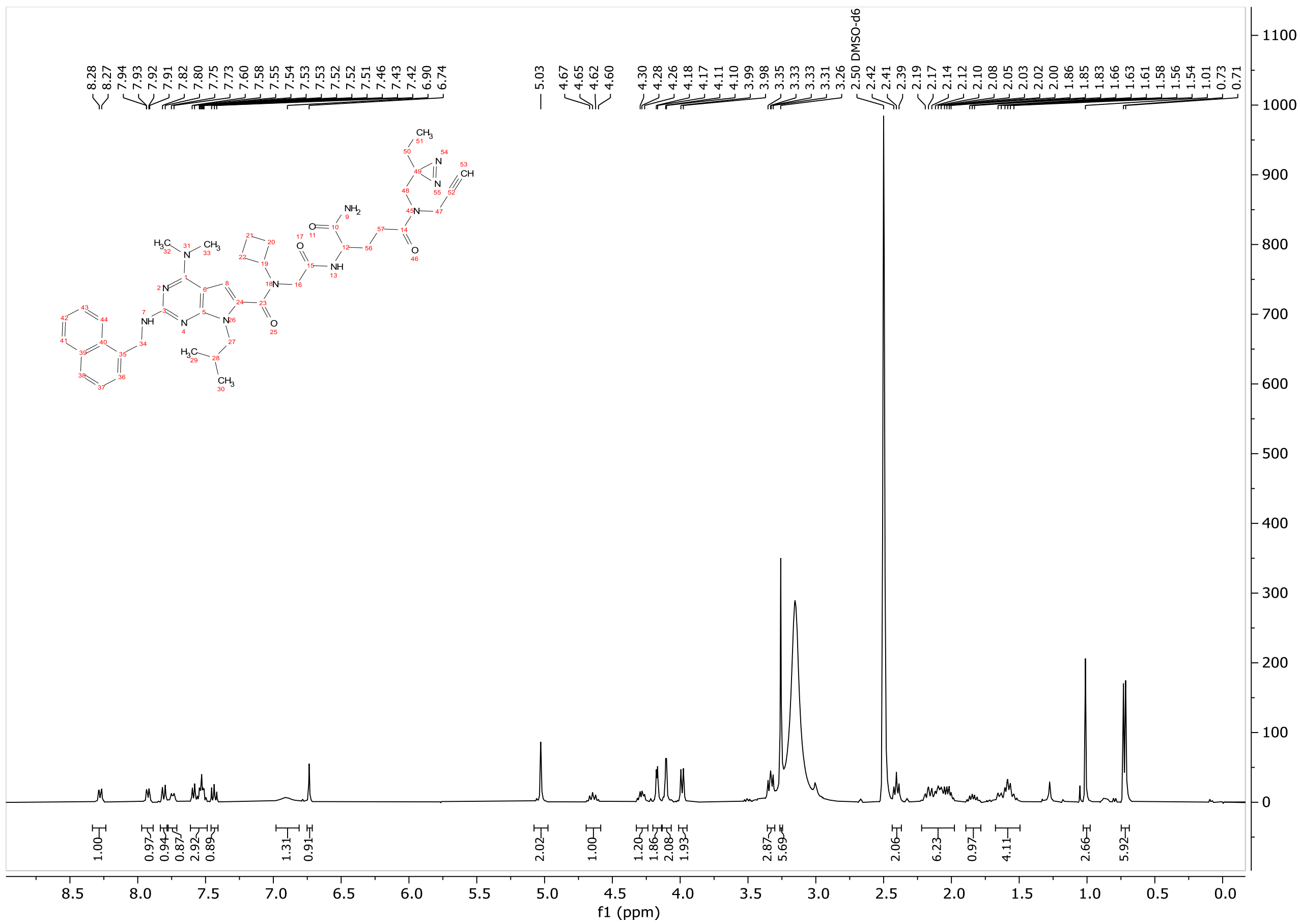

*^{1}H NMR of **OP** at rt in DMSO-d6*

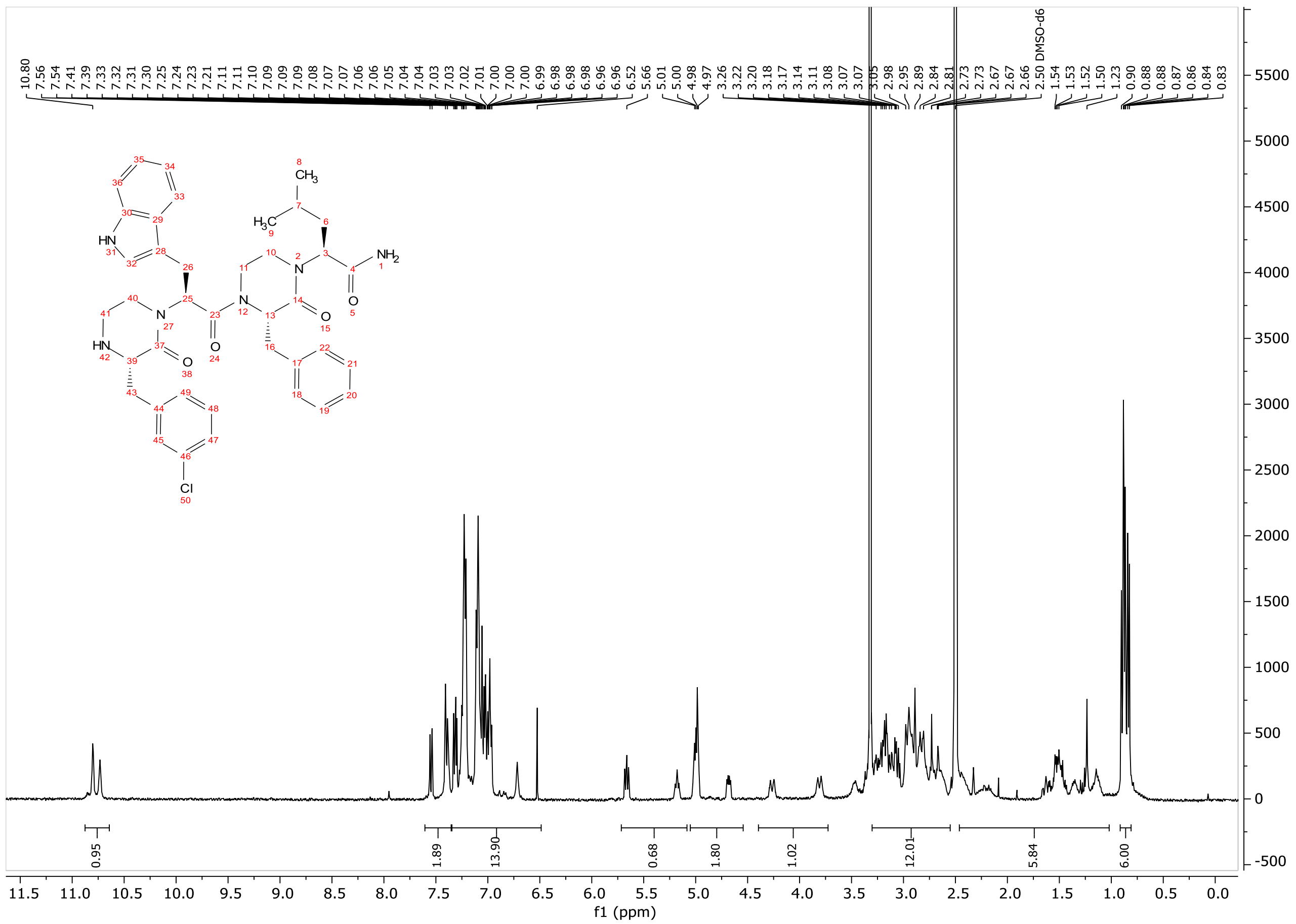

^{13}C NMR of **OP** at rt in DMSO-d_6

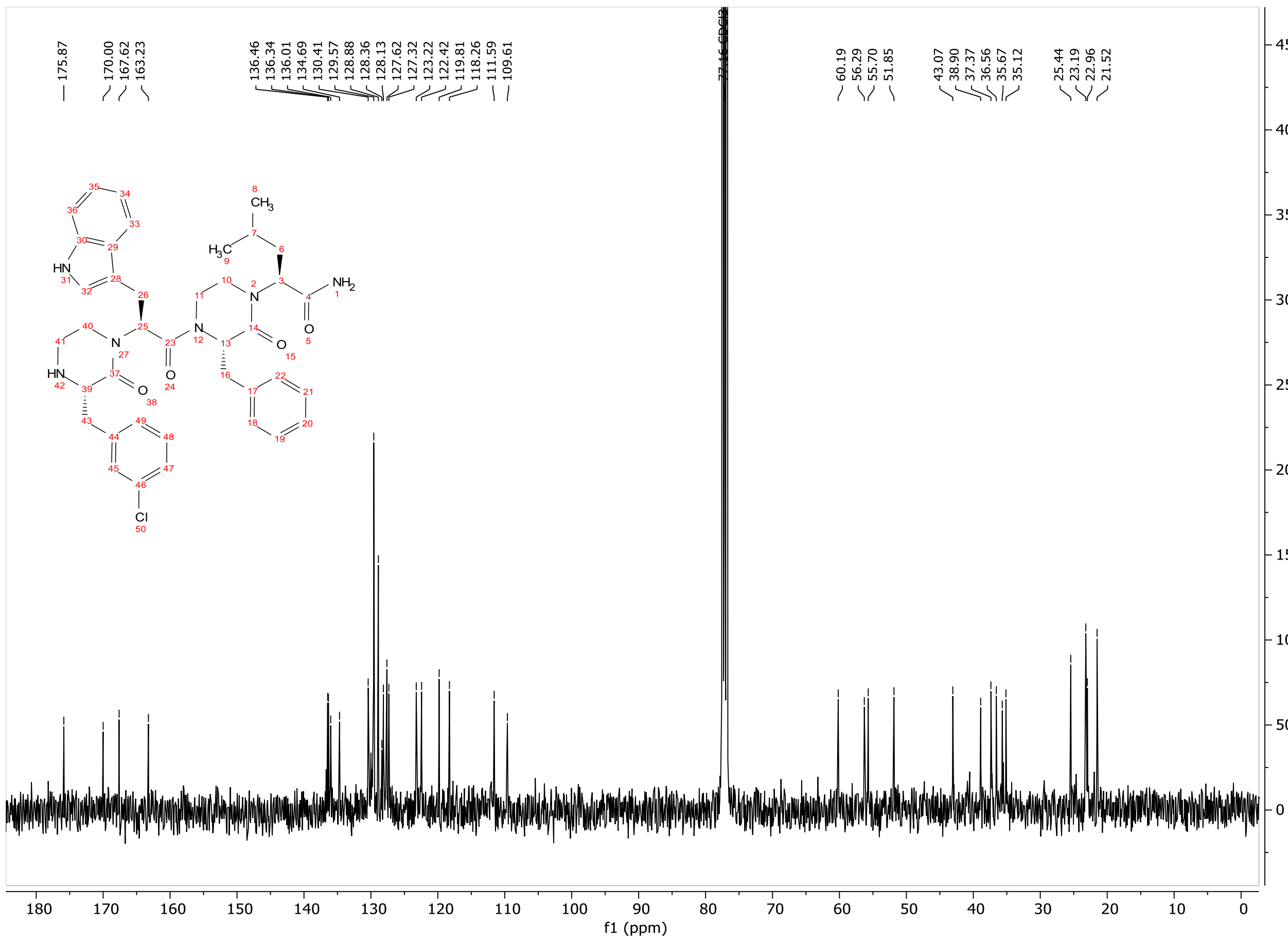

*^{1}H NMR of **OP-1** at rt in DMSO-d$_6$*

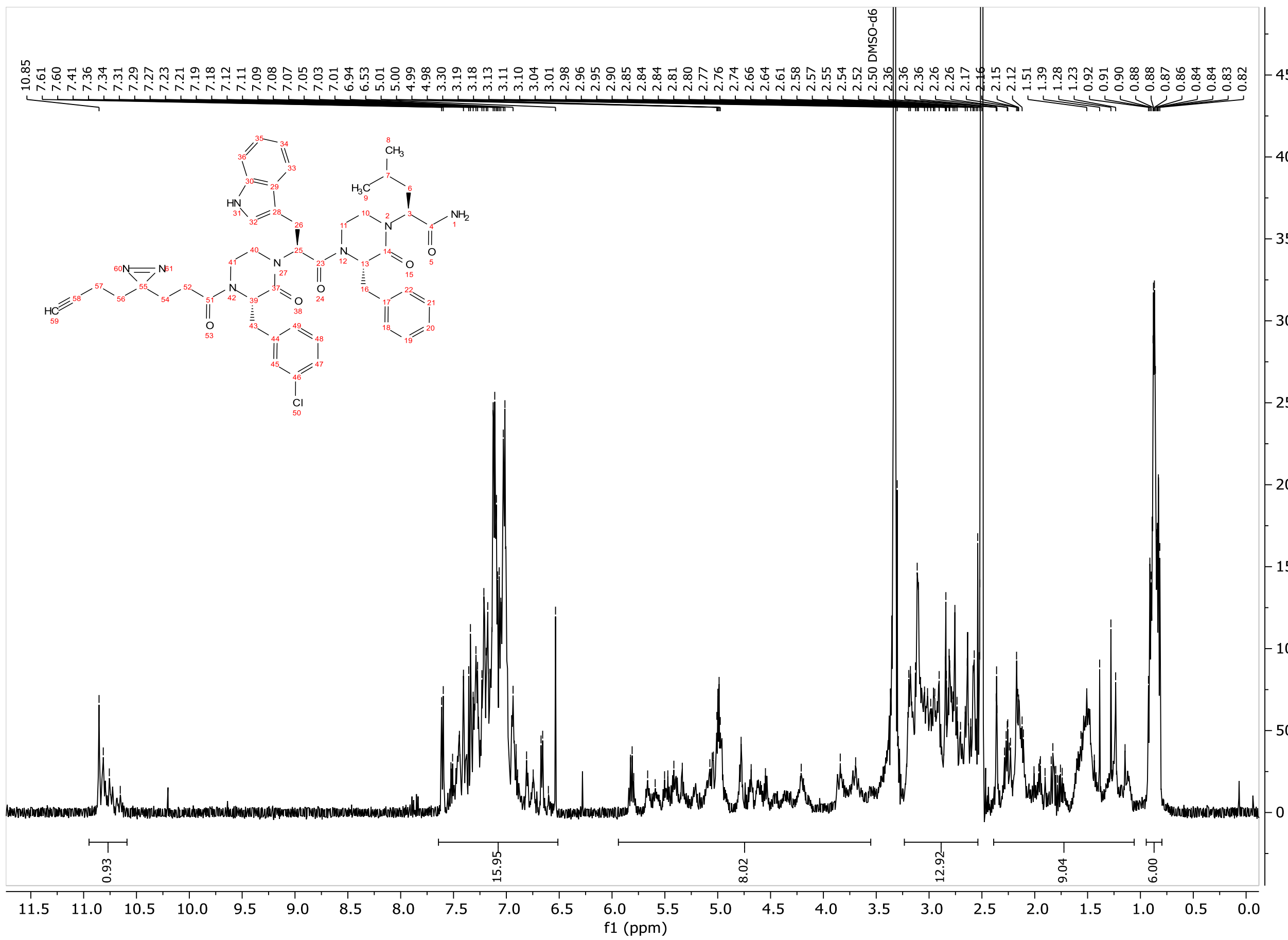

*^{1}H NMR of **OP-2** at rt in DMSO-d6*

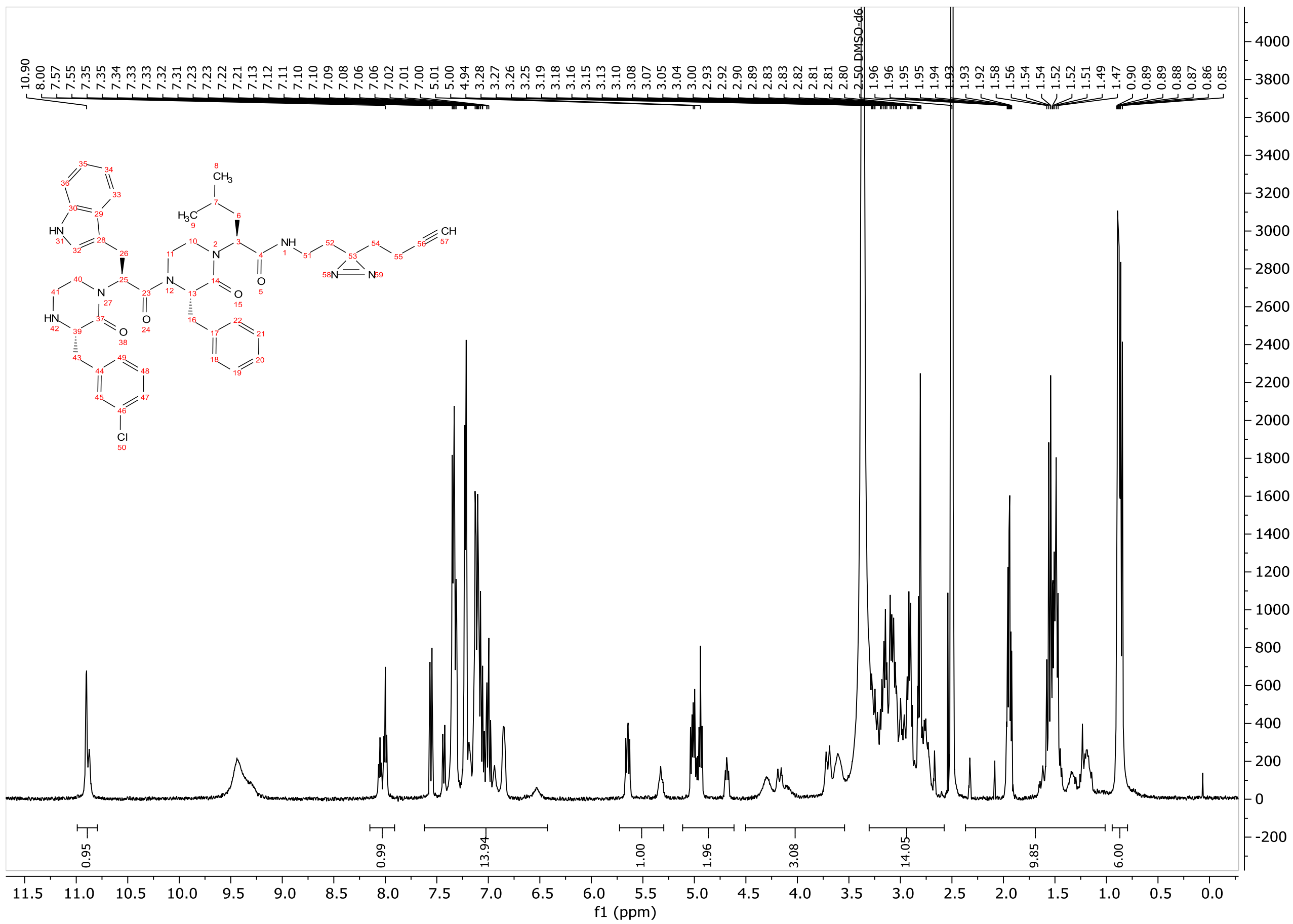

*^{1}H NMR of **NP** at rt in $CDCl_3$*

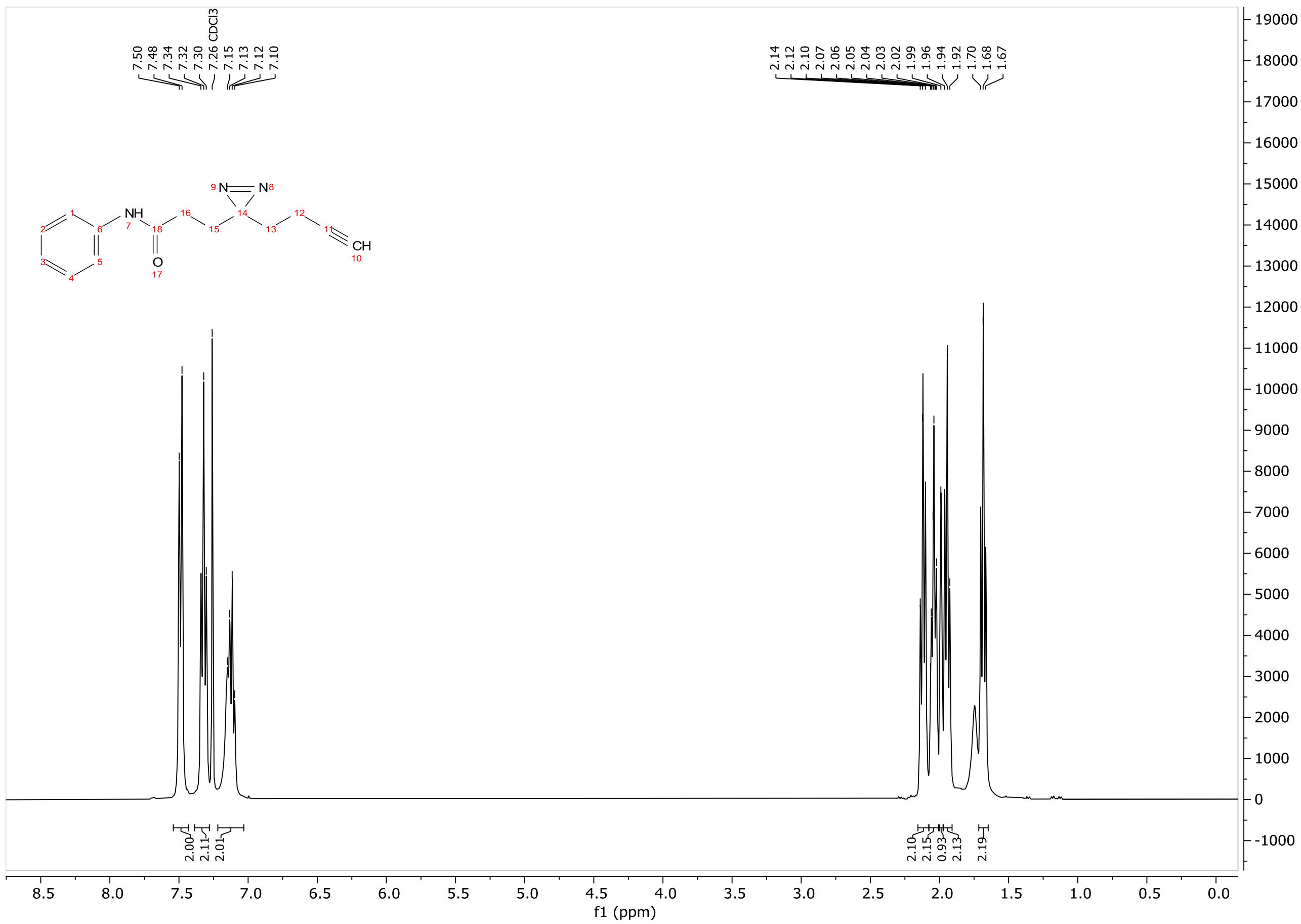

^{13}C NMR of **NP** at rt in $CDCl_3$

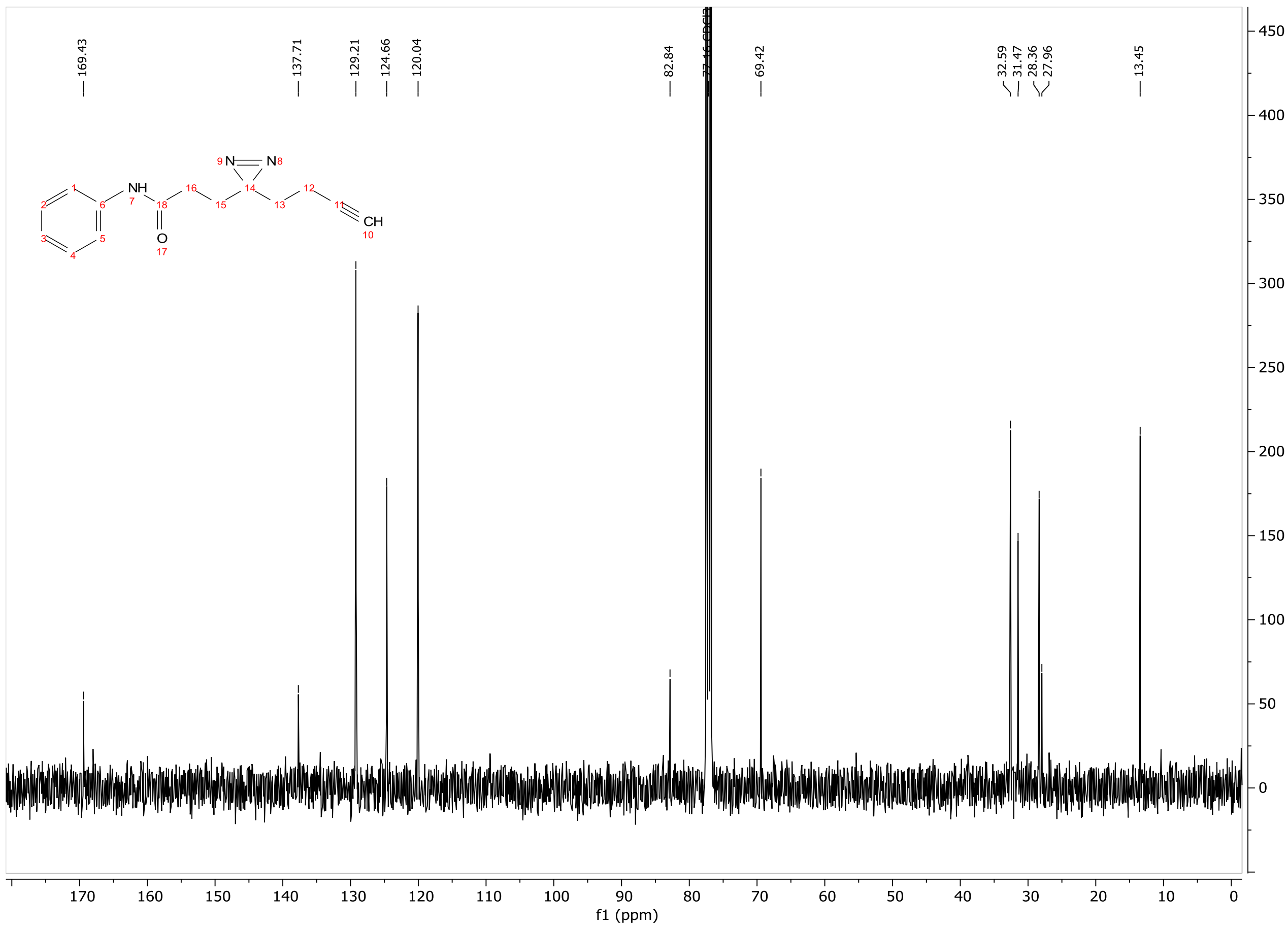

Analytical HPLC traces for final products

*HPLC trace of **OB-a***

<Chromatogram>

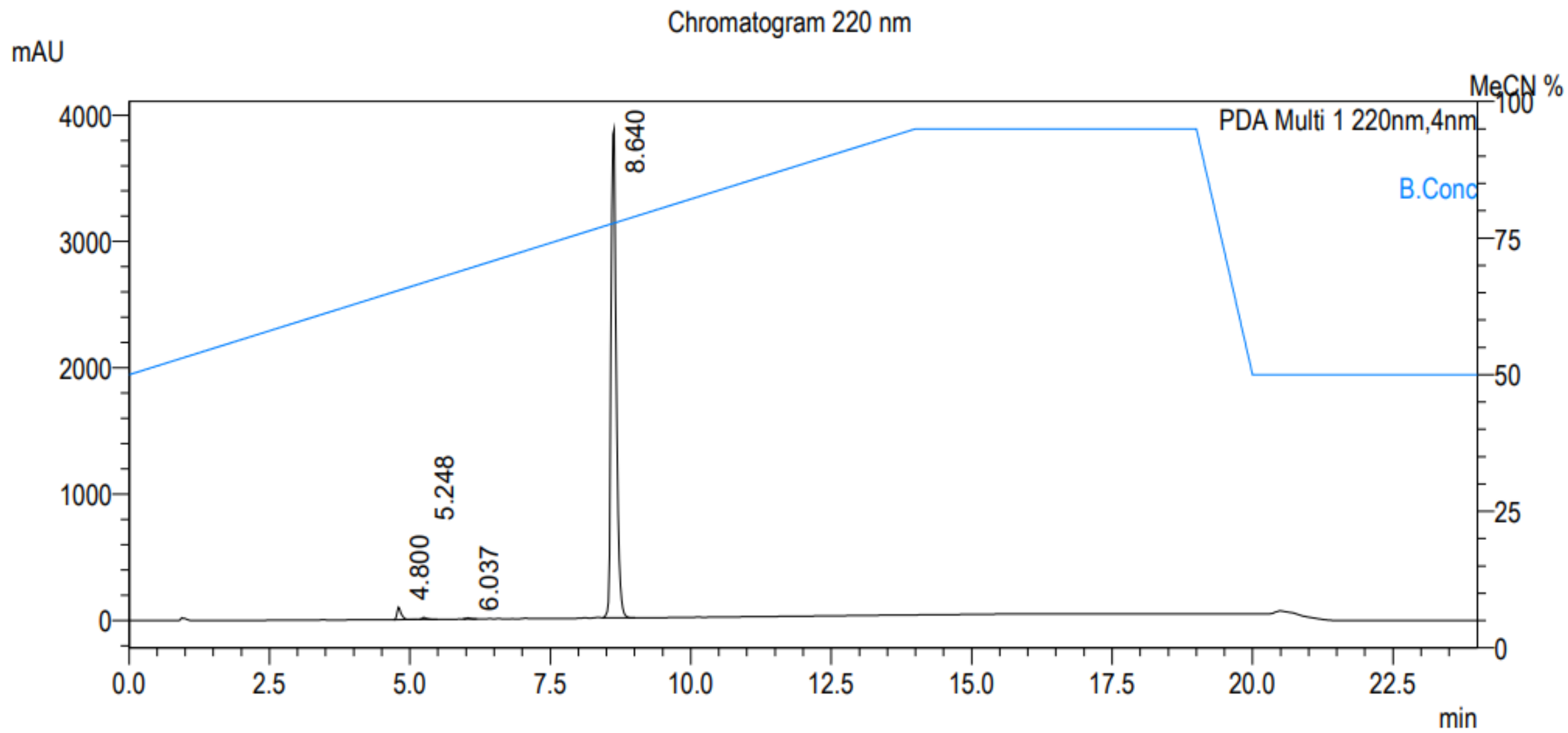

<Peak Table>

Peak Table

PDA Ch1 220nm

Peak#	Ret. Time	Area	Area%
1	4.800	495898	1.923
2	5.248	88933	0.345
3	6.037	52231	0.203
4	8.640	25145934	97.529
Total		25782995	100.000

HPLC trace of ***OB-a1***

<Chromatogram>

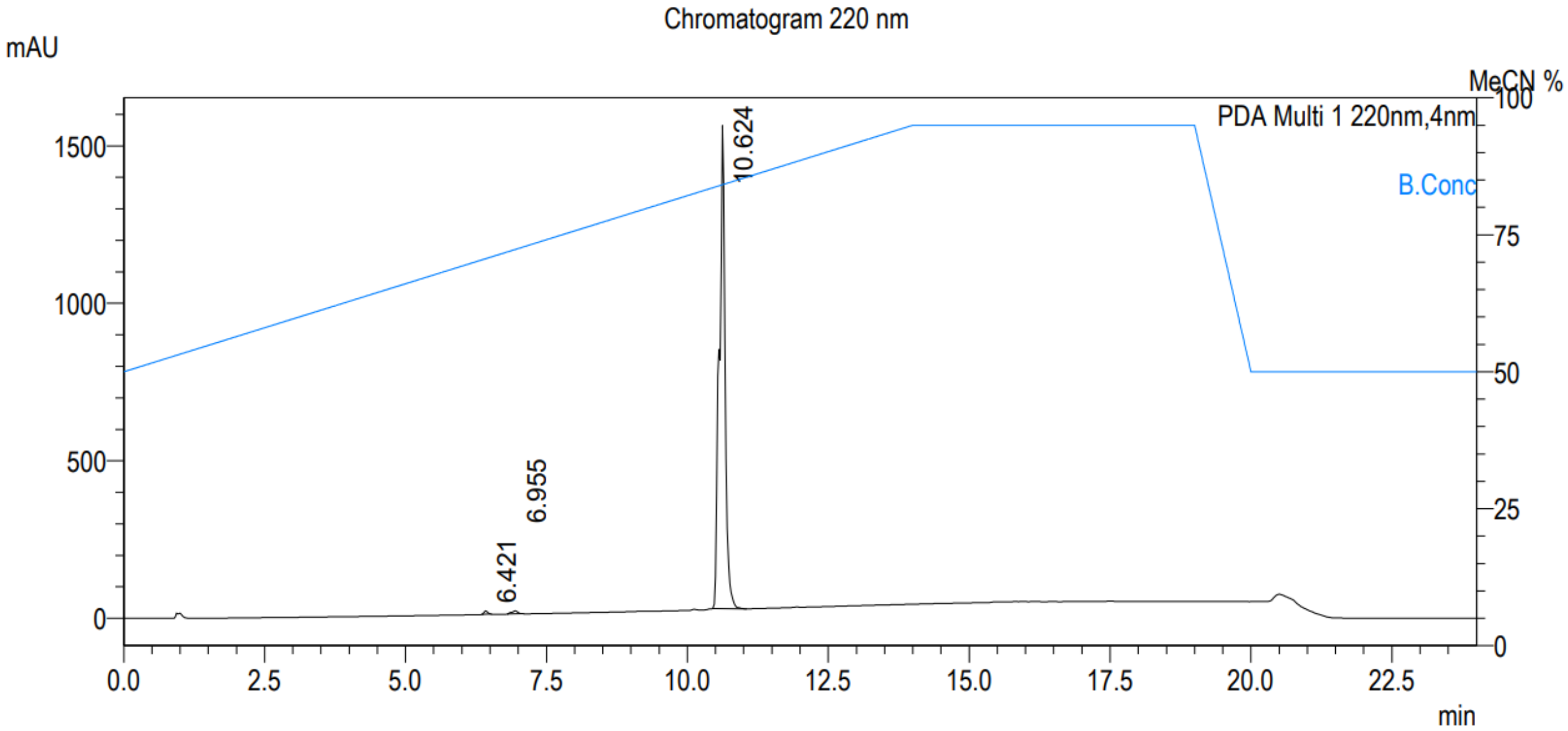

<Peak Table>

Peak Table

PDA Ch1 220nm

Peak#	Ret. Time	Area	Area%
1	6.421	47023	0.414
2	6.955	74726	0.657
3	10.624	11243722	98.929
Total		11365470	100.000

*HPLC trace of **OB-a2***

<Chromatogram>

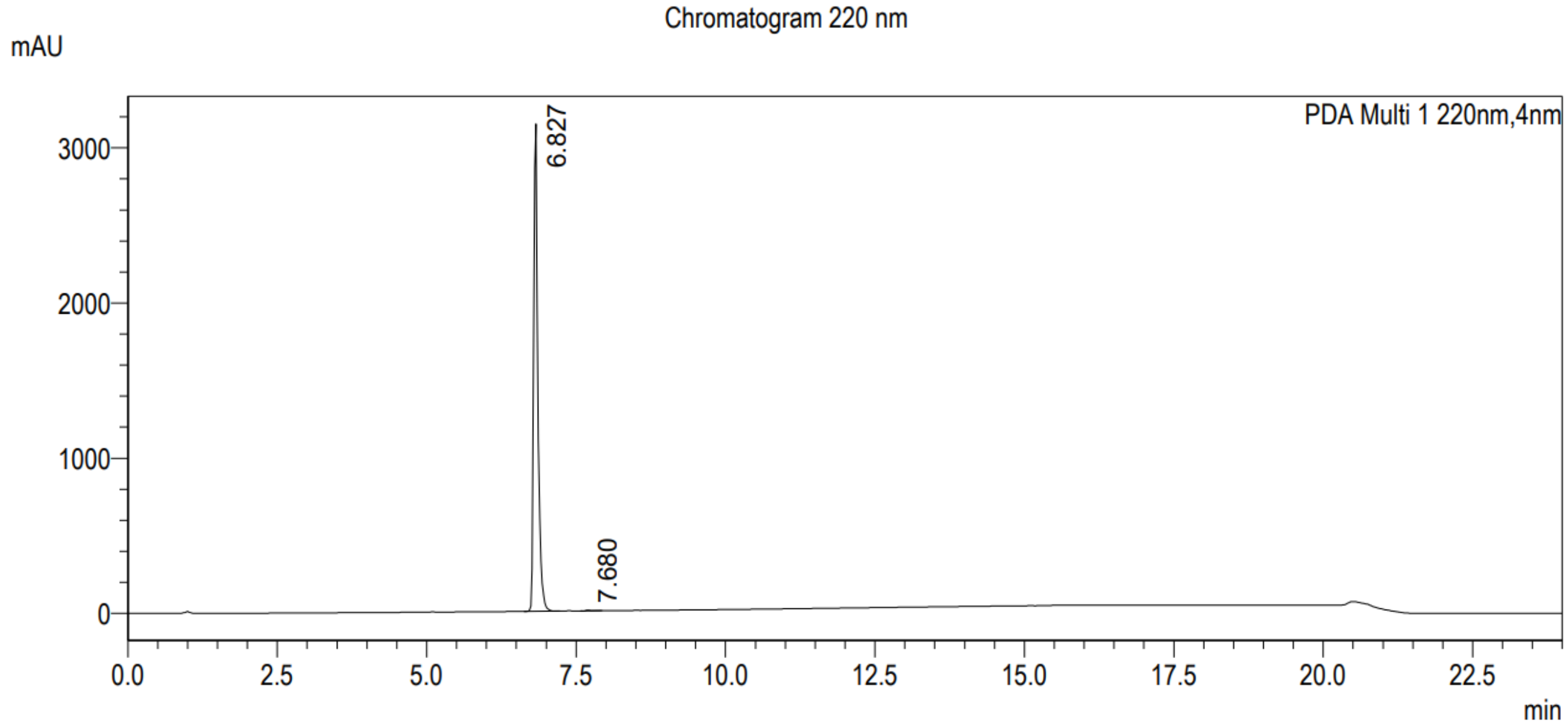

<Peak Table>

Peak Table

PDA Ch1 220nm

Peak#	Ret. Time	Area	Area%
1	6.827	15474181	99.807
2	7.680	29915	0.193
Total		15504096	100.000

*HPLC trace of **OB-a3***

<Chromatogram>

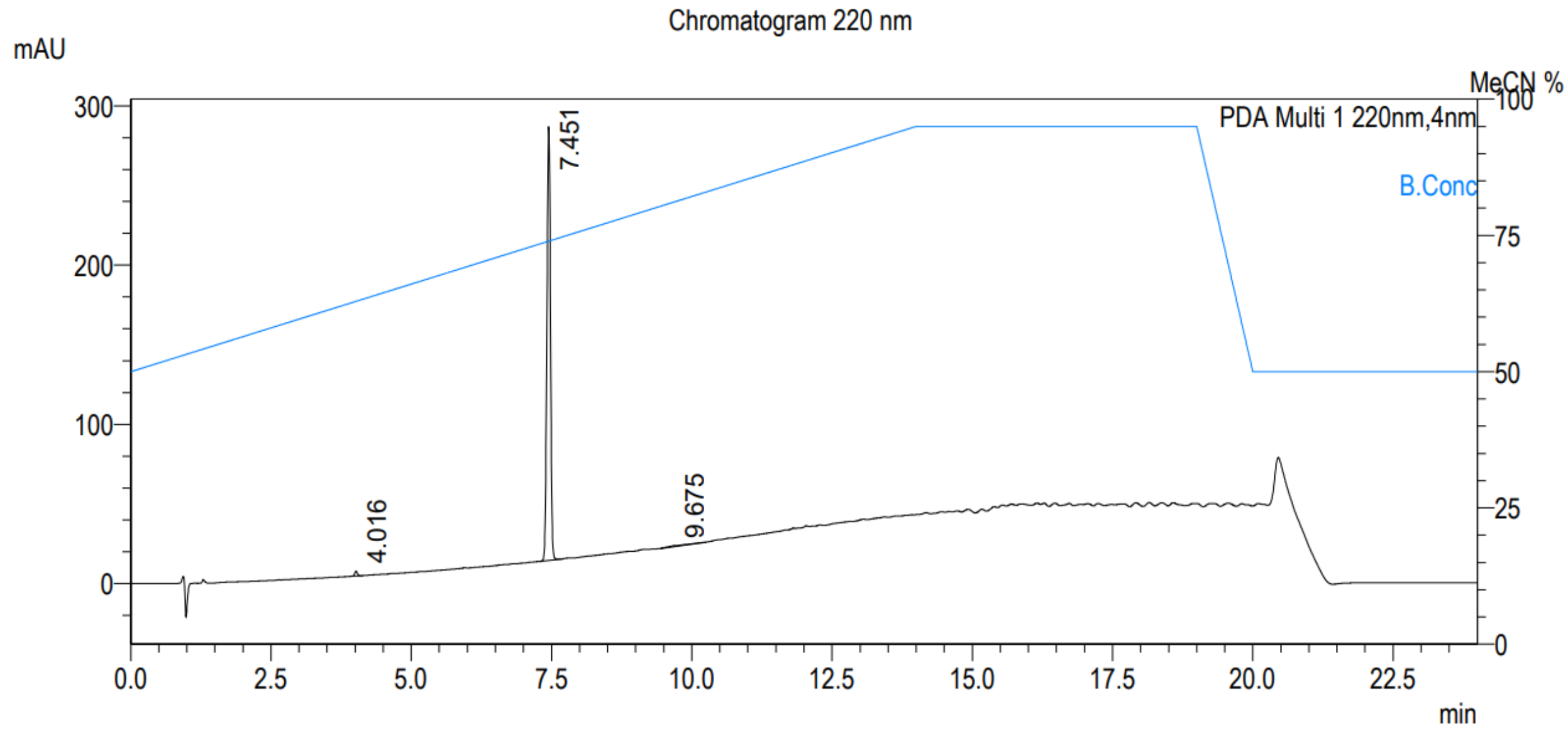

<Peak Table>

Peak Table

PDA Ch1 220nm

Peak#	Ret. Time	Area	Area%
1	4.016	9639	0.801
2	7.451	1181312	98.183
3	9.675	12224	1.016
Total		1203176	100.000

HPLC trace of ***OB-b***

<Chromatogram>

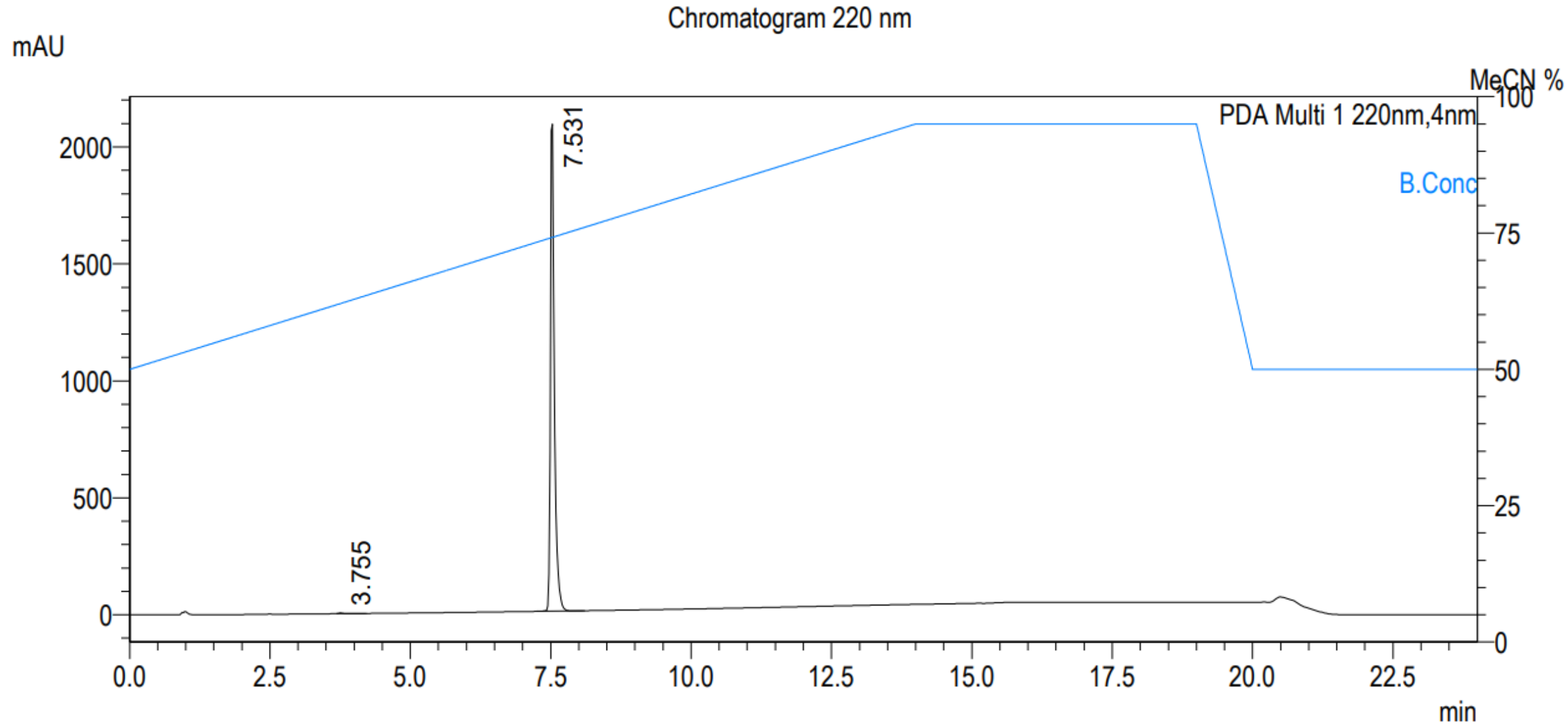

<Peak Table>

Peak Table

PDA Ch1 220nm

Peak#	Ret. Time	Area	Area%
1	3.755	16404	0.157
2	7.531	10458865	99.843
Total		10475269	100.000

HPLC trace of ***OB-b1***

<Chromatogram>

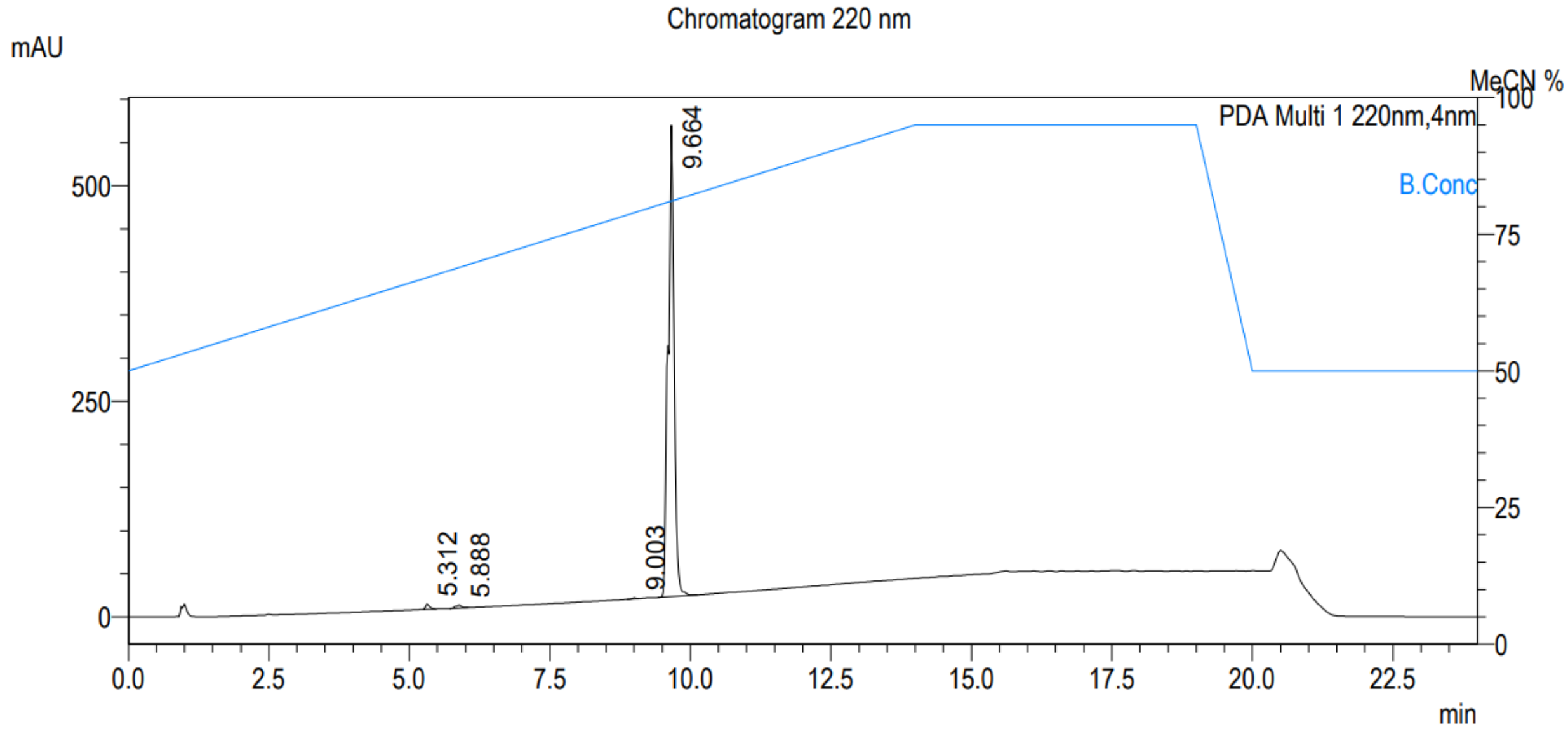

<Peak Table>

Peak Table

PDA Ch1 220nm

Peak#	Ret. Time	Area	Area%
1	5.312	27959	0.692
2	5.888	23842	0.590
3	9.003	3811	0.094
4	9.664	3984790	98.624
Total		4040402	100.000

HPLC trace of ***OB-b2***

<Chromatogram>

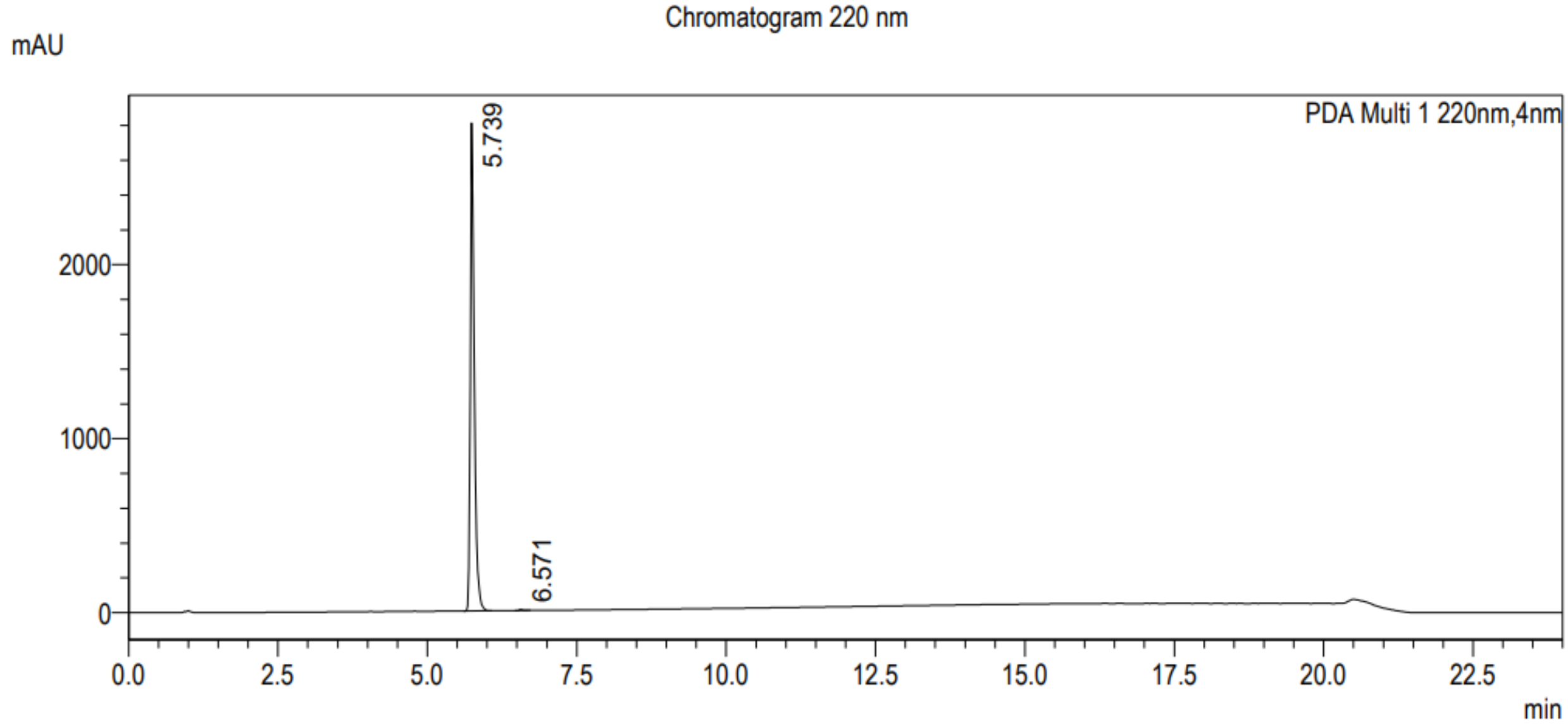

<Peak Table>

Peak Table

PDA Ch1 220nm

Peak#	Ret. Time	Area	Area%
1	5.739	12727975	99.792
2	6.571	26546	0.208
Total		12754520	100.000

*HPLC trace of **OB-b3***

<Chromatogram>

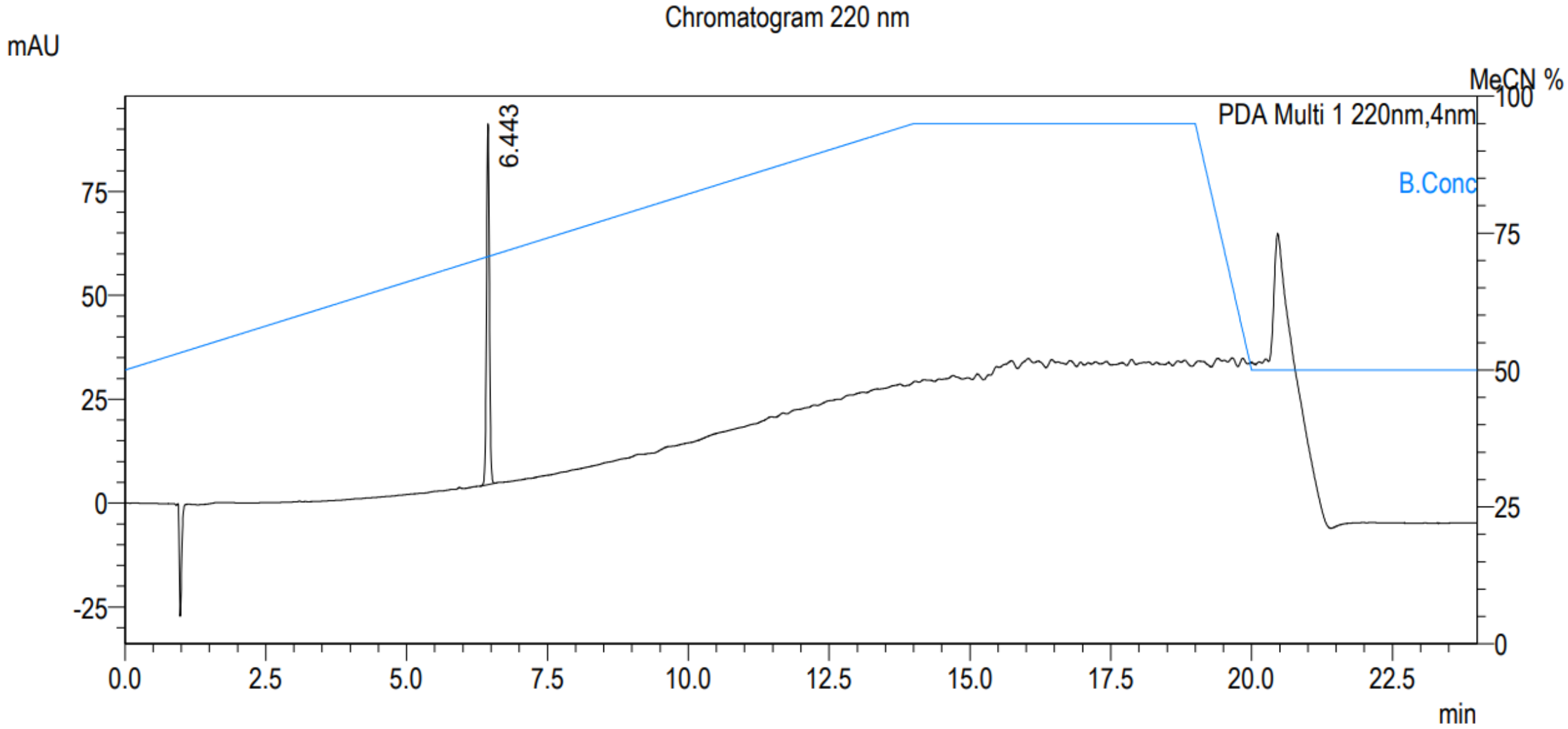

<Peak Table>

Peak Table

PDA Ch1 220nm

Peak#	Ret. Time	Area	Area%
1	6.443	304632	100.000
Total		304632	100.000

*HPLC trace of **PP***

<Chromatogram>

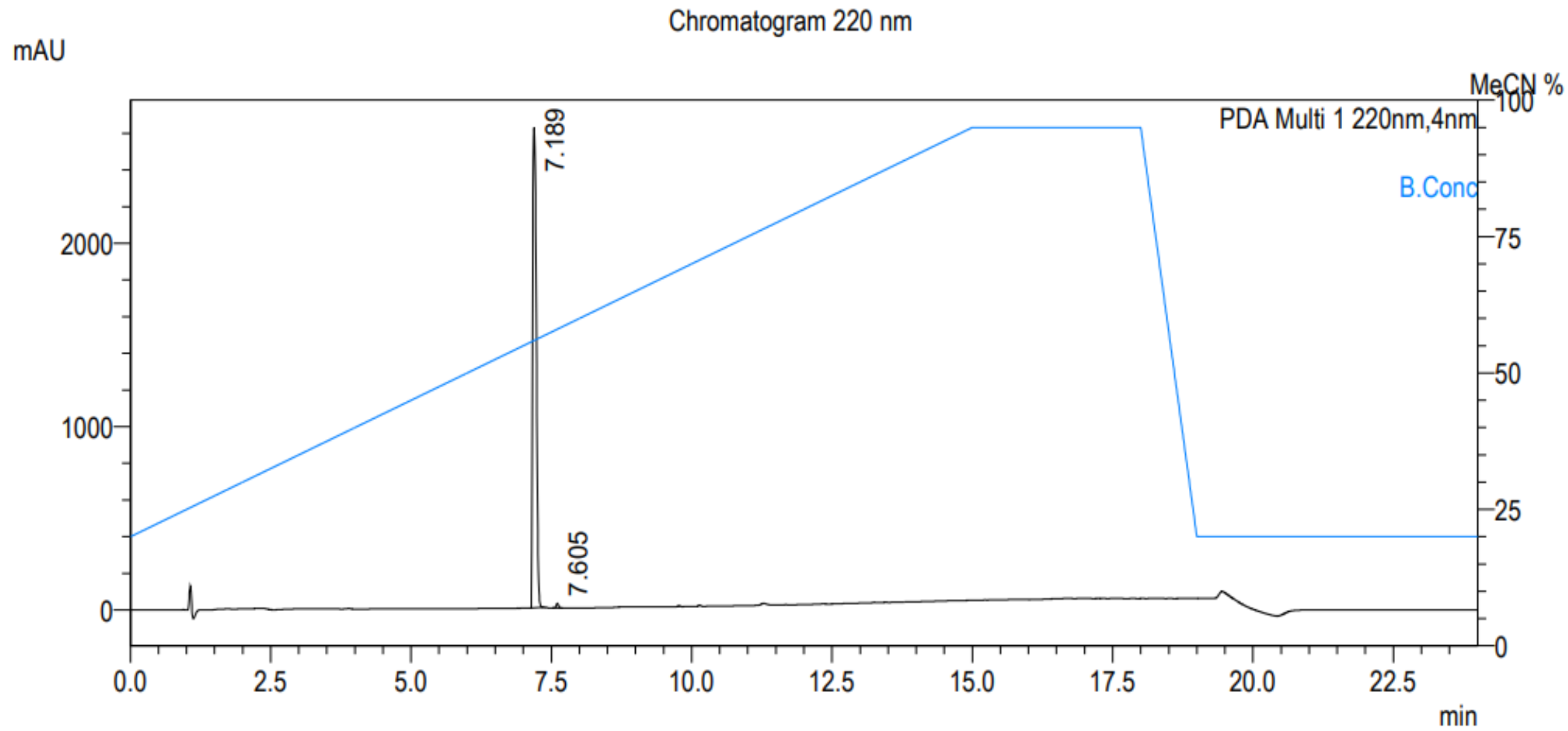

<Peak Table>

Peak Table

PDA Ch1 220nm

Peak#	Ret. Time	Area	Area%
1	7.189	11991400	99.402
2	7.605	72116	0.598
Total		12063516	100.000

*HPLC trace of **PP-1***

<Chromatogram>

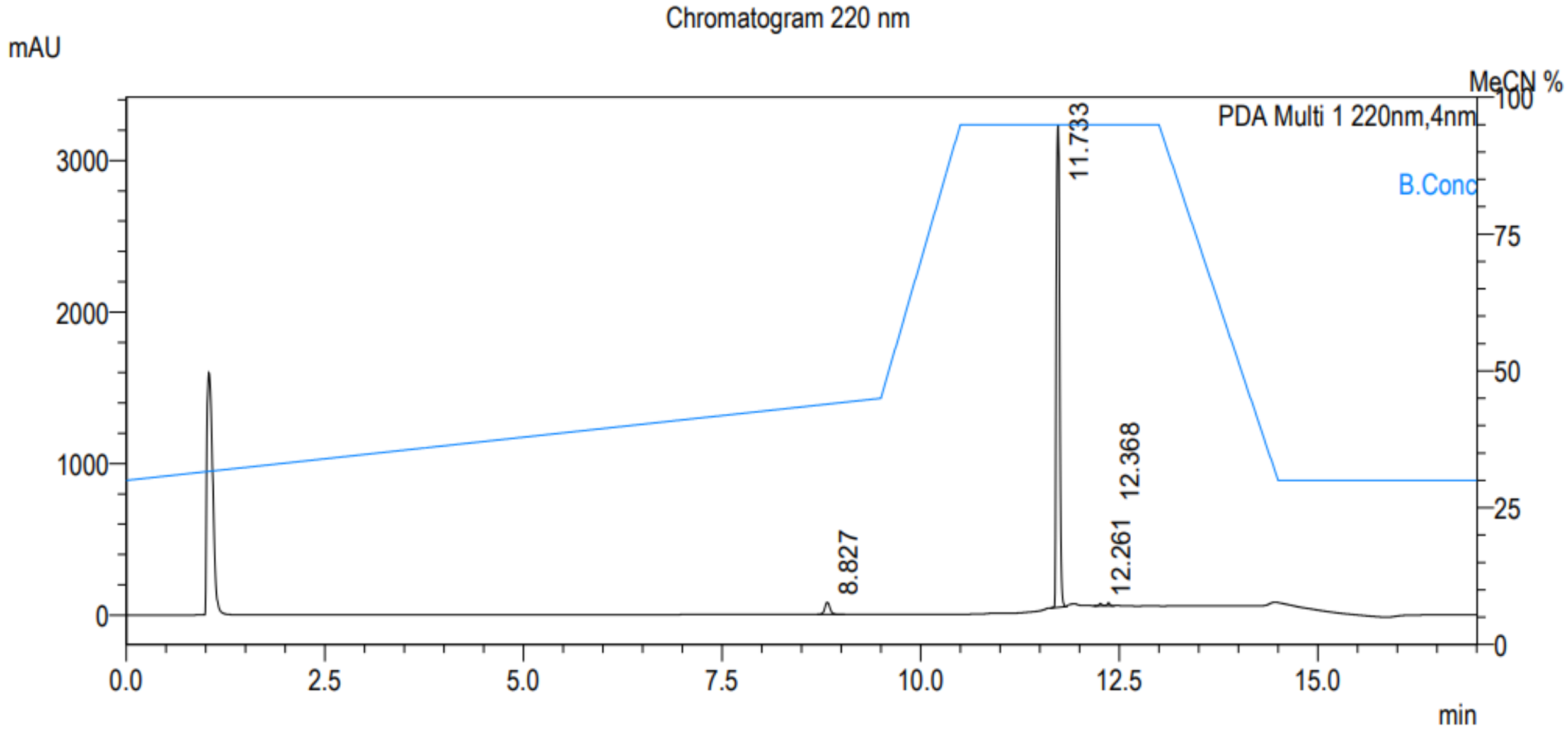

<Peak Table>

Peak Table

PDA Ch1 220nm

Peak#	Ret. Time	Area	Area%
1	8.827	348682	3.593
2	11.733	9269112	95.505
3	12.261	38940	0.401
4	12.368	48621	0.501
Total		9705355	100.000

HPLC trace of ***PP-2***

<Chromatogram>

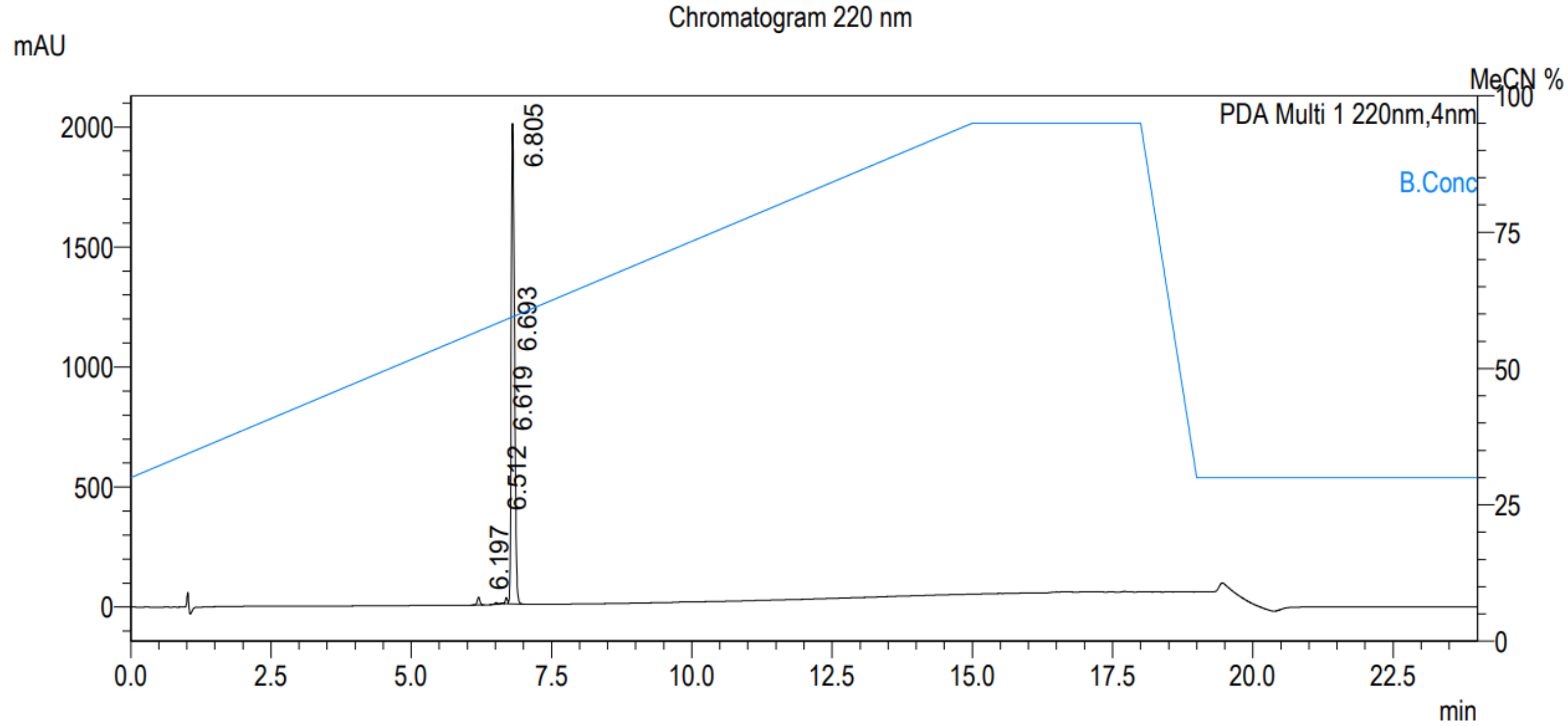

<Peak Table>

Peak Table

PDA Ch1 220nm

Peak#	Ret. Time	Area	Area%
1	6.197	109565	1.315
2	6.512	19905	0.239
3	6.619	5869	0.070
4	6.693	63732	0.765
5	6.805	8130110	97.610
Total		8329180	100.000

*HPLC trace of **OP***

<Chromatogram>

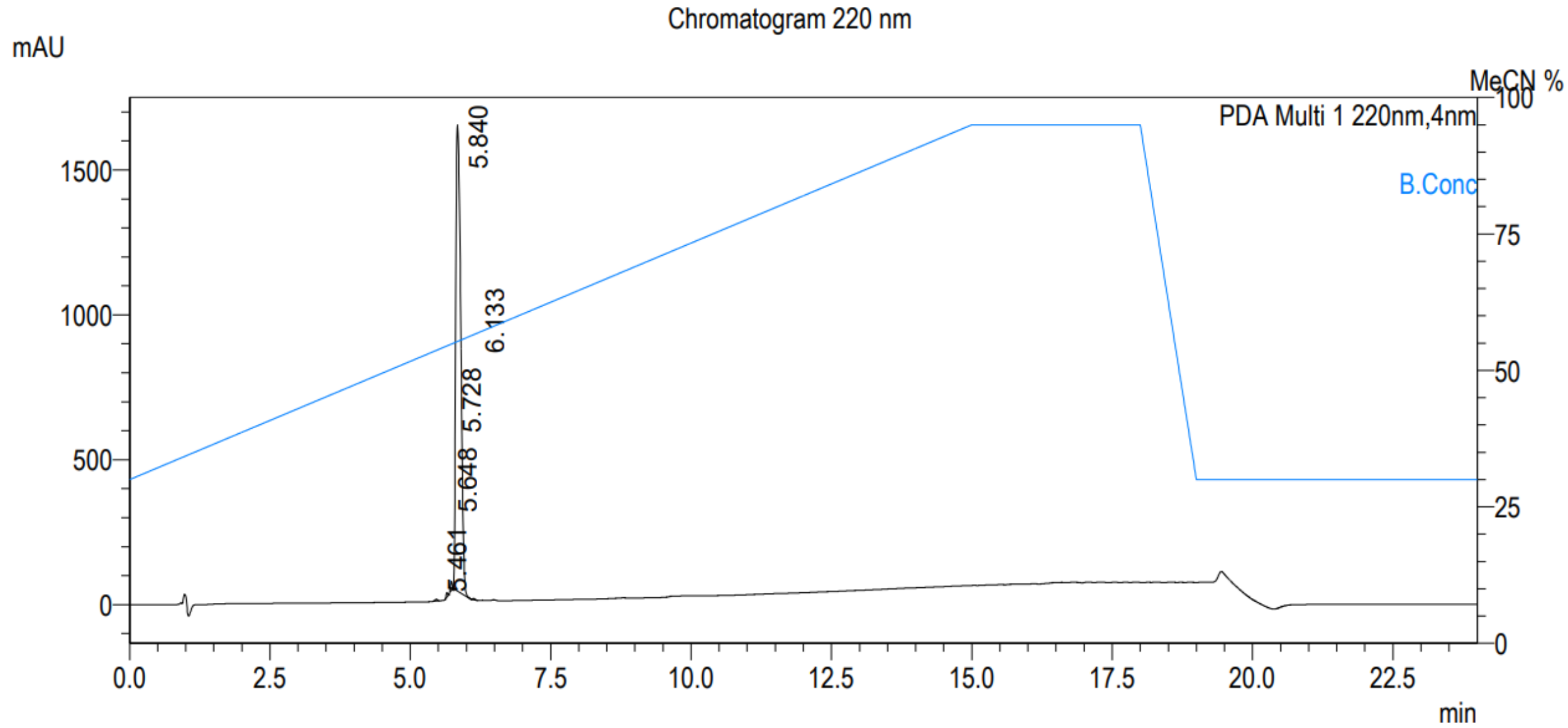

<Peak Table>

Peak Table

PDA Ch1 220nm

Peak#	Ret. Time	Area	Area%
1	5.461	17537	0.173
2	5.648	32159	0.316
3	5.728	61528	0.605
4	5.840	10043068	98.790
5	6.133	11815	0.116
Total		10166108	100.000

*HPLC trace of **OP-1***

<Chromatogram>

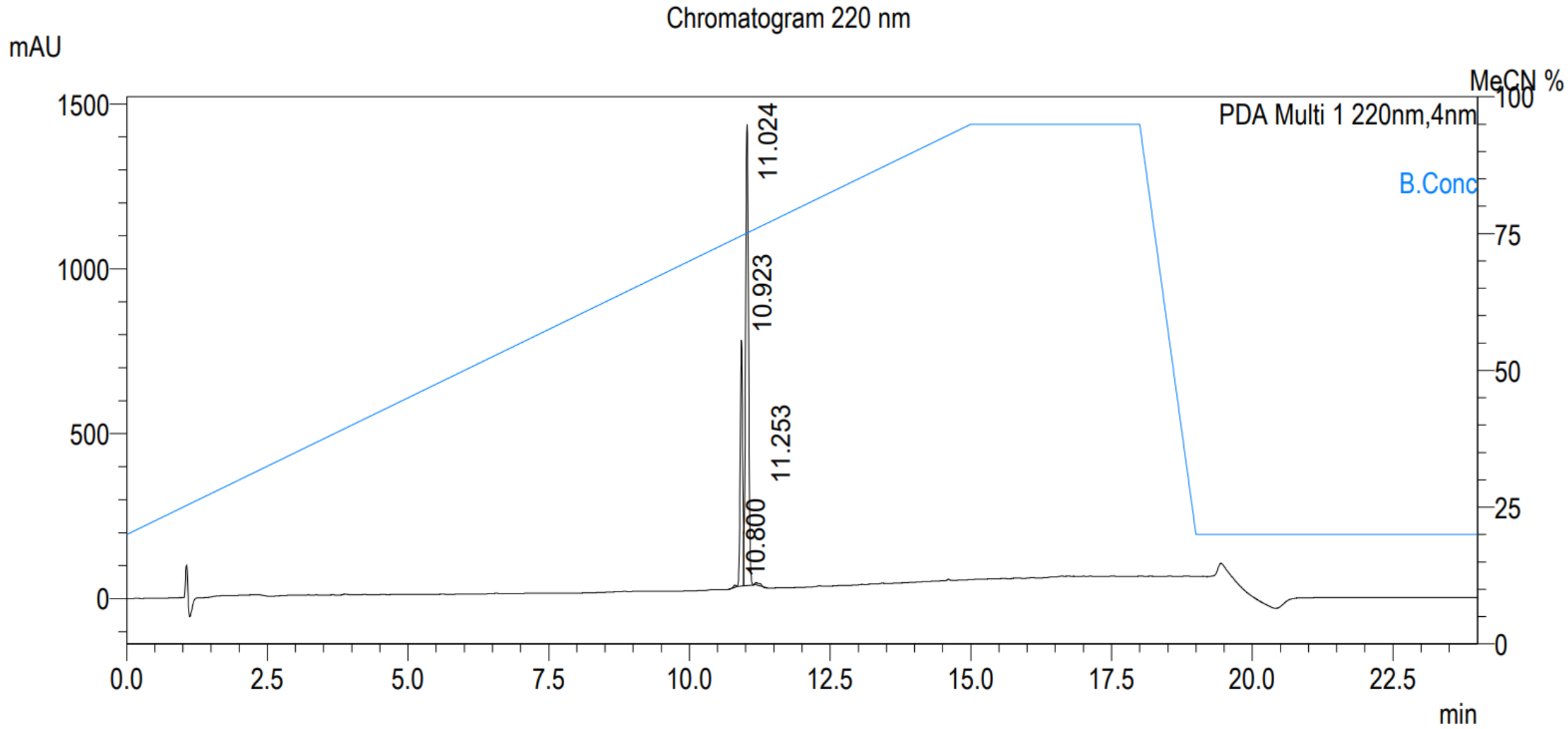

<Peak Table>

Peak Table

PDA Ch1 220nm

Peak#	Ret. Time	Area	Area%
1	10.800	19031	0.257
2	10.923	2192959	29.586
3	11.024	5153274	69.524
4	11.253	47001	0.634
Total		7412265	100.000

Note: Two peaks observed were suspected to be caused by the presence of rotamers.

*HPLC trace of **OP-2***

<Chromatogram>

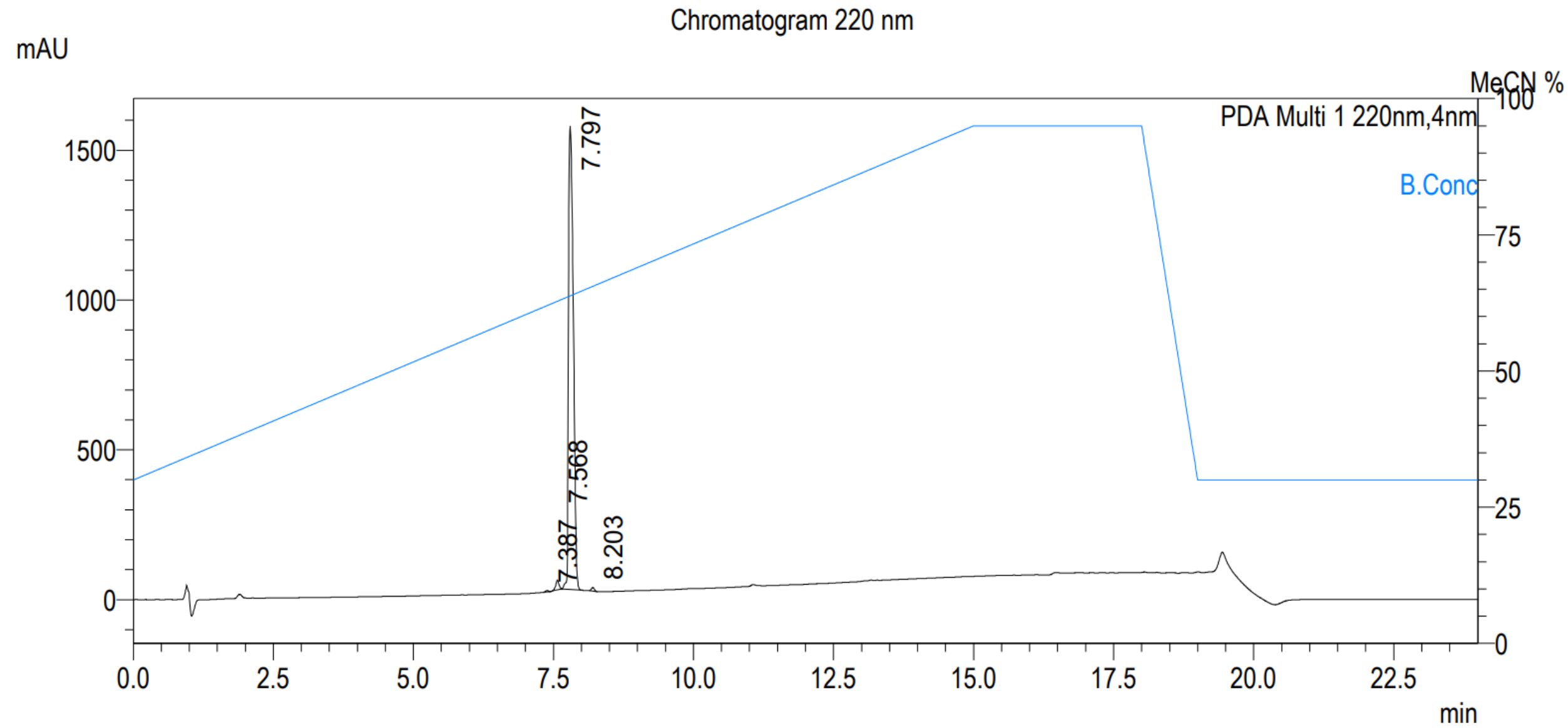

<Peak Table>

Peak Table

PDA Ch1 220nm

Peak#	Ret. Time	Area	Area%
1	7.387	13426	0.141
2	7.568	125774	1.322
3	7.797	9332000	98.085
4	8.203	42996	0.452
Total		9514196	100.000

HPLC trace of ***NP***

<Chromatogram>

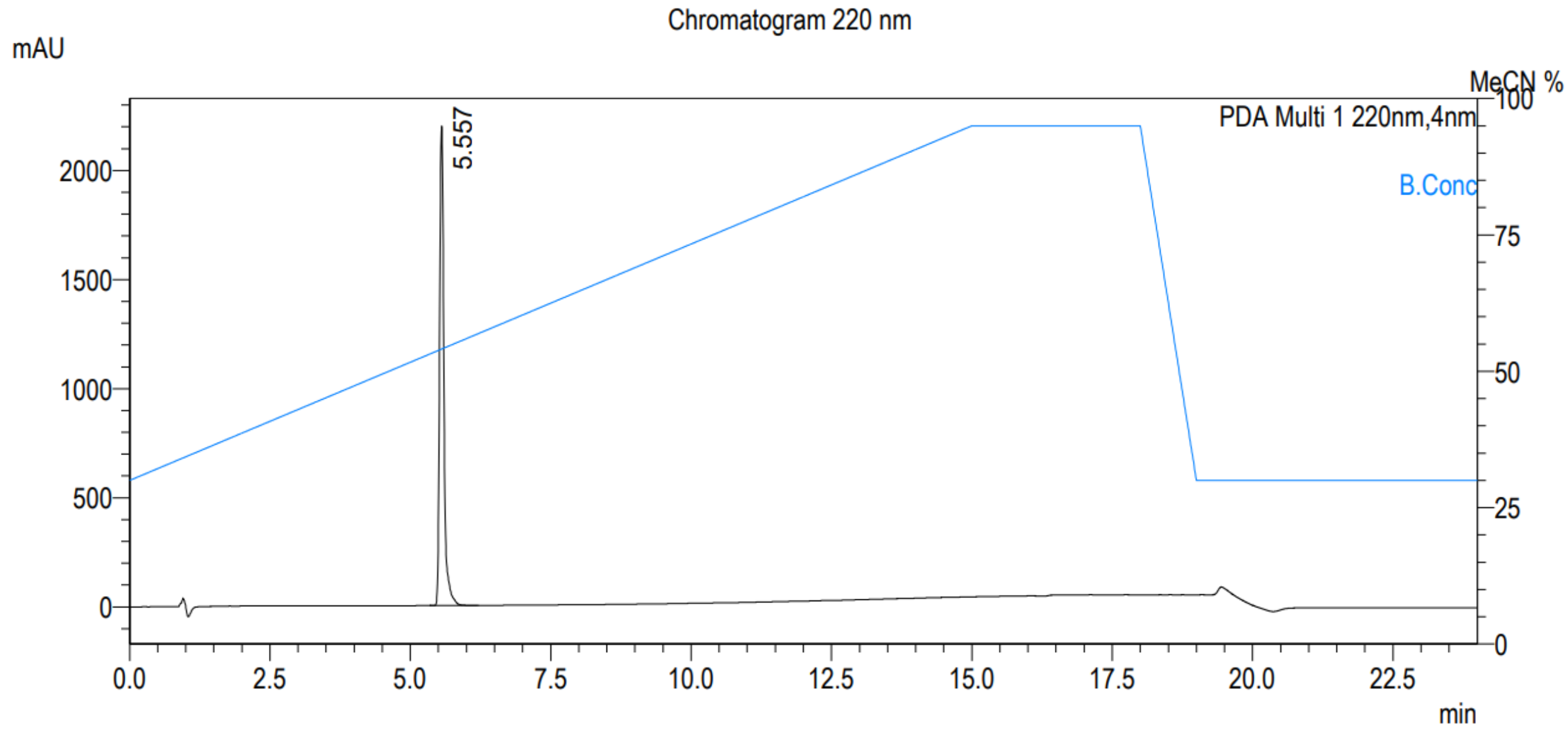

<Peak Table>

Peak Table

PDA Ch1 220nm

Peak#	Ret. Time	Area	Area%
1	5.557	11839380	100.000
Total		11839380	100.000

HPLC trace of Fluorescein-p53

<Chromatogram>

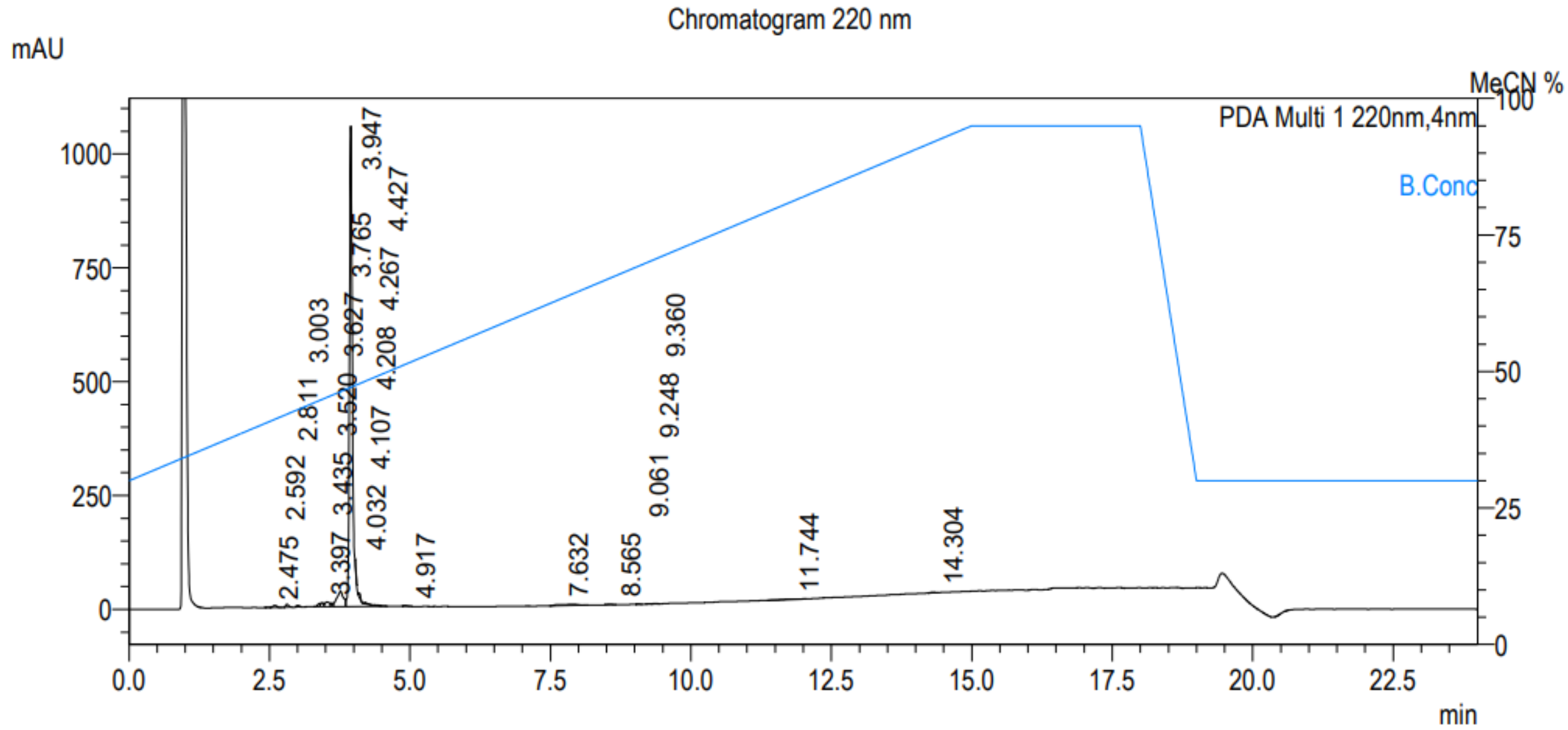

<Peak Table>

Peak Table

PDA Ch1 220nm

Peak#	Ret. Time	Area	Area%
1	2.475	2026	0.043
2	2.592	21503	0.457
3	2.811	22189	0.472
4	3.003	10125	0.215
5	3.397	20108	0.427
6	3.435	34085	0.724
7	3.520	59752	1.270
8	3.627	20787	0.442
9	3.765	272648	5.795
10	3.947	4095481	87.042
11	4.032	5825	0.124
12	4.107	21224	0.451
13	4.208	9869	0.210
14	4.267	3537	0.075
15	4.427	5132	0.109
16	4.917	8046	0.171
17	7.632	57401	1.220
18	8.565	6048	0.129
19	9.061	2645	0.056
20	9.248	2012	0.043
21	9.360	3521	0.075
22	11.744	13581	0.289
23	14.304	7614	0.162
Total		4705157	100.000

Uncropped blots

Figure 2C

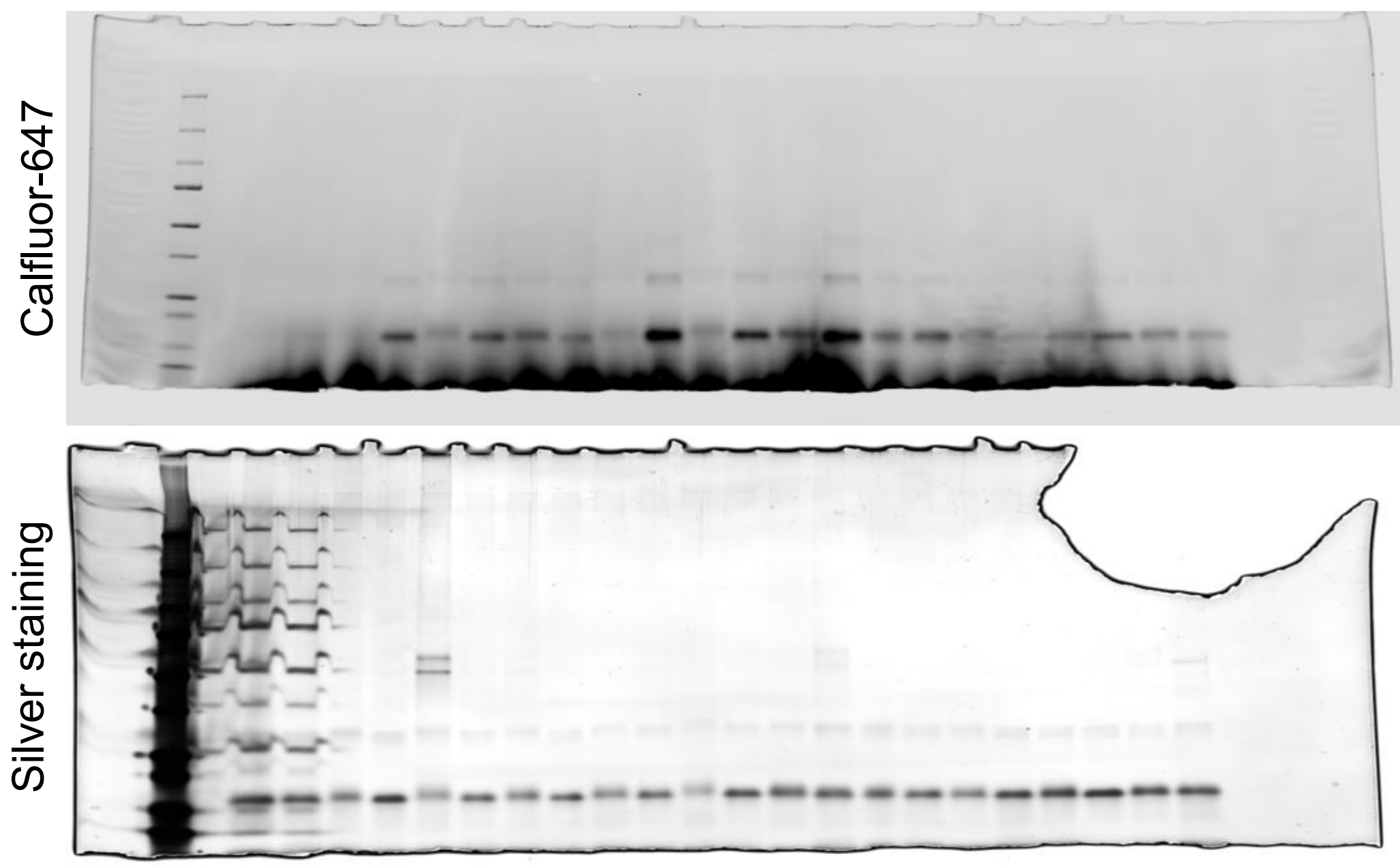

Figure 2D and S3

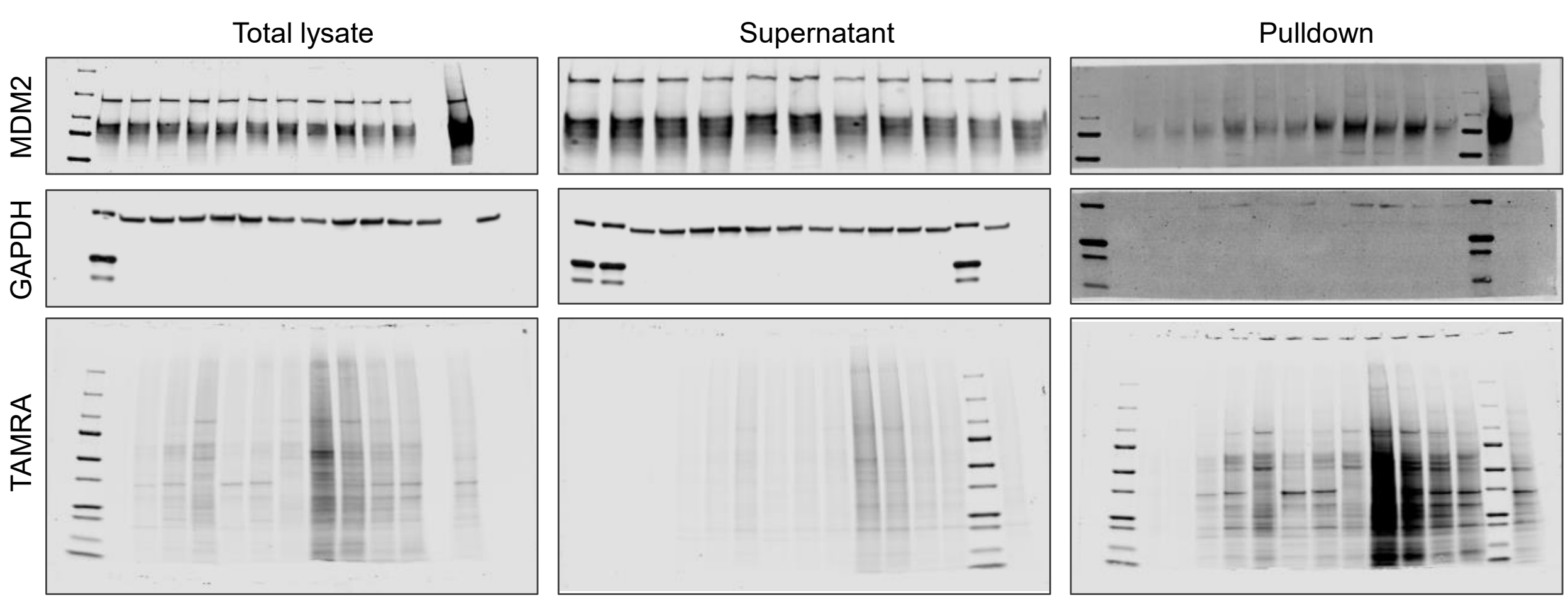

Figure S4A

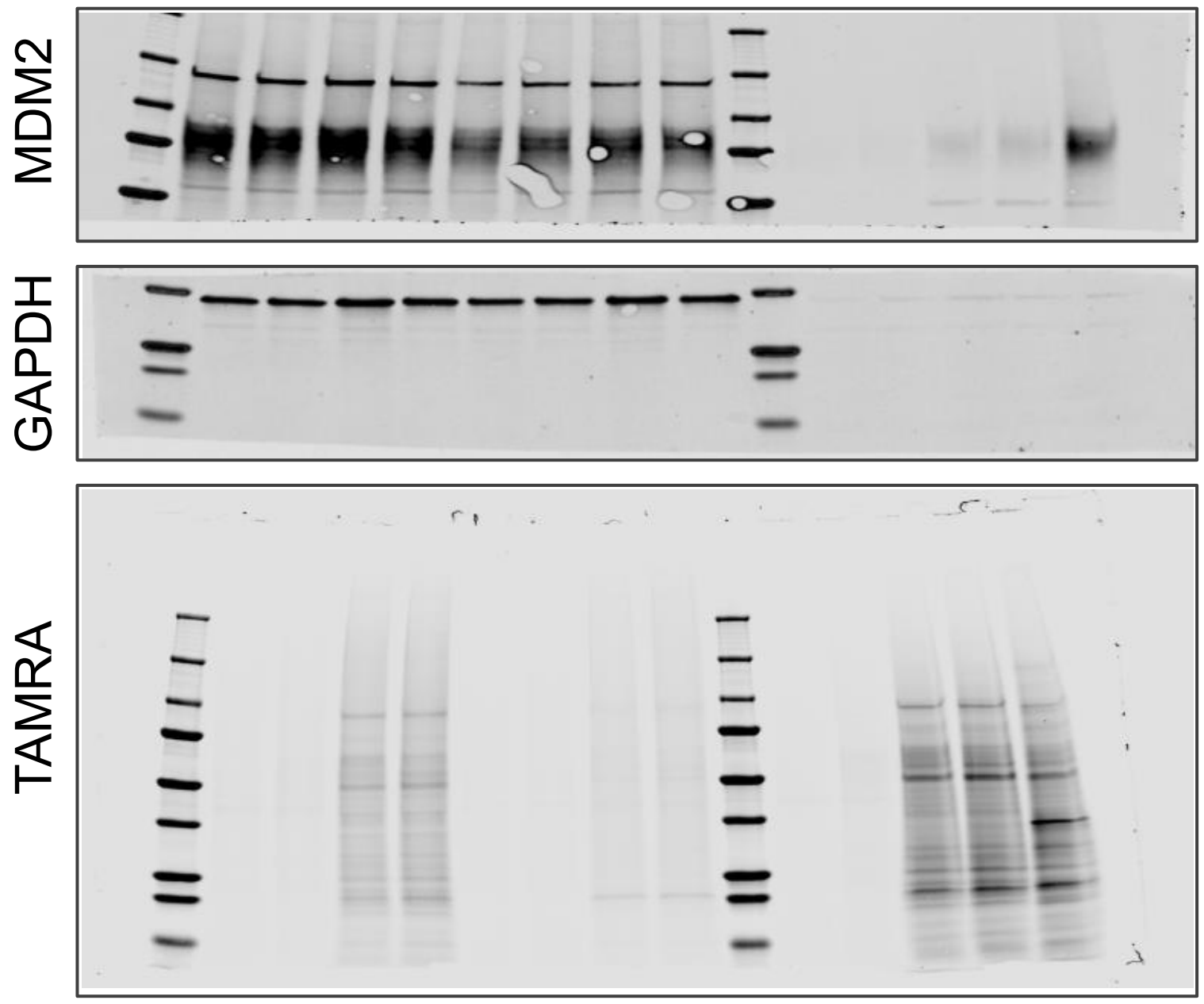

Figure S4B

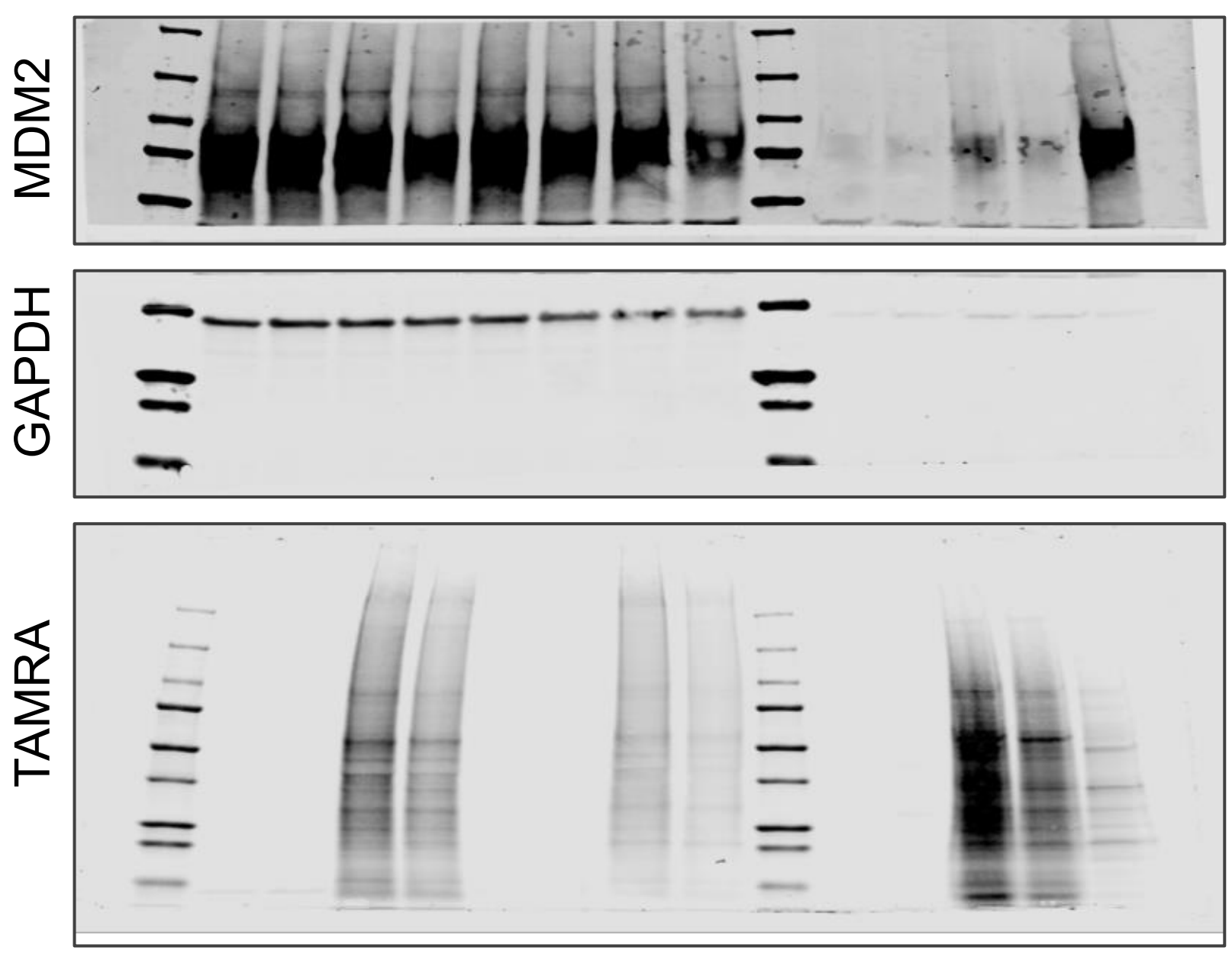

Figure S4C

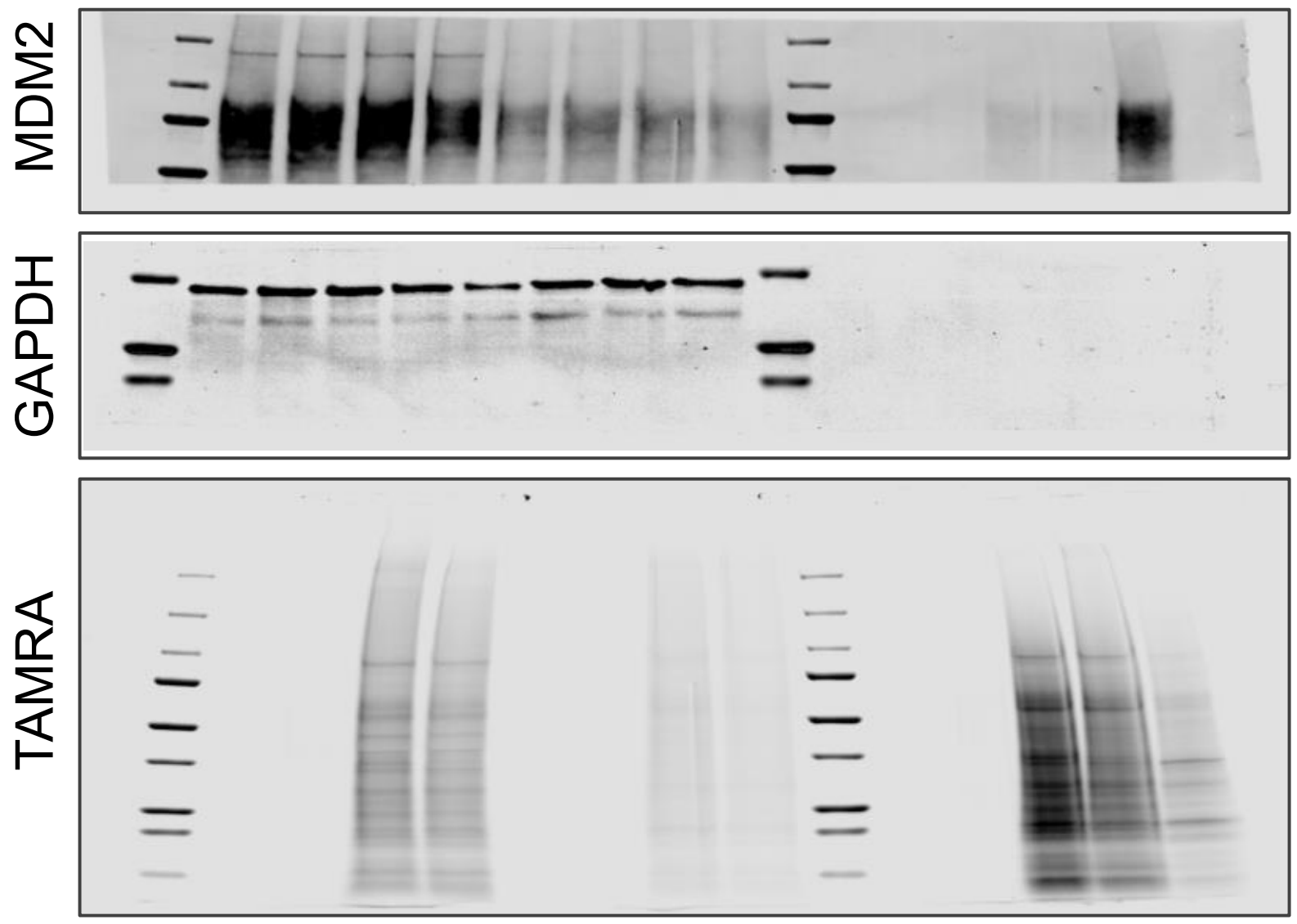

Figure S4D

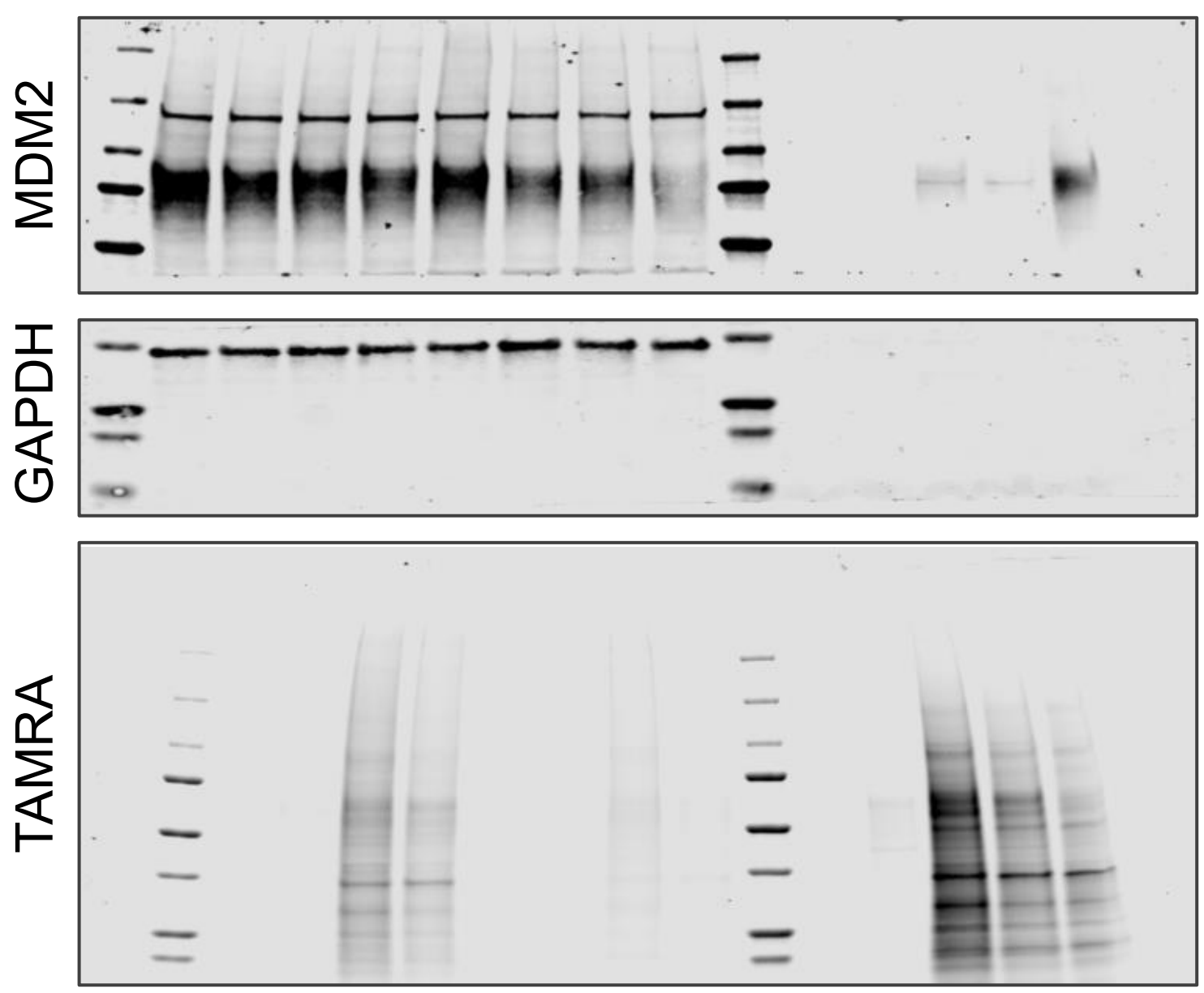

Figure S5F

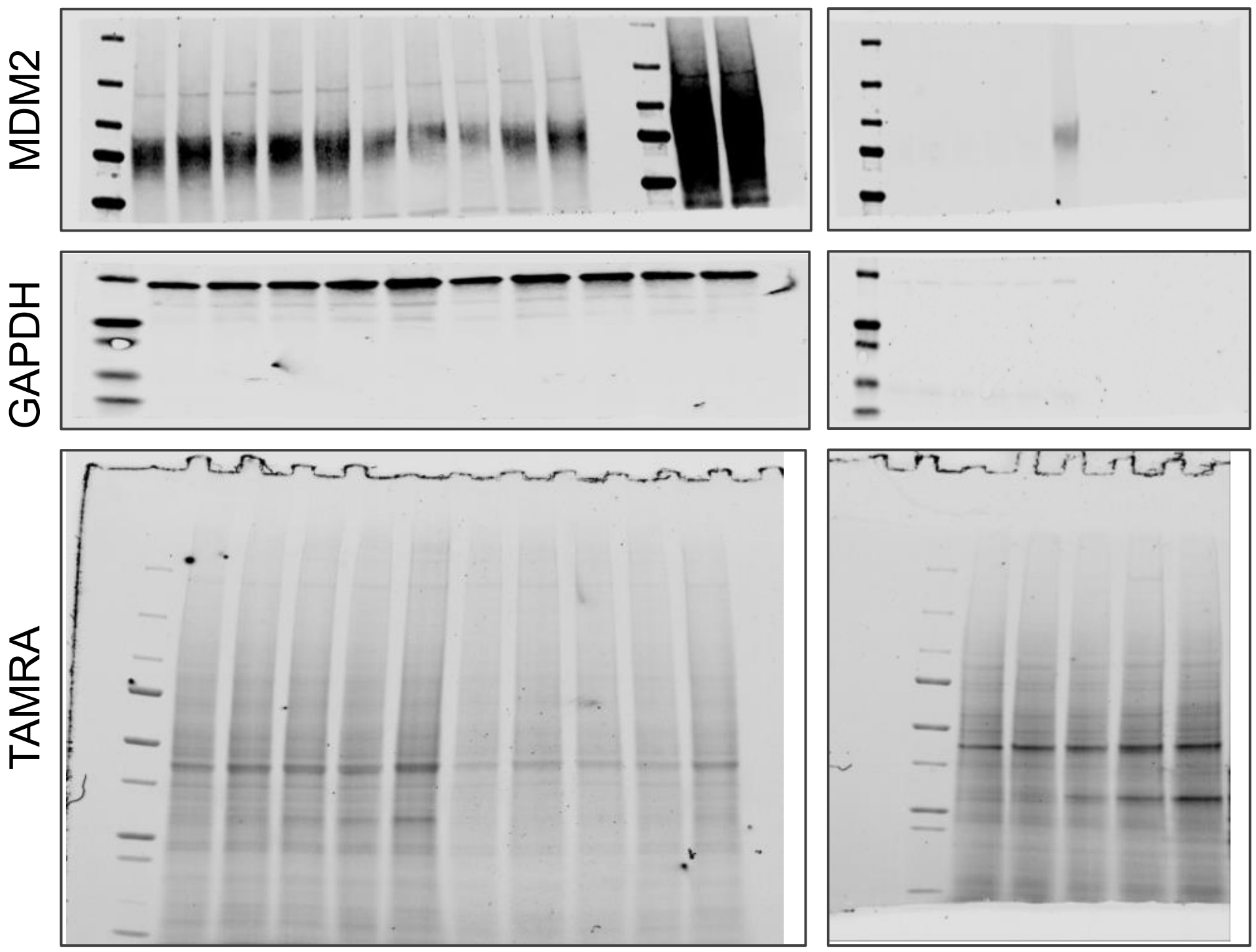

Supplement: Supplementary file 1 [file CB-007-D6CB00065G-s001.pdf]
